# Supplementary material for: Association between use of systemic and inhaled glucocorticoids and changes in brain volume and white matter microstructure: a cross-sectional study using data from the UK Biobank
Source: BMJ Open. 2022 Jul 28;12(8):e062446. doi: 10.1136/bmjopen-2022-062446 (PMC9438037; doi:10.1136/bmjopen-2022-062446)
Supplement: Supplementary data [file bmjopen-2022-062446supp002.pdf]

Supplement 2. Structural or diffusion MRI studies in patients with Cushing disease or exogenous glucocorticoid use

	**Cohort**	**Imaging modality**	**Findings**
**Cushing**			
Andela 2013 [1]	25 CD patients with long-term remission, 25 healthy controls	Volumetric MRI	Patients had reduced GMV in ACC and increased GMV in left posterior lobe of cerebellum. Patients reported more psychological and cognitive symptoms than controls, but these were not associated with GMV changes.
Bauduin 2020 [2]	25 CD patients with long-term remission, 25 healthy controls	Volumetric MRI	Patients had reduced cortical thickness in left caudal ACC, right rostral ACC, left cuneus, left PCC, and bilateral precuneus. Cortical thickness in left caudal ACC and left cuneus were inversely associated with anxiety, depressive symptoms, and disease duration.
Bourdeau 2002 [3]	38 patients with active CS (21 with CD 17 with adrenal CS), 18 patients with other non-ACTH-secreting sellar tumors, 20 normal controls	Volumetric MRI	Overall loss of brain volume and increased ventricle diameters in CS patients. Re-imaging in 22 CS patients at 40 months after correction of hypercortisolism showed a decrease in ventricle diameters and increase in brain volume compared to active disease.
Burkhardt 2015 [4]	19 patients with active untreated CD, 40 healthy controls	Volumetric MRI	CD patients had reduced GMV in hippocampus and cerebellum compared to controls.
Chen 2020 [5]	101 patients with active untreated CD, and 95 patients with NFA (controls)	Volumetric MRI	CD patients had more cortical and subcortical atrophy, more white matter hyperintensities, and decreased hippocampal height. Follow-up of 14 CD patients showed partial reversion of brain atrophy and white matter hyperintensities after correction of hypercortisolism.
Crespo 2014 [6]	35 patients with CS (27 cured, 8 medically treated), 35 controls	Volumetric MRI	No differences were found between cured and treated CS patients. Patients had decreased cortical thickness in the left superior frontal cortex, precentral cortex, left insular cortex, left and right rostral ACC, and right caudal middle frontal cortex compared to controls. Patients also had altered decision-making strategies.
Hou [7]	50 patients with active CD, 36 healthy controls	Volumetric MRI	Patients had reductions in total GMV and frontal, parietal, occipital, and temporal lobes; insula; cingulate lobe; and enlargement of lateral and third ventricles. All affected brain regions improved significantly after TSS. No differences in volume of hippocampus or amygdala.
Jiang 2017 [8]	34 patients with CD (14 with short-term remission, 20 with active CD), 34 controls	Volumetric MRI	Remitted CD patients had greater GMV in bilateral caudate; no differences in GMV of MFG or cerebellum compared to controls. Active CD patients had smaller GMV in MFG and cerebellum compared to controls and remitted patients.
Jiang 2017 [9]	15 patients with active CD, 15 healthy controls	DKI	White matter: increased MD in the splenium of the corpus callosum, bilateral frontal lobe, and left temporal lobe. AD was mainly increased in the bilateral

			frontal lobe, and RD mainly in the left temporal lobe. FA was mainly decreased in the splenium of the corpus callosum and the left temporal lobe.<br>Gray matter: increased MD, RD, and AD in the left hippocampus/parahippocampal gyrus and the left temporal lobe, increased radial kurtosis in the right cerebellar hemisphere, decreased axial kurtosis in the left frontal lobe and decreased mean kurtosis in left cerebellar hemisphere.
Merke 2005 [10]	11 pediatric patients with active CS, 10 healthy controls	Volumetric MRI	CD patients had smaller cerebral volumes, larger ventricles, and a smaller amygdala. One year after surgical cure, cerebral atrophy was reversed, but children showed a decline in IQ and school performance.
Momose 1971 [11]	31 patients with active CD, 64 patients with acromegaly, 36 patients with chromophobe adenoma	Pneumoencephalography	Cerebral cortical atrophy was present in 90% and cerebellar cortical atrophy in 74% of patients with CD. In controls with acromegaly, this was 30% and 3%, respectively. In controls with a chromophobe adenoma, this was 58% and 20%, respectively.
Pires 2015 [12]	35 patients with CS (8 active hypercortisolism, 7 medication-remitted cortisol, 20 surgically cured), 35 healthy controls	DTI	Patients had widespread alteration in white matter integrity (increased FA, decreased MD, RD, AD) compared to controls. Both active and cured CS patients showed increased FA, and decreased MD, RD, and AD; medically treated CS patients did not have significantly different AD values.
Pires 2017 [13]	35 patients with CS (8 active hypercortisolism, 7 medication-remitted cortisol, 20 surgically cured), 35 healthy controls	DTI	All patient groups had more depression and anxiety than controls. Depression scores correlated negatively to FA (in right corticospinal tract (CST), forceps major, forceps minor, left inferior fronto-occipital fasciculus (IFOF) (frontal part), right IFOF, right inferior longitudinal fasciculus (ILF), left superior longitudinal fasciculus (SLF) (anterior part) and right SLF), and positively to RD values (in frontal regions of the forceps minor and frontal areas of bilateral IFOFs). Although processing speed did not differ between groups, Symbol Digit Modalities Test scores correlated positively to both FA and AD values.
Resmini 2012 [14]	33 patients with CS (11 active, 22 cured), 34 controls	Volumetric MRI	Patients had reduced total and cortical brain gray matter volumes compared with controls. Subcortical gray matter (which includes hippocampal volume) was reduced only in 12 patients with severe memory impairment. No differences in hippocampal volume were reported between patients with active or cured CS.
Santos 2014 [15]	36 patients with CS (15 active, 21 in remission), 36 controls	Volumetric MRI	Patients with active CS had smaller cerebellar cortex volumes, and patients with remitted CS showed a similar trend. Cerebellar white matter volume showed no differences.
Santos 2015 [16]	38 patients with CS (15 active, 23 in remission), 38 controls	Volumetric MRI	Patients in remission had more white matter lesions than controls and active patients. Both CS groups had reduced total brain volume and GMV. No differences were found in white matter volume.

Santos 2017 [17]	39 patients with CS (16 active, 23 in remission), 39 healthy controls	Volumetric MRI	Active CS patients had smaller right amygdala volumes. Left amygdala volume was associated with depression and anxiety scores. No differences were found between patients in remission and controls.
Simmons 2000 [18]	63 patients with CD (all after surgical treatment), 63 controls with sellar pathology other than ACTH-secreting tumors	Volumetric MRI	CD patients had higher degrees of cerebral atrophy than controls.
Starkman 1992 [19]	12 patients with CS	Volumetric MRI	For 27% of patients, hippocampal volume fell outside the 95% confidence interval of the population. Plasma cortisol was negatively correlated with hippocampal volume.
Starkman 1999 [20]	22 patients with active CD	Volumetric MRI	Sixteen months after TSS, hippocampus volume increased up to 10%, and a smaller increase was observed in caudate volume.
Starkman 2003 [21]	24 patients with active CD	Volumetric MRI	Sixteen months after TSS, all patients showed an increase in hippocampal volume (which was significantly correlated with lower cortisol levels, and with one neuropsychological test), and 18 patients had an increase in caudate head volume.
Tirosh 2020 [22]	29 patients with CS (8 active, 21 recovering), 8 controls	Volumetric MRI	Patients with persistent disease had increased white matter volume and decreased cortex thickness and white matter intensity compared with patients achieving remission of CS, mainly in frontal and parietal lobes (but not FDR-corrected). Compared to healthy controls, patients recovering from CS had a decrease in subcortical GM volume, an increase in cortical thickness, and a decrease in white matter volume in multiple sites (including accumbens).<br>In all patients together, 24h UFC correlated negatively with intensity in caudate, hippocampus, accumbens, and corpus callosum; correlated negatively with white matter intensity in frontal and parietal lobes; and positively with lateral ventricles volumes. Changes in 24h UFC correlated negatively with change in total brain volume, supratentorium, cerebellar cortex, and putamen.
Toffanin 2011 [23]	10 patients with active CD	Volumetric MRI	After TSS, the volume of the hippocampal head increased significantly, but no change in hippocampal body or tail, nor in whole brain volume was observed.
Van der Werff 2014 [24]	22 patients with long-term remission of CD, 22 healthy controls	DTI	Patients had widespread FA reductions in whole brain analysis. ROI analysis revealed reduced FA in the bilateral cingulate cingulum, bilateral uncinate fasciculus and corpus callosum. No significant differences were found in tracts in the inferior parts of the brainstem, the white matter in the bilateral cerebellum, the bilateral hippocampal cingulum, the left inferior fronto-occipital fasciculus, and parts of the bilateral superior longitudinal fasciculus. Patients also had increased radial and mean diffusivity, but no difference in axial diffusivity.

**Exogenous GC**			
Bentson 1978 [25]	15 long-term GC users	CT	Patients showed varying degrees of apparent cerebral atrophy. Some correlation between dosage and degree of atrophy appeared to be present.
Brown 2004 [26]	17 chronic (>6 months) GC (prednisone) users, 15 controls	Volumetric MRI, PMRS	GC users had smaller hippocampal volume, lower N-acetyl aspartate ratios, more mood symptoms and poorer cognitive function.
Brown 2015 [27]	17 healthy adults who received hydrocortisone (160 mg/day)/placebo, phenytoin/placebo, hydrocortisone/phenytoin, or placebo/placebo, in a randomized, blinded, cross-over trial with 21-day washout between conditions.	Volumetric MRI	Hydrocortisone use was not associated with difference in total brain volume but was associated with a 1.69% reduction in total hippocampal volume compared to placebo. Phenytoin blocked this hippocampal volume reduction by hydrocortisone.
Brown 2019 [28]	46 chronic GC users, randomized to memantine or placebo in blinded, cross-over trial (two 24-week treatment periods, separated by four-week washout)	Volumetric MRI	Hippocampal volume decreased significantly from baseline to week 52 and from week 24 to week 52, without significant difference between baseline and week 24. Following 24 weeks of memantine, left dentate gyrus/CA3 volume was significantly larger than after placebo; a similar trend was observed in the right CA1. Subiculum showed no significant differences.
Brown 2008 [29]	15 chronic (>6 months) GC (prednisone) users, 13 controls	Volumetric MRI	GC users had significantly smaller amygdala volumes compared to controls. Duration of GC therapy correlated negatively with right amygdala volume.
Desai 2009 [30]	28 chronic (>6 months) GC (prednisone) users, randomized to 24 weeks of lamotrigine (n = 16) or placebo (n = 12) in blinded trial	Volumetric MRI	After 24 weeks, amygdala volume was reduced in both groups, but right amygdala volume was significantly less reduced in the lamotrigine group than in the placebo group.
Nguyn 2019 [31]	81 chronic (>6 months) GC (prednisone) users	Volumetric MRI	Cumulative GC exposure negatively associated with the volumes of the left and right hippocampal dentate gyrus/CA3; no associations were found for entorhinal, perirhinal, or parahippocampal gyri, subiculum, or CA1.

AD, axial diffusivity, ACC, anterior cingulate cortex; CD, Cushing disease; CS, Cushing syndrome; CT, computed tomography; FA, fractional anisotropy; DKI, diffusional kurtosis imaging; DMN, default mode network; GC, glucocorticoids; GMV, grey matter volume; MD, mean diffusivity; MFG, medial frontal gyrus; NFA, non-functioning pituitary adenoma; PCC, posterior cingulate cortex; PMRS, proton magnetic resonance spectroscopy; RD, radial diffusivity; RSFC, resting-state functional connectivity; TSS, transsphenoidal surgery; 24h UFC, 24-hour urinary free cortisol.

References

1. Andela, C.D., van der Werff, S.J., Pannekoek, J.N., van den Berg, S.M., Meijer, O.C., van Buchem, M.A., Rombouts, S.A., van der Mast, R.C., Romijn, J.A., Tiemensma, J., Biermasz, N.R., van der Wee, N.J., Pereira, A.M.: Smaller grey matter volumes in the anterior cingulate cortex and greater cerebellar volumes in patients with long-term remission of Cushing's disease: a case-control study. Eur J Endocrinol **169**(6), 811-819 (2013). doi:10.1530/eje-13-0471
2. Bauduin, S., van der Pal, Z., Pereira, A.M., Meijer, O.C., Giltay, E.J., van der Wee, N.J.A., van der Werff, S.J.A.: Cortical thickness abnormalities in long-term remitted Cushing's disease. Transl Psychiatry **10**(1), 293 (2020). doi:10.1038/s41398-020-00980-6
3. Bourdeau, I., Bard, C., Noël, B., Leclerc, I., Cordeau, M.P., Bélair, M., Lesage, J., Lafontaine, L., Lacroix, A.: Loss of brain volume in endogenous Cushing's syndrome and its reversibility after correction of hypercortisolism. J Clin Endocrinol Metab **87**(5), 1949-1954 (2002). doi:10.1210/jcem.87.5.8493
4. Burkhardt, T., Lüdecke, D., Spies, L., Wittmann, L., Westphal, M., Flitsch, J.: Hippocampal and cerebellar atrophy in patients with Cushing's disease. Neurosurg Focus **39**(5), E5 (2015). doi:10.3171/2015.8.Focus15324
5. Chen, Y., Zhang, J., Tan, H., Li, J., Yu, Y.: Detrimental effects of hypercortisolism on brain structure and related risk factors. Sci Rep **10**(1), 12708 (2020). doi:10.1038/s41598-020-68166-0
6. Crespo, I., Esther, G.M., Santos, A., Valassi, E., Yolanda, V.G., De Juan-Delago, M., Webb, S.M., Gómez-Ansón, B., Resmini, E.: Impaired decision-making and selective cortical frontal thinning in Cushing's syndrome. Clinical endocrinology **81**(6), 826-833 (2014).
7. Hou, B., Gao, L., Shi, L., Luo, Y., Guo, X., Young, G.S., Qin, L., Zhu, H., Lu, L., Wang, Z., Feng, M., Bao, X., Wang, R., Xing, B., Feng, F.: Reversibility of impaired brain structures after transsphenoidal surgery in Cushing's disease: a longitudinal study based on an artificial intelligence-assisted tool. J Neurosurg, 1-10 (2020). doi:10.3171/2019.10.Jns191400
8. Jiang, H., Ren, J., He, N.Y., Liu, C., Sun, Y.H., Jian, F.F., Bian, L.G., Shen, J.K., Yan, F.H., Pan, S.J., Sun, Q.F.: Volumetric magnetic resonance imaging analysis in patients with short-term remission of Cushing's disease. Clin Endocrinol (Oxf) **87**(4), 367-374 (2017). doi:10.1111/cen.13381
9. Jiang, H., He, N.Y., Sun, Y.H., Jian, F.F., Bian, L.G., Shen, J.K., Yan, F.H., Pan, S.J., Sun, Q.F.: Altered gray and white matter microstructure in Cushing's disease: A diffusional kurtosis imaging study. Brain Res **1665**, 80-87 (2017). doi:10.1016/j.brainres.2017.04.007
10. Merke, D.P., Giedd, J.N., Keil, M.F., Mehlinger, S.L., Wiggs, E.A., Holzer, S., Rawson, E., Vaituzis, A.C., Stratakis, C.A., Chrousos, G.P.: Children experience cognitive decline despite reversal of brain atrophy one year after resolution of Cushing syndrome. J Clin Endocrinol Metab **90**(5), 2531-2536 (2005). doi:10.1210/jc.2004-2488
11. Momose, K.J., Kjellberg, R.N., Kliman, B.: High incidence of cortical atrophy of the cerebral and cerebellar hemispheres in Cushing's disease. Radiology **99**(2), 341-348 (1971). doi:10.1148/99.2.341
12. Pires, P., Santos, A., Vives-Gilabert, Y., Webb, S.M., Sainz-Ruiz, A., Resmini, E., Crespo, I., de Juan-Delago, M., Gómez-Anson, B.: White matter alterations in the brains of patients with active, remitted, and cured cushing syndrome: a DTI study. AJNR Am J Neuroradiol **36**(6), 1043-1048 (2015). doi:10.3174/ajnr.A4322

13. Pires, P., Santos, A., Vives-Gilabert, Y., Webb, S.M., Sainz-Ruiz, A., Resmini, E., Crespo, I., de Juan-Delago, M., Gómez-Anson, B.: White matter involvement on DTI-MRI in Cushing's syndrome relates to mood disturbances and processing speed: a case-control study. Pituitary **20**(3), 340-348 (2017). doi:10.1007/s11102-017-0793-y
14. Resmini, E., Santos, A., Gómez-Anson, B., Vives, Y., Pires, P., Crespo, I., Portella, M.J., de Juan-Delago, M., Barahona, M.J., Webb, S.M.: Verbal and visual memory performance and hippocampal volumes, measured by 3-Tesla magnetic resonance imaging, in patients with Cushing's syndrome. J Clin Endocrinol Metab **97**(2), 663-671 (2012). doi:10.1210/jc.2011-2231
15. Santos, A., Resmini, E., Crespo, I., Pires, P., Vives-Gilabert, Y., Granell, E., Valassi, E., Gómez-Anson, B., Martínez-Momblán, M.A., Mataró, M., Webb, S.M.: Small cerebellar cortex volume in patients with active Cushing's syndrome. Eur J Endocrinol **171**(4), 461-469 (2014). doi:10.1530/eje-14-0371
16. Santos, A., Resmini, E., Gómez-Ansón, B., Crespo, I., Granell, E., Valassi, E., Pires, P., Vives-Gilabert, Y., Martínez-Momblán, M.A., de Juan, M., Mataró, M., Webb, S.M.: Cardiovascular risk and white matter lesions after endocrine control of Cushing's syndrome. Eur J Endocrinol **173**(6), 765-775 (2015). doi:10.1530/eje-15-0600
17. Santos, A., Granell, E., Gómez-Ansón, B., Crespo, I., Pires, P., Vives-Gilabert, Y., Valassi, E., Webb, S.M., Resmini, E.: Depression and Anxiety Scores Are Associated with Amygdala Volume in Cushing's Syndrome: Preliminary Study. Biomed Res Int **2017**, 2061935 (2017). doi:10.1155/2017/2061935
18. Simmons, N.E., Do, H.M., Lipper, M.H., Laws, E.R., Jr.: Cerebral atrophy in Cushing's disease. Surg Neurol **53**(1), 72-76 (2000). doi:10.1016/s0090-3019(99)00197-4
19. Starkman, M.N., Gebarski, S.S., Berent, S., Schteingart, D.E.: Hippocampal formation volume, memory dysfunction, and cortisol levels in patients with Cushing's syndrome. Biological psychiatry **32**(9), 756-765 (1992).
20. Starkman, M.N., Giordani, B., Gebarski, S.S., Berent, S., Schork, M.A., Schteingart, D.E.: Decrease in cortisol reverses human hippocampal atrophy following treatment of Cushing's disease. Biol Psychiatry **46**(12), 1595-1602 (1999). doi:10.1016/s0006-3223(99)00203-6
21. Starkman, M.N., Giordani, B., Gebarski, S.S., Schteingart, D.E.: Improvement in learning associated with increase in hippocampal formation volume. Biol Psychiatry **53**(3), 233-238 (2003). doi:10.1016/s0006-3223(02)01750-x
22. Tirosh, A., RaviPrakash, H., Papadakis, G.Z., Tatsi, C., Belyavskaya, E., Charalampos, L., Lodish, M.B., Bagci, U., Stratakis, C.A.: Computerized Analysis of Brain MRI Parameter Dynamics in Young Patients With Cushing Syndrome-A Case-Control Study. J Clin Endocrinol Metab **105**(5), e2069-2077 (2020). doi:10.1210/clinem/dgz303
23. Toffanin, T., Nifosì, F., Follador, H., Passamani, A., Zonta, F., Ferri, G., Scanarini, M., Amistà, P., Pigato, G., Scaroni, C., Mantero, F., Carollo, C., Perini, G.I.: Volumetric MRI analysis of hippocampal subregions in Cushing's disease: a model for glucocorticoid neural modulation. Eur Psychiatry **26**(1), 64-67 (2011). doi:10.1016/j.eurpsy.2010.09.003
24. van der Werff, S.J., Andela, C.D., Nienke Pannekoek, J., Meijer, O.C., van Buchem, M.A., Rombouts, S.A., van der Mast, R.C., Biermasz, N.R., Pereira, A.M., van der Wee, N.J.: Widespread reductions of white matter integrity in patients with long-term remission of Cushing's disease. Neuroimage Clin **4**, 659-667 (2014). doi:10.1016/j.nicl.2014.01.017
25. Bentson, J., Reza, M., Winter, J., Wilson, G.: Steroids and apparent cerebral atrophy on computed tomography scans. J Comput Assist Tomogr **2**(1), 16-23 (1978). doi:10.1097/00004728-197801000-00003

26. Brown, E.S., D, J.W., Frol, A., Bobadilla, L., Khan, D.A., Hanczyc, M., Rush, A.J., Fleckenstein, J., Babcock, E., Cullum, C.M.: Hippocampal volume, spectroscopy, cognition, and mood in patients receiving corticosteroid therapy. Biol Psychiatry **55**(5), 538-545 (2004). doi:10.1016/j.biopsych.2003.09.010
27. Brown, E.S., Jeon-Slaughter, H., Lu, H., Jamadar, R., Issac, S., Shad, M., Denniston, D., Tamminga, C., Nakamura, A., Thomas, B.P.: Hippocampal volume in healthy controls given 3-day stress doses of hydrocortisone. Neuropsychopharmacology **40**(5), 1216-1221 (2015). doi:10.1038/npp.2014.307
28. Brown, E.S., Kulikova, A., Van Enkevort, E., Nakamura, A., Ivleva, E.I., Tustison, N.J., Roberts, J., Yassa, M.A., Choi, C., Frol, A., Khan, D.A., Vazquez, M., Holmes, T., Malone, K.: A randomized trial of an NMDA receptor antagonist for reversing corticosteroid effects on the human hippocampus. Neuropsychopharmacology **44**(13), 2263-2267 (2019). doi:10.1038/s41386-019-0430-8
29. Brown, E.S., Woolston, D.J., Frol, A.B.: Amygdala volume in patients receiving chronic corticosteroid therapy. Biol Psychiatry **63**(7), 705-709 (2008). doi:10.1016/j.biopsych.2007.09.014
30. Desai, S., Khanani, S., Shad, M.U., Brown, E.S.: Attenuation of amygdala atrophy with lamotrigine in patients receiving corticosteroid therapy. J Clin Psychopharmacol **29**(3), 284-287 (2009). doi:10.1097/JCP.0b013e3181a3e2a7
31. Nguyen, D.M., Yassa, M.A., Tustison, N.J., Roberts, J.M., Kulikova, A., Nakamura, A., Ivleva, E.I., Van Enkevort, E., Brown, E.S.: The Relationship Between Cumulative Exogenous Corticosteroid Exposure and Volumes of Hippocampal Subfields and Surrounding Structures. J Clin Psychopharmacol **39**(6), 653-657 (2019). doi:10.1097/jcp.0000000000001120
