# Supplementary material for: Association between use of systemic and inhaled glucocorticoids and changes in brain volume and white matter microstructure: a cross-sectional study using data from the UK Biobank
Source: BMJ Open. 2022 Jul 28;12(8):e062446. doi: 10.1136/bmjopen-2022-062446 (PMC9438037; doi:10.1136/bmjopen-2022-062446)
Supplement: Supplementary data [file bmjopen-2022-062446supp004.pdf]

Supplements

Supplements 1, 2 and 3 are separate files.

Supplement 4. Violin plots of the imaging outcomes for the main analysis. AU, arbitrary units; GC, glucocorticoids

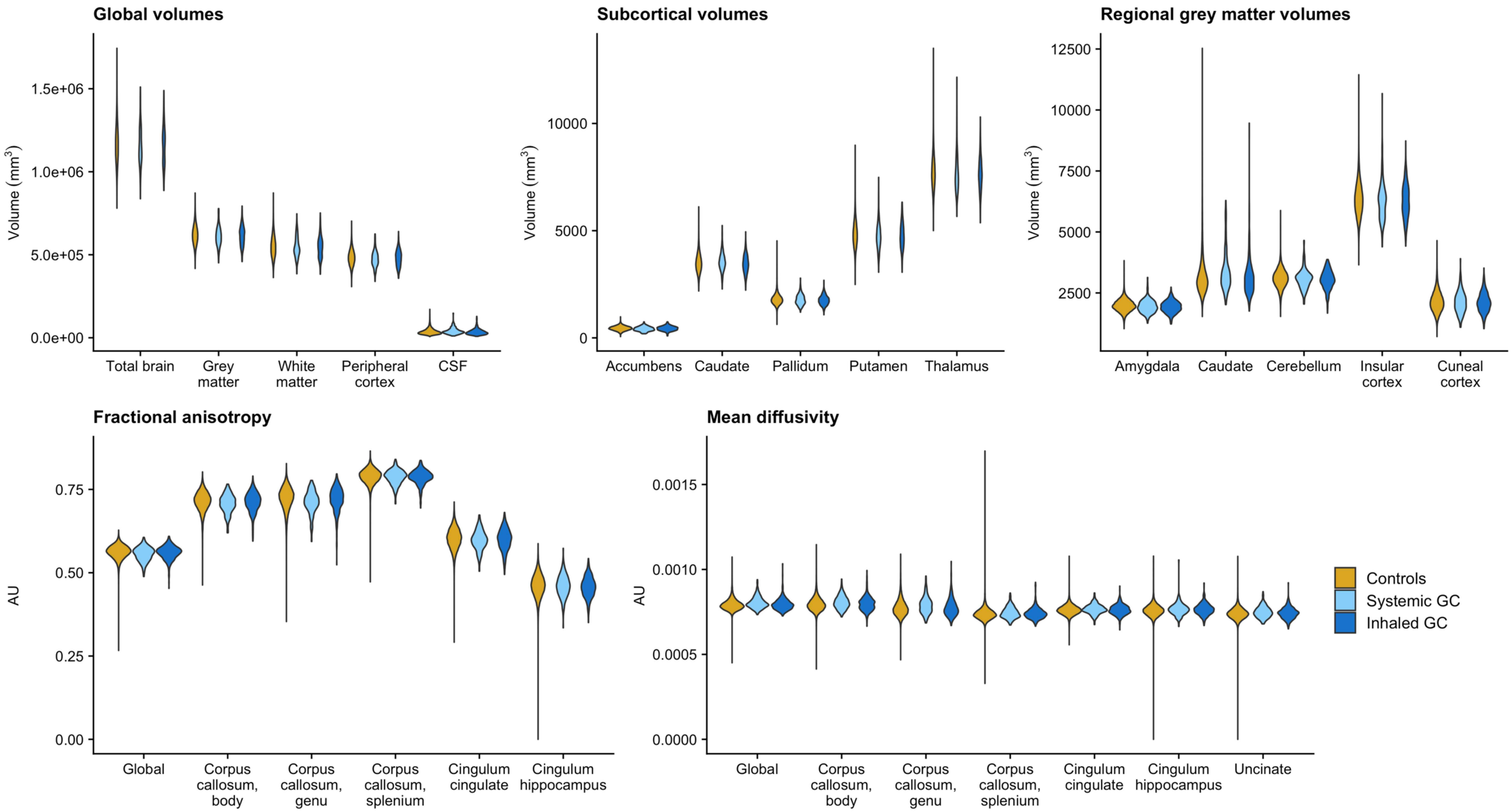

Supplement 5. Characteristics of included chronic glucocorticoid users and controls

	Patients using chronic systemic GC (n = 42)	Patients using chronic inhaled GC (n = 305)	Controls (n = 24106)	P value
**Sex: male, n (%)**	22 (52.4%)	137 (44.9%)	12154 (50.4%)	0.15
**Age at time of scanning in years, mean (SD)**	65.2 (7.0)	63.0 (7.6)	63.5 (7.5)	0.19
**Education level, n (%)**				0.81
College/University degree	24 (57.1)	171 (56.1)	12058 (50.0)	
A levels or equivalent	6 (14.3)	38 (12.5)	2930 (12.2)	
O levels/GCSE or equivalent	4 (9.5)	44 (14.4)	4155 (17.2)	
CSEs or equivalent	1 (2.4)	9 (3.0)	879 (3.6)	
NVQ, HND, HNC, or equivalent	1 (2.4)	14 (4.6)	1396 (5.8)	
Other professional qualifications	2 (4.8)	14 (4.6)	1150 (4.8)	
None of the above	1 (2.4)	14 (4.6)	1311 (5.4)	
Missing	3 (7.1)	1 (0.3)	227 (0.9)	
**BMI in kg/m^2, mean (SD)**	25.9 (3.7)	26.6 (4.4)	26.1 (4.1)	0.15
Number (%) missing	1 (2.4)	14 (4.6)	1325 (5.5)	
**Body fat percentage, mean (SD)**	30.0 (6.4)	32.0 (8.1)	30.2 (7.9)	4.6e-4
Number (%) missing	1 (2.4)	14 (4.6)	1331 (5.5)	
**Smoking status, n (%)**				0.42
Current	1 (2.4)	6 (2.0)	647 (2.7)	
Previous	8 (19.0)	112 (36.7)	7858 (32.6)	
Never	31 (73.8)	206 (67.5)	15380 (63.8)	
Missing	2 (4.8)	2 (0.7)	221 (0.9)	

BMI, body mass index; GC, glucocorticoids; n, number; SD, standard deviation.

P values were determined using analysis of variance (for continuous variables) and Fisher's exact test (for categorical variables, because of the low number of patients using chronic glucocorticoids).

Supplement 6. Imaging parameters, presented as the adjusted mean difference of patients using chronic systemic glucocorticoids (n = 42) or chronic inhaled glucocorticoids (n = 305) compared to controls (n = 24106)

	ANOVA			Systemic GC vs. controls			Inhaled GC vs. controls		
	**F value**	**P value**	**P_{FDR}**	**AMD***	**95% CI**	**P value**	**AMD***	**95% CI**	**P value**
Volumetric measures									
Global volumes (in mm^3)									
Total brain volume	17.0	4.1e-8	**1.5e-6**	-2535	-18869; 13798	0.90	3553	-2340; 9445	0.31
Grey matter volume	12.2	5.0e-6	**9.1e-5**	-1552	-10808; 7703	0.89	1636	-1703; 4975	0.45
White matter volume	10.8	2.0e-5	**1.8e-4**	-984	-11702; 9735	0.96	1917	-1950; 5784	0.44
Peripheral cortex	8.5	2.1e-4	**9.4e-4**	-2152	-10481; 6177	0.78	940	-2065; 3945	0.70
CSF volume	3.0	5.2e-2	**7.4e-2**	-2408	-7198; 2381	0.43	154	-1573; 1882	0.96
Subcortical volumes (in mm^3)									
Amygdala	5.8	2.9e-3	**8.2e-3**	52.1	-19.3; 123.5	0.19	-20.6	-46.4; 5.2	0.14
Caudate	7.2	7.6e-4	**2.9e-3**	112.7	-12.9; 238.2	0.09	-5.0	-50.3; 40.3	0.95
Hippocampus	4.9	7.8e-3	**1.7e-2**	59.2	-79.1; 197.5	0.54	-38.4	-88.3; 11.5	0.16
Pallidum	7.1	7.9e-4	**2.9e-3**	4.01	-68.2; 76.2	0.98	-23.0	-49.0; 3.1	0.094
Putamen	5.0	6.9e-3	**1.6e-2**	-65.4	-222.8; 92.0	0.55	-26.1	-82.9; 30.7	0.49
Thalamus	6.7	1.3e-3	**4.1e-3**	61.9	-120.7; 244.5	0.66	-11.6	-77.5; 54.3	0.88
Regional grey matter volumes (in mm^3)									
Amygdala	10.1	4.2e-5	**3.0e-4**	4.8	-60.8; 70.3	0.97	-15.1	-38.8; 8.5	0.27
Cerebellum	4.1	1.6e-2	**2.9e-2**	25.7	-76.5; 127.9	0.79	4.4	-32.4; 41.3	0.94
Cingulate gyrus, posterior	4.2	1.6e-2	**2.9e-2**	36.0	-158.8; 230.7	0.87	25.5	-44.8; 95.8	0.63

Hippocampus	9.1	1.1e-4	**6.6e-4**	63.5	-52.4; 179.5	0.37	-24.3	-66.1; 17.6	0.34
Precuneal cortex	8.6	1.8e-4	**9.1e-4**	170.0	-201.0; 541.2	0.49	-59.9	-194.0; 74.1	0.51
DTI measures									
Fractional anisotropy									
Global	5.4	4.4e-3	**1.1e-2**	-0.0066	-0.013; -3.2e-4	**0.038**	-0.0025	-0.0048; -2.3e-4	**0.027**
Genu of corpus callosum	5.8	3.2e-3	**8.2e-3**	-0.014	-0.025; -0.0031	**0.0087**	-0.0020	-0.0060; 0.0020	0.44
Cingulum hippocampus	3.7	2.4e-2	**3.9e-2**	0.0032	-0.0078; 0.014	0.73	-0.0034	-0.0074; 6.4e-4	0.11
Mean diffusivity									
Global	4.7	9.5e-3	**1.9e-2**	9.4e-6	8.7e-8; 1.9e-5	**0.05**	2.6e-6	-7.7e-7; 6.0e-6	0.16
Genu of corpus callosum	6.3	1.8e-3	**5.3e-3**	2.0e-5	5.5e-6; 3.5e-5	**0.0043**	2.8e-6	-2.5e-6; 8.0e-6	0.40
Splenium of corpus callosum	3.9	2.0e-2	**3.5e-2**	8.1e-6	-2.4e-6; 1.9e-5	0.16	5.2e-6	1.4e-6; 9.0e-6	**0.0044**
Cingulum hippocampus	11.6	9.0e-6	**1.1e-4**	8.2e-6	-2.4e-6; 1.9e-5	0.16	6.3e-6	2.5e-6; 1.0e-5	**5.0e-4**

* Adjusted mean difference, calculated using linear models, adjusted for age, sex, education, X-, Y-, and Z-position of the head in the scanner, head size, assessment centre, and year of imaging acquisition.

CI, confidence interval; P_{FDR}, Benjamini-Hochberg false discovery rate corrected P values; SE, standard error. P values in bold are statistically significant ($P < 0.05$).

Supplement 7. Cognitive outcome measures of chronic systemic glucocorticoid users (n = 42) and chronic inhaled glucocorticoid users (n = 305) vs. controls

	ANOVA			Systemic GC vs. controls			Inhaled GC vs. controls			Participants with available data, n (%)		
	**F value**	**P value**	**P_{FDR}**	**AMD***	**95% CI**	**P value**	**AMD***	**95% CI**	**P value**	**Systemic GC**	**Inhaled GC**	**Controls**
**Trail making A**	0.41	0.66	0.84	0.12	-0.26; 0.51	0.69	-0.07	-0.24; 0.10	0.55	30 (71)	151 (50)	16419 (68)
**Trail making B**	0.28	0.75	0.84	-0.08	-0.47; 0.31	0.84	0.00	-0.17; 0.17	1.00	28 (67)	148 (49)	16071 (67)
**Symbol substitution**	0.35	0.70	0.84	-0.08	-0.45; 0.30	0.84	-0.05	-0.21; 0.11	0.71	30 (71)	151 (50)	16442 (68)

* Adjusted mean difference between patients and controls, expressed in Z scores. Calculated using linear models, adjusting for age, sex, and education. Trail making A, and trail making B were log transformed before generation of Z scores because they were non-normally distributed. Variables were transformed such that higher values indicate a better performance.

CI, confidence interval; GC, glucocorticoids; n, number; P_{FDR}, Benjamini-Hochberg false discovery rate corrected P values

Supplement 8. Self-reported frequency of mental health parameters in the past two weeks of patients using systemic glucocorticoids (n = 222) or inhaled glucocorticoids (n = 557) and controls, presented as number of participants (%) per category

	**Systemic GC (n=222)**	**Inhaled GC (n=557)**	**Controls (n=24106)**
**Depressed mood**			
Not at all	170 (77)	455 (82)	19940 (83)
Several days	39 (18)	77 (14)	3017 (13)
More than half of the days	6 (2.7)	8 (1.4)	296 (1.2)
Nearly every day	1 (0.5)	3 (0.5)	150 (0.6)
Missing	6 (2.7)	14 (2.5)	703 (2.9)
**Disinterest**			
Not at all	174 (78)	468 (84)	20536 (85)
Several days	34 (15)	61 (11)	2568 (11)
More than half of the days	3 (1.3)	7 (1.3)	292 (1.2)
Nearly every day	5 (2.3)	5 (0.9)	174 (0.7)
Missing	6 (2.7)	16 (2.9)	536 (2.2)
**Tenseness/restlessness**			
Not at all	162 (73)	437 (78)	19412 (81)
Several days	46 (21)	89 (16)	3630 (15)
More than half of the days	3 (1.3)	12 (2.2)	272 (1.1)
Nearly every day	5 (2.3)	5 (0.9)	126 (0.5)
Missing	6 (2.7)	14 (2.5)	666 (2.8)
**Tiredness/lethargy**			
Not at all	95 (43)	280 (50)	13792 (57)
Several days	91 (41)	221 (40)	8345 (35)
More than half of the days	9 (4.1)	32 (5.7)	815 (3.4)
Nearly every day	19 (8.6)	15 (2.7)	555 (2.3)
Missing	8 (3.6)	9 (1.6)	599 (2.5)

GC, glucocorticoids; n, number.

Supplement 9. Self-reported frequency of mental health parameters in the past two weeks of chronic systemic glucocorticoid users (n = 42), chronic inhaled glucocorticoid users (n = 305) and controls, presented as number of participants (%) per category

	**Systemic GC (n = 42)**	**Inhaled GC (n = 305)**	**Controls (n = 24106)**
**Depressed mood**			
Not at all	33 (79)	257 (84)	19940 (83)
Several days	6 (14)	35 (11)	3017 (13)
More than half of the days	0 (0)	3 (0.9)	296 (1.2)
Nearly every day	0 (0)	1 (0.3)	150 (0.6)
Missing	3 (7.1)	9 (3.0)	703 (2.9)
**Disinterest**			
Not at all	34 (81)	267 (88)	20536 (85)
Several days	6 (14)	30 (9.8)	2568 (11)
More than half of the days	0 (0)	1 (0.3)	292 (1.2)
Nearly every day	0 (0)	0 (0)	174 (0.7)
Missing	2 (4.8)	7 (2.3)	536 (2.2)
**Tenseness/restlessness**			
Not at all	30 (71)	245 (80)	19412 (81)
Several days	10 (24)	48 (16)	3630 (15)
More than half of the days	0 (0)	6 (2.0)	272 (1.1)
Nearly every day	0 (0)	1 (0.3)	126 (0.5)
Missing	2 (4.8)	5 (1.6)	666 (2.8)
**Tiredness/lethargy**			
Not at all	24 (57)	156 (51)	13792 (57)
Several days	12 (29)	121 (40)	8345 (35)
More than half of the days	2 (4.8)	14 (4.6)	815 (3.4)
Nearly every day	2 (4.8)	8 (2.6)	555 (2.3)
Missing	2 (4.8)	6 (2.0)	599 (2.5)

GC, glucocorticoids; n, number.

Supplement 10. Likelihood of experiencing mental health complaints in the past two weeks of chronic systemic glucocorticoid users (n = 42) and chronic inhaled glucocorticoid users (n = 305) compared to controls

	Likelihood ratio test			Systemic GC vs. controls			Inhaled GC vs. controls		
	X^2	P value	P_{FDR}	OR	95% CI	P value	OR	95% CI	P value
**Depression**	1.1	0.57	0.57	1.21	0.45; 2.73	0.67	0.85	0.59; 1.18	0.34
**Disinterest**	2.2	0.33	0.44	1.41	0.53; 3.17	0.44	0.79	0.53; 1.13	0.21
**Tenseness**	2.5	0.28	0.44	1.84	0.84; 3.68	0.10	1.05	0.78; 1.40	0.73
**Tiredness**	4.4	0.11	0.44	0.96	0.49; 1.84	0.91	1.28	1.01; 1.61	**0.0037**

Calculated using logistic regression analysis, adjusting for age, sex, and education. P values in bold are statistically significant after Bonferroni correction for family-wise error rate of two tests ($P < 0.025$).

CI, confidence interval; GC, glucocorticoids; OR, odds ratio; P_{FDR}, Benjamini-Hochberg false discovery rate corrected P values.

Supplement 11. Sensitivity analysis: Characteristics of included glucocorticoid users and controls (without exclusion of participants based on psychiatric, neurological, or endocrine history or medication use)

	Patients using systemic GC (n = 312)	Patients using inhaled GC (n = 806)	Controls (n = 36310)	P value
**Sex: male, n (%)**	145 (46.5)	344 (42.7)	17041 (46.9)	0.057
**Age at time of scanning in years, mean (SD)**	66.1 (6.9)	62.8 (7.5)	63.7 (7.5)	3.6e-10
**Education level, n (%)**				0.37
College/University degree	143 (45.8)	407 (50.5)	17637 (48.6)	
A levels or equivalent	39 (12.5)	98 (12.2)	4392 (12.1)	
O levels/GCSE or equivalent	53 (17.0)	136 (16.9)	6400 (17.6)	
CSEs or equivalent	13 (4.2)	26 (3.2)	1372 (3.8)	
NVQ, HND, HNC, or equivalent	11 (3.5)	50 (6.2)	2142 (5.9)	
Other professional qualifications	21 (6.7)	45 (5.6)	1795 (4.9)	
None of the above	27 (8.7)	40 (5.0)	2208 (6.1)	
Missing	5 (1.6)	4 (0.5)	364 (1.0)	
**BMI in kg/m^2, mean (SD)**	26.7 (4.4)	27.1 (4.7)	26.5 (4.4)	2.2e-4
Number (%) missing	11 (3.5)	31 (3.8)	1932 (5.3)	
**Body fat percentage, mean (SD)**	31.9 (8.2)	32.6 (8.4)	31.1 (8.1)	5.5e-7
Number (%) missing	11 (3.5)	31 (3.8)	1942 (5.3)	
**Smoking status, n (%)**				0.096
Current	10 (3.2)	25 (3.1)	1231 (3.3)	
Previous	118 (37.8)	299 (37.1)	12063 (33.2)	
Never	181 (58.0)	477 (59.2)	22661 (62.4)	
Missing	3 (1.0)	5 (0.6)	355 (1.0)	

BMI, body mass index; GC, glucocorticoids; n, number; SD, standard deviation.

P values determined using analysis of variance (for continuous variables) and Pearson's Chi squared test (for categorical variables).

Supplement 12. Sensitivity analysis: Imaging parameters, presented as the adjusted mean difference of patients using systemic glucocorticoids (n = 312) or inhaled glucocorticoids (n = 806) compared to controls (n = 36310) (without exclusion of participants based on psychiatric, neurological, or endocrine history or medication use)

	ANOVA			Systemic GC vs. controls			Inhaled GC vs. controls		
	**F value**	**P value**	**P_{FDR}**	**AMD***	**95% CI**	**P value**	**AMD***	**95% CI**	**P value**
Volumetric measures									
Global volumes (in mm^3)									
Total brain volume	17.7	2.2e-8	**1.3e-7**	-3460	-9320; 2400	0.32	3535	-121; 7190	0.060
Grey matter volume	22.3	2.0e-10	**2.4e-9**	-2224	-5577; 1130	0.25	1454	-637; 3546	0.22
White matter volume	5.5	4.1e-3	**6.7e-3**	-1237	-5078; 2604	0.69	2080	-316; 4476	0.10
Peripheral cortex	24.6	2.0e-11	**4.4e-10**	-3318	-6330; -307	**0.028**	1172	-706; 3051	0.29
CSF volume	14.2	7.1e-7	**2.3e-6**	1220	-518; 2958	0.12	223	-861; 1307	0.65
Subcortical volumes (in mm^3)									
Accumbens	10.2	3.8e-5	**1.0e-4**	-8.9	-20.4; 2.7	0.16	-3.7	-10.9; 3.5	0.41
Caudate	4.5	1.1e-2	**1.7e-2**	58.6	13.8; 103.5	**0.0072**	-5.9	-33.9; 22.1	0.84
Pallidum	6.9	1.0e-3	**1.9e-3**	1.2	-24.5; 27.0	0.99	-16.2	-32.3; -0.2	**0.047**
Putamen	9.8	5.6e-5	**1.5e-4**	-33.8	-90.5; 22.9	0.32	-20.1	-55.5; 15.3	0.35
Thalamus	9.3	9.4e-5	**2.3e-4**	-19.9	-86.2; 46.5	0.72	-10.7	-52.1; 30.7	0.78
Regional grey matter volumes (in mm^3)									
Amygdala	21.0	7.8e-10	**7.0e-9**	-6.7	-30.4; 17.1	0.75	-21.7	-36.5; -6.8	**0.0023**
Caudate	12.3	4.7e-6	**1.4e-5**	149.6	66.9; 232.4	**1.0e-4**	42.9	-8.7; 94.5	0.12
Cerebellum	5.8	3.1e-3	**5.2e-3**	17.8	-19.4; 54.9	0.47	-2.9	-26.1; 20.3	0.93

Insular cortex	8.7	1.7e-4	**3.5e-4**	-42.1	-103.5; 19.4	0.23	8.0	-30.3; 46.3	0.84
Precuneal cortex	4.0	1.9e-2	**2.7e-2**	-9.7	-142.8; 123.4	0.97	-1.7	-84.7; 81.3	1.00
DTI measures									
Fractional anisotropy									
Global	15.5	1.8e-7	**9.4e-7**	-0.0031	-0.0055; -7.5e-4	**0.0066**	-0.0015	-0.0030; -4.9e-5	**0.041**
Body of corpus callosum	8.9	1.4e-4	**3.1e-4**	-0.0039	-0.0076; -0.0003	**0.032**	-0.0014	-0.0036; 8.9e-4	0.30
Genu of corpus callosum	15.2	2.5e-7	**1.1e-6**	-0.0056	-0.0097; -0.0014	**0.0055**	-0.0013	-0.0039; 0.0013	0.44
Cingulum cingulate	3.8	2.3e-2	**3.1e-2**	-0.0014	-0.0052; 0.0025	0.64	-0.0018	-0.0042; 5.9e-4	0.17
Mean diffusivity									
Global	24.5	2.4e-11	**4.4e-10**	6.6e-6	3.0e-6; 1.0e-5	**3.7e-5**	1.9e-6	-3.2e-7; 4.1e-6	5.7e-2
Body of corpus callosum	14.2	6.7e-7	**2.3e-6**	6.7e-6	1.9e-6; 1.1e-5	**0.0034**	4.0e-6	1.1e-6; 7.0e-6	**0.0048**
Genu of corpus callosum	17.9	1.7e-8	**1.2e-7**	8.0e-6	2.5e-6; 1.4e-5	**0.0023**	3.3e-6	-1.4e-7; 6.7e-6	0.0622
Splenium of corpus callosum	6.7	1.2e-3	**2.2e-3**	3.7e-6	-3.1e-7; 7.6e-6	0.076	4.0e-6	1.5e-6; 6.4e-6	**7.0e-4**
Cingulum cingulate	4.9	7.6e-3	**1.2e-2**	2.5e-6	-6.8e-7; 5.7e-6	0.15	2.2e-6	2.2e-7; 4.2e-6	**0.026**
Cingulum hippocampus	14.5	4.9e-7	**2.0e-6**	2.6e-6	-1.3e-6; 6.6e-6	0.25	4.5e-6	2.0e-6; 7.0e-6	**1.0e-4**
Uncinate fasciculus	7.3	6.6e-4	**1.3e-3**	4.0e-6	2.9e-7; 7.7e-6	**0.032**	1.6e-6	-7.5e-7; 3.9e-6	0.23

* Adjusted mean difference, calculated using linear models, adjusted for age, sex, education, X-, Y-, and Z-position of the head in the scanner, head size, assessment centre, and year of imaging acquisition.

P_{FDR}, Benjamini-Hochberg false discovery rate corrected P values; SE, standard error. P values in bold are statistically significant (P < 0.05).

Supplement 13. Sensitivity analysis: Cognitive outcome measures of systemic glucocorticoid users (n = 312) and inhaled glucocorticoid users (n = 806) vs. controls (n = 36310) (without exclusion of participants based on psychiatric, neurological, or endocrine history or medication use)

	ANOVA			Systemic GC vs. controls			Inhaled GC vs. controls			Participants with available data, n (%)		
	**F value**	**P value**	**P_{FDR}**	**AMD***	**95% CI**	**P value**	**AMD***	**95% CI**	**P value**	**Systemic GC**	**Inhaled GC**	**Controls**
**Trail making A**	6.6	0.0014	**0.0028**	-0.11	-0.26; 0.03	0.16	0.020	-0.08; 0.12	0.86	206 (66)	422 (52)	24297 (67)
**Trail making B**	6.7	0.0013	**0.0028**	-0.12	-0.27; 0.02	0.10	-0.018	-0.12; 0.08	0.88	194 (62)	415 (51)	23273 (64)
**Symbol substitution**	9.7	6.2e-5	**0.00037**	-0.15	-0.29; -0.01	**0.029**	-0.061	-0.16; 0.04	0.28	203 (65)	423 (52)	24337 (67)

* Adjusted mean difference between patients and controls, expressed in Z scores. Calculated using linear models, adjusted for age, sex, and education. Trail making A, and trail making B were log transformed before generation of Z scores because they were non-normally distributed. Variables were transformed such that higher values indicate a better performance.

GC, glucocorticoids; P_{FDR}, Benjamini-Hochberg false discovery rate corrected P values.

Supplement 14. Sensitivity analysis: Self-reported frequency of mental health parameters in the past two weeks of patients using systemic glucocorticoids (n = 312) or inhaled glucocorticoids (n = 806) and controls, presented as number of participants (%) per category (without exclusion of participants based on psychiatric, neurological, or endocrine history or medication use)

	**Systemic GC (n = 312)**	**Inhaled GC (n = 806)**	**Controls (n = 36310)**
**Depressed mood**			
Not at all	240 (76.9)	620 (76.9)	29014 (80.0)
Several days	55 (17.6)	139 (17.2)	5197 (14.3)
More than half of the days	8 (2.6)	14 (1.7)	593 (1.6)
Nearly every day	2 (0.6)	14 (1.7)	360 (1.0)
Missing	7 (2.2)	19 (2.4)	1146 (3.2)
**Disinterest**			
Not at all	237 (76.0)	639 (79.3)	29916 (82.4)
Several days	55 (17.6)	118 (14.6)	4583 (12.6)
More than half of the days	8 (2.6)	17 (2.1)	604 (1.7)
Nearly every day	5 (1.6)	12 (1.5)	357 (1.0)
Missing	7 (2.2)	20 (2.5)	850 (2.3)
**Tenseness/restlessness**			
Not at all	221 (70.8)	588 (73.0)	28266 (77.8)
Several days	71 (22.8)	157 (19.5)	6113 (16.8)
More than half of the days	6 (1.9)	23 (2.9)	565 (1.6)
Nearly every day	6 (1.9)	16 (2.0)	313 (0.9)
Missing	8 (2.6)	22 (2.7)	1053 (2.9)
**Tiredness/lethargy**			
Not at all	125 (40.0)	366 (45.4)	19107 (52.6)
Several days	130 (41.7)	321 (39.8)	13373 (36.8)
More than half of the days	22 (7.1)	53 (6.6)	1533 (4.2)
Nearly every day	26 (8.3)	51 (6.3)	1358 (3.7)
Missing	9 (2.9)	15 (1.9)	939 (2.6)

GC, glucocorticoids; n, number.

Supplement 15. Sensitivity analysis: Likelihood of experiencing mental health complaints in the past two weeks of systemic glucocorticoid users (n = 312) and inhaled glucocorticoid users (n = 806) compared to controls (without exclusion of participants based on psychiatric, neurological, or endocrine history or medication use)

	**Likelihood ratio test**			**Systemic GC vs. controls**			**Inhaled GC vs. controls**		
	**X^2**	**P value**	**P_{FDR}**	**OR**	**95% CI**	**P value**	**OR**	**95% CI**	**P value**
**Depression**	11.1	0.0039	0.0039	1.44	1.08; 1.89	**0.010**	1.23	1.03; 1.46	**0.023**
**Disinterest**	17.8	1.4e-4	1.9e-04	1.73	1.31; 2.27	**8.5e-05**	1.21	1.00; 1.45	0.041
**Tenseness**	24.0	6.1e-06	1.2e-05	1.68	1.29; 2.16	**7.0e-05**	1.31	1.11; 1.54	**0.0014**
**Tiredness**	39.2	3.1e-09	1.2e-08	1.79	1.42; 2.27	**9.0e-07**	1.33	1.15; 1.53	**1.1e-4**

Calculated using logistic regression analysis, adjusting for age, sex, and education. P values in bold are statistically significant after Bonferroni correction for family-wise error rate of two tests (P < 0.025).

CI, confidence interval; GC, glucocorticoids; OR, odds ratio; P_{FDR}, Benjamini-Hochberg false discovery rate corrected P values.

Supplement 16. Sensitivity analysis: Imaging parameters, presented as the adjusted mean difference of patients using systemic glucocorticoids (n = 222) or inhaled glucocorticoids (n = 557) compared to controls (n = 24106) (after exclusion of outlier values per group per variable)

	ANOVA			Systemic GC vs. controls			Inhaled GC vs. controls		
	**F value**	**P value**	**P_{FDR}**	**AMD***	**95% CI**	**P value**	**AMD***	**95% CI**	**P value**
Volumetric measures									
Global volumes (in mm³)									
Total brain volume	16.0	1.1e-7	**4.6e-7**	-3991	-10852; 2869	0.33	3756	-565; 8076	0.10
Grey matter volume	28.8	3.4e-13	**6.1e-12**	-3143	-7081; 794	0.14	1120	-1337; 3576	0.50
White matter volume	5.4	4.6e-3	**7.1e-3**	-1861	-6349; 2626	0.55	2374	-454; 5203	0.11
Peripheral cortex	27.0	2.0e-12	**1.8e-11**	-4412	-7948; -876	**0.011**	1148	-1058; 3355	0.41
CSF volume	16.8	5.0e-8	**2.3e-7**	1437	-210; 3084	0.10	-449	-1492; 594	0.53
Subcortical volumes (in mm³)									
Accumbens	13.0	2.3e-6	**5.8e-6**	-15.6	-28.8; -2.3	**0.018**	-4.6	-13.0; 3.7	0.37
Caudate	4.7	8.8e-3	**1.1e-2**	69.4	18.4; 120.3	**0.0049**	4.5	-27.4; 36.3	0.92
Hippocampus	5.4	4.7e-3	**7.1e-3**	-17.1	-71.2; 37.0	0.70	-17	-51.3; 17.3	0.44
Pallidum	4.9	7.4e-3	**9.8e-3**	5.7	-20.5; 31.8	0.83	-9.8	-26.3; 6.7	0.32
Putamen	13.7	1.1e-6	**3.4e-6**	-63	-127.1; 1.0	0.055	-19.9	-59.7; 20.0	0.44
Thalamus	10.0	4.6e-5	**8.7e-5**	-25.6	-98.2; 46.9	0.64	-0.6	-46.2; 45.1	1.00
Regional grey matter volumes (in mm³)									
Amygdala	28.3	5.1e-13	**6.1e-12**	-17.2	-43.8; 9.4	0.26	-22.6	-39.3; -5.9	**0.01**
Caudate	12.6	3.5e-6	**8.4e-6**	138.1	67.7; 208.6	**<0.0001**	15.1	-28.8; 59.1	0.66
Cerebellum	10.3	3.3e-5	**6.6e-5**	-1.1	-42.8; 40.6	1.00	-6.6	-32.5; 19.3	0.78

Cingulate gyrus, anterior	3.9	2.1e-2	**2.6e-2**	110.5	-7.8; 229.0	0.071	27.1	-47.9; 102.0	0.63
Hippocampus	3.3	3.9e-2	**4.6e-2**	24.3	-22.4; 70.9	0.41	2.4	-27.0; 31.8	0.97
Insular cortex	13.1	2.0e-6	**5.5e-6**	-74.8	-143.2; -6.4	**0.029**	8.7	-34.1; 51.4	0.85
Precuneal cortex	5.2	5.4e-3	**7.5e-3**	-60.1	-213.6; 93.3	0.59	0.0	-95.6; 95.6	1.00
DTI measures									
Fractional anisotropy									
Global	22.7	1.4e-10	**1.0e-9**	-0.0043	-0.0067; -0.0018	**2.0e-4**	-0.0019	-0.0035; -3.4e-4	**0.013**
Body of corpus callosum	11.4	1.1e-5	**2.5e-5**	-0.0048	-0.0086; -0.0010	**0.0097**	-0.0021	-0.0045; 3.4e-4	0.11
Genu of corpus callosum	15.3	2.3e-7	**8.4e-7**	-0.0059	-0.010; -0.0016	**0.0048**	-0.0017	-0.0044; 0.0010	0.28
Cingulum cingulate	6.5	1.5e-3	**2.5e-3**	-0.0022	-0.0065; 0.0021	0.42	-0.0026	-0.0053; 9.7e-5	0.061
Cingulum hippocampus	7.5	5.7e-4	**9.7e-4**	-0.00012	-0.0046; 0.0044	1.00	-0.0036	-0.0064; -7.5e-4	**0.010**
Mean diffusivity									
Global	29.1	2.4e-13	**6.1e-12**	7.1e-6	3.7e-6; 1.1e-5	**<0.0001**	2.5e-6	3.1e-7; 4.7e-6	**0.022**
Body of corpus callosum	17.1	3.6e-8	**1.9e-7**	7.5e-6	2.8e-6; 1.2e-5	**7.0e-4**	3.7e-6	6.9e-7; 6.6e-6	**0.012**
Genu of corpus callosum	21.6	4.3e-10	**2.6e-9**	9.5e-6	3.9e-6; 1.5e-5	**3.0e-4**	3.6e-6	2.9e-8; 7.1e-6	**0.048**
Splenium of corpus callosum	9.9	5.2e-5	**9.4e-5**	4.6e-6	7.3e-7; 8.4e-6	**0.016**	4.2e-6	1.8e-6; 6.7e-6	**2.0e-4**
Cingulum cingulate	5.3	5.2e-3	**7.5e-3**	2.6e-6	-9.4e-7; 6.1e-6	0.19	2.6e-6	3.6e-7; 4.8e-6	**0.019**
Cingulum hippocampus	13.7	1.1e-6	**3.4e-6**	4.4e-6	2.5e-7; 8.6e-6	**0.035**	4.3e-6	1.6e-6; 6.9e-6	**6.0e-4**
Uncinate fasciculus	11.3	1.2e-5	**2.5e-5**	5.8e-6	1.9e-6; 9.7e-6	**0.0018**	2.4e-6	-8.8e-8; 4.8e-6	0.061

* Adjusted mean difference, calculated using linear models, adjusted for age, sex, education, X-, Y-, and Z-position of the head in the scanner, head size, assessment centre, and year of imaging acquisition.

P_{FDR}, Benjamini-Hochberg false discovery rate corrected P values; SE, standard error. P values in bold are statistically significant ($P < 0.05$).

Supplement 17. Cognitive outcome measures of systemic glucocorticoid users (n = 222) and inhaled glucocorticoid users (n = 557) vs. controls (after exclusion of outlier values per group per variable)

	**ANOVA**			**Systemic GC vs. controls**			**Inhaled GC vs. controls**			**Participants with available data, n (%)**		
	**F value**	**P value**	**P_{FDR}**	**AMD***	**95% CI**	**P value**	**AMD***	**95% CI**	**P value**	**Systemic GC**	**Inhaled GC**	**Controls**
**Trail making A**	5.2	0.0057	**0.011**	-0.10	-0.25; 0.05	0.25	-0.018	-0.12; 0.09	0.88	143 (64)	286 (51)	15996 (66)
**Trail making B**	9.6	6.8e-5	**2.0e-4**	-0.16	-0.32; -0.01	**0.038**	-0.064	-0.17; 0.04	0.31	137 (62)	289 (52)	15733 (65)
**Symbol substitution**	11.6	8.9e-6	**5.3e-5**	-0.18	-0.34; -0.02	**0.021**	-0.046	-0.16; 0.06	0.55	141 (64)	295 (53)	16270 (67)

* Adjusted mean difference between patients and controls, expressed in Z scores. Calculated using linear models, adjusted for age, sex, and education. Trail making A, and trail making B were log transformed before generation of Z scores because they were non-normally distributed. Variables were transformed such that higher values indicate a better performance.

GC, glucocorticoids; P_{FDR}, Benjamini-Hochberg false discovery rate corrected P values.

Supplement 18. STROBE Statement – Checklist of items that should be included in reports of cohort studies

	Item No	Recommendation	Where to be found
**Title and abstract**	1	(*a*) Indicate the study's design with a commonly used term in the title or the abstract	Abstract: Design (p.2)
		(*b*) Provide in the abstract an informative and balanced summary of what was done and what was found	Abstract: Main outcome measures, Results (p.2)
**Introduction**			
Background/ rationale	2	Explain the scientific background and rationale for the investigation being reported	Introduction (p.4)
Objectives	3	State specific objectives, including any prespecified hypotheses	Introduction (p.4)
**Methods**			
Study design	4	Present key elements of study design early in the paper	Study design (p.5)
Setting	5	Describe the setting, locations, and relevant dates, including periods of recruitment, exposure, follow-up, and data collection	Study design, Data collection (p.5)
Participants	6	(*a*) Give the eligibility criteria, and the sources and methods of selection of participants. Describe methods of follow-up	Participants (pp.5-6)
		(*b*) For matched studies, give matching criteria and number of exposed and unexposed	*Not applicable*
Variables	7	Clearly define all outcomes, exposures, predictors, potential confounders, and effect modifiers. Give diagnostic criteria, if applicable	Data collection, Imaging data, Cognitive and Emotional data, Statistical analysis (pp.5-9)
Data sources/ measurement	8	For each variable of interest, give sources of data and details of methods of assessment (measurement). Describe comparability of assessment methods if there is more than one group	Data collection, Imaging data, Cognitive and Emotional data (pp.5-7)
Bias	9	Describe any efforts to address potential sources of bias	Statistical analysis (pp.7-9)
Study size	10	Explain how the study size was arrived at	Participants (pp.5-6)
Quantitative variables	11	Explain how quantitative variables were handled in the analyses. If applicable, describe which groupings were chosen and why	Statistical analysis (pp.7-9)
Statistical methods	12	(*a*) Describe all statistical methods, including those used to control for confounding	Statistical analysis (pp.7-9)
		(*b*) Describe any methods used to examine subgroups and interactions	
		(*c*) Explain how missing data were addressed	
		(*d*) If applicable, explain how loss to follow-up was addressed	
		(*e*) Describe any sensitivity analyses	

**Results**			
Participants	13	(a) Report numbers of individuals at each stage of study – e.g. numbers potentially eligible, examined for eligibility, confirmed eligible, included in the study, completing follow-up, and analyzed	Demographic characteristics (p.10) and Figure 1
		(b) Give reasons for non-participation at each stage	
		(c) Consider use of a flow diagram	
Descriptive data	14	(a) Give characteristics of study participants (e.g. demographic, clinical, social) and information on exposures and potential confounders	Demographic characteristics (p.10) and Table 1
		(b) Indicate number of participants with missing data for each variable of interest	
		(c) Summarize follow-up time (e.g., average and total amount)	*Not applicable*
Outcome data	15*	Report numbers of outcome events or summary measures over time	Results (pp.12-19)
Main results	16	(*a*) Give unadjusted estimates and, if applicable, confounder-adjusted estimates and their precision (e.g., 95% confidence interval). Make clear which confounders were adjusted for and why they were included	Results (pp.12-19), Tables 2-4, Supplements
		(*b*) Report category boundaries when continuous variables were categorized	Statistical analysis (p.8)
		(*c*) If relevant, consider translating estimates of relative risk into absolute risk for a meaningful time period	*Not applicable*
Other analyses	17	Report other analyses done – e.g. analyses of subgroups and interactions, and sensitivity analyses	Results (p.20), Supplements
**Discussion**			
Key results	18	Summarize key results with reference to study objectives	Discussion (pp.21-22)
Limitations	19	Discuss limitations of the study, taking into account sources of potential bias or imprecision. Discuss both direction and magnitude of any potential bias	Strengths and limitations (pp.23-25)
Interpretation	20	Give a cautious overall interpretation of results considering objectives, limitations, multiplicity of analyses, results from similar studies, and other relevant evidence	Findings in context, Potential consequences and implications (pp.21-23)
Generalizability	21	Discuss the generalizability (external validity) of the study results	Strengths and limitations (pp.23-25)
**Other information**			
Funding	22	Give the source of funding and the role of the funders for the present study and, if applicable, for the original study on which the present article is based	Funding (p.26)
