# Supplementary material for: Association between use of systemic and inhaled glucocorticoids and changes in brain volume and white matter microstructure: a cross-sectional study using data from the UK Biobank
Source: BMJ Open. 2022 Jul 28;12(8):e062446. doi: 10.1136/bmjopen-2022-062446 (PMC9438037; doi:10.1136/bmjopen-2022-062446)
Supplement: Author's manuscript [file bmjopen-2022-062446.draft_revisions.pdf]

BMJ Open is committed to open peer review. As part of this commitment we make the peer review history of every article we publish publicly available.

When an article is published we post the peer reviewers' comments and the authors' responses online. We also post the versions of the paper that were used during peer review. These are the versions that the peer review comments apply to.

The versions of the paper that follow are the versions that were submitted during the peer review process. They are not the versions of record or the final published versions. They should not be cited or distributed as the published version of this manuscript.

BMJ Open is an open access journal and the full, final, typeset and author-corrected version of record of the manuscript is available on our site with no access controls, subscription charges or pay-per-view fees (http://bmjopen.bmj.com).

If you have any questions on BMJ Open's open peer review process please email info.bmjopen@bmj.com

Oral and inhalation glucocorticoid use associate with changes in brain volume and white matter microstructure: a cross-sectional UK Biobank study

Journal:	*BMJ Open*
Manuscript ID	bmjopen-2022-062446
Article Type:	Original research
Date Submitted by the Author:	02-Mar-2022
Complete List of Authors:	van der Meulen, Merel; Leiden University Medical Center, Department of Medicine, division of Endocrinology<br>Amaya, Jorge Miguel; Leiden University Medical Center, Department of Medicine, division of Endocrinology<br>Dekkers, Olaf; Leiden University Medical Center, Department of Medicine, division of Endocrinology<br>Meijer, Onno C.; Leiden University Medical Center, Department of Medicine, division of Endocrinology
Keywords:	Magnetic resonance imaging < RADIOLOGY & IMAGING, Anxiety disorders < PSYCHIATRY, DIABETES & ENDOCRINOLOGY, INTERNAL MEDICINE, MENTAL HEALTH, Depression & mood disorders < PSYCHIATRY

SCHOLARONE™
Manuscripts

I, the Submitting Author has the right to grant and does grant on behalf of all authors of the Work (as defined in the below author licence), an exclusive licence and/or a non-exclusive licence for contributions from authors who are: i) UK Crown employees; ii) where BMJ has agreed a CC-BY licence shall apply, and/or iii) in accordance with the terms applicable for US Federal Government officers or employees acting as part of their official duties; on a worldwide, perpetual, irrevocable, royalty-free basis to BMJ Publishing Group Ltd ("BMJ") its licensees and where the relevant Journal is co-owned by BMJ to the co-owners of the Journal, to publish the Work in this journal and any other BMJ products and to exploit all rights, as set out in our licence.

The Submitting Author accepts and understands that any supply made under these terms is made by BMJ to the Submitting Author unless you are acting as an employee on behalf of your employer or a postgraduate student of an affiliated institution which is paying any applicable article publishing charge ("APC") for Open Access articles. Where the Submitting Author wishes to make the Work available on an Open Access basis (and intends to pay the relevant APC), the terms of reuse of such Open Access shall be governed by a Creative Commons licence – details of these licences and which Creative Commons licence will apply to this Work are set out in our licence referred to above.

Other than as permitted in any relevant BMJ Author's Self Archiving Policies, I confirm this Work has not been accepted for publication elsewhere, is not being considered for publication elsewhere and does not duplicate material already published. I confirm all authors consent to publication of this Work and authorise the granting of this licence.

For peer review only

Oral and inhalation glucocorticoid use associate with changes in brain volume and white matter microstructure: a cross-sectional UK Biobank study

Authors: Merel van der Meulen (0000-0002-0001-4408)[1], Jorge Miguel Amaya[1], Olaf M. Dekkers[1], Onno C. Meijer (0000-0002-8394-6859)[1]

[1] Department of Medicine, division of Endocrinology, Leiden University Medical Center, Leiden, the Netherlands

Merel van der Meulen: PhD candidate
Jorge Miguel Amaya: PhD candidate
Olaf M. Dekkers: full professor
Onno C. Meijer: full professor

Correspondence to: Merel van der Meulen, m.vandermeulen@lumc.nl, Department of Medicine, division of Endocrinology, Leiden University Medical Center, Leiden, the Netherlands

Tables: 5
Figures: 2
Word count: 5214

For peer review only

Abstract (298 words)

Objective: To test the hypothesis that oral and inhalation glucocorticoid use are associated with changes in grey matter volume (GMV) and white matter microstructure.

Design: Cross-sectional population-based cohort study.

Setting: UK Biobank.

Participants: After exclusion based on neurological, psychiatric, or endocrinological history, and use of psychotropic medication, 222 oral glucocorticoid users, 557 inhalation glucocorticoid users, and 24,106 controls with available T1 and diffusion MRI data were included.

Main outcome measures: Primary outcomes were differences in 22 volumetric and 14 diffusion imaging parameters between glucocorticoid users and controls, determined using linear regression analyses adjusted for potential confounders. Secondary outcomes included cognitive functioning (six tests) and emotional symptoms (four questions)

Results: Both oral and inhalation glucocorticoid use were associated with reduced white matter integrity (lower fractional anisotropy (FA) and higher mean diffusivity (MD)) compared to controls, with larger effect sizes in oral users (FA: adjusted mean difference (AMD) = -3.7e-3, -95% confidence interval (CI) = -6.4e-3 to 1.0e-3; MD: AMD=7.2e-6, 95%CI=3.2e-6 to 1.1e-5) than inhalation users (FA: AMD=-2.3e-3, 95%CI=-4.0e-3 to -5.7e-4; MD: AMD=2.7e-6, 95%CI=1.7e-7 to 5.2e-6). Oral use was also associated with larger GMV of the caudate nucleus (AMD=178.7 mm^3, 95%CI=82.2 to 275.0), while inhalation users had smaller amygdala GMV (AMD=-23.9 mm^3, 95%CI=-41.5 to -6.2) than controls. As for secondary outcomes, oral users performed worse on the symbol digit substitution task (AMD=-0.17 SD, 95%CI=-0.34 to -0.01), and reported more depressive symptoms (OR=1.76, 95%CI=1.25 to 2.43), disinterest (OR=1.84, 95%CI=1.29 to 2.56), tenseness/restlessness (OR=1.78, 95%CI=1.29 to 2.41), and tiredness/lethargy (OR=1.90, 95%CI=1.45 to 2.50) compared to controls. Inhalation users only reported more tiredness/lethargy than controls (OR=1.35, 95%CI=1.14 to 1.60).

Conclusions: Both oral and inhalation glucocorticoid use are associated with decreased white matter integrity and limited changes in GMV. This may contribute to the neuropsychiatric side effects of glucocorticoid medication, especially with chronic use.

For peer review only

Strengths and limitations of this study

Strengths:

- To the best of our knowledge, this is the largest study to date assessing the effects of glucocorticoid use on the brain, and the first to investigate these effects in inhalation glucocorticoid users.
- Relatively strict exclusion criteria were used to limit the potential confounding that may arise in observational cohort studies.

Limitations:

- The cross-sectional nature of this study precludes formal conclusions on causality.
- Dose and duration of medication use were not available in the UK Biobank, making thorough analyses on dose- or duration-dependant effects impossible.

For peer review only

Introduction

Due to their immunosuppressive properties, glucocorticoids are among the most prescribed drugs on the market, with an estimated annual prevalence of systemic glucocorticoid use between 0.5% and 3% [1-5]. Although efficacious, both systemic and local (especially inhaled) glucocorticoids are associated with many potentially serious metabolic, cardiovascular, and musculoskeletal side effects [6-9]. Besides these physical side effects, the use of synthetic glucocorticoids is also associated with neuropsychiatric symptoms and disorders, including depression, mania, delirium, and even a seven-fold increased suicide (attempt) rate [10, 11]. In addition, on an anatomical level, both preclinical and clinical studies have shown long-lasting effects of glucocorticoid overexposure on the brain. In patients with chronic endogenous glucocorticoid excess due to a pituitary tumour (Cushing disease), it has been established that long-term glucocorticoid excess is associated with global cerebral atrophy [12-18] and decreased cortical thickness and grey matter volumes in specific brain regions [13, 18-26]. Some of these effects were detected even after ten years of biochemical remission [22, 23]. Moreover, a few small studies have shown volumetric reductions in specific brain regions in patients using chronic and/or high-dose synthetic oral glucocorticoids [27-31]. Besides these structural abnormalities, several studies in animal models and patients with Cushing disease have also demonstrated widespread reductions in white matter integrity throughout the brain [32-36].

However, most clinical studies investigating the effects of glucocorticoid overexposure on brain structure have been performed in small, selected populations with chronic glucocorticoid excess due to Cushing disease or oral glucocorticoid use. It remains unknown whether these consequences can also be observed in a broader sample of people using glucocorticoid, including inhalation glucocorticoids. We therefore used data from the UK Biobank, a large population-based cohort study, to investigate whether, at a population level, differences in brain volumes and white matter microstructure could be detected between oral or inhalation glucocorticoid users and non-users. As secondary outcomes, we also assessed potential differences in cognitive and emotional functioning.

For peer review only

Methods

Study design

The UK Biobank is a large population-based prospective cohort, comprising over 500,000 participants aged 40-69 years at the time of recruitment (between 2006 and 2010)[37]. The protocol for the UK Biobank was approved by the North-West Multi-Centre Research Ethics Committee and all participants provided written informed consent for collection, storage, and use of their data. Data for the present study were obtained under application number 59004.

Data collection

Data were collected at the assessment centres and during an online follow-up. Data used for this study included data on demographic characteristics, health and medical history, brain imaging, cognitive and emotional functioning, and body composition. Data on demographic characteristics, cognition, and emotional functioning were collected using a touch screen device at the assessment centres. If patients had indicated that they did not want to answer a question on one or more of these characteristics, we coded this as missing. Data on health and medical history, including medication use, were collected using the touch screen device and a verbal interview (self-reported data), but also using hospital episode statistics (HES). Body composition was measured using body impedance on a Tanita BC418MA body composition analyser as described in the UK Biobank documentation [38]. The imaging acquisition is described in more detail below.

Participants

For the analysis presented in this study, we selected participants who

1. Had both T1-weighted magnetic resonance imaging (MRI) and diffusion tensor imaging (DTI) data available at the same imaging visit;
2. Did not have a history of psychiatric disease based on self-reported data or HES data, other than anxiety, depression, mania, and delirium, because anxiety, depression, mania, and delirium may be related to glucocorticoid use [10];
3. Did not use psychotropic medication;
4. And did not have any neurological condition based on self-reported or HES data.

Individuals who met these criteria and used oral glucocorticoids at the time of imaging were included in the oral glucocorticoid patient group (n = 222), and individuals who met these criteria and used inhalation glucocorticoids (but no oral glucocorticoids) at the time of imaging were included in the inhalation glucocorticoid group (n = 557). Individuals who met these criteria but had not used oral or inhalation glucocorticoids at any timepoint (before and including the imaging visit) and did not have any endocrinological disorder according to self-reported or HES data, were included in the control

For peer review only - http://bmjopen.bmj.com/site/about/guidelines.xhtml

group (n = 24,106). A flowchart of patient selection is presented in Figure 1, and Supplement 1 provides a list of all Biobank UK field codes that were used as inclusion or exclusion criteria.

Imaging data

Our study made use of imaging-derived phenotypes (IDPs) generated by an image-processing pipeline developed and run on behalf of the UK Biobank. Details on the brain imaging acquisition protocols, imaging processing and quality control, and generation of IDPs are provided by the UK Biobank[39, 40]. In short, all imaging was performed on a standard Siemens Skyra 3 Tesla scanner with a standard Siemens 32-channel radiofrequency receiver head coil. T1-weighted imaging was performed using a three-dimensional magnetization-prepared rapid acquisition with gradient echo sequence (3D MPRAGE) in the sagittal plane (voxel 1x1x1 mm; field-of-view 208x256x256 matrix). T1-weighted data were segmented using FAST (FMRIB's Automated Segmentation Tool [41]), to obtain volumes of cerebrospinal fluid (CSF), grey matter, and white matter, and to generate grey matter IDPs in 139 regions of interest (ROI). Subcortical structures were modelled using FIRST (FMRIB's Integrated Registration and Segmentation Tool [42]). For the present study, the mean volume of each bilateral structure was calculated over the two hemispheres, and the total cerebellar volume was calculated by adding up the volumes of all cerebellar lobules.

Diffusion imaging was performed using a standard Stejskal-Tanner pulse sequence to acquire 50 distinct diffusion-encoding directions for two diffusion-weighted shells with b values of 1000 and 2000 s/mm^2 (voxel 2x2x2 mm; field-of-view 104x104x72 matrix). The b = 1000 s/mm^2 data were fed into the diffusion-tensor-imaging (DTI) fitting tool (DTIFIT), which created DTI outputs including fractional anisotropy (FA) and mean diffusivity (MD). These outputs were then aligned to a standard-space white-matter skeleton using TBSS (Tract-Based Spatial Statistics [43]), and were averaged across a set of 48 standard-space tract masks defined by the John Hopkins University White Matter Atlas[44]. For the present study, the mean FA and MD of each bilateral structure of interest were calculated over the two hemispheres. Moreover, global FA and MD measures were calculated by averaging these metrics over all white matter tracts per individual.

Cognitive and emotional data

At the assessment centres, participants also completed a series of cognitive tests and questionnaires on a touch screen. For these analyses, six cognitive tasks were selected: reaction time (to assess simple processing speed; expressed as mean time to correctly identify matches), trail making A and B (to test visual attention; expressed as the duration to complete the numeric (A) or alphanumeric (B) path), fluid intelligence (to test reasoning and problem solving; expressed as a fluid intelligence score, which is the number of correct answers given to 13 questions), symbol digit substitution (to

For peer review only - http://bmjopen.bmj.com/site/about/guidelines.xhtml

assess complex processing speed; expressed as the number of symbol digit matches made correctly within two minutes, with no maximum), and digit span (to test numeric working memory; expressed as the maximum digits remembered correctly, with a maximum of 12). For fluid intelligence, symbol digit substitution, and digit span tests, higher scores represent a better cognitive performance, while for reaction time, and trail making A and B, higher scores represent a worse cognitive performance.

Moreover, we analysed four mental health questionnaire items that specifically asked about the participant's situation in the previous two weeks, in which the glucocorticoid users were likely already using glucocorticoid medication. These questions included the frequency of a depressed mood, disinterest, tenseness/restlessness, and tiredness/lethargy in the past two weeks, and were answered using categorical answer options ('Never', 'Several days', 'More than half of the days', or 'Nearly every day'). The entire questionnaire can be found via: https://biobank.ndph.ox.ac.uk/ukb/ukb/docs/TouchscreenQuestionsMainFinal.pdf.

Statistical analysis

Demographic characteristics were presented as mean and standard deviation (SD) or number and percentage and were compared across the three groups using analysis of variance (ANOVA) or chi squared test, respectively.

The primary outcomes of this study were the differences in imaging parameters between glucocorticoid users and controls for a selection of ROIs (22 volumetric parameters, 14 diffusion parameters), that have previously been shown to be affected by long-term glucocorticoid exposure (see Supplement 2). As secondary outcomes, potential differences in cognitive and emotional outcomes between glucocorticoid users and controls were assessed.

For the imaging and cognitive outcomes, multivariable linear regression models were used. The assumption of normality of the residuals was assessed using quantile-quantile (Q-Q) plots and homogeneity of variance across the groups was tested using Levene's test and visually assessed using scatter plots. Subsequently, ANOVA was used to assess whether any differences in outcome parameters existed between oral glucocorticoid users, inhalation glucocorticoid users, and controls. To account for multiple testing, P values were adjusted using the Benjamini-Hochberg false discovery rate (FDR) method. For those parameters with P values < 0.05 after FDR correction, post-hoc Dunnett tests were used to make pairwise comparisons between oral glucocorticoid users vs. controls, and inhalation glucocorticoid users vs. controls.

For multivariable linear models of the imaging parameters, covariates included age, sex, education, a measure of head size (the volumetric scaling from T1 image to standard space, corresponding to the inverse of head size), measures of head position (X-, Y-, and Z-position of the head in the scanner, and table position), assessment centre, and year of imaging acquisition. This

For peer review only - http://bmjopen.bmj.com/site/about/guidelines.xhtml

selection was based on recommendations by the UK Biobank [45], in addition to variables that potentially meet the criteria of a confounder for this study. Because fewer than 1% of the participants had missing values for the covariates, complete case analysis was performed for the analysis of these primary outcomes, and all subsequent analyses.

For the cognitive outcomes, variables with non-normally distributed residuals (reaction time, trail making A, trail making B) were normalized using log transformation. All cognitive outcomes were transformed such that higher values indicate better performance, and then converted into Z scores. The linear models of the cognitive outcomes were adjusted for age, sex, and education.

Since the emotional outcome parameters were categorical, logistic models were used, adjusted for age, sex, and education. Per symptom, the participants who reported a frequency of 'Several days', 'More than half of the days', or 'Nearly every day' were grouped together and were compared with participants who replied 'Never'. The likelihood ratio test was performed to determine whether the proportion of patients experiencing a mental health complaint in the past two weeks differed between the three groups. For those parameters with a statistically significant difference after FDR correction, the odds ratio (OR) of experiencing a mental health complaint in the past two weeks was calculated for each glucocorticoid user group compared to controls. P values pertaining to the ORs were Bonferroni-corrected for multiple testing.

Use of glucocorticoids is associated with weight gain and in particular with an increased body fat percentage [7], which has been reported to affect brain volume and white matter microstructure [46]. Therefore, mediation analysis was performed to test whether the effects of glucocorticoid on brain volume and white matter microstructure were mediated by body fat percentage (as measured by body impedance). For this analysis, all three significantly different volumetric outcomes, and the two (significantly different) global diffusion imaging parameters were considered. The mediation analysis was performed using the *mediation* package, with 1000 simulations and including the same covariates for imaging parameters as above.

Since the doses and duration of medication use are unknown in the UK Biobank, we were unable to perform subgroup analyses based on dose or duration of glucocorticoid use. Because inhalation glucocorticoid are expected to cause, on average, lower systemic concentrations of glucocorticoid than orally administered glucocorticoid [47], the inhalation glucocorticoid users likely represent a group of patients exposed to lower systemic concentrations than patients using oral glucocorticoid and might show less pronounced effects of glucocorticoid on brain parameters. This may give an indication of a dose-dependent effect of glucocorticoid on the brain. In addition, to assess whether we could identify potential duration- or cumulative dose-dependent effect of glucocorticoid use on brain parameters, we performed an additional analysis in the subgroups of glucocorticoid users who reported using glucocorticoid at two different visits (before and including

For peer review only

For peer review only - http://bmjopen.bmj.com/site/about/guidelines.xhtml

the imaging visit) and therefore likely represent a group of chronic or repeated glucocorticoid users. Since the low number of participants in this group resulted in a limited power, we performed the post-hoc tests for these subgroups not only on those parameters that were statistically significant in the ANOVA, but on those parameters assessed by post-hoc tests in the main analysis, because this allowed us to gain insight into the difference in effect size compared to the main analysis.

Lastly, to assess whether outlier values, possibly resulting from poor data quality or processing problems, affected the imaging or cognitive outcomes, the analyses were repeated while excluding outlier values of all outcome parameters (per outcome per study group), defined as more than 1.5 interquartile range (IQR) below the first quartile or above the third quartile. In addition, a sensitivity analysis of all outcome parameters was performed among all participants with imaging data available, without exclusion based on psychiatric, neurological, or endocrinological history, or medication use.

All statistical analyses and data visualization were performed in R (version 4.1.1) [48] using the packages tidyverse (version 1.3.1) [49], car (version 3.0-11) [50], emmeans (version 1.7.0; https://cran.r-project.org/package=emmeans), lmtest (0.9-38) [51], mediation (version 4.5.0) [52], fauxnaif (version 0.6.1; https://cran.r-project.org/package=fauxnaif), ggpubr (version 0.4.0; https://rpkgs.datanovia.com/ggpubr/), and cowplot (version 1.1.1; https://cran.r-project.org/package=cowplot).

Patient and public involvement

Patients and the public were not directly involved in the design or implementation of this study, since we used previously collected data.

For peer review only

For peer review only - http://bmjopen.bmj.com/site/about/guidelines.xhtml

Results

Demographic characteristics

In total, 222 patients using oral glucocorticoids, 557 patients using inhalation glucocorticoids, and 24,106 controls were included. As shown in Table 1, these groups did not differ significantly with respect to sex, education, and smoking status, while the oral glucocorticoid group was slightly older than the other groups (mean age 66.1 ± 7.2 years for oral glucocorticoid users; 63.3 ± 7.5 years for inhalation glucocorticoid users; 63.5 ± 7.5 years for controls), and the inhalation glucocorticoid group had a higher BMI and body fat percentage (Supplement 3.1).

For peer review only

Table 1. Characteristics of included patients using oral glucocorticoids (n = 222), inhalation glucocorticoids (n = 557), and controls

	**Patients using oral GC** (n = 222)	**Patients using inhalation GC** (n = 557)	**Controls** (n = 24106)	**P value**
**Sex: male, n (%)***	111 (50.0%)	253 (45.4%)	12154 (50.4%)	0.066
**Age at time of scanning in years, mean (SD)***	66.1 (7.2)	63.3 (7.5)	63.5 (7.5)	2.4e-6
**Education level, n (%)**				0.66
College/University degree	108 (48.6)	287 (51.5)	12058 (50.0)	
A levels or equivalent	26 (11.7)	66 (11.8)	2930 (12.2)	
O levels/GCSE or equivalent	38 (17.1)	96 (17.2)	4155 (17.2)	
CSEs or equivalent	9 (4.1)	17 (3.1)	879 (3.6)	
NVQ, HND, HNC, or equivalent	6 (2.7)	35 (6.3)	1396 (5.8)	
Other professional qualifications	13 (5.9)	29 (5.2)	1150 (4.8)	
None of the above	18 (8.1)	25 (4.5)	1311 (5.4)	
Missing	4 (1.8)	2 (0.4)	227 (0.9)	
**BMI in kg/m^2, mean (SD)**	26.2 (3.9)	26.7 (4.3)	26.1 (4.1)	1.0e-3
Number (%) missing	7 (3.2)	20 (3.6)	1325 (5.5)	
**Body fat percentage, mean (SD)**	30.9 (7.9)	32.1 (8.3)	30.2 (7.9)	3.5e-8
Number (%) missing	7 (3.2)	20 (3.6)	1331 (5.5)	
**Smoking status, n (%)**				0.44
Current	6 (2.7)	11 (2.0)	647 (2.7)	
Previous	76 (34.2)	200 (35.9)	7858 (32.6)	
Never	137 (61.7)	341 (61.2)	15380 (63.8)	
Missing	3 (1.4)	5 (0.9)	221 (0.9)	

* There were no missing values for the variables sex and age. BMI, body mass index; GC, glucocorticoids; n, number; SD, standard deviation.

For peer review only

Volumetric imaging parameters

Fifteen out of 22 predefined ROIs for the volumetric imaging were significantly different across the groups according to the ANOVA (Supplement 3.2). However, none of the 'global volume' parameters reached statistical significance in the post-hoc tests (Table 2). With respect to 'subcortical volumes', the caudate was larger in oral glucocorticoid users compared to controls (adjusted mean difference (AMD) = 77.8 mm^3, 95% confidence interval (CI) = 24.5 to 131.1). None of the subcortical volumes (containing both grey and white matter) differed significantly between inhalation glucocorticoid users and controls. Of the 'regional grey matter volumes', the caudate was larger in oral glucocorticoid users compared to controls (AMD = 178.7 mm^3, 95% CI = 82.2 to 275.0), and inhalation glucocorticoid users had smaller grey matter volumes in the amygdala (AMD = -23.9 mm^3, 95% CI =-41.5 to -6.2).

To assess whether chronic or repeated glucocorticoid exposure was associated with greater changes in imaging parameters, subgroup analyses among chronic oral glucocorticoid users (n = 42) and chronic inhalation glucocorticoid users (n = 305) were performed (demographic characteristics are presented in Supplement 4). As expected, only few of the investigated imaging parameters reached statistical significance due to the lower power resulting from the smaller group sizes than in the main analysis (Supplement 3.3). Nevertheless, in chronic oral glucocorticoid users, global volumes showed the same patterns of reduction as in the main analysis, and the caudate showed a larger increase in subcortical volume, but a smaller increase in grey matter volume. For chronic inhalation glucocorticoid users, the patterns were like those in the main analysis, with no striking differences in effect sizes (Supplements 3.3, 5, and 6).

For peer review only

Table 2. Imaging parameters, presented as the adjusted mean difference of patients using oral glucocorticoids (n = 222) or inhalation glucocorticoids (n = 557) compared to controls (n = 24106)

	ANOVA			Oral GC vs. controls			Inhalation GC vs. controls		
	**F value**	**P value**	**P_{FDR}**	**AMD***	**95% CI**	**P value**	**AMD***	**95% CI**	**P value**
Volumetric measures									
Global volumes (in mm^3)									
Total brain volume	19.7	2.8e-9	**1.0e-8**	-3688	-10627; 3252	0.39	3374	-1012; 7760	0.16
Grey matter volume	23.7	5.4e-11	**6.5e-10**	-1968	-5904; 1968	0.43	1012	-1476; 3500	0.57
White matter volume	6.7	0.0012	**0.0020**	-1720	-6273; 2833	0.61	2362	-516; 5240	0.13
Peripheral cortex	21.1	6.9e-10	**6.2e-9**	-3303	-6843; 237	0.072	1033	-1205; 3270	0.49
CSF volume	10.1	4.2e-5	**9.5e-5**	1215	-824; 3254	0.32	78	-1211; 1367	0.98
Subcortical volumes (in mm^3)									
Accumbens	12.0	6.0e-6	**1.7e-5**	-13.1	-26.7; 0.5	0.062	-6.5	-15.1; 2.1	0.17
Caudate	6.7	0.00126	**0.0020**	77.8	24.5; 131.1	**0.0023**	-2.7	-36.4; 30.9	0.97
Pallidum	7.7	4.5e-4	**7.8e-4**	0.8	-29.9; 31.4	1.00	-18.0	-37.3; 1.42	*0.074*
Putamen	10.9	1.8e-5	**4.6e-5**	-31.3	-98.2; 35.6	0.48	-27.9	-70.2; 14.4	0.25
Thalamus	8.2	2.7e-4	**4.9e-4**	3.6	-74.0; 81.1	0.99	-6.4	-55.4; 42.6	0.93
Regional grey matter volumes (in mm^3)									
Amygdala	23.8	5.0e-11	**6.5e-10**	-4.0	-31.9; 23.8	0.91	-23.9	-41.5; -6.2	**0.0052**
Caudate	13.0	2.3-6	**7.5e-6**	178.7	82.2; 275.0	**0.00010**	41.2	-19.8; 102.0	0.24
Cerebellum	10.8	2.0e-5	**4.8e-5**	25.1	-18.4; 68.5	0.34	-12.2	-39.7; 15.3	0.51
Insular cortex	8.5	2.0e-4	**3.9e-4**	-36.2	-108.4; 36.0	0.43	5.0	-40.6; 50.7	0.95

For peer review only

Precuneal cortex	5.5	0.0043	**0.0056**	-21.5	-179.0; 136.3	0.92	-7.4	-107.0; 92.4	0.97
DTI measures									
Fractional anisotropy									
Global	19.2	4.6e-9	**2.8e-8**	-0.0037	-0.0064; -0.0010	**0.0042**	-0.0023	-0.0040; -5.7e-4	**0.0057**
Body of corpus callosum	10.0	4.7e-5	**1.0e-4**	-0.0043	-0.0084; -1.2e-4	**0.043**	-0.0023	-0.0049; 3.0e-4	0.092
Genu of corpus callosum	16.8	5.4e-8	**2.1e-7**	-0.0064	-0.011; -0.0017	**0.0050**	-0.0019	-0.0049; 0.0011	0.27
Splenium of corpus callosum	5.4	0.0044	**0.0056**	-0.0021	-0.0053; 0.0012	0.27	-0.0032	-0.0052; -0.0012	**0.0010**
Cingulum cingulate	6.1	0.0024	**0.0034**	-0.0017	-0.0062; 0.0028	0.61	-0.0028	-0.0057; 8.9e-6	*0.051*
Cingulum hippocampus	6.4	0.0017	**0.0025**	6.5e-5	-0.0046; 0.0048	1.00	-3.4e-3	-0.0063; -3.8e-4	**0.024**
Mean diffusivity									
Global	25.9	5.8e-12	**2.1e-10**	7.2e-6	3.2e-6; 1.1e-5	**0.0001**	2.7e-6	1.7e-7; 5.2e-6	**0.034**
Body of corpus callosum	15.5	2.0e-7	**7.0e-7**	6.9e-6	1.7e-6; 1.2e-5	**0.0060**	4.8e-6	1.6e-6; 8.1e-6	**0.0020**
Genu of corpus callosum	18.0	1.6e-8	**7.0e-8**	8.4e-6	2.2e-6; 1.5e-5	**0.0049**	4.1e-6	1.7e-7; 8.0e-6	**0.039**
Splenium of corpus callosum	9.7	6.2e-5	**1.2e-4**	4.4e-6	-3.8e-8; 8.9e-6	0.050	5.3e-6	2.4e-6; 8.1e-6	**0.00010**
Cingulum cingulate	5.4	0.0043	**0.0056**	2.9e-6	-8.5e-7; 6.6e-6	0.16	2.8e-6	4.7e-7; 5.2e-6	**0.015**
Cingulum hippocampus	18.5	9.1e-9	**4.7e-8**	5.0e-6	4.2e-7; 9.5e-6	**0.029**	5.6e-6	2.8e-6; 8.5e-6	**<.00010**
Uncinate fasciculus	12.1	5.4e-6	**1.6e-5**	6.4e-6	2.2e-6; 1.1e-5	**0.0014**	2.2e-6	-4.4e-7; 4.9e-6	0.12

* Adjusted mean difference, calculated using linear models, adjusted for age, sex, education, X-, Y-, and Z-position of the head in the scanner, head size, assessment centre, and year of imaging acquisition.

CI, confidence interval; P_{FDR}, Benjamini-Hochberg false discovery rate corrected P values. P values in bold are statistically significant ($P < 0.05$).

For peer review only

Diffusion imaging parameters

All but one of the diffusion imaging parameters differed significantly across the groups. Post-hoc tests showed that global FA was reduced in oral glucocorticoid users (AMD = -3.7e-3, 95% CI = -6.4e-3 to 1.0e-3), and reductions in regional FA were observed in the body and genu of the corpus callosum (Table 2, Figure 2). Similarly, global FA was reduced in inhalation glucocorticoid users (AMD = -2.3e-3, 95% CI = -4.0e-3 to -5.7e-4), and the splenium of the corpus callosum and the cingulum of the hippocampus also showed a lower FA. For most ROIs, reductions in FA were smaller in inhalation glucocorticoid users than in oral glucocorticoid users.

Furthermore, global MD was higher in oral glucocorticoid users (AMD = 7.2e-6, 95% CI = 3.2e-6 to 1.1e-5) and inhalation glucocorticoid users compared to controls (AMD = 2.7e-6, 95% CI = 1.7e-7 to 5.2e-6). In oral glucocorticoid users, increases in regional MD were observed in the body and genu of the corpus callosum, the cingulum of the hippocampus, and the uncinate gyrus. For inhalation glucocorticoid users, the increase in MD was significant in the body, genu, and splenium of the corpus callosum, the cingulum of the cingulate cortex, and the cingulum of the hippocampus. Again, effect sizes were similar or smaller for most tracts compared to the effects observed in oral glucocorticoid users.

For chronic glucocorticoid users, the tendencies of FA and MD outcomes were in the same direction as the main analysis for all ROIs. Almost all associations with global and regional FA and MD showed a greater effect size among chronic oral glucocorticoid users than in the main analysis, although only the global FA and MD measures, and FA and MD in the genu of the corpus callosum reached significance. In chronic inhalation glucocorticoid users, however, the effect sizes were not remarkably different from those observed in the main analysis (Supplements 3.3, 5, and 6).

Cognitive and emotional outcomes

ANOVA showed differences between the groups on three cognitive tasks: trail making A, trail making B, and symbol substitution (Supplement 3.4). Post-hoc testing revealed that oral glucocorticoid users performed significantly worse on the symbol digit substitution task compared to controls (AMD = -0.17 SD, 95% CI = -0.34 to -0.01; Table 3). With regards to the emotional outcomes, between-group differences were observed in the frequency of depressive symptoms (P = 0.0049), disinterest (P = 0.0049), tenseness/restlessness (P = 0.0025) and tiredness/lethargy (P = 3.7e-7) (Supplements 3.5 and 7). Pairwise comparisons using logistic regression analysis revealed that oral glucocorticoid users experienced more depressive symptoms (OR = 1.76, 95% CI = 1.25 to 2.43), disinterest (OR = 1.84, 95% CI = 1.29 to 2.56), tenseness/restlessness (OR = 1.78, 95% CI = 1.29 to 2.41), and tiredness/lethargy (OR = 1.90, 95% CI = 1.45 to 2.50) compared to controls (Table 4), while inhalation

For peer review only

For peer review only - http://bmjopen.bmj.com/site/about/guidelines.xhtml

glucocorticoid users only reported more tiredness/lethargy than controls (OR = 1.35, 95% CI = 1.14 to 1.60).

For the chronic users, none of the cognitive outcomes was significantly different in oral or inhalation glucocorticoid users compared to controls in the post-hoc analysis. Effect sizes for chronic oral glucocorticoid users were even smaller than in the entire cohort, while two out of three were slightly larger in the chronic inhalation glucocorticoid users compared to the entire cohort (Supplement 3.6, Supplement 7). Likewise, the emotional outcome parameters did not differ significantly, except for tiredness/lethargy which was more common in inhalation glucocorticoid users compared to controls. Remarkably, most odds ratios were lower than in the main analysis (Supplements 3.7, 8, and 9).

For peer review only

For peer review only - http://bmjopen.bmj.com/site/about/guidelines.xhtml

Table 3. Cognitive outcome measures of patients using oral glucocorticoids (n = 222) or inhalation glucocorticoids (n = 557) compared to controls

	**ANOVA**			**Oral GC vs. controls**			**Inhalation GC vs. controls**			**Participants with available data, n (%)**		
	**F value**	**P value**	**P_{FDR}**	**AMD***	**95% CI**	**P value**	**AMD***	**95% CI**	**P value**	**Oral GC**	**Inhalation GC**	**Controls**
**Trail making A**	5.6	0.0036	**0.0073**	-0.11	-0.28; 0.06	0.25	-0.031	-0.15; 0.09	0.78	149 (67)	296 (53)	16419 (68)
**Trail making B**	6.1	0.0023	**0.0068**	-0.12	-0.30; 0.05	0.19	-0.0077	-0.13; 0.11	0.98	139 (63)	291 (52)	16071 (67)
**Symbol substitution**	10.3	3.5e-5	**2.1e-4**	-0.17	-0.34; -0.01	**0.038**	-0.035	-0.15; 0.08	0.72	146 (66)	298 (54)	16442 (68)

For peer review only

* Adjusted mean difference between patients and controls, expressed in Z scores. Calculated using linear models, adjusting for age, sex, and education. Trail making A, and trail making B were log transformed before generation of Z scores because they were non-normally distributed. Variables were transformed such that higher values indicate a better performance.

CI, confidence interval; GC, glucocorticoids; n, number; P_{FDR}, Benjamini-Hochberg false discovery rate corrected P values.

P values in bold are statistically significant (P < 0.05).

For peer review only - http://bmjopen.bmj.com/site/about/guidelines.xhtml

353 **Table 4**. Likelihood of experiencing mental health complaints in the past two weeks of oral
354 glucocorticoid users (n = 222) and inhalation glucocorticoid users (n = 557) compared to controls
355

	Likelihood ratio test			Oral GC vs. controls			Inhalation GC vs. controls		
	X^2	P value	P_{FDR}	OR	95% CI	P value	OR	95% CI	P value
**Depression**	10.6	0.0049	0.0049	1.76	1.25; 2.43	**8.2e-4**	1.10	0.87; 1.38	0.43
**Disinterest**	10.9	0.0043	0.0049	1.84	1.29; 2.56	**5.1e-4**	1.06	0.82; 1.36	0.64
**Tenseness**	13.4	0.0012	0.0025	1.78	1.29; 2.41	**3.0e-4**	1.16	0.92; 1.43	0.19
**Tiredness**	32.4	9.2e-8	3.7e-7	1.90	1.45; 2.50	**4.4e-6**	1.35	1.14; 1.60	**6.3e-4**

356

357 Calculated using logistic regression analysis, adjusting for age, sex, and education.
358 P values in bold are statistically significant after Bonferroni correction for family-wise error rate of
359 two tests (P < 0.025).
360 CI, confidence interval; GC, glucocorticoids; OR, odds ratio; P_{FDR}, Benjamini-Hochberg false discovery
361 rate corrected P values.
362
363

For peer review only

Sensitivity analyses

In the first sensitivity analysis we included the subjects that were previously excluded based on neurological, psychiatric, or endocrine history or medication use. The imaging outcomes were comparable to those of the main analysis, with similar ROIs being significantly different between the groups (Supplements 3.8 to 3.10, and Supplements 10 to 14), although the differences in diffusion parameters of between glucocorticoid users and controls were more pronounced in the main analysis than in the unselected group. The same was observed for the cognitive and emotional outcomes.

For the second sensitivity analysis, outliers of the imaging and cognitive outcomes (<3% for most parameters) were excluded (Supplements 3.11 to 3.14, and Supplements 15 and 16), which led to the same conclusions for the imaging outcomes, except for a small number of regions that had shown a tendency in the main analysis and reached significance after exclusion of outlier values (subcortical accumbens volume, insular grey matter volume, and MD in the splenium of the corpus callosum; all in oral glucocorticoid users). For the cognitive outcomes, exclusion of outliers resulted in not only a significant reduced score on the symbol digit substitution test, but also on the trail making B test for the oral glucocorticoid users.

Mediation analyses

To assess whether total body fat percentage could have mediated the effect of glucocorticoids on the brain, mediation analysis was performed. For none of the investigated imaging outcomes, a significant mediation effect by body fat percentage was found (Supplement 3.15), suggesting that the observed effects of glucocorticoids were independent of body fat.

For peer review only

Discussion

This study shows that in the large population-based cohort of the UK Biobank, the use of not only oral glucocorticoids but also inhalation glucocorticoids is associated with changes in several brain imaging parameters. Most notably, the previously reported glucocorticoid effects on white matter microstructure [32] were also detected in this population and are therefore likely to be widespread amongst glucocorticoid users. Subgroup analyses among people using chronic glucocorticoids suggested a potential dose- or duration-dependent effect of glucocorticoids on white matter microstructure, with smallest effect sizes in inhalation glucocorticoid users, larger effect sizes in oral glucocorticoid users, and the largest effect sizes in chronic oral glucocorticoid users. While it remains unclear whether the observed effect sizes have clinical consequences for the population of glucocorticoid users as a whole, these findings are remarkable given the common neuropsychiatric side effects of synthetic glucocorticoids, and the observed changes may play a role in those patients suffering from these side effects.

Findings in context

Previous studies in people exposed to high levels of endogenous glucocorticoid due to Cushing disease or high-dose synthetic oral glucocorticoids have shown that glucocorticoid overexposure is associated with global cerebral atrophy and cortical thinning, as well as volumetric changes in specific brain areas. For example, reductions of grey matter volume have been observed in the hippocampus [14, 24, 25, 27, 30, 31, 53], amygdala [18, 28, 54], cingulate cortex [13, 22, 23], insula [13], caudate [19], and cerebellum [17, 25, 26], which have all been implicated in cognitive processes and emotional regulation [55-60]. However, not all findings were consistent across studies, which may in part be due to differences between patient populations (e.g., with respect to duration and type of glucocorticoid exposure), the small sample sizes of the studies, and the different analysis methods used, with some studies only focussing on one specific brain region, and others performing whole-brain analysis. In general, studies have mainly been dedicated to structural imaging with a specific interest in grey matter volume, while diffusion imaging has only been performed by only a few studies in patients with Cushing disease [32-35].

The present study extends these findings by investigating brain volumes and white matter microstructure in not only systemic (oral) glucocorticoid users, but also inhalation glucocorticoid users, in whom neuropsychiatric side effects have been reported too [61]. The most remarkable and consistent effects were observed in white matter integrity, which was decreased in both oral and inhalation glucocorticoid users. Although these reductions were only about 10% of the effect sizes previously found in Cushing patients [32], this adds to the growing body of literature suggesting that glucocorticoids have important impact on white matter, and that non-neuronal cells such as

For peer review only - http://bmjopen.bmj.com/site/about/guidelines.xhtml

oligodendrocytes are very sensitive to glucocorticoids. Animal studies have shown that glucocorticoid exposure inhibits proliferation of oligodendrocyte progenitor cells throughout the white matter [62], and induce changes in the expression of myelin basic protein (MBP), an oligodendrocyte marker [36, 63]. Since oligodendrocytes are responsible for myelin production, glucocorticoid-induced changes in oligodendrocytes may underly the reduced white matter microstructure observed in patients using glucocorticoids. Besides oligodendrocytes, other glia cells including microglia and astrocytes are also affected by glucocorticoids, with multiple reports of decreased cell viability, proliferation, and immunoreactivity of microglia and astrocytes in response to glucocorticoids [64-67].

Although we observed some patterns in global and regional brain volumes in glucocorticoid users, most of these did not reach significance. Rather surprisingly, although none of the global volumes was significantly different between patients and controls, the direction of change for all the areas was different for oral (decreased volumes) vs. inhalation glucocorticoid users (increased volumes). We did observe a significant reduction in grey matter volume of the amygdala in inhalation glucocorticoid users, and an increase in total and grey matter volume of the caudate nucleus in oral glucocorticoid users. Decreased amygdala volumes have previously been reported in chronic oral glucocorticoid users [18, 28, 54]. However, the increase in caudate volume contrasts with two previous studies that found larger caudate volumes after treatment of Cushing disease compared to during active disease [19, 20], while one other study reported an increased caudate volume in remitted patients compared to controls, but no differences in patients with active Cushing disease compared to controls [21]. Those findings suggest that cortisol excess caused a decreased caudate volume in these patients and/or that the caudate volume increased in response to normalization of cortisol levels. The modest effects of glucocorticoids on brain volumes in the present population-based cohort study could indicate that white matter integrity is more sensitive to glucocorticoids than grey matter volume, and that longer or higher glucocorticoid exposure is needed to also induce volumetric changes.

For peer review only

Potential consequences and implications

It is well-known that exogenous glucocorticoids are associated with neuropsychiatric side effects, including not only potentially severe mood disturbances such as depression and mania, but also cognitive impairment such as concentration and memory problems [10]. In the present study, glucocorticoid users reported a higher frequency of several mental health complaints, while their cognitive performance was not significantly different, except for worse scores on the symbol digit substitution task in oral glucocorticoid users. It should be noted that only a few mood-related parameters assessed by the UK Biobank were selected for this study, because these were the only parameters that applied specifically to the previous two weeks, in which the glucocorticoid users

For peer review only - http://bmjopen.bmj.com/site/about/guidelines.xhtml

were likely already using their medication. Ideally, more aspects of mood would have been assessed to get a more comprehensive view on the glucocorticoid users' psychological functioning. Furthermore, the observed mood-related effects may not be caused by glucocorticoid use per se but could also be related to the condition for which glucocorticoids were prescribed. For example, autoimmune and inflammatory diseases commonly treated with glucocorticoids, such as rheumatoid arthritis and chronic obstructive pulmonary disorder, have also been associated with mental health impairment and reduced quality of life [68, 69].

Nevertheless, awareness for the potential of glucocorticoids to affect the brain and cause neuropsychiatric symptoms is important, since these medications are prescribed for a wide range of conditions by many different medical specialties and are used by a substantial proportion of the population. Moreover, further research into the underlying mechanisms, reversibility, and risk factors for development of neuropsychiatric side effects of glucocorticoids is warranted, ideally considering dose and duration of glucocorticoids, as well as single nucleotide polymorphisms (SNPs) in the glucocorticoid receptor gene (NR3C1) that affect glucocorticoid sensitivity. For those patients experiencing side effects, alternative treatment options should also be investigated. One promising direction is the development of selective GR modulators, since these (ideally) only activate the desired downstream signalling pathways in the desired cell types, limiting the potential side effects [70, 71].

Strengths and limitations

To the best of our knowledge, this is the largest study to date assessing the effects of glucocorticoid use on the brain, and the first to investigate these effects in inhalation glucocorticoid users. For the selection of patients and controls, we applied relatively strict exclusion criteria to limit the potential confounding that may arise in observational cohort studies. Although not all neurological disorders, especially peripheral disorders, may have a clear impact on brain volume or white matter microstructure, UK Biobank participants with these conditions were excluded to prevent any confounding by these comorbidities. Our sensitivity analysis suggested that these conditions did not have a large impact on the results. However, we decided not to exclude patients with a history of depression, anxiety, mania, or delirium, because these are known possible consequences of glucocorticoid use [10], and we did not want to exclude patients based on potentially glucocorticoid-related outcomes.

Another method used to limit confounding was adjustment of the regression analyses for relevant confounding variables, including demographic variables and variables related to the imaging visits (e.g., assessment centre, position of the head in the scanner). For both the volumetric and diffusion parameters, head size was used as covariate, because previous research not only found a

For peer review only

For peer review only - http://bmjopen.bmj.com/site/about/guidelines.xhtml

relation between head size and brain volume, but also between head size and DTI parameters [72, 73]. The use of this variable as covariate is also recommended by the UK Biobank [45]. We decided not to include a measure of body weight or body composition as covariate, because it is known that glucocorticoids can cause obesity [7], which is therefore more likely to be in the causal pathway than to be a confounder. Our mediation analysis, however, suggested that body fat percentage did not mediate the effect of glucocorticoids on the brain. Nevertheless, despite the correction for a wide range of potential confounders, it should be noted that the possibility of residual confounding cannot be excluded.

In addition, although a causal relation between glucocorticoid use and changes in the brain is likely based on the present and previous studies, the cross-sectional nature of this study officially does not allow for conclusions on causality. Demonstrating a dose-response effect of glucocorticoid on imaging parameters would have increased the likelihood of a causal relation, but unfortunately, dose and duration of medication use were not available in the UK Biobank. We were therefore only able to give an indication of a dose-response effect by performing separate analyses in oral glucocorticoid users, inhalation glucocorticoid users (representing a group exposed to lower systemic concentration of glucocorticoids), and subgroups of patients using oral or inhalation glucocorticoid chronically (representing groups with a longer duration of glucocorticoid use). The fact that the effect sizes of the associations between glucocorticoid use and diffusion imaging parameters are generally largest in the chronic oral glucocorticoid group, and smallest in the inhalation glucocorticoid group, indicates that a dose- or duration-dependent effect may exist, although the limited power of the small chronic oral glucocorticoid group precluded most associations from reaching significance. Moreover, while effect estimates were larger in chronic oral glucocorticoid users compared to the main group using oral glucocorticoids, this difference was not observed among inhalation glucocorticoid users. A potential explanation may be that inhalation glucocorticoids are generally prescribed for a longer duration than oral glucocorticoids, which is also reflected by the high percentage of inhalation glucocorticoid users (326/592, 55%) that could be included in the subgroup of chronic users, compared to the lower percentage of chronic oral glucocorticoid users (48/234, 21%).

Conclusion

This study shows that both oral and inhalation glucocorticoids are associated with decreased white matter integrity, which may in part underly the neuropsychiatric side effects observed in patients using glucocorticoids. Since these medications are widely used, awareness of these effects is necessary across medical specialties, and research into alternative treatment options is warranted.

For peer review only

Declarations

Author contribution statement: JMA and OCM contributed to the study conception. MvdM designed the study, performed the analyses, and wrote the first version of the manuscript. OMD contributed to the statistical analyses. All authors read and commented on the manuscript and approved the final version of the manuscript. MvdM and OCM are the guarantors of the manuscript and accept full responsibility for the work and conduct of the study, had access to the data, and controlled the decision to publish. The corresponding author attests that all listed authors meet authorship criteria and that no others meeting the criteria have been omitted.

Acknowledgements: We would like to thank dr. Roula Tsonaka for her advice regarding the statistical analysis, and dr. Steven van der Werff for his suggestion for this study.

Competing interests: All authors have completed the ICMJE uniform disclosure form at http://www.icmje.org/disclosure-of-interest/ and declare: MvdM received financial support from the MD/PhD grant of the Leiden University Medical Center, and JMA received financial support from CONACyT (the National Council for Science and Technology-Government of Mexico) for the submitted work; OCM has received research grants and honorariums from Corcept Therapeutics, and a speakers' fee from Ipsen; no other relationships or activities that could appear to have influenced the submitted work.

Funding: The UK Biobank was established by the Wellcome Trust, Medical Research Council, Department of Health, Scottish government, and Northwest Regional Development Agency. It also received funding from the Welsh Government, the British Heart Foundation, Cancer Research UK, and Diabetes UK. For the analyses presented in this manuscript, MvdM received a personal MD/PhD grant of the Leiden University Medical Center, and JMA received support from CONACyT (the National Council for Science and Technology-Government of Mexico).

Role of the funding source: The funding sources had no role in the study conduct, data collection, analyses, data interpretation, and the decision to submit the manuscript.

Ethics approval: This study was performed under the ethical approval obtained by UK Biobank from the National Health Service National Research Ethics Service (ref 11/NW/0382, 17 June 2011). Data for the present study were obtained from the UK Biobank under application number 59004.

For peer review only

For peer review only - http://bmjopen.bmj.com/site/about/guidelines.xhtml

Transparency declaration: The manuscript's guarantors (MvdM and OCM) affirm that the manuscript is an honest, accurate, and transparent account of the study being reported; that no important aspects of the study have been omitted; and that any discrepancies from the study as planned have been explained.

Data sharing statement: Data used for this study are available via application to the UK Biobank.

Dissemination to participants and related patient and public communities: Results of the study will be disseminated via the UK Biobank website, accessible for research participants and relevant patient and public communities.

STROBE checklist for observational studies can be found in Supplement 17.

For peer review only

References

1. Laugesen K, Jørgensen JOL, Petersen I, Sørensen HT. Fifteen-year nationwide trends in systemic glucocorticoid drug use in Denmark. Eur J Endocrinol. 2019;181(3):267-73.
2. Fardet L, Petersen I, Nazareth I. Prevalence of long-term oral glucocorticoid prescriptions in the UK over the past 20 years. Rheumatology (Oxford). 2011;50(11):1982-90.
3. Overman RA, Yeh JY, Deal CL. Prevalence of oral glucocorticoid usage in the United States: a general population perspective. Arthritis Care Res (Hoboken). 2013;65(2):294-8.
4. van Staa TP, Leufkens HG, Abenhaim L, Begaud B, Zhang B, Cooper C. Use of oral corticosteroids in the United Kingdom. Qjm. 2000;93(2):105-11.
5. Walsh LJ, Wong CA, Pringle M, Tattersfield AE. Use of oral corticosteroids in the community and the prevention of secondary osteoporosis: a cross sectional study. Bmj. 1996;313(7053):344-6.
6. Rhen T, Cidlowski JA. Antiinflammatory action of glucocorticoids--new mechanisms for old drugs. N Engl J Med. 2005;353(16):1711-23.
7. Fardet L, Fève B. Systemic glucocorticoid therapy: a review of its metabolic and cardiovascular adverse events. Drugs. 2014;74(15):1731-45.
8. Chalitsios CV, Shaw DE, McKeever TM. Risk of osteoporosis and fragility fractures in asthma due to oral and inhaled corticosteroids: two population-based nested case-control studies. Thorax. 2021;76(1):21-8.
9. Savas M, Muka T, Wester VL, van den Akker ELT, Visser JA, Braunstahl GJ, et al. Associations Between Systemic and Local Corticosteroid Use With Metabolic Syndrome and Body Mass Index. J Clin Endocrinol Metab. 2017;102(10):3765-74.
10. Judd LL, Schettler PJ, Brown ES, Wolkowitz OM, Sternberg EM, Bender BG, et al. Adverse consequences of glucocorticoid medication: psychological, cognitive, and behavioral effects. Am J Psychiatry. 2014;171(10):1045-51.
11. Fardet L, Petersen I, Nazareth I. Suicidal behavior and severe neuropsychiatric disorders following glucocorticoid therapy in primary care. Am J Psychiatry. 2012;169(5):491-7.
12. Bourdeau I, Bard C, Noël B, Leclerc I, Cordeau MP, Bélair M, et al. Loss of brain volume in endogenous Cushing's syndrome and its reversibility after correction of hypercortisolism. J Clin Endocrinol Metab. 2002;87(5):1949-54.
13. Hou B, Gao L, Shi L, Luo Y, Guo X, Young GS, et al. Reversibility of impaired brain structures after transsphenoidal surgery in Cushing's disease: a longitudinal study based on an artificial intelligence-assisted tool. J Neurosurg. 2020:1-10.
14. Chen Y, Zhang J, Tan H, Li J, Yu Y. Detrimental effects of hypercortisolism on brain structure and related risk factors. Sci Rep. 2020;10(1):12708.
15. Santos A, Resmini E, Gómez-Ansón B, Crespo I, Granell E, Valassi E, et al. Cardiovascular risk and white matter lesions after endocrine control of Cushing's syndrome. Eur J Endocrinol. 2015;173(6):765-75.
16. Simmons NE, Do HM, Lipper MH, Laws ER, Jr. Cerebral atrophy in Cushing's disease. Surg Neurol. 2000;53(1):72-6.
17. Momose KJ, Kjellberg RN, Kliman B. High incidence of cortical atrophy of the cerebral and cerebellar hemispheres in Cushing's disease. Radiology. 1971;99(2):341-8.
18. Merke DP, Giedd JN, Keil MF, Mehlinger SL, Wiggs EA, Holzer S, et al. Children experience cognitive decline despite reversal of brain atrophy one year after resolution of Cushing syndrome. J Clin Endocrinol Metab. 2005;90(5):2531-6.
19. Starkman MN, Giordani B, Gebarski SS, Berent S, Schork MA, Schteingart DE. Decrease in cortisol reverses human hippocampal atrophy following treatment of Cushing's disease. Biol Psychiatry. 1999;46(12):1595-602.
20. Starkman MN, Giordani B, Gebarski SS, Schteingart DE. Improvement in learning associated with increase in hippocampal formation volume. Biol Psychiatry. 2003;53(3):233-8.

For peer review only

For peer review only - http://bmjopen.bmj.com/site/about/guidelines.xhtml

21. Jiang H, Ren J, He NY, Liu C, Sun YH, Jian FF, et al. Volumetric magnetic resonance imaging analysis in patients with short-term remission of Cushing's disease. Clin Endocrinol (Oxf). 2017;87(4):367-74.
22. Bauduin S, van der Pal Z, Pereira AM, Meijer OC, Giltay EJ, van der Wee NJA, et al. Cortical thickness abnormalities in long-term remitted Cushing's disease. Transl Psychiatry. 2020;10(1):293.
23. Andela CD, van der Werff SJ, Pannekoek JN, van den Berg SM, Meijer OC, van Buchem MA, et al. Smaller grey matter volumes in the anterior cingulate cortex and greater cerebellar volumes in patients with long-term remission of Cushing's disease: a case-control study. Eur J Endocrinol. 2013;169(6):811-9.
24. Toffanin T, Nifosì F, Follador H, Passamani A, Zonta F, Ferri G, et al. Volumetric MRI analysis of hippocampal subregions in Cushing's disease: a model for glucocorticoid neural modulation. Eur Psychiatry. 2011;26(1):64-7.
25. Burkhardt T, Lüdecke D, Spies L, Wittmann L, Westphal M, Flitsch J. Hippocampal and cerebellar atrophy in patients with Cushing's disease. Neurosurg Focus. 2015;39(5):E5.
26. Santos A, Resmini E, Crespo I, Pires P, Vives-Gilabert Y, Granell E, et al. Small cerebellar cortex volume in patients with active Cushing's syndrome. Eur J Endocrinol. 2014;171(4):461-9.
27. Brown ES, D JW, Frol A, Bobadilla L, Khan DA, Hanczyc M, et al. Hippocampal volume, spectroscopy, cognition, and mood in patients receiving corticosteroid therapy. Biol Psychiatry. 2004;55(5):538-45.
28. Desai S, Khanani S, Shad MU, Brown ES. Attenuation of amygdala atrophy with lamotrigine in patients receiving corticosteroid therapy. J Clin Psychopharmacol. 2009;29(3):284-7.
29. Brown ES, Kulikova A, Van Enkevort E, Nakamura A, Ivleva EI, Tustison NJ, et al. A randomized trial of an NMDA receptor antagonist for reversing corticosteroid effects on the human hippocampus. Neuropsychopharmacology. 2019;44(13):2263-7.
30. Nguyen DM, Yassa MA, Tustison NJ, Roberts JM, Kulikova A, Nakamura A, et al. The Relationship Between Cumulative Exogenous Corticosteroid Exposure and Volumes of Hippocampal Subfields and Surrounding Structures. J Clin Psychopharmacol. 2019;39(6):653-7.
31. Brown ES, Jeon-Slaughter H, Lu H, Jamadar R, Issac S, Shad M, et al. Hippocampal volume in healthy controls given 3-day stress doses of hydrocortisone. Neuropsychopharmacology. 2015;40(5):1216-21.
32. van der Werff SJ, Andela CD, Nienke Pannekoek J, Meijer OC, van Buchem MA, Rombouts SA, et al. Widespread reductions of white matter integrity in patients with long-term remission of Cushing's disease. Neuroimage Clin. 2014;4:659-67.
33. Pires P, Santos A, Vives-Gilabert Y, Webb SM, Sainz-Ruiz A, Resmini E, et al. White matter alterations in the brains of patients with active, remitted, and cured cushing syndrome: a DTI study. AJNR Am J Neuroradiol. 2015;36(6):1043-8.
34. Jiang H, He NY, Sun YH, Jian FF, Bian LG, Shen JK, et al. Altered gray and white matter microstructure in Cushing's disease: A diffusional kurtosis imaging study. Brain Res. 2017;1665:80-7.
35. Pires P, Santos A, Vives-Gilabert Y, Webb SM, Sainz-Ruiz A, Resmini E, et al. White matter involvement on DTI-MRI in Cushing's syndrome relates to mood disturbances and processing speed: a case-control study. Pituitary. 2017;20(3):340-8.
36. Amaya JM, Suidgeest E, Sahut-Barnola I, Dumontet T, Montanier N, Pagès G, et al. Effects of Long-Term Endogenous Corticosteroid Exposure on Brain Volume and Glial Cells in the AdKO Mouse. Front Neurosci. 2021;15:604103.
37. Sudlow C, Gallacher J, Allen N, Beral V, Burton P, Danesh J, et al. UK Biobank: an open access resource for identifying the causes of a wide range of complex diseases of middle and old age. PLoS Med. 2015;12(3):e1001779.
38. UK Biobank. UK Biobank Body Composition Measurement Version 1.0 2011 [Available from: https://biobank.ndph.ox.ac.uk/ukb/ukb/docs/body_composition.pdf.
39. Alfaro-Almagro F, Jenkinson M, Bangerter NK, Andersson JLR, Griffanti L, Douaud G, et al. Image processing and Quality Control for the first 10,000 brain imaging datasets from UK Biobank. Neuroimage. 2018;166:400-24.

For peer review only - http://bmjopen.bmj.com/site/about/guidelines.xhtml

40. Smith SM, Alfaro-Almagro F, Miller KL. UK Biobank Brain Imaging Documentation 2018 [cited 2021. Available from: biobank.ctsu.ox.ac.uk/crystal/docs/brain_mri.pdf.
41. Zhang Y, Brady M, Smith S. Segmentation of brain MR images through a hidden Markov random field model and the expectation-maximization algorithm. IEEE Trans Med Imaging. 2001;20(1):45-57.
42. Patenaude B, Smith SM, Kennedy DN, Jenkinson M. A Bayesian model of shape and appearance for subcortical brain segmentation. Neuroimage. 2011;56(3):907-22.
43. Smith SM, Jenkinson M, Johansen-Berg H, Rueckert D, Nichols TE, Mackay CE, et al. Tract-based spatial statistics: voxelwise analysis of multi-subject diffusion data. Neuroimage. 2006;31(4):1487-505.
44. Mori S, Wakana S, Van Zijl PC, Nagae-Poetscher L. MRI atlas of human white matter: Elsevier; 2005.
45. Alfaro-Almagro F, McCarthy P, Afyouni S, Andersson JLR, Bastiani M, Miller KL, et al. Confound modelling in UK Biobank brain imaging. Neuroimage. 2021;224:117002.
46. Dekkers IA, Jansen PR, Lamb HJ. Obesity, Brain Volume, and White Matter Microstructure at MRI: A Cross-sectional UK Biobank Study. Radiology. 2019;292(1):270.
47. Daley-Yates PT. Inhaled corticosteroids: potency, dose equivalence and therapeutic index. Br J Clin Pharmacol. 2015;80(3):372-80.
48. R Core Team. R: A language and environment for statistical computing. Vienna, Austria: R Foundation for Statistical Computing; 2020 [Available from: https://www.R-project.org/.
49. Wickham H AM, Bryan J, Chang W, McGowan LD, François R, Grolemund G, Hayes A, Henry L, Hester J, Kuhn M, Pedersen TL, Miller E, Bache SM, Müller K, Ooms J, Robinson D, Seidel DP, Spinu V, Takahashi K, Vaughan D, Wilke C, Woo K, Yutani H,. Welcome to the tidyverse. Journal of Open Source Software. 2019;4:1686.
50. Fox J, Weisberg S. An R Companion to Applied Regression. Third ed. Thousand Oaks CA: Sage; 2019.
51. Zeileis A, Hothorn T. Diagnostic Checking in Regression Relationships. R News. 2002;2(3):7-10.
52. Tingley D, Yamamoto T, Hirose K, Keele L, Imai K. mediation: R Package for Causal Mediation Analysis. Journal of Statistical Software. 2014;59(5):1-38.
53. Resmini E, Santos A, Gómez-Anson B, Vives Y, Pires P, Crespo I, et al. Verbal and visual memory performance and hippocampal volumes, measured by 3-Tesla magnetic resonance imaging, in patients with Cushing's syndrome. J Clin Endocrinol Metab. 2012;97(2):663-71.
54. Brown ES, Woolston DJ, Frol AB. Amygdala volume in patients receiving chronic corticosteroid therapy. Biol Psychiatry. 2008;63(7):705-9.
55. Bartsch T, Wulff P. The hippocampus in aging and disease: From plasticity to vulnerability. Neuroscience. 2015;309:1-16.
56. Pessoa L. Emotion and cognition and the amygdala: from "what is it?" to "what's to be done?". Neuropsychologia. 2010;48(12):3416-29.
57. Rolls ET. The cingulate cortex and limbic systems for emotion, action, and memory. Brain Struct Funct. 2019;224(9):3001-18.
58. Gasquoine PG. Contributions of the insula to cognition and emotion. Neuropsychol Rev. 2014;24(2):77-87.
59. Grahn JA, Parkinson JA, Owen AM. The role of the basal ganglia in learning and memory: neuropsychological studies. Behav Brain Res. 2009;199(1):53-60.
60. Schmahmann JD, Caplan D. Cognition, emotion and the cerebellum. Brain. 2006;129(2):290-2.
61. Savas M, Vinkers CH, Rosmalen JGM, Hartman CA, Wester VL, van den Akker ELT, et al. Systemic and Local Corticosteroid Use Is Associated with Reduced Executive Cognition, and Mood and Anxiety Disorders. Neuroendocrinology. 2020;110(3-4):282-91.

For peer review only

For peer review only - http://bmjopen.bmj.com/site/about/guidelines.xhtml

62. Alonso G. Prolonged corticosterone treatment of adult rats inhibits the proliferation of oligodendrocyte progenitors present throughout white and gray matter regions of the brain. Glia. 2000;31(3):219-31.
63. Miguel-Hidalgo JJ, Carter K, Deloach PH, Sanders L, Pang Y. Glucocorticoid-Induced Reductions of Myelination and Connexin 43 in Mixed Central Nervous System Cell Cultures Are Prevented by Mifepristone. Neuroscience. 2019;411:255-69.
64. Unemura K, Kume T, Kondo M, Maeda Y, Izumi Y, Akaike A. Glucocorticoids decrease astrocyte numbers by reducing glucocorticoid receptor expression in vitro and in vivo. J Pharmacol Sci. 2012;119(1):30-9.
65. Zhang H, Zhao Y, Wang Z. Chronic corticosterone exposure reduces hippocampal astrocyte structural plasticity and induces hippocampal atrophy in mice. Neurosci Lett. 2015;592:76-81.
66. van Olst L, Bielefeld P, Fitzsimons CP, de Vries HE, Schouten M. Glucocorticoid-mediated modulation of morphological changes associated with aging in microglia. Aging Cell. 2018;17(4):e12790.
67. Freitas AE, Egea J, Buendia I, Gómez-Rangel V, Parada E, Navarro E, et al. Agmatine, by Improving Neuroplasticity Markers and Inducing Nrf2, Prevents Corticosterone-Induced Depressive-Like Behavior in Mice. Mol Neurobiol. 2016;53(5):3030-45.
68. Machin AR, Babatunde O, Haththotuwa R, Scott I, Blagojevic-Bucknall M, Corp N, et al. The association between anxiety and disease activity and quality of life in rheumatoid arthritis: a systematic review and meta-analysis. Clin Rheumatol. 2020;39(5):1471-82.
69. Pelgrim CE, Peterson JD, Gosker HR, Schols A, van Helvoort A, Garssen J, et al. Psychological co-morbidities in COPD: Targeting systemic inflammation, a benefit for both? Eur J Pharmacol. 2019;842:99-110.
70. Meijer OC, Koorneef LL, Kroon J. Glucocorticoid receptor modulators. Ann Endocrinol (Paris). 2018;79(3):107-11.
71. Van Moortel L, Gevaert K, De Bosscher K. Improved Glucocorticoid Receptor Ligands: Fantastic Beasts, but How to Find Them? Front Endocrinol (Lausanne). 2020;11:559673.
72. Takao H, Hayashi N, Inano S, Ohtomo K. Effect of head size on diffusion tensor imaging. Neuroimage. 2011;57(3):958-67.
73. Takao H, Hayashi N, Ohtomo K. Sex dimorphism in the white matter: fractional anisotropy and brain size. J Magn Reson Imaging. 2014;39(4):917-23.

For peer review only

For peer review only - http://bmjopen.bmj.com/site/about/guidelines.xhtml

Tables and figures

Figure 1. Flowchart of participant inclusion

DWI, diffusion-weighted imaging; GC, glucocorticoids; n, number; T1, T1-weighted magnetic resonance imaging (MRI).

For peer review only

For peer review only - http://bmjopen.bmj.com/site/about/guidelines.xhtml

Figure 2. Bar plots showing the adjusted mean difference (with 95% confidence interval) of all imaging parameters for patients using oral glucocorticoids (GC) (n = 222) or inhalation GC (n = 557), and subgroups of chronic oral GC users (n = 42), or chronic inhalation GC users (n = 305) vs. controls (n = 24106). Volumes are in mm^3.

For peer review only

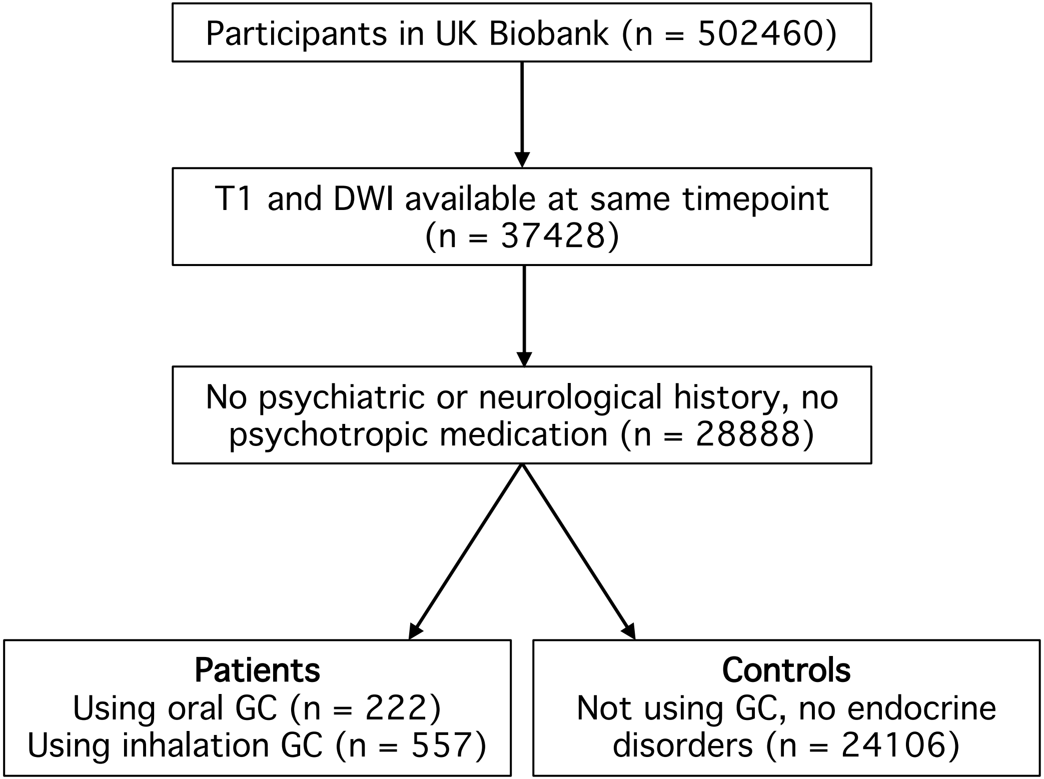
Participants in UK Biobank (n = 502460)
T1 and DWI available at same timepoint (n = 37428)
No psychiatric or neurological history, no psychotropic medication (n = 28888)
Patients
Using oral GC (n = 222)
Using inhalation GC (n = 557)
Controls
Not using GC, no endocrine disorders (n = 24106)

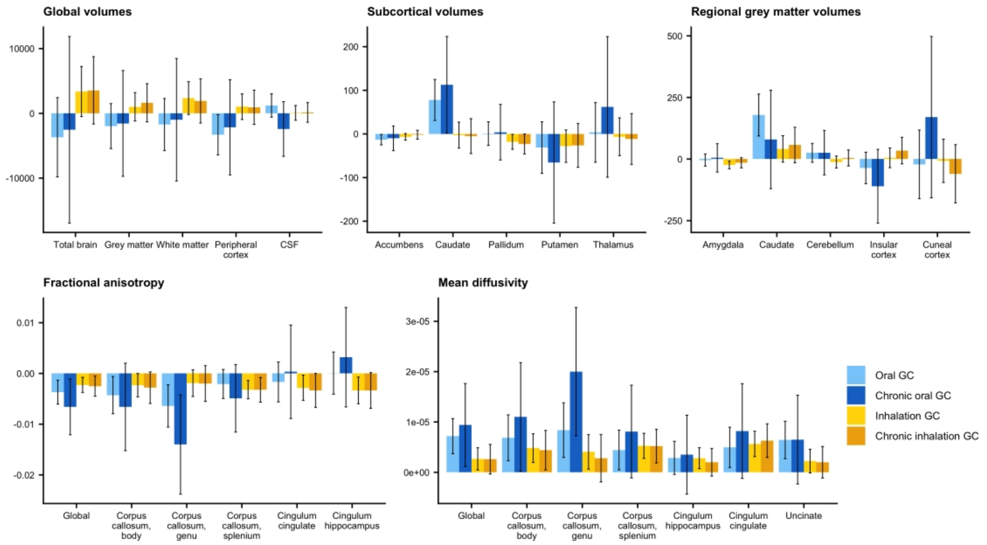

Bar plots showing the adjusted mean difference (with 95% confidence interval) of all imaging parameters for patients using oral glucocorticoids (GC) (n = 222) or inhalation GC (n = 557), and subgroups of chronic oral GC users (n = 42), or chronic inhalation GC users (n = 305) vs. controls (n = 24106). Volumes are in mm3.

1481x846mm (72 x 72 DPI)

Field ID	Code	Description
20003	1140874790	betamethasone
20003	1140874816	dexamethasone
20003	1140874954	hydrocortistab 20mg tablet
20003	1140874956	hydrocortone 10mg tablet
20003	1140874978	medrone 2mg tablet
20003	1140874976	methylprednisolone
20003	1140874950	prednesol 5mg tablet
20003	1140874930	prednisolone
20003	1141157402	prednisolone product
20003	1140868364	prednisone
20003	1140884704	cortisone product
20003	1140874896	hydrocortisone
20003	1140910424	hc - hydrocortisone
20003	1141157294	hydrocortisone product
20003	1141173346	cortisone
20003	1140874944	precortisyl 1mg tablet
20003	1140857532	cortelan 25mg tablet
20003	1140857534	oradexon 500micrograms tablet
20003	1140868370	decortisyl 5mg tablet
20003	1140868426	triamcinolone
20003	1140868434	ledercort 2mg tablet
20003	1140851210	cortenema 100mg/60ml enema
20003	1140874792	betnelan 500mcg tablet
20003	1140874794	betnesol 500mcg soluble tablet
20003	1140874810	cortistab 5mg tablet
20003	1140874814	cortisyl 25mg tablet
20003	1140874822	decadron 500micrograms tablet
20003	1140874936	deltacortril enteric 2.5mg e/c tablet
20003	1140874940	deltastab 1mg tablet
20003	1140910634	deltahydrocortisone
20003	1140910484	cortisol product

For peer review only

Field ID	Code	Description
20003	1140855466	bextasol 100micrograms inhaler
20003	1140881922	becodisks 100micrograms disks+diskhaler
20003	1140881938	beclomethasone dipropionate+salbutamol
20003	1141167594	qvar 50 inhaler
20003	1141174512	budesonide+eformoterol
20003	1141174520	symbicort 100/6 turbohaler
20003	1140862572	budesonide
20003	1140862574	pulmicort ls 50micrograms inhaler
20003	1140862584	pulmicort ls 50micrograms spacer inhaler
20003	1140862476	beclazone 50 inhaler
20003	1140862380	becloforte 250micrograms inhaler
20003	1140862474	aerobec 50mcg autohaler
20003	1140862382	becotide 50 inhaler
20003	1140888098	fluticasone
20003	1141164086	salmeterol+fluticasone propionate
20003	1141176832	seretide 50 evohaler
20003	1141176842	dexsol 2mg/5ml oral solution
20003	1140864286	flixotide 25micrograms inhaler
20003	1141191818	asmanex twisthaler 200mcg breath-actuated dry powder inhaler
20003	1140884654	beclomethasone
20003	1140909786	beclometasone
20003	1141179072	pulvinal beclomethasone diprop 100mcg breath-act dry pdr inh

For peer review only

Codes for the psychotropic medications used as exclusion criteria

Field ID	Code	Description
20003	1140921600	citalopram
20003	1141180212	escitalopram
20003	1141190158	cipralex 5mg tablet
20003	1141151946	cipramil 10mg tablet
20003	1140879540	fluoxetine
20003	1140867876	prozac 20mg capsule
20003	1140867878	sertraline
20003	1140879544	fluvoxamine
20003	1140867860	faverin 50mg tablet
20003	1140867888	paroxetine
20003	1140882236	seroxat 20mg tablet
20003	1140867884	lustral 50mg tablet
20003	1140916282	venlafaxine
20003	1141200564	duloxetine
20003	1141201834	cymbalta 30mg gastro-resistant capsule
20003	1140916288	efexor 37.5mg tablet
20003	1140917460	nefazodone
20003	1140917466	dutonin 100mg tablet
20003	1140879634	trazodone
20003	1141151978	reboxetine
20003	1141151982	edronax 4mg tablet
20003	1140879688	viloxazine
20003	1140867770	vivalan 50mg tablet
20003	1141199446	atomoxetine
20003	1141199460	strattera 10mg capsule
20003	1141176854	bupropion
20003	1140879616	amitriptyline
20003	1140867938	amitriptyline+chlordiazepoxide 12.5mg/5mg capsule
20003	1140867948	amitriptyline hydrochloride+perphenazine 10mg/2mg tablet
20003	1140867658	elavil 10mg tablet
20003	1140879620	clomipramine
20003	1140867690	anafranil 10mg capsule
20003	1140879624	desipramine
20003	1140909806	dosulepin
20003	1140867624	prothiaden 25mg capsule
20003	1141168396	doxepin hydrochloride 5% cream
20003	1140882312	sinequan 10mg capsule
20003	1140879630	imipramine
20003	1140867712	tofranil 10mg tablet
20003	1140867726	lofepramine
20003	1141146062	lomont 70mg/5ml s/f suspension
20003	1140882310	gamanil 70mg tablet

For peer review only

20003	1140867818	nortriptyline
20003	1140867940	fluphenazine hydrochloride+nortriptyline 1.5mg/30mg tablet
20003	1140867942	fluphenazine hcl+nortriptyline 500micrograms/10mg tablet
20003	1140867824	aventyl 10mg capsule
20003	1140879632	protriptyline
20003	1140867756	trimipramine
20003	1140867758	surmontil 10mg tablet
20003	1140867720	iprindole
20003	1140867722	prondol 15mg tablet
20003	1140867734	concordin 5mg tablet
20003	1140856074	butriptyline
20003	1140856076	evadyne 25mg tablet
20003	1140867774	amoxapine
20003	1140867840	asendis 25mg tablet
20003	1140879552	maprotiline
20003	1140867784	ludiomil 10mg tablet
20003	1140879556	mianserin
20003	1141152732	mirtazapine
20003	1140867856	isocarboxazid
20003	1140867858	marplan 10mg tablet
20003	1140910704	maoi - phenelzine
20003	140867850	phenelzine
20003	1140867852	nardil 15mg tablet
20003	1140867914	tranylcypromine
20003	1140867916	parnate 10mg tablet
20003	1140879668	selegiline
20003	1140872348	eldepryl 5mg tablet
20003	1141169666	zelapar 1.25mg tablet
20003	1140867920	moclobemide
20003	1140867922	manerix 150mg tablet
20003	1140867960	tryptophan product
20003	1140879674	pipothiazine
20003	1141153490	amisulpride
20003	1141184742	solian 100mg/ml s/f oral solution
20003	1141195974	aripiprazole
20003	1141202024	abilify 5mg tablet
20003	1140928916	olanzapine
20003	1141167976	zyprexa 2.5mg tablet
20003	1141152848	quetiapine
20003	1141152860	seroquel 25mg tablet
20003	1140867444	risperidone
20003	1141177762	risperdal 0.5mg tablet
20003	1140867078	benperidol
20003	1140867084	droperidol
20003	1140867080	anquil 250micrograms tablet
20003	1140867086	droleptan 10mg tablet

For peer review only

20003	1140867168	haloperidol
20003	1140867180	dozic 1mg/ml oral liquid
20003	1140867184	haldol 5mg tablet
20003	1140867092	serenace 500micrograms capsule
20003	1140867546	fluspirilene
20003	1140867548	redeptin 2mg/1ml injection
20003	1140867218	pimozide
20003	1140879658	chlorpromazine
20003	1140910358	cpz - chlorpromazine
20003	1140867398	fluphenazine decanoate
20003	1140867940	fluphenazine hydrochloride+nortriptyline 1.5mg/30mg tablet
20003	1140867942	fluphenazine hcl+nortriptyline 500micrograms/10mg tablet
20003	1140867944	tranylcypromine+trifluoperazine 10mg/1mg tablet
20003	1140867948	amitriptyline hydrochloride+perphenazine 10mg/2mg tablet
20003	1140909802	levomepromazine
20003	1140867122	nozinan 25mg tablet
20003	1140867134	pericyazine
20003	1140867136	neulactil 2.5mg tablet
20003	1140867208	perphenazine
20003	1140867210	fentazin 2mg tablet
20003	1140909804	pipotiazine
20003	1140867572	piportil depot 50mg/1ml oily injection
20003	1140868170	prochlorperazine
20003	1140868172	stemetil 5mg tablet
20003	1140879746	promazine
20003	1140867288	sparine 50mg/5ml suspension
20003	1140882082	promethazine product
20003	1140879750	thioridazine
20003	1140867312	melleril 10mg tablet
20003	1140867304	sulpiride
20003	1140868120	trifluoperazine
20003	1140867244	stelazine 1mg tablet
20003	1140856052	chlorprothixene
20003	1140882100	zuclopenthixol
20003	1140867150	flupenthixol
20003	1140867152	depixol 3mg tablet
20003	1140867156	moditen 1mg tablet
20003	1140867342	clopixol 2mg tablet
20003	1140867306	dolmatil 200mg tablet
20003	1141185130	sulpor 200mg/5ml oral solution
20003	1140867406	loxapine
20003	1140867414	loxapac 10mg capsule
20003	1140867420	clozapine
20003	1140879704	remoxipride
20003	1140867432	roxiam 150mg m/r capsule
20003	1140882320	clozaril 25mg tablet

For peer review only

20003	1141169714	zotepine
20003	1141169722	zoleptil 25mg tablet
20003	1140927956	sertindole
20003	1140927970	serdolect 4mg tablet
20003	1140855960	fortunan 500micrograms tablet
20003	1141200004	pregabalin
20003	1141200072	lyrica 25mg capsule
20003	1140883656	hydroxyzine
20003	1140863286	atarax 10mg tablet
20003	1140863292	ucerax 25mg tablet
20003	1140863302	lorazepam
20003	1140863308	alprazolam
20003	1140863310	xanax 250mcg tablet
20003	1140863318	bromazepam
20003	1140863320	lexotan 1.5mg tablet
20003	1140863364	ativan 1mg tablet
20003	1140863372	medazepam
20003	1140863374	nobrium 5mg capsule
20003	1140863268	clobazam
20003	1140863272	frisium 10mg capsule
20003	1140863274	potassium clorazepate
20003	1140863276	tranxene 7.5mg capsule
20003	1140863152	diazepam
20003	1140863164	rimapam 2mg tablet
20003	1140863170	diazemuls 10mg/2ml injection
20003	1140863172	dialar 2mg/5ml syrup
20003	1140863234	stesolid 5mg rectal solution
20003	1140863238	tensium 2mg tablet
20003	1140863244	valium 2mg tablet
20003	1140863250	valium 2mg/5ml syrup
20003	1140863442	oxazepam
20003	1140863454	buspar 5mg tablet
20003	1140879730	buspirone
20003	1140855944	prazepam
20003	1140855946	centrax 10mg tablet
20003	1140863328	chlordiazepoxide
20003	1140863350	librium 5mg tablet
20003	1140872150	clonazepam
20003	1140872152	rivotril 500mcg tablet
20003	1140856092	pacitron 500mg tablet
20003	1140856108	villescon tablet

For peer review only

Codes for the psychi

Field ID	Code
20002	1081
20002	1082
20002	1083
20002	1086
20002	1240
20002	1243
20002	1244
20002	1245
20002	1246
20002	1247
20002	1248
20002	1249
20002	1250
20002	1251
20002	1252
20002	1254
20002	1255
20002	1256
20002	1257
20002	1258
20002	1259
20002	1260
20002	1261
20002	1262
20002	1263
20002	1264
20002	1265
20002	1266
20002	1267
20002	1289
20002	1394
20002	1397
20002	1408
20002	1409
20002	1410
20002	1425
20002	1433
20002	1436
20002	1437
20002	1469
20002	1470
20002	1491

For peer review only

20002	1531
20002	1659
20001	1031
20001	1032
20001	1033
41270	C700
41270	C701
41270	C709
41270	C710
41270	C711
41270	C712
41270	C713
41270	C714
41270	C715
41270	C716
41270	C717
41270	C718
41270	C719
41270	C720
41270	C721
41270	C722
41270	C723
41270	C724
41270	C725
41270	C728
41270	C729
41270	F000
41270	F001
41270	F002
41270	F009
41270	F010
41270	F011
41270	F012
41270	F013
41270	F018
41270	F019
41270	F020
41270	F021
41270	F022
41270	F023
41270	F024
41270	F028
41270	F03
41270	F04
41270	F051
41270	F060

For peer review only

41270 F061
41270 F062
41270 F065
41270 F066
41270 F067
41270 F068
41270 F069
41270 F070
41270 F071
41270 F072
41270 F078
41270 F079
41270 F09
41270 F100
41270 F101
41270 F102
41270 F103
41270 F104
41270 F105
41270 F106
41270 F107
41270 F108
41270 F109
41270 F110
41270 F111
41270 F112
41270 F113
41270 F114
41270 F115
41270 F117
41270 F119
41270 F120
41270 F121
41270 F122
41270 F123
41270 F125
41270 F128
41270 F129
41270 F130
41270 F131
41270 F132
41270 F133
41270 F134
41270 F139
41270 F140
41270 F141

For peer review only

41270 F142
41270 F145
41270 F149
41270 F150
41270 F151
41270 F152
41270 F153
41270 F155
41270 F158
41270 F159
41270 F161
41270 F162
41270 F163
41270 F165
41270 F167
41270 F168
41270 F169
41270 F170
41270 F171
41270 F172
41270 F173
41270 F174
41270 F177
41270 F179
41270 F181
41270 F182
41270 F183
41270 F185
41270 F189
41270 F190
41270 F191
41270 F192
41270 F193
41270 F194
41270 F195
41270 F198
41270 F199
41270 F200
41270 F201
41270 F202
41270 F203
41270 F204
41270 F205
41270 F206
41270 F208
41270 F209

For peer review only

41270 F21
41270 F220
41270 F228
41270 F229
41270 F230
41270 F231
41270 F232
41270 F233
41270 F238
41270 F239
41270 F24
41270 F250
41270 F251
41270 F252
41270 F258
41270 F259
41270 F28
41270 F29
41270 F431
41270 F432
41270 F438
41270 F439
41270 F440
41270 F441
41270 F442
41270 F443
41270 F444
41270 F445
41270 F446
41270 F447
41270 F448
41270 F449
41270 F450
41270 F451
41270 F452
41270 F453
41270 F454
41270 F458
41270 F459
41270 F480
41270 F481
41270 F488
41270 F489
41270 F500
41270 F501
41270 F502

For peer review only

41270 F505
41270 F508
41270 F509
41270 F530
41270 F531
41270 F538
41270 F539
41270 F54
41270 F55
41270 F59
41270 F600
41270 F601
41270 F602
41270 F603
41270 F604
41270 F605
41270 F606
41270 F607
41270 F608
41270 F609
41270 F61
41270 F620
41270 F621
41270 F628
41270 F629
41270 F630
41270 F631
41270 F632
41270 F633
41270 F638
41270 F639
41270 F680
41270 F681
41270 F688
41270 F69
41270 F700
41270 F701
41270 F708
41270 F709
41270 F711
41270 F719
41270 F729
41270 F780
41270 F789
41270 F790
41270 F798

For peer review only

41270 F799
41270 F800
41270 F801
41270 F802
41270 F803
41270 F809
41270 F810
41270 F812
41270 F819
41270 F82
41270 F83
41270 F840
41270 F841
41270 F843
41270 F844
41270 F845
41270 F849
41270 F89
41270 F900
41270 F909
41270 F911
41270 F918
41270 F919
41270 F920
41270 F929
41270 F930
41270 F940
41270 F941
41270 F950
41270 F951
41270 F952
41270 F958
41270 F959
41270 F981
41270 F985
41270 F986
41270 F988
41270 F99
41270 G000
41270 G001
41270 G002
41270 G003
41270 G008
41270 G009
41270 G01
41270 G020

For peer review only

41270 G021
41270 G030
41270 G031
41270 G032
41270 G038
41270 G039
41270 G040
41270 G042
41270 G048
41270 G049
41270 G050
41270 G051
41270 G052
41270 G058
41270 G060
41270 G061
41270 G062
41270 G07
41270 G08
41270 G09
41270 G10
41270 G110
41270 G111
41270 G112
41270 G113
41270 G114
41270 G118
41270 G119
41270 G120
41270 G121
41270 G122
41270 G128
41270 G129
41270 G130
41270 G131
41270 G138
41270 G14
41270 G20
41270 G210
41270 G211
41270 G212
41270 G213
41270 G214
41270 G218
41270 G219
41270 G22

For peer review only

41270 G230
41270 G231
41270 G232
41270 G233
41270 G238
41270 G239
41270 G240
41270 G241
41270 G242
41270 G243
41270 G244
41270 G245
41270 G248
41270 G249
41270 G250
41270 G251
41270 G252
41270 G253
41270 G254
41270 G255
41270 G258
41270 G259
41270 G300
41270 G301
41270 G308
41270 G309
41270 G310
41270 G311
41270 G312
41270 G318
41270 G319
41270 G320
41270 G328
41270 G35
41270 G360
41270 G368
41270 G369
41270 G370
41270 G371
41270 G372
41270 G373
41270 G374
41270 G378
41270 G379
41270 G400
41270 G401

For peer review only

41270 G402
41270 G403
41270 G404
41270 G405
41270 G406
41270 G407
41270 G408
41270 G409
41270 G410
41270 G411
41270 G412
41270 G418
41270 G419
41270 G430
41270 G431
41270 G432
41270 G433
41270 G438
41270 G439
41270 G440
41270 G441
41270 G442
41270 G443
41270 G444
41270 G448
41270 G450
41270 G451
41270 G452
41270 G453
41270 G454
41270 G458
41270 G459
41270 G460
41270 G461
41270 G462
41270 G463
41270 G464
41270 G465
41270 G466
41270 G467
41270 G468
41270 G474
41270 G500
41270 G501
41270 G508
41270 G509

For peer review only

41270 G510
41270 G511
41270 G512
41270 G513
41270 G514
41270 G518
41270 G519
41270 G520
41270 G521
41270 G522
41270 G523
41270 G527
41270 G528
41270 G529
41270 G530
41270 G531
41270 G532
41270 G533
41270 G538
41270 G540
41270 G541
41270 G542
41270 G543
41270 G544
41270 G545
41270 G546
41270 G547
41270 G548
41270 G549
41270 G550
41270 G551
41270 G552
41270 G553
41270 G558
41270 G560
41270 G561
41270 G562
41270 G563
41270 G564
41270 G568
41270 G569
41270 G570
41270 G571
41270 G572
41270 G573
41270 G574

For peer review only

41270 G575
41270 G576
41270 G578
41270 G579
41270 G580
41270 G587
41270 G588
41270 G589
41270 G590
41270 G598
41270 G600
41270 G602
41270 G603
41270 G608
41270 G609
41270 G610
41270 G611
41270 G618
41270 G619
41270 G620
41270 G621
41270 G622
41270 G628
41270 G629
41270 G630
41270 G631
41270 G632
41270 G633
41270 G634
41270 G635
41270 G636
41270 G638
41270 G64
41270 G700
41270 G702
41270 G708
41270 G709
41270 G710
41270 G711
41270 G712
41270 G713
41270 G718
41270 G719
41270 G720
41270 G721
41270 G722

For peer review only

41270 G723
41270 G724
41270 G728
41270 G729
41270 G730
41270 G731
41270 G735
41270 G736
41270 G737
41270 G800
41270 G801
41270 G802
41270 G803
41270 G808
41270 G809
41270 G810
41270 G811
41270 G819
41270 G820
41270 G821
41270 G822
41270 G823
41270 G824
41270 G825
41270 G830
41270 G831
41270 G832
41270 G833
41270 G834
41270 G835
41270 G838
41270 G839
41270 G900
41270 G901
41270 G902
41270 G903
41270 G904
41270 G908
41270 G909
41270 G910
41270 G911
41270 G912
41270 G913
41270 G918
41270 G919
41270 G92

For peer review only

41270 G930
41270 G931
41270 G932
41270 G933
41270 G934
41270 G935
41270 G936
41270 G937
41270 G938
41270 G939
41270 G940
41270 G941
41270 G942
41270 G948
41270 G950
41270 G951
41270 G952
41270 G958
41270 G959
41270 G960
41270 G961
41270 G968
41270 G969
41270 G970
41270 G971
41270 G972
41270 G978
41270 G979
41270 G98
41270 G990
41270 G991
41270 G992
41270 G998
41270 S0200
41270 S0201
41270 S0210
41270 S0211
41270 S0270
41270 S0271
41270 S0280
41270 S9281
41270 S0290
41270 S0291
41270 S040
41270 S041
41270 S042

For peer review only

41270 S043
41270 S045
41270 S048
41270 S049
41270 S0600
41270 S0601
41270 S0610
41270 S0620
41270 S0621
41270 S0630
41270 S0631
41270 S0640
41270 S0641
41270 S0650
41270 S0651
41270 S0660
41270 S0661
41270 S0670
41270 S0680
41270 S0681
41270 S0690
41270 S0691
41270 S097
41270 S099
41270 T901
41270 T902
41270 T903
41270 T904
41270 T908
41270 T909
41270 T913

For peer review only

atric and neurological conditions used as exclusion criteria

Description
stroke
transient ischaemic attack (tia)
subdural haemorrhage/haematoma
subarachnoid haemorrhage
neurological injury/trauma
psychological/psychiatric problem
infection of nervous system
brain abscess/intracranial abscess
encephalitis
meningitis
spinal abscess
cranial nerve problem/palsy
bell's palsy/facial nerve palsy
spinal cord disorder
paraplegia
peripheral nerve disorder
peripheral neuropathy
acute infective polyneuritis/guillain-barre syndrome
trapped nerve/compressed nerve
chronic/degenerative neurological problem
motor neuron disease
myasthenia gravis
multiple sclerosis
Parkinson’s disease
dementia/Alzheimer’s/cognitive impairment
epilepsy
migraine
head injury
spinal injury
schizophrenia
peripheral nerve injury
other demyelinating disease (not multiple sclerosis)
alcohol dependency
opioid dependency
other substance abuse/dependency
cerebral aneurysm
cerebral palsy
headaches (not migraine)
myasthenia gravis
post-traumatic stress disorder
anorexia/bulimia/other eating disorder
brain haemorrhage

For peer review only

post-natal depression
meningioma / benign meningeal tumour
meningeal cancer / malignant meningioma
brain cancer / primary malignant brain tumour
spinal cord or cranial nerve cancer
C70.0 Cerebral meninges
C70.1 Spinal meninges
C70.9 Meninges, unspecified
C71.0 Cerebrum, except lobes and ventricles
C71.1 Frontal lobe
C71.2 Temporal lobe
C71.3 Parietal lobe
C71.4 Occipital lobe
C71.5 Cerebral ventricle
C71.6 Cerebellum
C71.7 Brain stem
C71.8 Overlapping lesion of brain
C71.9 Brain, unspecified
C72.0 Spinal cord
C72.1 Cauda equina
C72.2 Olfactory nerve
C72.3 Optic nerve
C72.4 Acoustic nerve
C72.5 Other and unspecified cranial nerves
C72.8 Overlapping lesion of brain and other parts of central nervous system
C72.9 Central nervous system, unspecified
F00.0 Dementia in Alzheimer's disease with early onset
F00.1 Dementia in Alzheimer's disease with late onset
F00.2 Dementia in Alzheimer's disease, atypical or mixed type
F00.9 Dementia in Alzheimer's disease, unspecified
F01.0 Vascular dementia of acute onset
F01.1 Multi-infarct dementia
F01.2 Subcortical vascular dementia
F01.3 Mixed cortical and subcortical vascular dementia
F01.8 Other vascular dementia
F01.9 Vascular dementia, unspecified
F02.0 Dementia in Pick's disease
F02.1 Dementia in Creutzfeldt-Jakob disease
F02.2 Dementia in Huntington's disease
F02.3 Dementia in Parkinson's disease
F02.4 Dementia in human immunodeficiency virus [HIV] disease
F02.8 Dementia in other specified diseases classified elsewhere
F03 Unspecified dementia
F04 Organic amnesic syndrome, not induced by alcohol and other psychoactive substances
F05.1 Delirium superimposed on dementia
F06.0 Organic hallucinosis

For peer review only

F06.1 Organic catatonic disorder
F06.2 Organic delusional [schizophrenia-like] disorder
F06.5 Organic dissociative disorder
F06.6 Organic emotionally labile [asthenic] disorder
F06.7 Mild cognitive disorder
F06.8 Other specified mental disorders due to brain damage and dysfunction and to physical disease
F06.9 Unspecified mental disorder due to brain damage and dysfunction and to physical disease
F07.0 Organic personality disorder
F07.1 Postencephalitic syndrome
F07.2 Postconcussional syndrome
F07.8 Other organic personality and behavioural disorders due to brain disease, damage and dysfunct
F07.9 Unspecified organic personality and behavioural disorder due to brain disease, damage and dys
F09 Unspecified organic or symptomatic mental disorder
F10.0 Acute intoxication
F10.1 Harmful use
F10.2 Dependence syndrome
F10.3 Withdrawal state
F10.4 Withdrawal state with delirium
F10.5 Psychotic disorder
F10.6 Amnesic syndrome
F10.7 Residual and late-onset psychotic disorder
F10.8 Other mental and behavioural disorders
F10.9 Unspecified mental and behavioural disorder
F11.0 Acute intoxication
F11.1 Harmful use
F11.2 Dependence syndrome
F11.3 Withdrawal state
F11.4 Withdrawal state with delirium
F11.5 Psychotic disorder
F11.7 Residual and late-onset psychotic disorder
F11.9 Unspecified mental and behavioural disorder
F12.0 Acute intoxication
F12.1 Harmful use
F12.2 Dependence syndrome
F12.3 Withdrawal state
F12.5 Psychotic disorder
F12.8 Other mental and behavioural disorders
F12.9 Unspecified mental and behavioural disorder
F13.0 Acute intoxication
F13.1 Harmful use
F13.2 Dependence syndrome
F13.3 Withdrawal state
F13.4 Withdrawal state with delirium
F13.9 Unspecified mental and behavioural disorder
F14.0 Acute intoxication
F14.1 Harmful use

For peer review only

F14.2 Dependence syndrome
F14.5 Psychotic disorder
F14.9 Unspecified mental and behavioural disorder
F15.0 Acute intoxication
F15.1 Harmful use
F15.2 Dependence syndrome
F15.3 Withdrawal state
F15.5 Psychotic disorder
F15.8 Other mental and behavioural disorders
F15.9 Unspecified mental and behavioural disorder
F16.1 Harmful use
F16.2 Dependence syndrome
F16.3 Withdrawal state
F16.5 Psychotic disorder
F16.7 Residual and late-onset psychotic disorder
F16.8 Other mental and behavioural disorders
F16.9 Unspecified mental and behavioural disorder
F17.0 Acute intoxication
F17.1 Harmful use
F17.2 Dependence syndrome
F17.3 Withdrawal state
F17.4 Withdrawal state with delirium
F17.7 Residual and late-onset psychotic disorder
F17.9 Unspecified mental and behavioural disorder
F18.1 Harmful use
F18.2 Dependence syndrome
F18.3 Withdrawal state
F18.5 Psychotic disorder
F18.9 Unspecified mental and behavioural disorder
F19.0 Acute intoxication
F19.1 Harmful use
F19.2 Dependence syndrome
F19.3 Withdrawal state
F19.4 Withdrawal state with delirium
F19.5 Psychotic disorder
F19.8 Other mental and behavioural disorders
F19.9 Unspecified mental and behavioural disorder
F20.0 Paranoid schizophrenia
F20.1 Hebephrenic schizophrenia
F20.2 Catatonic schizophrenia
F20.3 Undifferentiated schizophrenia
F20.4 Postschizophrenic depression
F20.5 Residual schizophrenia
F20.6 Simple schizophrenia
F20.8 Other schizophrenia
F20.9 Schizophrenia, unspecified

For peer review only

F21 Schizotypal disorder
F22.0 Delusional disorder
F22.8 Other persistent delusional disorders
F22.9 Persistent delusional disorder, unspecified
F23.0 Acute polymorphic psychotic disorder without symptoms of schizophrenia
F23.1 Acute polymorphic psychotic disorder with symptoms of schizophrenia
F23.2 Acute schizophrenia-like psychotic disorder
F23.3 Other acute predominantly delusional psychotic disorders
F23.8 Other acute and transient psychotic disorders
F23.9 Acute and transient psychotic disorder, unspecified
F24 Induced delusional disorder
F25.0 Schizoaffective disorder, manic type
F25.1 Schizoaffective disorder, depressive type
F25.2 Schizoaffective disorder, mixed type
F25.8 Other schizoaffective disorders
F25.9 Schizoaffective disorder, unspecified
F28 Other nonorganic psychotic disorders
F29 Unspecified nonorganic psychosis
F43.1 Posttraumatic stress disorder
F43.2 Adjustment disorders
F43.8 Other reactions to severe stress
F43.9 Reaction to severe stress, unspecified
F44.0 Dissociative amnesia
F44.1 Dissociative fugue
F44.2 Dissociative stupor
F44.3 Trance and possession disorders
F44.4 Dissociative motor disorders
F44.5 Dissociative convulsions
F44.6 Dissociative anaesthesia and sensory loss
F44.7 Mixed dissociative [conversion] disorders
F44.8 Other dissociative [conversion] disorders
F44.9 Dissociative [conversion] disorder, unspecified
F45.0 Somatisation disorder
F45.1 Undifferentiated somatoform disorder
F45.2 Hypochondriacal disorder
F45.3 Somatoform autonomic dysfunction
F45.4 Persistent somatoform pain disorder
F45.8 Other somatoform disorders
F45.9 Somatoform disorder, unspecified
F48.0 Neurasthenia
F48.1 Depersonalisation-derealisation syndrome
F48.8 Other specified neurotic disorders
F48.9 Neurotic disorder, unspecified
F50.0 Anorexia nervosa
F50.1 Atypical anorexia nervosa
F50.2 Bulimia nervosa

For peer review only

F50.5 Vomiting associated with other psychological disturbances
F50.8 Other eating disorders
F50.9 Eating disorder, unspecified
F53.0 Mild mental and behavioural disorders associated with the puerperium, not elsewhere classifie
F53.1 Severe mental and behavioural disorders associated with the puerperium, not elsewhere classif
F53.8 Other mental and behavioural disorders associated with the puerperium, not elsewhere classifi
F53.9 Puerperal mental disorder, unspecified
F54 Psychological and behavioural factors associated with disorders or diseases classified elsewhere
F55 Abuse of non-dependence-producing substances
F59 Unspecified behavioural syndromes associated with physiological disturbances and physical facto
F60.0 Paranoid personality disorder
F60.1 Schizoid personality disorder
F60.2 Dissocial personality disorder
F60.3 Emotionally unstable personality disorder
F60.4 Histrionic personality disorder
F60.5 Anankastic personality disorder
F60.6 Anxious [avoidant] personality disorder
F60.7 Dependent personality disorder
F60.8 Other specific personality disorders
F60.9 Personality disorder, unspecified
F61 Mixed and other personality disorders
F62.0 Enduring personality change after catastrophic experience
F62.1 Enduring personality change after psychiatric illness
F62.8 Other enduring personality changes
F62.9 Enduring personality change, unspecified
F63.0 Pathological gambling
F63.1 Pathological fire-setting [pyromania]
F63.2 Pathological stealing [kleptomania]
F63.3 Trichotillomania
F63.8 Other habit and impulse disorders
F63.9 Habit and impulse disorder, unspecified
F68.0 Elaboration of physical symptoms for psychological reasons
F68.1 Intentional production or feigning of symptoms or disabilities, either physical or psychological [
F68.8 Other specified disorders of adult personality and behaviour
F69 Unspecified disorder of adult personality and behaviour
F70.0 Mild mental retardation (With the statement of no, or minimal, impairment of behaviour)
F70.1 Mild mental retardation (Significant impairment of behaviour requiring attention or treatment)
F70.8 Mild mental retardation (Other impairments of behaviour)
F70.9 Mild mental retardation (Without mention of impairment of behaviour)
F71.1 Moderate mental retardation (Significant impairment of behaviour requiring attention or treatr
F71.9 Moderate mental retardation (Without mention of impairment of behaviour)
F72.9 Severe mental retardation (Without mention of impairment of behaviour)
F78.0 Other mental retardation (With the statement of no, or minimal, impairment of behaviour)
F78.9 Other mental retardation (Without mention of impairment of behaviour)
F79.0 Unspecified mental retardation (With the statement of no, or minimal, impairment of behaviou
F79.8 Unspecified mental retardation (Other impairments of behaviour)

For peer review only

F79.9 Unspecified mental retardation (Without mention of impairment of behaviour)
F80.0 Specific speech articulation disorder
F80.1 Expressive language disorder
F80.2 Receptive language disorder
F80.3 Acquired aphasia with epilepsy [Landau-Kleffner]
F80.9 Developmental disorder of speech and language, unspecified
F81.0 Specific reading disorder
F81.2 Specific disorder of arithmetical skills
F81.9 Developmental disorder of scholastic skills, unspecified
F82 Specific developmental disorder of motor function
F83 Mixed specific developmental disorders
F84.0 Childhood autism
F84.1 Atypical autism
F84.3 Other childhood disintegrative disorder
F84.4 Overactive disorder associated with mental retardation and stereotyped movements
F84.5 Asperger's syndrome
F84.9 Pervasive developmental disorder, unspecified
F89 Unspecified disorder of psychological development
F90.0 Disturbance of activity and attention
F90.9 Hyperkinetic disorder, unspecified
F91.1 Unsocialised conduct disorder
F91.8 Other conduct disorders
F91.9 Conduct disorder, unspecified
F92.0 Depressive conduct disorder
F92.9 Mixed disorder of conduct and emotions, unspecified
F93.0 Separation anxiety disorder of childhood
F94.0 Elective mutism
F94.1 Reactive attachment disorder of childhood
F95.0 Transient tic disorder
F95.1 Chronic motor or vocal tic disorder
F95.2 Combined vocal and multiple motor tic disorder [de la Tourette]
F95.8 Other tic disorders
F95.9 Tic disorder, unspecified
F98.1 Nonorganic encopresis
F98.5 Stuttering [stammering]
F98.6 Cluttering
F98.8 Other specified behavioural and emotional disorders with onset usually occurring in childhood [illegible]
F99 Mental disorder, not otherwise specified
G00.0 Haemophilus meningitis
G00.1 Pneumococcal meningitis
G00.2 Streptococcal meningitis
G00.3 Staphylococcal meningitis
G00.8 Other bacterial meningitis
G00.9 Bacterial meningitis, unspecified
G01 Meningitis in bacterial diseases classified elsewhere
G02.0 Meningitis in viral diseases classified elsewhere

For peer review only

G02.1 Meningitis in mycoses
G03.0 Nonpyogenic meningitis
G03.1 Chronic meningitis
G03.2 Benign recurrent meningitis [Mollaret]
G03.8 Meningitis due to other specified causes
G03.9 Meningitis, unspecified
G04.0 Acute disseminated encephalitis
G04.2 Bacterial meningoencephalitis and meningomyelitis, not elsewhere classified
G04.8 Other encephalitis, myelitis and encephalomyelitis
G04.9 Encephalitis, myelitis and encephalomyelitis, unspecified
G05.0 Encephalitis, myelitis and encephalomyelitis in bacterial diseases classified elsewhere
G05.1 Encephalitis, myelitis and encephalomyelitis in viral diseases classified elsewhere
G05.2 Encephalitis, myelitis and encephalomyelitis in other infectious and parasitic diseases classified
G05.8 Encephalitis, myelitis and encephalomyelitis in other diseases classified elsewhere
G06.0 Intracranial abscess and granuloma
G06.1 Intraspinal abscess and granuloma
G06.2 Extradural and subdural abscess, unspecified
G07 Intracranial and intraspinal abscess and granuloma in diseases classified elsewhere
G08 Intracranial and intraspinal phlebitis and thrombophlebitis
G09 Sequelae of inflammatory diseases of central nervous system
G10 Huntington's disease
G11.0 Congenital nonprogressive ataxia
G11.1 Early-onset cerebellar ataxia
G11.2 Late-onset cerebellar ataxia
G11.3 Cerebellar ataxia with defective DNA repair
G11.4 Hereditary spastic paraplegia
G11.8 Other hereditary ataxias
G11.9 Hereditary ataxia, unspecified
G12.0 Infantile spinal muscular atrophy, type I [Werdnig-Hoffman]
G12.1 Other inherited spinal muscular atrophy
G12.2 Motor neuron disease
G12.8 Other spinal muscular atrophies and related syndromes
G12.9 Spinal muscular atrophy, unspecified
G13.0 Paraneoplastic neuromyopathy and neuropathy
G13.1 Other systemic atrophy primarily affecting central nervous system in neoplastic disease
G13.8 Systemic atrophy primarily affecting central nervous system in other diseases classified elsewh
G14 Postpolio syndrome
G20 Parkinson's disease
G21.0 Malignant neuroleptic syndrome
G21.1 Other drug-induced secondary Parkinsonism
G21.2 Secondary Parkinsonism due to other external agents
G21.3 Postencephalitic Parkinsonism
G21.4 Vascular parkinsonism
G21.8 Other secondary Parkinsonism
G21.9 Secondary Parkinsonism, unspecified
G22 Parkinsonism in diseases classified elsewhere

For peer review only

G23.0 Hallervorden-Spatz disease
G23.1 Progressive supranuclear ophthalmoplegia [Steele-Richardson-Olszewski]
G23.2 Striatonigral degeneration
G23.3 Multiple system atrophy, cerebellar type
G23.8 Other specified degenerative diseases of basal ganglia
G23.9 Degenerative disease of basal ganglia, unspecified
G24.0 Drug-induced dystonia
G24.1 Idiopathic familial dystonia
G24.2 Idiopathic nonfamilial dystonia
G24.3 Spasmodic torticollis
G24.4 Idiopathic orofacial dystonia
G24.5 Blepharospasm
G24.8 Other dystonia
G24.9 Dystonia, unspecified
G25.0 Essential tremor
G25.1 Drug-induced tremor
G25.2 Other specified forms of tremor
G25.3 Myoclonus
G25.4 Drug-induced chorea
G25.5 Other chorea
G25.8 Other specified extrapyramidal and movement disorders
G25.9 Extrapyramidal and movement disorder, unspecified
G30.0 Alzheimer's disease with early onset
G30.1 Alzheimer's disease with late onset
G30.8 Other Alzheimer's disease
G30.9 Alzheimer's disease, unspecified
G31.0 Circumscribed brain atrophy
G31.1 Senile degeneration of brain, not elsewhere classified
G31.2 Degeneration of nervous system due to alcohol
G31.8 Other specified degenerative diseases of nervous system
G31.9 Degenerative disease of nervous system, unspecified
G32.0 Subacute combined degeneration of spinal cord in diseases classified elsewhere
G32.8 Other specified degenerative disorders of nervous system in diseases classified elsewhere
G35 Multiple sclerosis
G36.0 Neuromyelitis optica [Devic]
G36.8 Other specified acute disseminated demyelination
G36.9 Acute disseminated demyelination, unspecified
G37.0 Diffuse sclerosis
G37.1 Central demyelination of corpus callosum
G37.2 Central pontine myelinolysis
G37.3 Acute transverse myelitis in demyelinating disease of central nervous system
G37.4 Subacute necrotising myelitis
G37.8 Other specified demyelinating diseases of central nervous system
G37.9 Demyelinating disease of central nervous system, unspecified
G40.0 Localisation-related (focal) (partial) idiopathic epilepsy and epileptic syndromes with seizures o
G40.1 Localisation-related (focal) (partial) symptomatic epilepsy and epileptic syndromes with simple

For peer review only

G40.2 Localisation-related (focal) (partial) symptomatic epilepsy and epileptic syndromes with comple
G40.3 Generalised idiopathic epilepsy and epileptic syndromes
G40.4 Other generalised epilepsy and epileptic syndromes
G40.5 Special epileptic syndromes
G40.6 Grand mal seizures, unspecified (with or without petit mal)
G40.7 Petit mal, unspecified, without grand mal seizures
G40.8 Other epilepsy
G40.9 Epilepsy, unspecified
G41.0 Grand mal status epilepticus
G41.1 Petit mal status epilepticus
G41.2 Complex partial status epilepticus
G41.8 Other status epilepticus
G41.9 Status epilepticus, unspecified
G43.0 Migraine without aura [common migraine]
G43.1 Migraine with aura [classical migraine]
G43.2 Status migrainosus
G43.3 Complicated migraine
G43.8 Other migraine
G43.9 Migraine, unspecified
G44.0 Cluster headache syndrome
G44.1 Vascular headache, not elsewhere classified
G44.2 Tension-type headache
G44.3 Chronic posttraumatic headache
G44.4 Drug-induced headache, not elsewhere classified
G44.8 Other specified headache syndromes
G45.0 Vertebro-basilar artery syndrome
G45.1 Carotid artery syndrome (hemispheric)
G45.2 Multiple and bilateral precerebral artery syndromes
G45.3 Amaurosis fugax
G45.4 Transient global amnesia
G45.8 Other transient cerebral ischaemic attacks and related syndromes
G45.9 Transient cerebral ischaemic attack, unspecified
G46.0 Middle cerebral artery syndrome
G46.1 Anterior cerebral artery syndrome
G46.2 Posterior cerebral artery syndrome
G46.3 Brain stem stroke syndrome
G46.4 Cerebellar stroke syndrome
G46.5 Pure motor lacunar syndrome
G46.6 Pure sensory lacunar syndrome
G46.7 Other lacunar syndromes
G46.8 Other vascular syndromes of brain in cerebrovascular diseases
G47.4 Narcolepsy and cataplexy
G50.0 Trigeminal neuralgia
G50.1 Atypical facial pain
G50.8 Other disorders of trigeminal nerve
G50.9 Disorder of trigeminal nerve, unspecified

For peer review only

G51.0 Bell's palsy
G51.1 Geniculate ganglionitis
G51.2 Melkersson's syndrome
G51.3 Clonic hemifacial spasm
G51.4 Facial myokymia
G51.8 Other disorders of facial nerve
G51.9 Disorder of facial nerve, unspecified
G52.0 Disorders of olfactory nerve
G52.1 Disorders of glossopharyngeal nerve
G52.2 Disorders of vagus nerve
G52.3 Disorders of hypoglossal nerve
G52.7 Disorders of multiple cranial nerves
G52.8 Disorders of other specified cranial nerves
G52.9 Cranial nerve disorder, unspecified
G53.0 Postzoster neuralgia
G53.1 Multiple cranial nerve palsies in infectious and parasitic diseases classified elsewhere
G53.2 Multiple cranial nerve palsies in sarcoidosis
G53.3 Multiple cranial nerve palsies in neoplastic disease
G53.8 Other cranial nerve disorders in other diseases classified elsewhere
G54.0 Brachial plexus disorders
G54.1 Lumbosacral plexus disorders
G54.2 Cervical root disorders, not elsewhere classified
G54.3 Thoracic root disorders, not elsewhere classified
G54.4 Lumbosacral root disorders, not elsewhere classified
G54.5 Neuralgic amyotrophy
G54.6 Phantom limb syndrome with pain
G54.7 Phantom limb syndrome without pain
G54.8 Other nerve root and plexus disorders
G54.9 Nerve root and plexus disorder, unspecified
G55.0 Nerve root and plexus compressions in neoplastic disease
G55.1 Nerve root and plexus compressions in intervertebral disk disorders
G55.2 Nerve root and plexus compressions in spondylosis
G55.3 Nerve root and plexus compressions in other dorsopathies
G55.8 Nerve root and plexus compressions in other diseases classified elsewhere
G56.0 Carpal tunnel syndrome
G56.1 Other lesions of median nerve
G56.2 Lesion of ulnar nerve
G56.3 Lesion of radial nerve
G56.4 Causalgia
G56.8 Other mononeuropathies of upper limb
G56.9 Mononeuropathy of upper limb, unspecified
G57.0 Lesion of sciatic nerve
G57.1 Meralgia paraesthetica
G57.2 Lesion of femoral nerve
G57.3 Lesion of lateral popliteal nerve
G57.4 Lesion of medial popliteal nerve

For peer review only

G57.5 Tarsal tunnel syndrome
G57.6 Lesion of plantar nerve
G57.8 Other mononeuropathies of lower limb
G57.9 Mononeuropathy of lower limb, unspecified
G58.0 Intercostal neuropathy
G58.7 Mononeuritis multiplex
G58.8 Other specified mononeuropathies
G58.9 Mononeuropathy, unspecified
G59.0 Diabetic mononeuropathy
G59.8 Other mononeuropathies in diseases classified elsewhere
G60.0 Hereditary motor and sensory neuropathy
G60.2 Neuropathy in association with hereditary ataxia
G60.3 Idiopathic progressive neuropathy
G60.8 Other hereditary and idiopathic neuropathies
G60.9 Hereditary and idiopathic neuropathy, unspecified
G61.0 Guillain-Barre syndrome
G61.1 Serum neuropathy1
G61.8 Other inflammatory polyneuropathies
G61.9 Inflammatory polyneuropathy, unspecified
G62.0 Drug-induced polyneuropathy
G62.1 Alcoholic polyneuropathy
G62.2 Polyneuropathy due to other toxic agents
G62.8 Other specified polyneuropathies
G62.9 Polyneuropathy, unspecified
G63.0 Polyneuropathy in infectious and parasitic diseases classified elsewhere
G63.1 Polyneuropathy in neoplastic disease
G63.2 Diabetic polyneuropathy
G63.3 Polyneuropathy in other endocrine and metabolic diseases
G63.4 Polyneuropathy in nutritional deficiency
G63.5 Polyneuropathy in systemic connective tissue disorders
G63.6 Polyneuropathy in other musculoskeletal disorders
G63.8 Polyneuropathy in other diseases classified elsewhere
G64 Other disorders of peripheral nervous system
G70.0 Myasthenia gravis
G70.2 Congenital and developmental myasthenia
G70.8 Other specified myoneural disorders
G70.9 Myoneural disorder, unspecified
G71.0 Muscular dystrophy
G71.1 Myotonic disorders
G71.2 Congenital myopathies
G71.3 Mitochondrial myopathy, not elsewhere classified
G71.8 Other primary disorders of muscles
G71.9 Primary disorder of muscle, unspecified
G72.0 Drug-induced myopathy
G72.1 Alcoholic myopathy
G72.2 Myopathy due to other toxic agents

For peer review only

G72.3 Periodic paralysis
G72.4 Inflammatory myopathy, not elsewhere classified
G72.8 Other specified myopathies
G72.9 Myopathy, unspecified
G73.0 Myasthenic syndromes in endocrine diseases
G73.1 Eaton-Lambert syndrome
G73.5 Myopathy in endocrine diseases
G73.6 Myopathy in metabolic diseases
G73.7 Myopathy other diseases classified elsewhere
G80.0 Spastic cerebral palsy
G80.1 Spastic diplegia
G80.2 Infantile hemiplegia
G80.3 Dyskinetic cerebral palsy
G80.8 Other infantile cerebral palsy
G80.9 Infantile cerebral palsy, unspecified
G81.0 Flaccid hemiplegia
G81.1 Spastic hemiplegia
G81.9 Hemiplegia, unspecified
G82.0 Flaccid paraplegia
G82.1 Spastic paraplegia
G82.2 Paraplegia, unspecified
G82.3 Flaccid tetraplegia
G82.4 Spastic tetraplegia
G82.5 Tetraplegia, unspecified
G83.0 Diplegia of upper limbs
G83.1 Monoplegia of lower limb
G83.2 Monoplegia of upper limb
G83.3 Monoplegia, unspecified
G83.4 Cauda equina syndrome
G83.5 Locked-in syndrome
G83.8 Other specified paralytic syndromes
G83.9 Paralytic syndrome, unspecified
G90.0 Idiopathic peripheral autonomic neuropathy
G90.1 Familial dysautonomia [Riley-Day]
G90.2 Horner's syndrome
G90.3 Multisystem degeneration
G90.4 Autonomic dysreflexia
G90.8 Other disorders of autonomic nervous system
G90.9 Disorder of autonomic nervous system, unspecified
G91.0 Communicating hydrocephalus
G91.1 Obstructive hydrocephalus
G91.2 Normal-pressure hydrocephalus
G91.3 Posttraumatic hydrocephalus, unspecified
G91.8 Other hydrocephalus
G91.9 Hydrocephalus, unspecified
G92 Toxic encephalopathy

For peer review only

G93.0 Cerebral cysts
G93.1 Anoxic brain damage, not elsewhere classified
G93.2 Benign intracranial hypertension
G93.3 Postviral fatigue syndrome
G93.4 Encephalopathy, unspecified
G93.5 Compression of brain
G93.6 Cerebral oedema
G93.7 Reye's syndrome
G93.8 Other specified disorders of brain
G93.9 Disorder of brain, unspecified
G94.0 Hydrocephalus in infectious and parasitic diseases classified elsewhere
G94.1 Hydrocephalus in neoplastic disease
G94.2 Hydrocephalus in other diseases classified elsewhere
G94.8 Other specified disorders of brain in diseases classified elsewhere
G95.0 Syringomyelia and syringobulbia
G95.1 Vascular myelopathies
G95.2 Cord compression, unspecified
G95.8 Other specified diseases of spinal cord
G95.9 Disease of spinal cord, unspecified
G96.0 Cerebrospinal fluid leak
G96.1 Disorders of meninges, not elsewhere classified
G96.8 Other specified disorders of central nervous system
G96.9 Disorder of central nervous system, unspecified
G97.0 Cerebrospinal fluid leak from spinal puncture
G97.1 Other reaction to spinal and lumbar puncture
G97.2 Intracranial hypotension following ventricular shunting
G97.8 Other postprocedural disorders of nervous system
G97.9 Postprocedural disorder of nervous system, unspecified
G98 Other disorders of nervous system, not elsewhere classified
G99.0 Autonomic neuropathy in endocrine and metabolic diseases
G99.1 Other disorders of autonomic nervous system in other diseases classified elsewhere
G99.2 Myelopathy in diseases classified elsewhere
G99.8 Other specified disorders of nervous system in diseases classified elsewhere
S02.00 Fracture of vault of skull (closed)
S02.01 Fracture of vault of skull (open)
S02.10 Fracture of base of skull (closed)
S02.11 Fracture of base of skull (open)
S02.70 Multiple fractures involving skull and facial bones (closed)
S02.71 Multiple fractures involving skull and facial bones (open)
S02.80 Fractures of other skull and facial bones (closed)
S02.81 Fractures of other skull and facial bones (open)
S02.90 Fracture of skull and facial bones, part unspecified (closed)
S02.91 Fracture of skull and facial bones, part unspecified (open)
S04.0 Injury of optic nerve and pathways
S04.1 Injury of oculomotor nerve
S04.2 Injury of trochlear nerve

For peer review only

S04.3 Injury of trigeminal nerve
S04.5 Injury of facial nerve
S04.8 Injury of other cranial nerves
S04.9 Injury of unspecified cranial nerve
S06.00 Concussion (without open intracranial wound)
S06.01 Concussion (with open intracranial wound)
S06.10 Traumatic cerebral oedema (without open intracranial wound)
S06.20 Diffuse brain injury (without open intracranial wound)
S06.21 Diffuse brain injury (with open intracranial wound)
S06.30 Focal brain injury (without open intracranial wound)
S06.31 Focal brain injury (with open intracranial wound)
S06.40 Epidural haemorrhage (without open intracranial wound)
S06.41 Epidural haemorrhage (with open intracranial wound)
S06.50 Traumatic subdural haemorrhage (without open intracranial wound)
S06.51 Traumatic subdural haemorrhage (with open intracranial wound)
S06.60 Traumatic subarachnoid haemorrhage (without open intracranial wound)
S06.61 Traumatic subarachnoid haemorrhage (with open intracranial wound)
S06.70 Intracranial injury with prolonged coma (without open intracranial wound)
S06.80 Other intracranial injuries (without open intracranial wound)
S06.81 Other intracranial injuries (with open intracranial wound)
S06.90 Intracranial injury, unspecified (without open intracranial wound)
S06.91 Intracranial injury, unspecified (with open intracranial wound)
S09.7 Multiple injuries of head
S09.9 Unspecified injury of head
T90.1 Sequelae of open wound of head
T90.2 Sequelae of fracture of skull and facial bones
T90.3 Sequelae of injury of cranial nerves
T90.5 Sequelae of intracranial injury
T90.8 Sequelae of other specified injuries of head
T90.9 Sequelae of unspecified injury of head
T91.3 Sequelae of injury of spinal cord

For peer review only

Codes for the endoc

Field ID	Code
20002	1214
20002	1220
20002	1221
20002	1222
20002	1223
20002	1224
20002	1225
20002	1226
20002	1228
20002	1229
20002	1230
20002	1232
20002	1233
20002	1234
20002	1235
20002	1236
20002	1237
20002	1238
20002	1239
20002	1350
20002	1428
20002	1429
20002	1430
20002	1431
20002	1432
20002	1521
20002	1522
20002	1610
20002	1611
20002	1682
41270	C73
41270	C740
41270	C741
41270	C749
41270	C750
41270	C751
41270	C752
41270	C753
41270	C754
41270	C755
41270	C758
41270	C759

For peer review only

41270 E011
41270 E018
41270 E02
41270 E030
41270 E031
41270 E032
41270 E033
41270 E034
41270 E035
41270 E038
41270 E039
41270 E040
41270 E041
41270 E042
41270 E048
41270 E049
41270 E050
41270 E051
41270 E052
41270 E053
41270 E054
41270 E055
41270 E058
41270 E059
41270 E060
41270 E061
41270 E062
41270 E063
41270 E064
41270 E065
41270 E069
41270 E070
41270 E071
41270 E078
41270 E079
41270 E100
41270 E101
41270 E102
41270 E103
41270 E104
41270 E105
41270 E106
41270 E107
41270 E108
41270 E109
41270 E110

For peer review only

41270 E111
41270 E112
41270 E113
41270 E114
41270 E115
41270 E116
41270 E117
41270 E118
41270 E119
41270 E121
41270 E123
41270 E125
41270 E128
41270 E129
41270 E130
41270 E131
41270 E132
41270 E133
41270 E134
41270 E135
41270 E136
41270 E137
41270 E138
41270 E139
41270 E140
41270 E141
41270 E142
41270 E143
41270 E144
41270 E145
41270 E146
41270 E147
41270 E148
41270 E149
41270 E15
41270 E160
41270 E161
41270 E162
41270 E163
41270 E164
41270 E168
41270 E169
41270 E200
41270 E201
41270 E208
41270 E209

For peer review only

41270 E210
41270 E211
41270 E212
41270 E213
41270 E214
41270 E215
41270 E220
41270 E221
41270 E222
41270 E228
41270 E229
41270 E230
41270 E231
41270 E232
41270 E233
41270 E236
41270 E237
41270 E240
41270 E241
41270 E242
41270 E243
41270 E244
41270 E248
41270 E249
41270 E250
41270 E258
41270 E259
41270 E270
41270 E271
41270 E272
41270 E273
41270 E274
41270 E275
41270 E278
41270 E279
41270 E281
41270 E282
41270 E283
41270 E288
41270 E289
41270 E290
41270 E291
41270 E298
41270 E299
41270 E351
41270 E340

For peer review only

rine conditions used as exclusion criteria for the control group

Description
testicular problems (not cancer)
diabetes
gestational diabetes
type 1 diabetes
type 2 diabetes
thyroid problem (not cancer)
hyperthyroidism/thyrotoxicosis
hypothyroidism/myxoedema
thyroid radioablation therapy
parathyroid gland problem (not cancer)
parathyroid hyperplasia/adenoma
disorder of adrenal gland
adrenal tumour
adrenocortical insufficiency/addison's disease
hyperaldosteronism/conn's syndrome
phaeochromocytoma
disorder or pituitary gland
pituitary adenoma/tumour
cushings syndrome
polycystic ovaries/polycystic ovarian syndrome
thyroiditis
acromegaly
hypopituitarism
hyperprolactinaemia
carcinoid syndrome/tumour
diabetes insipidus
grave's disease
thyroid goitre
hyperparathyroidism
benign insulinoma
C73 Malignant neoplasm of thyroid gland
C74.0 Cortex of adrenal gland
C74.1 Medulla of adrenal gland
C74.9 Adrenal gland, unspecified
C75.0 Parathyroid gland
C75.1 Pituitary gland
C75.2 Craniopharyngeal duct
C75.3 Pineal gland
C75.4 Carotid body
C75.5 Aortic body and other paraganglia
C75.8 Pluriglandular involvement, unspecified
C75.9 Endocrine gland, unspecified

For peer review only

E01.1 Iodine-deficiency-related multinodular (endemic) goitre
E01.8 Other iodine-deficiency-related thyroid disorders and allied conditions
E02 Subclinical iodine-deficiency hypothyroidism
E03.0 Congenital hypothyroidism with diffuse goitre
E03.1 Congenital hypothyroidism without goitre
E03.2 Hypothyroidism due to medicaments and other exogenous substances
E03.3 Postinfectious hypothyroidism
E03.4 Atrophy of thyroid (acquired)
E03.5 Myxoedema coma
E03.8 Other specified hypothyroidism
E03.9 Hypothyroidism, unspecified
E04.0 Non-toxic diffuse goitre
E04.1 Non-toxic single thyroid nodule
E04.2 Non-toxic multinodular goitre
E04.8 Other specified non-toxic goitre
E04.9 Non-toxic goitre, unspecified
E05.0 Thyrotoxicosis with diffuse goitre
E05.1 Thyrotoxicosis with toxic single thyroid nodule
E05.2 Thyrotoxicosis with toxic multinodular goitre
E05.3 Thyrotoxicosis from ectopic thyroid tissue
E05.4 Thyrotoxicosis factitia
E05.5 Thyroid crisis or storm
E05.8 Other thyrotoxicosis
E05.9 Thyrotoxicosis, unspecified
E06.0 Acute thyroiditis
E06.1 Subacute thyroiditis
E06.2 Chronic thyroiditis with transient thyrotoxicosis
E06.3 Autoimmune thyroiditis
E06.4 Drug-induced thyroiditis
E06.5 Other chronic thyroiditis
E06.9 Thyroiditis, unspecified
E07.0 Hypersecretion of calcitonin
E07.1 Dyshormogenetic goitre
E07.8 Other specified disorders of thyroid
E07.9 Disorder of thyroid, unspecified
E10.0 With coma
E10.1 With ketoacidosis
E10.2 With renal complications
E10.3 With ophthalmic complications
E10.4 With neurological complications
E10.5 With peripheral circulatory complications
E10.6 With other specified complications
E10.7 With multiple complications
E10.8 With unspecified complications
E10.9 Without complications
E11.0 With coma

For peer review only

E11.1 With ketoacidosis
E11.2 With renal complications
E11.3 With ophthalmic complications
E11.4 With neurological complications
E11.5 With peripheral circulatory complications
E11.6 With other specified complications
E11.7 With multiple complications
E11.8 With unspecified complications
E11.9 Without complications
E12.1 With ketoacidosis
E12.3 With ophthalmic complications
E12.5 With peripheral circulatory complications
E12.8 With unspecified complications
E12.9 Without complications
E13.0 With coma
E13.1 With ketoacidosis
E13.2 With renal complications
E13.3 With ophthalmic complications
E13.4 With neurological complications
E13.5 With peripheral circulatory complications
E13.6 With other specified complications
E13.7 With multiple complications
E13.8 With unspecified complications
E13.9 Without complications
E14.0 With coma
E14.1 With ketoacidosis
E14.2 Withrenal complications
E14.3 With ophthalmic complications
E14.4 With neurological complications
E14.5 With peripheral circulatory complications
E14.6 With other specified complications
E14.7 With multiple complications
E14.8 With unspecified complications
E14.9 Without complications
E15 Nondiabetic hypoglycaemic coma
E16.0 Drug-induced hypoglycaemia without coma
E16.1 Other hypoglycaemia
E16.2 Hypoglycaemia, unspecified
E16.3 Increased secretion of glucagon
E16.4 Abnormal secretion of gastrin
E16.8 Other specified disorders of pancreatic internal secretion
E16.9 Disorder of pancreatic internal secretion, unspecified
E20.0 Idiopathic hypoparathyroidism
E20.1 Pseudohypoparathyroidism
E20.8 Other hypoparathyroidism
E20.9 Hypoparathyroidism, unspecified

For peer review only

E21.0 Primary hyperparathyroidism
E21.1 Secondary hyperparathyroidism, not elsewhere classified
E21.2 Other hyperparathyroidism
E21.3 Hyperparathyroidism, unspecified
E21.4 Other specified disorders of parathyroid gland
E21.5 Disorder of parathyroid gland, unspecified
E22.0 Acromegaly and pituitary gigantism
E22.1 Hyperprolactinaemia
E22.2 Syndrome of inappropriate secretion of antidiuretic hormone
E22.8 Other hyperfunction of pituitary gland
E22.9 Hyperfunction of pituitary gland, unspecified
E23.0 Hypopituitarism
E23.1 Drug-induced hypopituitarism
E23.2 Diabetes insipidus
E23.3 Hypothalamic dysfunction, not elsewhere classified
E23.6 Other disorders of pituitary gland
E23.7 Disorder of pituitary gland, unspecified
E24.0 Pituitary-dependent Cushing's disease
E24.1 Nelson's syndrome
E24.2 Drug-induced Cushing's syndrome
E24.3 Ectopic ACTH syndrome
E24.4 Alcohol-induced pseudo-Cushing's syndrome
E24.8 Other Cushing's syndrome
E24.9 Cushing's syndrome, unspecified
E25.0 Congenital adrenogenital disorders associated with enzyme deficiency
E25.8 Other adrenogenital disorders
E25.9 Adrenogenital disorder, unspecified
E27.0 Other adrenocortical overactivity
E27.1 Primary adrenocortical insufficiency
E27.2 Addisonian crisis
E27.3 Drug-induced adrenocortical insufficiency
E27.4 Other and unspecified adrenocortical insufficiency
E27.5 Adrenomedullary hyperfunction
E27.8 Other specified disorders of adrenal gland
E27.9 Disorder of adrenal gland, unspecified
E28.1 Androgen excess
E28.2 Polycystic ovarian syndrome
E28.3 Primary ovarian failure
E28.8 Other ovarian dysfunction
E28.9 Ovarian dysfunction, unspecified
E29.0 Testicular hyperfunction
E29.1 Testicular hypofunction
E29.8 Other testicular dysfunction
E29.9 Testicular dysfunction, unspecified
E35.1 Disorders of adrenal glands in diseases classified elsewhere
E34.0 Carcinoid syndrome

For peer review only

Field ID	Description
25010	Volume of brain, grey+white matter
25008	Volume of white matter
25006	Volume of grey matter
25002	Volume of peripheral cortical grey matter
25004	Volume of ventricular cerebrospinal fluid
25888	Volume of grey matter in Amygdala (left)
25889	Volume of grey matter in Amygdala (right)
25880	Volume of grey matter in Caudate (left)
25881	Volume of grey matter in Caudate (right)
25838	Volume of grey matter in Cingulate Gyrus, anterior division (left)
25839	Volume of grey matter in Cingulate Gyrus, anterior division (right)
25840	Volume of grey matter in Cingulate Gyrus, posterior division (left)
25841	Volume of grey matter in Cingulate Gyrus, posterior division (right)
25886	Volume of grey matter in Hippocampus (left)
25887	Volume of grey matter in Hippocampus (right)
25784	Volume of grey matter in Insular Cortex (left)
25785	Volume of grey matter in Insular Cortex (right)
25788	Volume of grey matter in Middle Frontal Gyrus (left)
25789	Volume of grey matter in Middle Frontal Gyrus (right)
25844	Volume of grey matter in Cuneal Cortex (left)
25845	Volume of grey matter in Cuneal Cortex (right)
25842	Volume of grey matter in Precuneous Cortex (left)
25843	Volume of grey matter in Precuneous Cortex (right)
25900	Volume of grey matter in Crus I Cerebellum (left)
25902	Volume of grey matter in Crus I Cerebellum (right)
25901	Volume of grey matter in Crus I Cerebellum (vermis)
25903	Volume of grey matter in Crus II Cerebellum (left)
25905	Volume of grey matter in Crus II Cerebellum (right)
25904	Volume of grey matter in Crus II Cerebellum (vermis)
25893	Volume of grey matter in I-IV Cerebellum (left)
25894	Volume of grey matter in I-IV Cerebellum (right)
25915	Volume of grey matter in IX Cerebellum (left)
25917	Volume of grey matter in IX Cerebellum (right)
25916	Volume of grey matter in IX Cerebellum (vermis)
25895	Volume of grey matter in V Cerebellum (left)
25896	Volume of grey matter in V Cerebellum (right)
25897	Volume of grey matter in VI Cerebellum (left)
25899	Volume of grey matter in VI Cerebellum (right)
25898	Volume of grey matter in VI Cerebellum (vermis)
25909	Volume of grey matter in VIIIa Cerebellum (left)
25911	Volume of grey matter in VIIIa Cerebellum (right)
25910	Volume of grey matter in VIIIa Cerebellum (vermis)
25912	Volume of grey matter in VIIIb Cerebellum (left)
25914	Volume of grey matter in VIIIb Cerebellum (right)
25913	Volume of grey matter in VIIIb Cerebellum (vermis)

For peer review only

25906	Volume of grey matter in VIIb Cerebellum (left)
25908	Volume of grey matter in VIIb Cerebellum (right)
25907	Volume of grey matter in VIIb Cerebellum (vermis)
25890	Volume of grey matter in Ventral Striatum (left)
25891	Volume of grey matter in Ventral Striatum (right)
25918	Volume of grey matter in X Cerebellum (left)
25919	Volume of grey matter in X Cerebellum (vermis)
25920	Volume of grey matter in X Cerebellum (right)
25023	Volume of accumbens (left)
25024	Volume of accumbens (right)
25021	Volume of amygdala (left)
25022	Volume of amygdala (right)
25013	Volume of caudate (left)
25014	Volume of caudate (right)
25019	Volume of hippocampus (left)
25020	Volume of hippocampus (right)
25017	Volume of pallidum (left)
25018	Volume of pallidum (right)
25015	Volume of putamen (left)
25016	Volume of putamen (right)
25011	Volume of thalamus (left)
25012	Volume of thalamus (right)
25059	Mean FA in body of corpus callosum on FA skeleton
25091	Mean FA in cingulum cingulate gyrus on FA skeleton (left)
25090	Mean FA in cingulum cingulate gyrus on FA skeleton (right)
25093	Mean FA in cingulum hippocampus on FA skeleton (left)
25092	Mean FA in cingulum hippocampus on FA skeleton (right)
25058	Mean FA in genu of corpus callosum on FA skeleton
25060	Mean FA in splenium of corpus callosum on FA skeleton
25101	Mean FA in uncinate fasciculus on FA skeleton (left)
25100	Mean FA in uncinate fasciculus on FA skeleton (right)
25107	Mean MD in body of corpus callosum on FA skeleton
25139	Mean MD in cingulum cingulate gyrus on FA skeleton (left)
25138	Mean MD in cingulum cingulate gyrus on FA skeleton (right)
25141	Mean MD in cingulum hippocampus on FA skeleton (left)
25140	Mean MD in cingulum hippocampus on FA skeleton (right)
25106	Mean MD in genu of corpus callosum on FA skeleton
25108	Mean MD in splenium of corpus callosum on FA skeleton
25149	Mean MD in uncinate fasciculus on FA skeleton (left)
25148	Mean MD in uncinate fasciculus on FA skeleton (right)

For the calculation of the global FA and global MD values, all available mean FA and mean MD were

**Field ID**	**Description**
25079	Mean FA in anterior corona radiata on FA skeleton (left)

For peer review only

25078 Mean FA in anterior corona radiata on FA skeleton (right)
25073 Mean FA in anterior limb of internal capsule on FA skeleton (left)
25072 Mean FA in anterior limb of internal capsule on FA skeleton (right)
25059 Mean FA in body of corpus callosum on FA skeleton
25071 Mean FA in cerebral peduncle on FA skeleton (left)
25070 Mean FA in cerebral peduncle on FA skeleton (right)
25091 Mean FA in cingulum cingulate gyrus on FA skeleton (left)
25090 Mean FA in cingulum cingulate gyrus on FA skeleton (right)
25093 Mean FA in cingulum hippocampus on FA skeleton (left)
25092 Mean FA in cingulum hippocampus on FA skeleton (right)
25063 Mean FA in corticospinal tract on FA skeleton (left)
25062 Mean FA in corticospinal tract on FA skeleton (right)
25089 Mean FA in external capsule on FA skeleton (left)
25088 Mean FA in external capsule on FA skeleton (right)
25095 Mean FA in fornix cres+stria terminalis on FA skeleton (left)
25094 Mean FA in fornix cres+stria terminalis on FA skeleton (right)
25061 Mean FA in fornix on FA skeleton
25058 Mean FA in genu of corpus callosum on FA skeleton
25067 Mean FA in inferior cerebellar peduncle on FA skeleton (left)
25066 Mean FA in inferior cerebellar peduncle on FA skeleton (right)
25065 Mean FA in medial lemniscus on FA skeleton (left)
25064 Mean FA in medial lemniscus on FA skeleton (right)
25056 Mean FA in middle cerebellar peduncle on FA skeleton
25057 Mean FA in pontine crossing tract on FA skeleton
25083 Mean FA in posterior corona radiata on FA skeleton (left)
25082 Mean FA in posterior corona radiata on FA skeleton (right)
25075 Mean FA in posterior limb of internal capsule on FA skeleton (left)
25074 Mean FA in posterior limb of internal capsule on FA skeleton (right)
25085 Mean FA in posterior thalamic radiation on FA skeleton (left)
25084 Mean FA in posterior thalamic radiation on FA skeleton (right)
25077 Mean FA in retrolenticular part of internal capsule on FA skeleton (left)
25076 Mean FA in retrolenticular part of internal capsule on FA skeleton (right)
25087 Mean FA in sagittal stratum on FA skeleton (left)
25086 Mean FA in sagittal stratum on FA skeleton (right)
25060 Mean FA in splenium of corpus callosum on FA skeleton
25069 Mean FA in superior cerebellar peduncle on FA skeleton (left)
25068 Mean FA in superior cerebellar peduncle on FA skeleton (right)
25081 Mean FA in superior corona radiata on FA skeleton (left)
25080 Mean FA in superior corona radiata on FA skeleton (right)
25099 Mean FA in superior fronto-occipital fasciculus on FA skeleton (left)
25098 Mean FA in superior fronto-occipital fasciculus on FA skeleton (right)
25097 Mean FA in superior longitudinal fasciculus on FA skeleton (left)
25096 Mean FA in superior longitudinal fasciculus on FA skeleton (right)
25103 Mean FA in tapetum on FA skeleton (left)
25102 Mean FA in tapetum on FA skeleton (right)
25101 Mean FA in uncinate fasciculus on FA skeleton (left)

For peer review only

25100 Mean FA in uncinate fasciculus on FA skeleton (right)
25127 Mean MD in anterior corona radiata on FA skeleton (left)
25126 Mean MD in anterior corona radiata on FA skeleton (right)
25121 Mean MD in anterior limb of internal capsule on FA skeleton (left)
25120 Mean MD in anterior limb of internal capsule on FA skeleton (right)
25107 Mean MD in body of corpus callosum on FA skeleton
25119 Mean MD in cerebral peduncle on FA skeleton (left)
25118 Mean MD in cerebral peduncle on FA skeleton (right)
25139 Mean MD in cingulum cingulate gyrus on FA skeleton (left)
25138 Mean MD in cingulum cingulate gyrus on FA skeleton (right)
25141 Mean MD in cingulum hippocampus on FA skeleton (left)
25140 Mean MD in cingulum hippocampus on FA skeleton (right)
25111 Mean MD in corticospinal tract on FA skeleton (left)
25110 Mean MD in corticospinal tract on FA skeleton (right)
25137 Mean MD in external capsule on FA skeleton (left)
25136 Mean MD in external capsule on FA skeleton (right)
25143 Mean MD in fornix cres+stria terminalis on FA skeleton (left)
25142 Mean MD in fornix cres+stria terminalis on FA skeleton (right)
25109 Mean MD in fornix on FA skeleton
25106 Mean MD in genu of corpus callosum on FA skeleton
25115 Mean MD in inferior cerebellar peduncle on FA skeleton (left)
25114 Mean MD in inferior cerebellar peduncle on FA skeleton (right)
25113 Mean MD in medial lemniscus on FA skeleton (left)
25112 Mean MD in medial lemniscus on FA skeleton (right)
25104 Mean MD in middle cerebellar peduncle on FA skeleton
25105 Mean MD in pontine crossing tract on FA skeleton
25131 Mean MD in posterior corona radiata on FA skeleton (left)
25130 Mean MD in posterior corona radiata on FA skeleton (right)
25123 Mean MD in posterior limb of internal capsule on FA skeleton (left)
25122 Mean MD in posterior limb of internal capsule on FA skeleton (right)
25133 Mean MD in posterior thalamic radiation on FA skeleton (left)
25132 Mean MD in posterior thalamic radiation on FA skeleton (right)
25125 Mean MD in retrolenticular part of internal capsule on FA skeleton (left)
25124 Mean MD in retrolenticular part of internal capsule on FA skeleton (right)
25135 Mean MD in sagittal stratum on FA skeleton (left)
25134 Mean MD in sagittal stratum on FA skeleton (right)
25108 Mean MD in splenium of corpus callosum on FA skeleton
25117 Mean MD in superior cerebellar peduncle on FA skeleton (left)
25116 Mean MD in superior cerebellar peduncle on FA skeleton (right)
25129 Mean MD in superior corona radiata on FA skeleton (left)
25128 Mean MD in superior corona radiata on FA skeleton (right)
25147 Mean MD in superior fronto-occipital fasciculus on FA skeleton (left)
25146 Mean MD in superior fronto-occipital fasciculus on FA skeleton (right)
25145 Mean MD in superior longitudinal fasciculus on FA skeleton (left)
25144 Mean MD in superior longitudinal fasciculus on FA skeleton (right)
25151 Mean MD in tapetum on FA skeleton (left)

For peer review only

25150 Mean MD in tapetum on FA skeleton (right)
25149 Mean MD in uncinate fasciculus on FA skeleton (left)
25148 Mean MD in uncinate fasciculus on FA skeleton (right)

For peer review only

For peer review only

For peer review only

averaged, respectively:

Field ID	Description
20023	Mean time to correctly identify matches (Reaction time)
20016	Fluid intelligence score (Fluid intelligence)
6348	Duration to complete numeric path (trail #1) (Trail making A)
6350	Duration to complete alphanumeric path (trail #2) (Trail making B)
23324	Number of symbol digit matches made correctly (Symbol digit substitution)
4282	Maximum digits remembered correctly (Digit span)

For peer review only

Field ID	Description
2050	Frequency of depressed mood in last 2 weeks
2060	Frequency of unenthusiasm / disinterest in last 2 weeks
2070	Frequency of tenseness / restlessness in last 2 weeks
2080	Frequency of tiredness / lethargy in last 2 weeks

For peer review only

Field ID	Description
31	Sex
21003	Age when attended assessment center
6138	Qualifications (education)
20116	Smoking status
53	Year of imaging
54	Assessment center
25756	Scanner lateral (X) brain position
25757	Scanner transverse (Y) brain position
25758	Scanner longitudinal (Z) brain position
25759	Scanner table position
25000	Volumetric scaling from T1 head image to standard space (measure of head size)
23104	Body mass index measured by body impedance
23099	Total body fat percentage measured by body impedance

For peer review only

Supplement 2. Structural or diffusion MRI studies in patients with Cushing disease or exogenous glucocorticoid use

	Cohort	Imaging modality	Findings
**Cushing**			
Andela 2013 [1]	25 CD patients with long-term remission, 25 healthy controls	Volumetric MRI	Patients had reduced GMV in ACC and increased GMV in left posterior lobe of cerebellum. Patients reported more psychological and cognitive symptoms than controls, but these were not associated with GMV changes.
Bauduin 2020 [2]	25 CD patients with long-term remission, 25 healthy controls	Volumetric MRI	Patients had reduced cortical thickness in left caudal ACC, right rostral ACC, left cuneus, left PCC, and bilateral precuneus. Cortical thickness in left caudal ACC and left cuneus were inversely associated with anxiety, depressive symptoms, and disease duration.
Bourdeau 2002 [3]	38 patients with active CS (21 with CD 17 with adrenal CS), 18 patients with other non-ACTH-secreting sellar tumors, 20 normal controls	Volumetric MRI	Overall loss of brain volume and increased ventricle diameters in CS patients. Re-imaging in 22 CS patients at 40 months after correction of hypercortisolism showed a decrease in ventricle diameters and increase in brain volume compared to active disease.
Burkhardt 2015 [4]	19 patients with active untreated CD, 40 healthy controls	Volumetric MRI	CD patients had reduced GMV in hippocampus and cerebellum compared to controls.
Chen 2020 [5]	101 patients with active untreated CD, and 95 patients with NFA (controls)	Volumetric MRI	CD patients had more cortical and subcortical atrophy, more white matter hyperintensities, and decreased hippocampal height. Follow-up of 14 CD patients showed partial reversion of brain atrophy and white matter hyperintensities after correction of hypercortisolism.
Crespo 2014 [6]	35 patients with CS (27 cured, 8 medically treated), 35 controls	Volumetric MRI	No differences were found between cured and treated CS patients. Patients had decreased cortical thickness in the left superior frontal cortex, precentral cortex, left insular cortex, left and right rostral ACC, and right caudal middle frontal cortex compared to controls. Patients also had altered decision-making strategies.
Hou [7]	50 patients with active CD, 36 healthy controls	Volumetric MRI	Patients had reductions in total GMV and frontal, parietal, occipital, and temporal lobes; insula; cingulate lobe; and enlargement of lateral and third ventricles. All affected brain regions improved significantly after TSS. No differences in volume of hippocampus or amygdala.
Jiang 2017 [8]	34 patients with CD (14 with short-term remission, 20 with active CD), 34 controls	Volumetric MRI	Remitted CD patients had greater GMV in bilateral caudate; no differences in GMV of MFG or cerebellum compared to controls. Active CD patients had smaller GMV in MFG and cerebellum compared to controls and remitted patients.
Jiang 2017 [9]	15 patients with active CD, 15 healthy controls	DKI	White matter: increased MD in the splenium of the corpus callosum, bilateral frontal lobe, and left temporal lobe. AD was mainly increased in the bilateral

For peer review only

			frontal lobe, and RD mainly in the left temporal lobe. FA was mainly decreased in the splenium of the corpus callosum and the left temporal lobe.<br>Gray matter: increased MD, RD, and AD in the left hippocampus/parahippocampal gyrus and the left temporal lobe, increased radial kurtosis in the right cerebellar hemisphere, decreased axial kurtosis in the left frontal lobe and decreased mean kurtosis in left cerebellar hemisphere.
Merke 2005 [10]	11 pediatric patients with active CS, 10 healthy controls	Volumetric MRI	CD patients had smaller cerebral volumes, larger ventricles, and a smaller amygdala. One year after surgical cure, cerebral atrophy was reversed, but children showed a decline in IQ and school performance.
Momose 1971 [11]	31 patients with active CD, 64 patients with acromegaly, 36 patients with chromophobe adenoma	Pneumoencephalography	Cerebral cortical atrophy was present in 90% and cerebellar cortical atrophy in 74% of patients with CD. In controls with acromegaly, this was 30% and 3%, respectively. In controls with a chromophobe adenoma, this was 58% and 20%, respectively.
Pires 2015 [12]	35 patients with CS (8 active hypercortisolism, 7 medication-remitted cortisol, 20 surgically cured), 35 healthy controls	DTI	Patients had widespread alteration in white matter integrity (increased FA, decreased MD, RD, AD) compared to controls. Both active and cured CS patients showed increased FA, and decreased MD, RD, and AD; medically treated CS patients did not have significantly different AD values.
Pires 2017 [13]	35 patients with CS (8 active hypercortisolism, 7 medication-remitted cortisol, 20 surgically cured), 35 healthy controls	DTI	All patient groups had more depression and anxiety than controls. Depression scores correlated negatively to FA (in right corticospinal tract (CST), forceps major, forceps minor, left inferior fronto-occipital fasciculus (IFOF) (frontal part), right IFOF, right inferior longitudinal fasciculus (ILF), left superior longitudinal fasciculus (SLF) (anterior part) and right SLF), and positively to RD values (in frontal regions of the forceps minor and frontal areas of bilateral IFOFs). Although processing speed did not differ between groups, Symbol Digit Modalities Test scores correlated positively to both FA and AD values.
Resmini 2012 [14]	33 patients with CS (11 active, 22 cured), 34 controls	Volumetric MRI	Patients had reduced total and cortical brain gray matter volumes compared with controls. Subcortical gray matter (which includes hippocampal volume) was reduced only in 12 patients with severe memory impairment. No differences in hippocampal volume were reported between patients with active or cured CS.
Santos 2014 [15]	36 patients with CS (15 active, 21 in remission), 36 controls	Volumetric MRI	Patients with active CS had smaller cerebellar cortex volumes, and patients with remitted CS showed a similar trend. Cerebellar white matter volume showed no differences.
Santos 2015 [16]	38 patients with CS (15 active, 23 in remission), 38 controls	Volumetric MRI	Patients in remission had more white matter lesions than controls and active patients. Both CS groups had reduced total brain volume and GMV. No differences were found in white matter volume.

For peer review only

Santos 2017 [17]	39 patients with CS (16 active, 23 in remission), 39 healthy controls	Volumetric MRI	Active CS patients had smaller right amygdala volumes. Left amygdala volume was associated with depression and anxiety scores. No differences were found between patients in remission and controls.
Simmons 2000 [18]	63 patients with CD (all after surgical treatment), 63 controls with sellar pathology other than ACTH-secreting tumors	Volumetric MRI	CD patients had higher degrees of cerebral atrophy than controls.
Starkman 1992 [19]	12 patients with CS	Volumetric MRI	For 27% of patients, hippocampal volume fell outside the 95% confidence interval of the population. Plasma cortisol was negatively correlated with hippocampal volume.
Starkman 1999 [20]	22 patients with active CD	Volumetric MRI	Sixteen months after TSS, hippocampus volume increased up to 10%, and a smaller increase was observed in caudate volume.
Starkman 2003 [21]	24 patients with active CD	Volumetric MRI	Sixteen months after TSS, all patients showed an increase in hippocampal volume (which was significantly correlated with lower cortisol levels, and with one neuropsychological test), and 18 patients had an increase in caudate head volume.
Tirosh 2020 [22]	29 patients with CS (8 active, 21 recovering), 8 controls	Volumetric MRI	Patients with persistent disease had increased white matter volume and decreased cortex thickness and white matter intensity compared with patients achieving remission of CS, mainly in frontal and parietal lobes (but not FDR-corrected). Compared to healthy controls, patients recovering from CS had a decrease in subcortical GM volume, an increase in cortical thickness, and a decrease in white matter volume in multiple sites (including accumbens).<br>In all patients together, 24h UFC correlated negatively with intensity in caudate, hippocampus, accumbens, and corpus callosum; correlated negatively with white matter intensity in frontal and parietal lobes; and positively with lateral ventricles volumes. Changes in 24h UFC correlated negatively with change in total brain volume, supratentorium, cerebellar cortex, and putamen.
Toffanin 2011 [23]	10 patients with active CD	Volumetric MRI	After TSS, the volume of the hippocampal head increased significantly, but no change in hippocampal body or tail, nor in whole brain volume was observed.
Van der Werff 2014 [24]	22 patients with long-term remission of CD, 22 healthy controls	DTI	Patients had widespread FA reductions in whole brain analysis. ROI analysis revealed reduced FA in the bilateral cingulate cingulum, bilateral uncinate fasciculus and corpus callosum. No significant differences were found in tracts in the inferior parts of the brainstem, the white matter in the bilateral cerebellum, the bilateral hippocampal cingulum, the left inferior fronto-occipital fasciculus, and parts of the bilateral superior longitudinal fasciculus. Patients also had increased radial and mean diffusivity, but no difference in axial diffusivity.

For peer review only

**Exogenous GC**			
Bentson 1978 [25]	15 long-term GC users	CT	Patients showed varying degrees of apparent cerebral atrophy. Some correlation between dosage and degree of atrophy appeared to be present.
Brown 2004 [26]	17 chronic (>6 months) GC (prednisone) users, 15 controls	Volumetric MRI, PMRS	GC users had smaller hippocampal volume, lower N-acetyl aspartate ratios, more mood symptoms and poorer cognitive function.
Brown 2015 [27]	17 healthy adults who received hydrocortisone (160 mg/day)/placebo, phenytoin/placebo, hydrocortisone/phenytoin, or placebo/placebo, in a randomized, blinded, cross-over trial with 21-day washout between conditions.	Volumetric MRI	Hydrocortisone use was not associated with difference in total brain volume but was associated with a 1.69% reduction in total hippocampal volume compared to placebo. Phenytoin blocked this hippocampal volume reduction by hydrocortisone.
Brown 2019 [28]	46 chronic GC users, randomized to memantine or placebo in blinded, cross-over trial (two 24-week treatment periods, separated by four-week washout)	Volumetric MRI	Hippocampal volume decreased significantly from baseline to week 52 and from week 24 to week 52, without significant difference between baseline and week 24. Following 24 weeks of memantine, left dentate gyrus/CA3 volume was significantly larger than after placebo; a similar trend was observed in the right CA1. Subiculum showed no significant differences.
Brown 2008 [29]	15 chronic (>6 months) GC (prednisone) users, 13 controls	Volumetric MRI	GC users had significantly smaller amygdala volumes compared to controls. Duration of GC therapy correlated negatively with right amygdala volume.
Desai 2009 [30]	28 chronic (>6 months) GC (prednisone) users, randomized to 24 weeks of lamotrigine (n = 16) or placebo (n = 12) in blinded trial	Volumetric MRI	After 24 weeks, amygdala volume was reduced in both groups, but right amygdala volume was significantly less reduced in the lamotrigine group than in the placebo group.
Nguyn 2019 [31]	81 chronic (>6 months) GC (prednisone) users	Volumetric MRI	Cumulative GC exposure negatively associated with the volumes of the left and right hippocampal dentate gyrus/CA3; no associations were found for entorhinal, perirhinal, or parahippocampal gyri, subiculum, or CA1.

AD, axial diffusivity, ACC, anterior cingulate cortex; CD, Cushing disease; CS, Cushing syndrome; CT, computed tomography; FA, fractional anisotropy; DKI, diffusional kurtosis imaging; DMN, default mode network; GC, glucocorticoids; GMV, grey matter volume; MD, mean diffusivity; MFG, medial frontal gyrus; NFA, non-functioning pituitary adenoma; PCC, posterior cingulate cortex; PMRS, proton magnetic resonance spectroscopy; RD, radial diffusivity; RSFC, resting-state functional connectivity; TSS, transsphenoidal surgery; 24h UFC, 24-hour urinary free cortisol.

For peer review only

References

1. Andela, C.D., van der Werff, S.J., Pannekoek, J.N., van den Berg, S.M., Meijer, O.C., van Buchem, M.A., Rombouts, S.A., van der Mast, R.C., Romijn, J.A., Tiemensma, J., Biermasz, N.R., van der Wee, N.J., Pereira, A.M.: Smaller grey matter volumes in the anterior cingulate cortex and greater cerebellar volumes in patients with long-term remission of Cushing's disease: a case-control study. Eur J Endocrinol **169**(6), 811-819 (2013). doi:10.1530/eje-13-0471
2. Bauduin, S., van der Pal, Z., Pereira, A.M., Meijer, O.C., Giltay, E.J., van der Wee, N.J.A., van der Werff, S.J.A.: Cortical thickness abnormalities in long-term remitted Cushing's disease. Transl Psychiatry **10**(1), 293 (2020). doi:10.1038/s41398-020-00980-6
3. Bourdeau, I., Bard, C., Noël, B., Leclerc, I., Cordeau, M.P., Bélair, M., Lesage, J., Lafontaine, L., Lacroix, A.: Loss of brain volume in endogenous Cushing's syndrome and its reversibility after correction of hypercortisolism. J Clin Endocrinol Metab **87**(5), 1949-1954 (2002). doi:10.1210/jcem.87.5.8493
4. Burkhardt, T., Lüdecke, D., Spies, L., Wittmann, L., Westphal, M., Flitsch, J.: Hippocampal and cerebellar atrophy in patients with Cushing's disease. Neurosurg Focus **39**(5), E5 (2015). doi:10.3171/2015.8.Focus15324
5. Chen, Y., Zhang, J., Tan, H., Li, J., Yu, Y.: Detrimental effects of hypercortisolism on brain structure and related risk factors. Sci Rep **10**(1), 12708 (2020). doi:10.1038/s41598-020-68166-0
6. Crespo, I., Esther, G.M., Santos, A., Valassi, E., Yolanda, V.G., De Juan-Delago, M., Webb, S.M., Gómez-Ansón, B., Resmini, E.: Impaired decision-making and selective cortical frontal thinning in Cushing's syndrome. Clinical endocrinology **81**(6), 826-833 (2014).
7. Hou, B., Gao, L., Shi, L., Luo, Y., Guo, X., Young, G.S., Qin, L., Zhu, H., Lu, L., Wang, Z., Feng, M., Bao, X., Wang, R., Xing, B., Feng, F.: Reversibility of impaired brain structures after transsphenoidal surgery in Cushing's disease: a longitudinal study based on an artificial intelligence-assisted tool. J Neurosurg, 1-10 (2020). doi:10.3171/2019.10.Jns191400
8. Jiang, H., Ren, J., He, N.Y., Liu, C., Sun, Y.H., Jian, F.F., Bian, L.G., Shen, J.K., Yan, F.H., Pan, S.J., Sun, Q.F.: Volumetric magnetic resonance imaging analysis in patients with short-term remission of Cushing's disease. Clin Endocrinol (Oxf) **87**(4), 367-374 (2017). doi:10.1111/cen.13381
9. Jiang, H., He, N.Y., Sun, Y.H., Jian, F.F., Bian, L.G., Shen, J.K., Yan, F.H., Pan, S.J., Sun, Q.F.: Altered gray and white matter microstructure in Cushing's disease: A diffusional kurtosis imaging study. Brain Res **1665**, 80-87 (2017). doi:10.1016/j.brainres.2017.04.007
10. Merke, D.P., Giedd, J.N., Keil, M.F., Mehlinger, S.L., Wiggs, E.A., Holzer, S., Rawson, E., Vaituzis, A.C., Stratakis, C.A., Chrousos, G.P.: Children experience cognitive decline despite reversal of brain atrophy one year after resolution of Cushing syndrome. J Clin Endocrinol Metab **90**(5), 2531-2536 (2005). doi:10.1210/jc.2004-2488
11. Momose, K.J., Kjellberg, R.N., Kliman, B.: High incidence of cortical atrophy of the cerebral and cerebellar hemispheres in Cushing's disease. Radiology **99**(2), 341-348 (1971). doi:10.1148/99.2.341
12. Pires, P., Santos, A., Vives-Gilabert, Y., Webb, S.M., Sainz-Ruiz, A., Resmini, E., Crespo, I., de Juan-Delago, M., Gómez-Anson, B.: White matter alterations in the brains of patients with active, remitted, and cured cushing syndrome: a DTI study. AJNR Am J Neuroradiol **36**(6), 1043-1048 (2015). doi:10.3174/ajnr.A4322

For peer review only

13. Pires, P., Santos, A., Vives-Gilabert, Y., Webb, S.M., Sainz-Ruiz, A., Resmini, E., Crespo, I., de Juan-Delago, M., Gómez-Anson, B.: White matter involvement on DTI-MRI in Cushing's syndrome relates to mood disturbances and processing speed: a case-control study. Pituitary **20**(3), 340-348 (2017). doi:10.1007/s11102-017-0793-y
14. Resmini, E., Santos, A., Gómez-Anson, B., Vives, Y., Pires, P., Crespo, I., Portella, M.J., de Juan-Delago, M., Barahona, M.J., Webb, S.M.: Verbal and visual memory performance and hippocampal volumes, measured by 3-Tesla magnetic resonance imaging, in patients with Cushing's syndrome. J Clin Endocrinol Metab **97**(2), 663-671 (2012). doi:10.1210/jc.2011-2231
15. Santos, A., Resmini, E., Crespo, I., Pires, P., Vives-Gilabert, Y., Granell, E., Valassi, E., Gómez-Anson, B., Martínez-Momblán, M.A., Mataró, M., Webb, S.M.: Small cerebellar cortex volume in patients with active Cushing's syndrome. Eur J Endocrinol **171**(4), 461-469 (2014). doi:10.1530/eje-14-0371
16. Santos, A., Resmini, E., Gómez-Ansón, B., Crespo, I., Granell, E., Valassi, E., Pires, P., Vives-Gilabert, Y., Martínez-Momblán, M.A., de Juan, M., Mataró, M., Webb, S.M.: Cardiovascular risk and white matter lesions after endocrine control of Cushing's syndrome. Eur J Endocrinol **173**(6), 765-775 (2015). doi:10.1530/eje-15-0600
17. Santos, A., Granell, E., Gómez-Ansón, B., Crespo, I., Pires, P., Vives-Gilabert, Y., Valassi, E., Webb, S.M., Resmini, E.: Depression and Anxiety Scores Are Associated with Amygdala Volume in Cushing's Syndrome: Preliminary Study. Biomed Res Int **2017**, 2061935 (2017). doi:10.1155/2017/2061935
18. Simmons, N.E., Do, H.M., Lipper, M.H., Laws, E.R., Jr.: Cerebral atrophy in Cushing's disease. Surg Neurol **53**(1), 72-76 (2000). doi:10.1016/s0090-3019(99)00197-4
19. Starkman, M.N., Gebarski, S.S., Berent, S., Schteingart, D.E.: Hippocampal formation volume, memory dysfunction, and cortisol levels in patients with Cushing's syndrome. Biological psychiatry **32**(9), 756-765 (1992).
20. Starkman, M.N., Giordani, B., Gebarski, S.S., Berent, S., Schork, M.A., Schteingart, D.E.: Decrease in cortisol reverses human hippocampal atrophy following treatment of Cushing's disease. Biol Psychiatry **46**(12), 1595-1602 (1999). doi:10.1016/s0006-3223(99)00203-6
21. Starkman, M.N., Giordani, B., Gebarski, S.S., Schteingart, D.E.: Improvement in learning associated with increase in hippocampal formation volume. Biol Psychiatry **53**(3), 233-238 (2003). doi:10.1016/s0006-3223(02)01750-x
22. Tirosh, A., RaviPrakash, H., Papadakis, G.Z., Tatsi, C., Belyavskaya, E., Charalampos, L., Lodish, M.B., Bagci, U., Stratakis, C.A.: Computerized Analysis of Brain MRI Parameter Dynamics in Young Patients With Cushing Syndrome-A Case-Control Study. J Clin Endocrinol Metab **105**(5), e2069-2077 (2020). doi:10.1210/clinem/dgz303
23. Toffanin, T., Nifosì, F., Follador, H., Passamani, A., Zonta, F., Ferri, G., Scanarini, M., Amistà, P., Pigato, G., Scaroni, C., Mantero, F., Carollo, C., Perini, G.I.: Volumetric MRI analysis of hippocampal subregions in Cushing's disease: a model for glucocorticoid neural modulation. Eur Psychiatry **26**(1), 64-67 (2011). doi:10.1016/j.eurpsy.2010.09.003
24. van der Werff, S.J., Andela, C.D., Nienke Pannekoek, J., Meijer, O.C., van Buchem, M.A., Rombouts, S.A., van der Mast, R.C., Biermasz, N.R., Pereira, A.M., van der Wee, N.J.: Widespread reductions of white matter integrity in patients with long-term remission of Cushing's disease. Neuroimage Clin **4**, 659-667 (2014). doi:10.1016/j.nicl.2014.01.017
25. Bentson, J., Reza, M., Winter, J., Wilson, G.: Steroids and apparent cerebral atrophy on computed tomography scans. J Comput Assist Tomogr **2**(1), 16-23 (1978). doi:10.1097/00004728-197801000-00003

For peer review only

26. Brown, E.S., D, J.W., Frol, A., Bobadilla, L., Khan, D.A., Hanczyc, M., Rush, A.J., Fleckenstein, J., Babcock, E., Cullum, C.M.: Hippocampal volume, spectroscopy, cognition, and mood in patients receiving corticosteroid therapy. Biol Psychiatry **55**(5), 538-545 (2004). doi:10.1016/j.biopsych.2003.09.010

27. Brown, E.S., Jeon-Slaughter, H., Lu, H., Jamadar, R., Issac, S., Shad, M., Denniston, D., Tamminga, C., Nakamura, A., Thomas, B.P.: Hippocampal volume in healthy controls given 3-day stress doses of hydrocortisone. Neuropsychopharmacology **40**(5), 1216-1221 (2015). doi:10.1038/npp.2014.307

28. Brown, E.S., Kulikova, A., Van Enkevort, E., Nakamura, A., Ivleva, E.I., Tustison, N.J., Roberts, J., Yassa, M.A., Choi, C., Frol, A., Khan, D.A., Vazquez, M., Holmes, T., Malone, K.: A randomized trial of an NMDA receptor antagonist for reversing corticosteroid effects on the human hippocampus. Neuropsychopharmacology **44**(13), 2263-2267 (2019). doi:10.1038/s41386-019-0430-8

29. Brown, E.S., Woolston, D.J., Frol, A.B.: Amygdala volume in patients receiving chronic corticosteroid therapy. Biol Psychiatry **63**(7), 705-709 (2008). doi:10.1016/j.biopsych.2007.09.014

30. Desai, S., Khanani, S., Shad, M.U., Brown, E.S.: Attenuation of amygdala atrophy with lamotrigine in patients receiving corticosteroid therapy. J Clin Psychopharmacol **29**(3), 284-287 (2009). doi:10.1097/JCP.0b013e3181a3e2a7

31. Nguyen, D.M., Yassa, M.A., Tustison, N.J., Roberts, J.M., Kulikova, A., Nakamura, A., Ivleva, E.I., Van Enkevort, E., Brown, E.S.: The Relationship Between Cumulative Exogenous Corticosteroid Exposure and Volumes of Hippocampal Subfields and Surrounding Structures. J Clin Psychopharmacol **39**(6), 653-657 (2019). doi:10.1097/jcp.0000000000001120

For peer review only

Abbreviation	Meaning
GC	Glucocorticoids
FDR	False discovery rate
Lower CI	Lower end of the 95% confidence interval
Upper	Higher end of the 95% confidence interval

For peer review only

Post-hoc tests of demographic variables

		ANOVA of continuous demo	
		**F value**	**P value**
**Main population**	**Age**	13.0	2.4E-06
	**BMI**	5.8	2.9E-03
	**Body fat percentage**	15.3	2.2E-07
**Chronic users**	**Body fat percentage**	7.7	4.6E-04
**Population without exclusion criteria**	**Age**	21.8	3.6E-10
	**BMI**	8.4	2.2E-04
	**Body fat percentage**	14.4	5.5E-07

For peer review only

ographic variables	Post-hoc Dunnett's test			
	Oral GC vs. controls			
	Estimate	Lower CI	Upper CI	P value
	2.6	1.4	3.7	**<.0001**
	0.03	-0.6	0.7	0.98
	0.7	-0.5	1.9	0.36
	-0.2	-3.0	2.6	0.97
	2.4	1.5	3.4	**<.0001**
	0.2	-0.4	0.7	0.67
	0.8	-0.2	1.8	0.16

For peer review only

Inhalation GC vs. controls

Estimate	Lower CI	Upper CI	P value
-0.2	-0.9	0.5	0.81
0.6	0.2	1.0	**0.0013**
1.9	1.1	2.7	**<.0001**
1.8	0.8	2.9	**0.0002**
-0.9	-1.5	-0.3	**0.0020**
0.6	0.3	1.0	**0.0001**
1.5	0.9	2.2	**<.0001**

For peer review only

Primary comparison: oral GC vs. inhalation GC vs. controls

	ANOVA of imaging parameters		
	**F value**	**P value**	**P_FDR**
**Volumetric measures**			
Global volumes			
Total brain volume	19.7	2.8E-09	**2.0E-08**
Grey matter volume	23.7	5.4E-11	**6.5E-10**
White matter volume	6.7	1.2E-03	**2.0E-03**
Peripheral cortex	21.1	6.9E-10	**6.2E-09**
CSF volume	10.1	4.2E-05	**9.5E-05**
Subcortical volumes			
Accumbens	12.0	6.0E-06	**1.7E-05**
Amygdala	3.1	4.4E-02	5.4E-02
Caudate	6.7	1.3E-03	**2.0E-03**
Hippocampus	2.5	8.0E-02	8.7E-02
Pallidum	7.7	4.5E-04	**7.8E-04**
Putamen	10.9	1.8E-05	**4.6E-05**
Thalamus	8.2	2.7E-04	**4.9E-04**
Regional grey matter volumes			
Amygdala	23.8	5.0E-11	**6.5E-10**
Caudate	13.0	2.3E-06	**7.5E-06**
Cerebellum	10.8	2.0E-05	**4.8E-05**
Cingulate gyrus, anterior	2.6	7.4E-02	8.3E-02
Cingulate gyrus, posterior	1.2	3.1E-01	3.2E-01
Cuneal cortex	2.4	9.1E-02	9.6E-02
Hippocampus	2.7	6.8E-02	7.9E-02
Insular cortex	8.5	2.0E-04	**3.9E-04**
Medial frontal gyrus	0.5	6.1E-01	6.1E-01
Precuneal cortex	5.5	4.3E-03	**5.6E-03**
**DTI measures**			
Fractional anisotropy			
Global	19.2	4.6E-09	**2.8E-08**
Body of corpus callosum	10.0	4.7E-05	**1.0E-04**
Genu of corpus callosum	16.8	5.3E-08	**2.1E-07**
Splenium of corpus callosum	5.4	4.4E-03	**5.6E-03**
Cingulum cingulate	6.1	2.4E-03	**3.4E-03**
Cingulum hippocampus	6.4	1.7E-03	**2.5E-03**
Uncinate	2.8	6.1E-02	7.3E-02
Mean diffusivity			
Global	25.9	5.8E-12	**2.1E-10**
Body of corpus callosum	15.5	2.0E-07	**7.0E-07**
Genu of corpus callosum	18.0	1.6E-08	**7.0E-08**
Splenium of corpus callosum	9.7	6.2E-05	**1.2E-04**
Cingulum cingulate	5.4	4.3E-03	**5.6E-03**

For peer review only

Cingulum hippocampus	18.5	9.1E-09	**4.7E-08**
Uncinate	12.1	5.4E-06	**1.6E-05**

For peer review only

Post-hoc Dunnett's test

Oral GC vs. controls				Inhalation GC
**Estimate**	**Lower CI**	**Upper CI**	**P value**	**Estimate**
-3688	-10627	3252	0.39	3374
-1968	-5904	1968	0.44	1012
-1720	-6273	2833	0.61	2362
-3303	-6843	237	0.072	1033
1215	-824	3254	0.32	78
-13.1	-26.7	0.5	0.062	-6.5
77.8	24.5	131.1	**0.0023**	-2.7
0.8	-29.9	31.4	1.00	-18.0
-31.3	-98.2	35.6	0.48	-27.9
3.6	-74.0	81.1	0.99	-6.4
-4.0	-31.9	23.8	0.91	-23.9
178.7	82.2	275.0	**1.0E-04**	41.2
25.1	-18.4	68.5	0.34	-12.2
-36.2	-108.4	36.0	0.43	5.0
-21.5	-179.0	136.3	0.92	-7.4
-0.0037	-0.0064	-0.0010	**0.0042**	-0.0023
-0.0043	-0.0084	-1.2E-04	**0.043**	-0.0023
-0.0064	-0.011	-0.0017	**0.005**	-0.0019
-0.0021	-0.0053	0.0012	0.27	-0.0032
-0.0017	-0.0062	0.0028	0.61	-0.0028
6.5E-05	-0.0046	0.0048	1.00	-0.0034
7.2E-06	3.2E-06	1.1E-05	**1.0E-04**	2.7E-06
6.9E-06	1.7E-06	1.2E-05	**6.0E-03**	4.8E-06
8.4E-06	2.2E-06	1.5E-05	**4.9E-03**	4.1E-06
4.4E-06	-3.8E-08	8.9E-06	5.2E-02	5.3E-06
2.9E-06	-8.5E-07	6.6E-06	1.6E-01	2.8E-06

For peer review only

5.0E-06	4.2E-07	9.5E-06	**2.9E-02**	5.6E-06
6.4E-06	2.2E-06	1.1E-05	**1.4E-03**	2.2E-06

For peer review only

: vs. controls

Lower CI	Upper CI	P value
-1012	7760	0.16
-1476	3500	0.57
-516	5240	0.13
-1205	3270	0.49
-1211	1367	0.98
-15.1	2.1	0.17
-36.4	30.9	0.97
-37.3	1.42	0.074
-70.2	14.4	0.25
-55.4	42.6	0.93
-41.5	-6.2	**0.0052**
-19.8	102.0	0.24
-39.7	15.3	0.51
-40.6	50.7	0.95
-107.0	92.4	0.97
-0.0040	-5.7E-04	**0.0057**
-0.0049	3.0E-04	0.092
-0.0049	0.0011	0.27
-0.0052	-0.0012	**0.0010**
-0.0057	8.9E-06	0.051
-0.0063	-3.8E-04	**0.024**
1.7E-07	5.2E-06	**3.4E-02**
1.6E-06	8.1E-06	**2.0E-03**
1.7E-07	8.0E-06	**3.9E-02**
2.4E-06	8.1E-06	**1.0E-04**
4.7E-07	5.2E-06	**1.5E-02**

For peer review only

2.8E-06	8.5E-06	**<.0001**
-4.4E-07	4.9E-06	1.2E-01

For peer review only

Subanalysis: chronic oral GC vs. chronic inhalation GC vs. controls

ANOVA of imaging parameters

	F value	P value	P_FDR
**Volumetric measures**			
Global volumes			
Total brain volume	17.0	4.1E-08	**1.5E-06**
Grey matter volume	12.2	5.0E-06	**9.1E-05**
White matter volume	10.8	2.0E-05	**1.8E-04**
Peripheral cortex	8.5	2.1E-04	**9.4E-04**
CSF volume	3.0	5.2E-02	**7.4E-02**
Subcortical volumes			
Accumbens	0.4	7.0E-01	7.1E-01
Amygdala	5.8	2.9E-03	**8.2E-03**
Caudate	7.2	7.6E-04	**2.9E-03**
Hippocampus	4.9	7.8E-03	**1.7E-02**
Pallidum	7.1	7.9E-04	**2.9E-03**
Putamen	5.0	6.9E-03	**1.6E-02**
Thalamus	6.7	1.3E-03	**4.1E-03**
Regional grey matter volumes			
Amygdala	10.1	4.2E-05	**3.0E-04**
Caudate	1.5	2.2E-01	2.4E-01
Cerebellum	4.1	1.6E-02	**2.9E-02**
Cingulate gyrus, anterior	0.2	8.3E-01	8.3E-01
Cingulate gyrus, posterior	4.2	1.6E-02	**2.9E-02**
Cuneal cortex	2.9	5.4E-02	7.4E-02
Hippocampus	9.1	1.1E-04	**6.6E-04**
Insular cortex	3.0	4.7E-02	7.1E-02
Medial frontal gyrus	1.6	2.1E-01	2.4E-01
Precuneal cortex	8.6	1.8E-04	**9.1E-04**
**DTI measures**			
Fractional anisotropy			
Global	5.4	4.4E-03	**1.1E-02**
Body of corpus callosum	2.8	5.8E-02	7.8E-02
Genu of corpus callosum	5.8	3.2E-03	**8.2E-03**
Splenium of corpus callosum	2.7	6.5E-02	8.3E-02
Cingulum cingulate	2.3	1.0E-01	1.3E-01
Cingulum hippocampus	3.7	2.4E-02	**3.9E-02**
Uncinate	1.3	2.7E-01	2.9E-01
Mean diffusivity			
Global	4.7	9.5E-03	**1.9E-02**
Body of corpus callosum	3.3	3.6E-02	5.7E-02
Genu of corpus callosum	6.3	1.8E-03	**5.3E-03**
Splenium of corpus callosum	3.9	2.0E-02	**3.5E-02**
Cingulum cingulate	0.5	6.3E-01	6.7E-01

For peer review only

Cingulum hippocampus	11.6	9.0E-06	**1.1E-04**
Uncinate	2.0	1.3E-01	1.6E-01

For peer review only

Post-hoc Dunnett's test

Oral GC vs. controls				Inhalation GC
Estimate	Lower CI	Upper CI	P value	Estimate
-2535	-18869	13798	0.90	3553
-1552	-10808	7703	0.89	1636
-984	-11702	9735	0.96	1917
-2152	-10481	6177	0.78	940
-2408	-7198	2381	0.43	154
-9.9	-42	22.1	0.49	-1.8
52.1	-19.3	123.5	0.19	-20.6
112.7	-12.9	238.2	0.09	-5.0
59.2	-79.1	197.5	0.54	-38.4
4.01	-68.2	76.2	0.98	-23.0
-65.4	-222.8	92.0	0.55	-26.1
61.9	-120.7	244.5	0.66	-11.6
4.8	-60.8	70.3	0.97	-15.1
79.6	-147.1	306.0	0.65	57.1
25.7	-76.5	127.9	0.79	4.4
36.0	-158.8	230.7	0.87	25.5
63.5	-52.4	179.5	0.37	-24.3
-110.3	-280.0	59.3	0.26	34.4
170.0	-201.0	541.2	0.49	-59.9
-0.0066	-0.013	-3.2E-04	**0.038**	-0.0025
-0.0066	-0.016	0.0032	0.24	-0.0028
-0.014	-0.025	-0.0031	**0.0087**	-0.0020
-0.0049	-0.012	0.0028	0.27	-0.0032
0.00033	-0.010	0.0109	0.99	-0.0034
0.0032	-0.0078	0.014	0.73	-0.0034
9.4E-06	8.7E-08	1.9E-05	**0.048**	2.6E-06
1.1E-05	-1.6E-06	2.3E-05	0.10	4.4E-06
2.0E-05	5.5E-06	3.5E-05	**0.0043**	2.8E-06
8.1E-06	-2.4E-06	1.9E-05	0.16	5.2E-06
3.5E-06	-5.3E-06	1.2E-05	0.58	2.0E-06

For peer review only

8.2E-06	-2.4E-06	1.9E-05	0.16	6.3E-06
6.5E-06	-3.4E-06	1.6E-05	0.25	2.0E-06

For peer review only

vs. controls

Lower CI	Upper CI	P value
-2340	9445	0.31
-1703	4975	0.45
-1950	5784	0.44
-2065	3945	0.70
-1573	1882	0.96
-11.7	11.4	0.97
-46.4	5.2	0.14
-50.3	40.3	0.95
-88.3	11.5	0.16
-49.0	3.1	0.094
-82.9	30.7	0.49
-77.5	54.3	0.88
-38.8	8.5	0.27
-24.7	139.0	0.22
-32.4	41.3	0.94
-44.8	95.8	0.63
-66.1	17.6	0.34
-26.8	95.6	0.36
-194.0	74.1	0.51
-0.0048	-2.3E-04	**0.027**
-0.0063	7.2E-04	0.14
-0.0060	0.0020	0.44
-0.0060	-4.9E-04	**0.017**
-0.0072	4.5E-04	0.093
-0.0074	6.4E-04	0.11
-7.7E-07	6.0E-06	0.16
4.7E-08	8.8E-06	**0.05**
-2.5E-06	8.0E-06	0.40
1.4E-06	9.0E-06	**0.0044**
-1.2E-06	5.2E-06	0.28

For peer review only

2.5E-06	1.0E-05	**5.0E-04**
-1.6E-06	5.6E-06	0.36

For peer review only

Primary comparison: oral GC vs. inhalation GC vs. controls

	ANOVA of cognitive parameters			Post-hoc Dun[illegible]
				Oral GC vs. co[illegible]
	F value	P value	P_FDR	Estimate
Reaction time	1.0	0.37	0.41	
Fluid intelligence score	0.89	0.41	0.41	
Digit span	1.4	0.25	0.38	
Trail making A	5.6	0.0036	0.0073	-0.11
Trail making B	6.1	0.0023	0.0068	-0.12
Symbol substitution	10.3	3.5E-05	2.1E-04	-0.17

For peer review only

nett's test

ntrols				Inhalation GC vs. controls		
Lower CI	Upper CI	P value		Estimate	Lower CI	Upper CI
-0.28	0.06	0.25		-0.03	-0.15	0.09
-0.30	0.05	0.19		-0.01	-0.13	0.11
-0.34	-0.008	**0.038**		-0.04	-0.15	0.08

For peer review only

P value
0.78
0.98
0.72

For peer review only

Primary comparison: oral GC vs. inhalation GC vs. controls

	Likelihood ratio test of emotional parameters			Post-hoc pairv
				Oral GC vs. co
	Chi squared statistic	P value	P_FDR	OR
Depression	10.6	0.0049	0.0049	1.76
Disinterest	10.9	0.0043	0.0049	1.84
Tenseness	13.4	0.0012	0.0025	1.78
Tiredness	32.4	9.2E-08	3.7E-07	1.90

For the pairwise comparisons, P values in bold are statistically significant after Bonferroni correctio

For peer review only

wise comparisons

ontrols			Inhalation GC vs. controls		
	95% CI	P value	OR	95% CI	P value
	1.25; 2.43	**0.00082**	1.10	0.87; 1.38	0.43
	1.29; 2.56	**0.00051**	1.06	0.82; 1.36	0.64
	1.29; 2.41	**0.00030**	1.16	0.92; 1.43	0.19
	1.45; 2.50	**4.4E-06**	1.35	1.14; 1.60	**6.3E-04**

n for family-wise error rate of two tests (P < 0.025)

For peer review only

Subanalysis: chronic oral GC vs. chronic inhalation GC vs. controls

	ANOVA of cognitive parameters			Post-hoc Dun
				Oral GC vs. co
	F value	P value	P_FDR	Estimate
Reaction time	0.17	0.84	0.84	
Fluid intelligence score	1.1	0.34	0.84	
Digit span	3.5	0.031	0.19	
Trail making A	0.41	0.66	0.84	0.12
Trail making B	0.28	0.75	0.84	-0.080
Symbol substitution	0.35	0.70	0.84	-0.078

For peer review only

nett's test

ntrols				Inhalation GC vs. controls		
Lower CI	Upper CI	P value		Estimate	Lower CI	Upper CI
-0.26	0.51	0.69		-0.070	-0.24	0.10
-0.47	0.31	0.84		-4.7E-04	-0.17	0.17
-0.45	0.30	0.84		-0.049	-0.21	0.11

For peer review only

P value
0.55
1.00
0.71

For peer review only

Subanalysis: chronic oral GC vs. chronic inhalation GC vs. controls

	Likelihood ratio test of emotional parameters			Post-hoc pairv
				Oral GC vs. co
	Chi squared statistic	P value	P_FDR	OR
Depression	1.1	0.57	0.57	1.21
Disinterest	2.2	0.33	0.44	1.41
Tenseness	2.5	0.28	0.44	1.84
Tiredness	4.4	0.11	0.44	0.96

For the pairwise comparisons, P values in bold are statistically significant after Bonferroni c

For peer review only

wise comparisons

ontrols			Inhalation GC vs. controls		
95% CI	P value		OR	95% CI	P value
0.45; 2.73	0.67		0.85	0.59; 1.18	0.34
0.53; 3.17	0.44		0.79	0.53; 1.13	0.21
0.84; 3.68	0.10		1.05	0.78; 1.40	0.73
0.49; 1.84	0.91		1.28	1.01; 1.61	**3.7E-02**

:orrection for family-wise error rate of two tests (P < 0.025)

For peer review only

Sensitivity analysis among participants without exclusion based on p

ANOVA of imaging parameters

	F value	P value	P_FDR
**Volumetric measures**			
Global volumes			
Total brain volume	17.7	2.2E-08	**1.3E-07**
Grey matter volume	22.3	2.0E-10	**2.4E-09**
White matter volume	5.5	4.1E-03	**6.7E-03**
Peripheral cortex	24.6	2.0E-11	**4.4E-10**
CSF volume	14.2	7.1E-07	**2.3E-06**
Subcortical volumes			
Accumbens	10.2	3.8E-05	**1.0E-04**
Amygdala	1.5	2.2E-01	2.4E-01
Caudate	4.5	1.1E-02	**1.7E-02**
Hippocampus	2.1	1.2E-01	1.5E-01
Pallidum	6.9	1.0E-03	**1.9E-03**
Putamen	9.8	5.6E-05	**1.5E-04**
Thalamus	9.3	9.4E-05	**2.3E-04**
Regional grey matter volumes			
Amygdala	21.0	7.8E-10	**7.0E-09**
Caudate	12.3	4.7E-06	**1.4E-05**
Cerebellum	5.8	3.1E-03	**5.2E-03**
Cingulate gyrus, anterior	1.6	2.0E-01	2.2E-01
Cingulate gyrus, posterior	0.6	5.3E-01	5.5E-01
Cuneal cortex	1.5	2.2E-01	2.4E-01
Hippocampus	1.8	1.6E-01	1.9E-01
Insular cortex	8.7	1.7E-04	**3.5E-04**
Medial frontal gyrus	0.6	5.7E-01	5.7E-01
Precuneal cortex	4.0	1.9E-02	**2.7E-02**
**DTI measures**			
Fractional anisotropy			
Global	15.5	1.8E-07	**9.4E-07**
Body of corpus callosum	8.9	1.4E-04	**3.1E-04**
Genu of corpus callosum	15.2	2.5E-07	**1.1E-06**
Splenium of corpus callosum	2.2	1.1E-01	1.3E-01
Cingulum cingulate	3.8	2.3E-02	**3.1E-02**
Cingulum hippocampus	2.7	6.4E-02	8.6E-02
Uncinate	2.5	8.2E-02	1.1E-01
Mean diffusivity			
Global	24.5	2.4E-11	**4.4E-10**
Body of corpus callosum	14.2	6.7E-07	**2.3E-06**
Genu of corpus callosum	17.9	1.7E-08	**1.2E-07**
Splenium of corpus callosum	6.7	1.2E-03	**2.2E-03**

For peer review only

Cingulum cingulate	4.9	7.6E-03	**1.2E-02**
Cingulum hippocampus	14.5	4.9E-07	**2.0E-06**
Uncinate	7.3	6.6E-04	**1.3E-03**

For peer review only

ɔsychiatric, neurological or endocrine history (oral GC vs. inhalation GC vs. c

Post-hoc Dunnett's test				
**Oral GC vs. controls**				**Inhalation GC**
**Estimate**	**Lower CI**	**Upper CI**	**P value**	**Estimate**
-3460	-9320	2400	0.32	3535
-2224	-5577	1130	0.25	1454
-1237	-5078	2604	0.69	2080
-3318	-6330	-307	**0.028**	1172
1220	-518	2958	0.12	223
-8.9	-20.4	2.7	0.16	-3.7
58.6	13.8	103.5	**0.0072**	-5.9
1.2	-24.5	27.0	0.99	-16.2
-33.8	-90.5	22.9	0.32	-20.1
-19.9	-86.2	46.5	0.72	-10.7
-6.7	-30.4	17.1	0.75	-21.7
149.6	66.9	232.4	**0.00010**	42.9
17.8	-19.4	54.9	0.47	-2.9
-42.1	-103.5	19.4	0.23	8.0
-9.7	-142.8	123.4	0.97	-1.7
-0.0031	-0.0055	-7.5E-04	**0.0066**	-0.0015
-0.0039	-0.0076	-0.0003	**0.032**	-0.0014
-0.0056	-0.0097	-0.0014	**0.0055**	-0.0013
-0.0014	-0.0052	0.0025	0.64	-0.0018
6.6E-06	3.0E-06	1.0E-05	**3.7E-05**	1.9E-06
6.7E-06	1.9E-06	1.1E-05	**0.0034**	4.0E-06
8.0E-06	2.5E-06	1.4E-05	**0.0023**	3.3E-06
3.7E-06	-3.1E-07	7.6E-06	0.076	4.0E-06

For peer review only

2.5E-06	-6.8E-07	5.7E-06	0.15	2.2E-06
2.6E-06	-1.3E-06	6.6E-06	0.25	4.5E-06
4.0E-06	2.9E-07	7.7E-06	**0.032**	1.6E-06

For peer review only

:ontrols)

: vs. controls		
Lower CI	Upper CI	P value
-121	7190	0.060
-637	3546	0.22
-316	4476	0.10
-706	3051	0.29
-861	1307	0.65
-10.9	3.5	0.41
-33.9	22.1	0.84
-32.3	-0.2	**0.047**
-55.5	15.3	0.35
-52.1	30.7	0.78
-36.5	-6.8	**0.0023**
-8.7	94.5	0.12
-26.1	20.3	0.93
-30.3	46.3	0.84
-84.7	81.3	1.00
-0.0030	-4.9E-05	**0.041**
-0.0036	8.9E-04	0.30
-0.0039	0.0013	0.44
-0.0042	5.9E-04	0.17
-3.2E-07	4.1E-06	0.057
1.1E-06	7.0E-06	**0.0048**
-1.4E-07	6.7E-06	0.062
1.5E-06	6.4E-06	**7.0E-04**

For peer review only

2.2E-07	4.2E-06	**0.026**
2.0E-06	7.0E-06	**1.0E-04**
-7.5E-07	3.9E-06	0.23

For peer review only

Sensitivity analysis among participants without exclusion based on psychia

	ANOVA			Post-hoc Dunn
				Oral GC vs. co
	F value	P value	P_FDR	Estimate
Reaction time	1.0	0.35	0.42	
Fluid intelligence score	1.9	0.15	0.23	
Digit span	0.5	0.63	0.63	
Trail making A	6.6	0.0014	**0.0028**	-0.11
Trail making B	6.7	0.0013	**0.0028**	-0.12
Symbol substitution	9.7	6.2E-05	**0.00037**	-0.15

For peer review only

atric, neurological or endocrine history (oral GC vs. inhalation GC vs. contro

nett's test

ontrols				Inhalation GC vs. controls		
	Lower CI	Upper CI	P value	Estimate	Lower CI	Upper CI
	-0.26	0.03	0.16	0.020	-0.08	0.12
	-0.27	0.02	0.10	-0.018	-0.12	0.08
	-0.29	-0.01	**0.029**	-0.061	-0.16	0.04

For peer review only

ls)

P value
0.86
0.88
0.28

For peer review only

Sensitivity analysis among participants without exclusion based on psyc

	Likelihood ratio test of emotional parameters			Post-hoc pairv
				Oral GC vs. co
	Chi squared statistic	P value	P_FDR	OR
Depression	11.1	0.0039	0.0039	1.44
Disinterest	17.8	0.00014	1.9E-04	1.73
Tenseness	24.0	6.1E-06	1.2E-05	1.68
Tiredness	39.2	3.1E-09	1.2E-08	1.79

For the pairwise comparisons, P values in bold are statistically significant after Bonferroni co

For peer review only

:hiatric, neurological or endocrine history (oral GC vs. inhalation GC vs. con

wise comparisons

›ntrols		Inhalation GC vs. controls		
**95% CI**	**P value**	**OR**	**95% CI**	**P value**
1.08; 1.89	**0.010**	1.23	1.03; 1.46	**0.023**
1.31; 2.27	**8.5E-05**	1.21	1.00; 1.45	0.041
1.29; 2.16	**7.0E-05**	1.31	1.11; 1.54	**0.0014**
1.42; 2.27	**9.0E-07**	1.33	1.15; 1.53	**0.00011**

rrection for family-wise error rate of two tests (P < 0.025)

For peer review only

trols)

For peer review only

	Oral GC users (n = 222)	
	**Number of outliers**	**%**
**Volumetric measures**		
Global volumes		
Total brain volume	2	0.9
Grey matter volume	6	2.7
White matter volume	2	0.9
Peripheral cortex	6	2.7
CSF volume	9	4.1
Subcortical volumes		
Accumbens	2	0.9
Amygdala	3	1.4
Caudate	5	2.3
Hippocampus	3	1.4
Pallidum	2	0.9
Putamen	7	3.2
Thalamus	4	1.8
Regional grey matter volumes		
Amygdala	5	2.3
Caudate	18	8.1
Cerebellum	11	5.0
Cingulate gyrus, anterior	7	3.2
Cingulate gyrus, posterior	5	2.3
Cuneal cortex	3	1.4
Hippocampus	4	1.8
Insular cortex	6	2.7
Medial frontal gyrus	3	1.4
Precuneal cortex	7	3.2
**DTI measures**		
Fractional anisotropy		
Global	2	0.9
Body of corpus callosum	4	1.8
Genu of corpus callosum	10	4.5
Splenium of corpus callosum	4	1.8
Cingulum cingulate	4	1.8
Cingulum hippocampus	3	1.4
Uncinate	1	0.5
Mean diffusivity		
Global	7	3.2
Body of corpus callosum	3	1.4
Genu of corpus callosum	4	1.8
Splenium of corpus callosum	5	2.3
Cingulum cingulate	6	2.7

For peer review only

Cingulum hippocampus	4	1.8
Uncinate	4	1.8

For peer review only

Inhalation GC users (n = 557)		Controls (n = 24106)	
Number of outliers	%	Number of outliers	%
1	0.2	134	0.6
1	0.2	153	0.6
3	0.5	130	0.5
1	0.2	159	0.7
22	3.9	859	3.6
7	1.3	195	0.8
6	1.1	217	0.9
2	0.4	269	1.1
11	2.0	399	1.7
4	0.7	490	2.0
1	0.2	237	1.0
6	1.1	225	0.9
7	1.3	281	1.2
27	4.8	1138	4.7
6	1.1	316	1.3
21	3.8	758	3.1
11	2.0	359	1.5
2	0.4	304	1.3
7	1.3	248	1.0
2	0.4	239	1.0
4	0.7	229	0.9
2	0.4	216	0.9
12	2.2	388	1.6
13	2.3	456	1.9
12	2.2	520	2.2
20	3.6	331	1.4
4	0.7	215	0.9
4	0.7	277	1.1
6	1.1	221	0.9
11	2.0	613	2.5
15	2.7	468	1.9
11	2.0	535	2.2
12	2.2	543	2.3
10	1.8	447	1.9

For peer review only

16	2.9	343	1.4
6	1.1	322	1.3

For peer review only

Sensitivity analysis after exclusion of outlier values

ANOVA of imaging parameters

	F value	P value	P_FDR
**Volumetric measures**			
Global volumes			
Total brain volume	16.0	1.1E-07	**4.6E-07**
Grey matter volume	28.8	3.4E-13	**6.1E-12**
White matter volume	5.4	4.6E-03	**7.1E-03**
Peripheral cortex	27.0	2.0E-12	**1.8E-11**
CSF volume	16.8	5.0E-08	**2.3E-07**
Subcortical volumes			
Accumbens	13.0	2.3E-06	**5.8E-06**
Amygdala	2.7	6.9E-02	7.7E-02
Caudate	4.7	8.8E-03	**1.1E-02**
Hippocampus	5.4	4.7E-03	**7.1E-03**
Pallidum	4.9	7.4E-03	**9.8E-03**
Putamen	13.7	1.1E-06	**3.4E-06**
Thalamus	10.0	4.6E-05	**8.7E-05**
Regional grey matter volumes			
Amygdala	28.3	5.1E-13	**6.1E-12**
Caudate	12.6	3.5E-06	**8.4E-06**
Cerebellum	10.3	3.3E-05	**6.6E-05**
Cingulate gyrus, anterior	3.9	2.1E-02	**2.6E-02**
Cingulate gyrus, posterior	2.3	1.0E-01	1.1E-01
Cuneal cortex	1.5	2.2E-01	2.2E-01
Hippocampus	3.3	3.9E-02	**4.6E-02**
Insular cortex	13.1	2.0E-06	**5.5E-06**
Medial frontal gyrus	0.4	6.8E-01	6.8E-01
Precuneal cortex	5.2	5.4E-03	**7.5E-03**
**DTI measures**			
Fractional anisotropy			
Global	22.7	1.4E-10	**1.0E-09**
Body of corpus callosum	11.4	1.1E-05	**2.5E-05**
Genu of corpus callosum	15.3	2.3E-07	**8.4E-07**
Splenium of corpus callosum	2.7	6.4E-02	7.5E-02
Cingulum cingulate	6.5	1.5E-03	**2.5E-03**
Cingulum hippocampus	7.5	5.7E-04	**9.7E-04**
Uncinate	2.6	7.6E-02	8.3E-02
Mean diffusivity			
Global	29.1	2.4E-13	**6.1E-12**
Body of corpus callosum	17.1	3.6E-08	**1.9E-07**
Genu of corpus callosum	21.6	4.3E-10	**2.6E-09**
Splenium of corpus callosum	9.9	5.2E-05	**9.4E-05**

For peer review only

Cingulum cingulate	5.3	5.2E-03	**7.5E-03**
Cingulum hippocampus	13.7	1.1E-06	**3.4E-06**
Uncinate	11.3	1.2E-05	**2.5E-05**

P values in blue were not significant (P < 0.05) in the original analysis, but are in this analysis

For peer review only

Post-hoc Dunnett's test

Oral GC vs. controls				Inhalation GC
**Estimate**	**Lower CI**	**Upper CI**	**P value**	**Estimate**
-3991	-10852	2869	0.33	3756
-3143	-7081	794	0.14	1120
-1861	-6349	2626	0.55	2374
-4412	-7948	-876	0.011	1148
1437	-210	3084	0.10	-449
-15.6	-28.8	-2.3	0.018	-4.6
69.4	18.4	120.3	**0.0049**	4.5
-17.1	-71.2	37.0	0.70	-17
5.7	-20.5	31.8	0.83	-9.8
-63	-127.1	1.0	0.055	-19.9
-25.6	-98.2	46.9	0.64	-0.6
-17.2	-43.8	9.4	0.26	-22.6
138.1	67.7	208.6	**<.0001**	15.1
-1.1	-42.8	40.6	1.00	-6.6
110.5	-7.8	229.0	0.071	27.1
24.3	-22.4	70.9	0.41	2.4
-74.8	-143.2	-6.4	0.029	8.7
-60.1	-213.6	93.3	0.59	0.014
-0.0043	-0.0067	-0.0018	**2.0E-04**	-0.0019
-0.0048	-0.0086	-0.0010	**0.0097**	-0.0021
-0.0059	-0.010	-0.0016	**0.0048**	-0.0017
-0.0022	-0.0065	0.0021	0.42	-0.0026
-1.2E-04	-0.0046	0.0044	1.00	-0.0036
7.1E-06	3.7E-06	1.1E-05	**<.0001**	2.5E-06
7.5E-06	2.8E-06	1.2E-05	**7.0E-04**	3.7E-06
9.5E-06	3.9E-06	1.5E-05	**3.0E-04**	3.6E-06
4.6E-06	7.3E-07	8.4E-06	0.016	4.2E-06

For peer review only

2.6E-06	-9.4E-07	6.1E-06	0.19	2.6E-06
4.4E-06	2.5E-07	8.6E-06	**0.035**	4.3E-06
5.8E-06	1.9E-06	9.7E-06	**0.0018**	2.4E-06

after exclusion of outliers.

For peer review only

vs. controls

Lower CI	Upper CI	P value
-565	8076	0.10
-1337	3576	0.50
-454	5203	0.11
-1058	3355	0.41
-1492	594	0.53
-13.0	3.7	0.37
-27.4	36.3	0.92
-51.3	17.3	0.44
-26.3	6.7	0.32
-59.7	20.0	0.44
-46.2	45.1	1.00
-39.3	-5.9	**0.01**
-28.8	59.1	0.66
-32.5	19.3	0.78
-47.9	102.0	0.63
-27.0	31.8	0.97
-34.1	51.4	0.85
-95.6	95.6	1.00
-0.0035	-3.4E-04	**0.013**
-0.0045	3.4E-04	0.11
-0.0044	0.0010	0.28
-0.0053	9.7E-05	0.061
-0.0064	-7.5E-04	**0.010**
3.1E-07	4.7E-06	**0.022**
6.9E-07	6.6E-06	**0.012**
2.9E-08	7.1E-06	**0.048**
1.8E-06	6.7E-06	**2.0E-04**

For peer review only

3.6E-07	4.8E-06	**0.019**
1.6E-06	6.9E-06	**6.0E-04**
-8.8E-08	4.8E-06	0.061

For peer review only

	Oral GC users (n = 222)		Inhalation GC users (n = 557)
	**Number of outliers**	**%**	**Number of outliers**
Reaction time*	4	1.8	11
Fluid intelligence score	2	0.9	1
Digit span	1	0.5	24
Trail making A*	6	2.7	10
Trail making B*	2	0.9	2
Symbol substitution	5	2.3	3

* Reaction time, trail making A, and trail making B were log transformed for normalization.

For peer review only

	Controls (n = 24106)	
%	Number of outliers	%
2.0	389	1.6
0.2	50	0.2
4.3	79	0.3
1.8	423	1.8
0.4	338	1.4
0.5	172	0.7

For peer review only

Sensitivity analysis after exclusion of outlier values

	ANOVA			Post-hoc Dun[illegible]
				Oral GC vs. co[illegible]
	**F value**	**P value**	**P_FDR**	**Estimate**
Reaction time	1.0	0.379	0.45	
Fluid intelligence score	0.8	0.446	0.45	
Digit span	3.1	0.047	0.070	
Trail making A	5.2	0.0057	**0.011**	-0.10
Trail making B	9.6	6.8E-05	**2.0E-04**	-0.16
Symbol substitution	11.6	8.9E-06	**5.3E-05**	-0.18

For peer review only

nett's test

ontrols				Inhalation GC vs. controls		
Lower CI	Upper CI	P value		Estimate	Lower CI	Upper CI
-0.25	0.05	0.25		-0.02	-0.12	0.09
-0.32	-0.01	**0.04**		-0.06	-0.17	0.04
-0.34	-0.02	**0.02**		-0.05	-0.16	0.06

For peer review only

P value
0.88
0.31
0.55

For peer review only

Mediation analysis for total body fat percentage

Region		Estimate
Caudate, subcortical volume	ACME	-1.2
	ADE	78.5
	Total effect	77.4
	Proportion mediated	0.0
Amygdala, regional grey matter volume	ACME	-1.2
	ADE	1.2
	Total effect	-0.1
	Proportion mediated	17.7
Caudate, regional grey matter volume	ACME	-0.9
	ADE	184.0
	Total effect	183.0
	Proportion mediated	0.0
Global FA	ACME	-2.5E-05
	ADE	-3.8E-03
	Total effect	-3.8E-03
	Proportion mediated	6.6E-03
Global MD	ACME	-4.8E-08
	ADE	7.5E-06
	Total effect	7.5E-06
	Proportion mediated	-6.4E-03

ACME, average causal mediation effects; ADE, average direct effects.

For peer review only

Lower CI	Upper CI	P value	Total sample size used
-3.7	1.0	0.27	23338
31.2	124.6	<2e-16	
30.0	123.3	<2e-16	
-0.1	0.0	0.27	
-3.6	1.0	0.29	23338
-24.2	26.7	0.93	
-25.7	26.0	0.98	
-1.9	1.9	0.94	
-3.1	0.7	0.27	23338
79.4	293.5	<2e-16	
77.9	293.0	<2e-16	
0.0	0.0	0.27	
-8.6E-05	0	0.3	23338
-6.3E-03	0	<2e-16	
-6.4E-03	0	<2e-16	
-5.5E-03	4.0E-02	0.3	
-1.5E-07	0	0.31	23338
3.7E-06	0	<2e-16	
3.7E-06	0	<2e-16	
-2.5E-02	1.0E-02	0.31	

For peer review only

Supplements

Supplements 1, 2 and 3 are separate files.

For peer review only

Supplement 4. Characteristics of included chronic glucocorticoid users and controls

	Patients using chronic oral GC (n = 42)	Patients using chronic inhalation GC (n = 305)	Controls (n = 24106)	P value
**Sex: male, n (%)**	22 (52.4%)	137 (44.9%)	12154 (50.4%)	0.15
**Age at time of scanning in years, mean (SD)**	65.2 (7.0)	63.0 (7.6)	63.5 (7.5)	0.19
**Education level, n (%)**				0.81
College/University degree	24 (57.1)	171 (56.1)	12058 (50.0)	
A levels or equivalent	6 (14.3)	38 (12.5)	2930 (12.2)	
O levels/GCSE or equivalent	4 (9.5)	44 (14.4)	4155 (17.2)	
CSEs or equivalent	1 (2.4)	9 (3.0)	879 (3.6)	
NVQ, HND, HNC, or equivalent	1 (2.4)	14 (4.6)	1396 (5.8)	
Other professional qualifications	2 (4.8)	14 (4.6)	1150 (4.8)	
None of the above	1 (2.4)	14 (4.6)	1311 (5.4)	
Missing	3 (7.1)	1 (0.3)	227 (0.9)	
**BMI in kg/m^2, mean (SD)**	25.9 (3.7)	26.6 (4.4)	26.1 (4.1)	0.15
Number (%) missing	1 (2.4)	14 (4.6)	1325 (5.5)	
**Body fat percentage, mean (SD)**	30.0 (6.4)	32.0 (8.1)	30.2 (7.9)	4.6e-4
Number (%) missing	1 (2.4)	14 (4.6)	1331 (5.5)	
**Smoking status, n (%)**				0.42
Current	1 (2.4)	6 (2.0)	647 (2.7)	
Previous	8 (19.0)	112 (36.7)	7858 (32.6)	
Never	31 (73.8)	206 (67.5)	15380 (63.8)	
Missing	2 (4.8)	2 (0.7)	221 (0.9)	

For peer review only

BMI, body mass index; GC, glucocorticoids; n, number; SD, standard deviation.

P values were determined using analysis of variance (for continuous variables) and Fisher’s exact test (for categorical variables, because of the low number of patients using chronic glucocorticoids).

For peer review only

Supplement 5. Imaging parameters, presented as the adjusted mean difference of patients using chronic oral glucocorticoids (n = 42) or chronic inhalation glucocorticoids (n = 305) compared to controls (n = 24106)

	ANOVA			Oral GC vs. controls			Inhalation GC vs. controls		
	**F value**	**P value**	**P_{FDR}**	**AMD***	**95% CI**	**P value**	**AMD***	**95% CI**	**P value**
Volumetric measures									
Global volumes (in mm^3)									
Total brain volume	17.0	4.1e-8	**1.5e-6**	-2535	-18869; 13798	0.90	3553	-2340; 9445	0.31
Grey matter volume	12.2	5.0e-6	**9.1e-5**	-1552	-10808; 7703	0.89	1636	-1703; 4975	0.45
White matter volume	10.8	2.0e-5	**1.8e-4**	-984	-11702; 9735	0.96	1917	-1950; 5784	0.44
Peripheral cortex	8.5	2.1e-4	**9.4e-4**	-2152	-10481; 6177	0.78	940	-2065; 3945	0.70
CSF volume	3.0	5.2e-2	**7.4e-2**	-2408	-7198; 2381	0.43	154	-1573; 1882	0.96
Subcortical volumes (in mm^3)									
Amygdala	5.8	2.9e-3	**8.2e-3**	52.1	-19.3; 123.5	0.19	-20.6	-46.4; 5.2	0.14
Caudate	7.2	7.6e-4	**2.9e-3**	112.7	-12.9; 238.2	0.09	-5.0	-50.3; 40.3	0.95
Hippocampus	4.9	7.8e-3	**1.7e-2**	59.2	-79.1; 197.5	0.54	-38.4	-88.3; 11.5	0.16
Pallidum	7.1	7.9e-4	**2.9e-3**	4.01	-68.2; 76.2	0.98	-23.0	-49.0; 3.1	0.094
Putamen	5.0	6.9e-3	**1.6e-2**	-65.4	-222.8; 92.0	0.55	-26.1	-82.9; 30.7	0.49
Thalamus	6.7	1.3e-3	**4.1e-3**	61.9	-120.7; 244.5	0.66	-11.6	-77.5; 54.3	0.88
Regional grey matter volumes (in mm^3)									
Amygdala	10.1	4.2e-5	**3.0e-4**	4.8	-60.8; 70.3	0.97	-15.1	-38.8; 8.5	0.27
Cerebellum	4.1	1.6e-2	**2.9e-2**	25.7	-76.5; 127.9	0.79	4.4	-32.4; 41.3	0.94
Cingulate gyrus, posterior	4.2	1.6e-2	**2.9e-2**	36.0	-158.8; 230.7	0.87	25.5	-44.8; 95.8	0.63

For peer review only

Hippocampus	9.1	1.1e-4	**6.6e-4**	63.5	-52.4; 179.5	0.37	-24.3	-66.1; 17.6	0.34
Precuneal cortex	8.6	1.8e-4	**9.1e-4**	170.0	-201.0; 541.2	0.49	-59.9	-194.0; 74.1	0.51
DTI measures									
Fractional anisotropy									
Global	5.4	4.4e-3	**1.1e-2**	-0.0066	-0.013; -3.2e-4	**0.038**	-0.0025	-0.0048; -2.3e-4	**0.027**
Genu of corpus callosum	5.8	3.2e-3	**8.2e-3**	-0.014	-0.025; -0.0031	**0.0087**	-0.0020	-0.0060; 0.0020	0.44
Cingulum hippocampus	3.7	2.4e-2	**3.9e-2**	0.0032	-0.0078; 0.014	0.73	-0.0034	-0.0074; 6.4e-4	0.11
Mean diffusivity									
Global	4.7	9.5e-3	**1.9e-2**	9.4e-6	8.7e-8; 1.9e-5	**0.05**	2.6e-6	-7.7e-7; 6.0e-6	0.16
Genu of corpus callosum	6.3	1.8e-3	**5.3e-3**	2.0e-5	5.5e-6; 3.5e-5	**0.0043**	2.8e-6	-2.5e-6; 8.0e-6	0.40
Splenium of corpus callosum	3.9	2.0e-2	**3.5e-2**	8.1e-6	-2.4e-6; 1.9e-5	0.16	5.2e-6	1.4e-6; 9.0e-6	**0.0044**
Cingulum hippocampus	11.6	9.0e-6	**1.1e-4**	8.2e-6	-2.4e-6; 1.9e-5	0.16	6.3e-6	2.5e-6; 1.0e-5	**5.0e-4**

* Adjusted mean difference, calculated using linear models, adjusted for age, sex, education, X-, Y-, and Z-position of the head in the scanner, head size, assessment centre, and year of imaging acquisition.

CI, confidence interval; P_{FDR}, Benjamini-Hochberg false discovery rate corrected P values; SE, standard error. P values in bold are statistically significant ($P < 0.05$).

For peer review only

Supplement 6. Cognitive outcome measures of chronic oral glucocorticoid users (n = 42) and chronic inhalation glucocorticoid users (n = 305) vs. controls

	**ANOVA**			**Oral GC vs. controls**			**Inhalation GC vs. controls**			**Participants with available data, n (%)**		
	**F value**	**P value**	**P_{FDR}**	**AMD***	**95% CI**	**P value**	**AMD***	**95% CI**	**P value**	**Oral GC**	**Inhalation GC**	**Controls**
**Trail making A**	0.41	0.66	0.84	0.12	-0.26; 0.51	0.69	-0.07	-0.24; 0.10	0.55	30 (71)	151 (50)	16419 (68)
**Trail making B**	0.28	0.75	0.84	-0.08	-0.47; 0.31	0.84	0.00	-0.17; 0.17	1.00	28 (67)	148 (49)	16071 (67)
**Symbol substitution**	0.35	0.70	0.84	-0.08	-0.45; 0.30	0.84	-0.05	-0.21; 0.11	0.71	30 (71)	151 (50)	16442 (68)

* Adjusted mean difference between patients and controls, expressed in Z scores. Calculated using linear models, adjusting for age, sex, and education. Trail making A, and trail making B were log transformed before generation of Z scores because they were non-normally distributed. Variables were transformed such that higher values indicate a better performance.

CI, confidence interval; GC, glucocorticoids; n, number; P_{FDR}, Benjamini-Hochberg false discovery rate corrected P values

For peer review only

Supplement 7. Self-reported frequency of mental health parameters in the past two weeks of patients using oral glucocorticoids (n = 222) or inhalation glucocorticoids (n = 557) and controls, presented as number of participants (%) per category

	**Oral GC (n=222)**	**Inhalation GC (n=557)**	**Controls (n=24106)**
**Depressed mood**			
Not at all	170 (77)	455 (82)	19940 (83)
Several days	39 (18)	77 (14)	3017 (13)
More than half of the days	6 (2.7)	8 (1.4)	296 (1.2)
Nearly every day	1 (0.5)	3 (0.5)	150 (0.6)
Missing	6 (2.7)	14 (2.5)	703 (2.9)
**Disinterest**			
Not at all	174 (78)	468 (84)	20536 (85)
Several days	34 (15)	61 (11)	2568 (11)
More than half of the days	3 (1.3)	7 (1.3)	292 (1.2)
Nearly every day	5 (2.3)	5 (0.9)	174 (0.7)
Missing	6 (2.7)	16 (2.9)	536 (2.2)
**Tenseness/restlessness**			
Not at all	162 (73)	437 (78)	19412 (81)
Several days	46 (21)	89 (16)	3630 (15)
More than half of the days	3 (1.3)	12 (2.2)	272 (1.1)
Nearly every day	5 (2.3)	5 (0.9)	126 (0.5)
Missing	6 (2.7)	14 (2.5)	666 (2.8)
**Tiredness/lethargy**			
Not at all	95 (43)	280 (50)	13792 (57)
Several days	91 (41)	221 (40)	8345 (35)
More than half of the days	9 (4.1)	32 (5.7)	815 (3.4)
Nearly every day	19 (8.6)	15 (2.7)	555 (2.3)
Missing	8 (3.6)	9 (1.6)	599 (2.5)

GC, glucocorticoids; n, number.

For peer review only

Supplement 8. Self-reported frequency of mental health parameters in the past two weeks of chronic oral glucocorticoid users (n = 42), chronic inhalation glucocorticoid users (n = 305) and controls, presented as number of participants (%) per category

	**Oral GC (n = 42)**	**Inhalation GC (n = 305)**	**Controls (n = 24106)**
**Depressed mood**			
Not at all	33 (79)	257 (84)	19940 (83)
Several days	6 (14)	35 (11)	3017 (13)
More than half of the days	0 (0)	3 (0.9)	296 (1.2)
Nearly every day	0 (0)	1 (0.3)	150 (0.6)
Missing	3 (7.1)	9 (3.0)	703 (2.9)
**Disinterest**			
Not at all	34 (81)	267 (88)	20536 (85)
Several days	6 (14)	30 (9.8)	2568 (11)
More than half of the days	0 (0)	1 (0.3)	292 (1.2)
Nearly every day	0 (0)	0 (0)	174 (0.7)
Missing	2 (4.8)	7 (2.3)	536 (2.2)
**Tenseness/restlessness**			
Not at all	30 (71)	245 (80)	19412 (81)
Several days	10 (24)	48 (16)	3630 (15)
More than half of the days	0 (0)	6 (2.0)	272 (1.1)
Nearly every day	0 (0)	1 (0.3)	126 (0.5)
Missing	2 (4.8)	5 (1.6)	666 (2.8)
**Tiredness/lethargy**			
Not at all	24 (57)	156 (51)	13792 (57)
Several days	12 (29)	121 (40)	8345 (35)
More than half of the days	2 (4.8)	14 (4.6)	815 (3.4)
Nearly every day	2 (4.8)	8 (2.6)	555 (2.3)
Missing	2 (4.8)	6 (2.0)	599 (2.5)

GC, glucocorticoids; n, number.

For peer review only

Supplement 9. Likelihood of experiencing mental health complaints in the past two weeks of chronic oral glucocorticoid users (n = 42) and chronic inhalation glucocorticoid users (n = 305) compared to controls

	Likelihood ratio test			Oral GC vs. controls			Inhalation GC vs. controls		
	X^2	P value	P_{FDR}	OR	95% CI	P value	OR	95% CI	P value
**Depression**	1.1	0.57	0.57	1.21	0.45; 2.73	0.67	0.85	0.59; 1.18	0.34
**Disinterest**	2.2	0.33	0.44	1.41	0.53; 3.17	0.44	0.79	0.53; 1.13	0.21
**Tenseness**	2.5	0.28	0.44	1.84	0.84; 3.68	0.10	1.05	0.78; 1.40	0.73
**Tiredness**	4.4	0.11	0.44	0.96	0.49; 1.84	0.91	1.28	1.01; 1.61	**0.0037**

Calculated using logistic regression analysis, adjusting for age, sex, and education. P values in bold are statistically significant after Bonferroni correction for family-wise error rate of two tests (P < 0.025).

CI, confidence interval; GC, glucocorticoids; OR, odds ratio; P_{FDR}, Benjamini-Hochberg false discovery rate corrected P values.

For peer review only

Supplement 10. Sensitivity analysis: Characteristics of included glucocorticoid users and controls (without exclusion of participants based on psychiatric, neurological, or endocrine history or medication use)

	Patients using oral GC (n = 312)	Patients using inhalation GC (n = 806)	Controls (n = 36310)	P value
**Sex: male, n (%)**	145 (46.5)	344 (42.7)	17041 (46.9)	0.057
**Age at time of scanning in years, mean (SD)**	66.1 (6.9)	62.8 (7.5)	63.7 (7.5)	3.6e-10
**Education level, n (%)**				0.37
College/University degree	143 (45.8)	407 (50.5)	17637 (48.6)	
A levels or equivalent	39 (12.5)	98 (12.2)	4392 (12.1)	
O levels/GCSE or equivalent	53 (17.0)	136 (16.9)	6400 (17.6)	
CSEs or equivalent	13 (4.2)	26 (3.2)	1372 (3.8)	
NVQ, HND, HNC, or equivalent	11 (3.5)	50 (6.2)	2142 (5.9)	
Other professional qualifications	21 (6.7)	45 (5.6)	1795 (4.9)	
None of the above	27 (8.7)	40 (5.0)	2208 (6.1)	
Missing	5 (1.6)	4 (0.5)	364 (1.0)	
**BMI in kg/m^2, mean (SD)**	26.7 (4.4)	27.1 (4.7)	26.5 (4.4)	2.2e-4
Number (%) missing	11 (3.5)	31 (3.8)	1932 (5.3)	
**Body fat percentage, mean (SD)**	31.9 (8.2)	32.6 (8.4)	31.1 (8.1)	5.5e-7
Number (%) missing	11 (3.5)	31 (3.8)	1942 (5.3)	
**Smoking status, n (%)**				0.096
Current	10 (3.2)	25 (3.1)	1231 (3.3)	
Previous	118 (37.8)	299 (37.1)	12063 (33.2)	
Never	181 (58.0)	477 (59.2)	22661 (62.4)	
Missing	3 (1.0)	5 (0.6)	355 (1.0)	

For peer review only

BMI, body mass index; GC, glucocorticoids; n, number; SD, standard deviation.

P values determined using analysis of variance (for continuous variables) and Pearson's Chi squared test (for categorical variables).

For peer review only

Supplement 11. Sensitivity analysis: Imaging parameters, presented as the adjusted mean difference of patients using oral glucocorticoids (n = 312) or inhalation glucocorticoids (n = 806) compared to controls (n = 36310) (without exclusion of participants based on psychiatric, neurological, or endocrine history or medication use)

	ANOVA			Oral GC vs. controls			Inhalation GC vs. controls		
	**F value**	**P value**	**P_{FDR}**	**AMD***	**95% CI**	**P value**	**AMD***	**95% CI**	**P value**
Volumetric measures									
Global volumes (in mm^3)									
Total brain volume	17.7	2.2e-8	**1.3e-7**	-3460	-9320; 2400	0.32	3535	-121; 7190	0.060
Grey matter volume	22.3	2.0e-10	**2.4e-9**	-2224	-5577; 1130	0.25	1454	-637; 3546	0.22
White matter volume	5.5	4.1e-3	**6.7e-3**	-1237	-5078; 2604	0.69	2080	-316; 4476	0.10
Peripheral cortex	24.6	2.0e-11	**4.4e-10**	-3318	-6330; -307	**0.028**	1172	-706; 3051	0.29
CSF volume	14.2	7.1e-7	**2.3e-6**	1220	-518; 2958	0.12	223	-861; 1307	0.65
Subcortical volumes (in mm^3)									
Accumbens	10.2	3.8e-5	**1.0e-4**	-8.9	-20.4; 2.7	0.16	-3.7	-10.9; 3.5	0.41
Caudate	4.5	1.1e-2	**1.7e-2**	58.6	13.8; 103.5	**0.0072**	-5.9	-33.9; 22.1	0.84
Pallidum	6.9	1.0e-3	**1.9e-3**	1.2	-24.5; 27.0	0.99	-16.2	-32.3; -0.2	**0.047**
Putamen	9.8	5.6e-5	**1.5e-4**	-33.8	-90.5; 22.9	0.32	-20.1	-55.5; 15.3	0.35
Thalamus	9.3	9.4e-5	**2.3e-4**	-19.9	-86.2; 46.5	0.72	-10.7	-52.1; 30.7	0.78
Regional grey matter volumes (in mm^3)									
Amygdala	21.0	7.8e-10	**7.0e-9**	-6.7	-30.4; 17.1	0.75	-21.7	-36.5; -6.8	**0.0023**
Caudate	12.3	4.7e-6	**1.4e-5**	149.6	66.9; 232.4	**1.0e-4**	42.9	-8.7; 94.5	0.12
Cerebellum	5.8	3.1e-3	**5.2e-3**	17.8	-19.4; 54.9	0.47	-2.9	-26.1; 20.3	0.93

For peer review only

Insular cortex	8.7	1.7e-4	**3.5e-4**	-42.1	-103.5; 19.4	0.23	8.0	-30.3; 46.3	0.84
Precuneal cortex	4.0	1.9e-2	**2.7e-2**	-9.7	-142.8; 123.4	0.97	-1.7	-84.7; 81.3	1.00
DTI measures									
Fractional anisotropy									
Global	15.5	1.8e-7	**9.4e-7**	-0.0031	-0.0055; -7.5e-4	**0.0066**	-0.0015	-0.0030; -4.9e-5	**0.041**
Body of corpus callosum	8.9	1.4e-4	**3.1e-4**	-0.0039	-0.0076; -0.0003	**0.032**	-0.0014	-0.0036; 8.9e-4	0.30
Genu of corpus callosum	15.2	2.5e-7	**1.1e-6**	-0.0056	-0.0097; -0.0014	**0.0055**	-0.0013	-0.0039; 0.0013	0.44
Cingulum cingulate	3.8	2.3e-2	**3.1e-2**	-0.0014	-0.0052; 0.0025	0.64	-0.0018	-0.0042; 5.9e-4	0.17
Mean diffusivity									
Global	24.5	2.4e-11	**4.4e-10**	6.6e-6	3.0e-6; 1.0e-5	**3.7e-5**	1.9e-6	-3.2e-7; 4.1e-6	5.7e-2
Body of corpus callosum	14.2	6.7e-7	**2.3e-6**	6.7e-6	1.9e-6; 1.1e-5	**0.0034**	4.0e-6	1.1e-6; 7.0e-6	**0.0048**
Genu of corpus callosum	17.9	1.7e-8	**1.2e-7**	8.0e-6	2.5e-6; 1.4e-5	**0.0023**	3.3e-6	-1.4e-7; 6.7e-6	0.0622
Splenium of corpus callosum	6.7	1.2e-3	**2.2e-3**	3.7e-6	-3.1e-7; 7.6e-6	0.076	4.0e-6	1.5e-6; 6.4e-6	**7.0e-4**
Cingulum cingulate	4.9	7.6e-3	**1.2e-2**	2.5e-6	-6.8e-7; 5.7e-6	0.15	2.2e-6	2.2e-7; 4.2e-6	**0.026**
Cingulum hippocampus	14.5	4.9e-7	**2.0e-6**	2.6e-6	-1.3e-6; 6.6e-6	0.25	4.5e-6	2.0e-6; 7.0e-6	**1.0e-4**
Uncinate fasciculus	7.3	6.6e-4	**1.3e-3**	4.0e-6	2.9e-7; 7.7e-6	**0.032**	1.6e-6	-7.5e-7; 3.9e-6	0.23

* Adjusted mean difference, calculated using linear models, adjusted for age, sex, education, X-, Y-, and Z-position of the head in the scanner, head size, assessment centre, and year of imaging acquisition.

P_{FDR}, Benjamini-Hochberg false discovery rate corrected P values; SE, standard error. P values in bold are statistically significant ($P < 0.05$).

Supplement 12. Sensitivity analysis: Cognitive outcome measures of oral glucocorticoid users (n = 312) and inhalation glucocorticoid users (n = 806) vs. controls (n = 36310) (without exclusion of participants based on psychiatric, neurological, or endocrine history or medication use)

	ANOVA			Oral GC vs. controls			Inhalation GC vs. controls			Participants with available data, n (%)		
	**F value**	**P value**	**P_{FDR}**	**AMD***	**95% CI**	**P value**	**AMD***	**95% CI**	**P value**	**Oral GC**	**Inhalation GC**	**Controls**
**Trail making A**	6.6	0.0014	**0.0028**	-0.11	-0.26; 0.03	0.16	0.020	-0.08; 0.12	0.86	206 (66)	422 (52)	24297 (67)
**Trail making B**	6.7	0.0013	**0.0028**	-0.12	-0.27; 0.02	0.10	-0.018	-0.12; 0.08	0.88	194 (62)	415 (51)	23273 (64)
**Symbol substitution**	9.7	6.2e-5	**0.00037**	-0.15	-0.29; -0.01	**0.029**	-0.061	-0.16; 0.04	0.28	203 (65)	423 (52)	24337 (67)

* Adjusted mean difference between patients and controls, expressed in Z scores. Calculated using linear models, adjusted for age, sex, and education. Trail making A, and trail making B were log transformed before generation of Z scores because they were non-normally distributed. Variables were transformed such that higher values indicate a better performance.

GC, glucocorticoids; P_{FDR}, Benjamini-Hochberg false discovery rate corrected P values.

For peer review only

Supplement 13. Sensitivity analysis: Self-reported frequency of mental health parameters in the past two weeks of patients using oral glucocorticoids (n = 312) or inhalation glucocorticoids (n = 806) and controls, presented as number of participants (%) per category (without exclusion of participants based on psychiatric, neurological, or endocrine history or medication use)

	Oral GC (n = 312)	Inhalation GC (n = 806)	Controls (n = 36310)
**Depressed mood**			
Not at all	240 (76.9)	620 (76.9)	29014 (80.0)
Several days	55 (17.6)	139 (17.2)	5197 (14.3)
More than half of the days	8 (2.6)	14 (1.7)	593 (1.6)
Nearly every day	2 (0.6)	14 (1.7)	360 (1.0)
Missing	7 (2.2)	19 (2.4)	1146 (3.2)
**Disinterest**			
Not at all	237 (76.0)	639 (79.3)	29916 (82.4)
Several days	55 (17.6)	118 (14.6)	4583 (12.6)
More than half of the days	8 (2.6)	17 (2.1)	604 (1.7)
Nearly every day	5 (1.6)	12 (1.5)	357 (1.0)
Missing	7 (2.2)	20 (2.5)	850 (2.3)
**Tenseness/restlessness**			
Not at all	221 (70.8)	588 (73.0)	28266 (77.8)
Several days	71 (22.8)	157 (19.5)	6113 (16.8)
More than half of the days	6 (1.9)	23 (2.9)	565 (1.6)
Nearly every day	6 (1.9)	16 (2.0)	313 (0.9)
Missing	8 (2.6)	22 (2.7)	1053 (2.9)
**Tiredness/lethargy**			
Not at all	125 (40.0)	366 (45.4)	19107 (52.6)
Several days	130 (41.7)	321 (39.8)	13373 (36.8)
More than half of the days	22 (7.1)	53 (6.6)	1533 (4.2)
Nearly every day	26 (8.3)	51 (6.3)	1358 (3.7)
Missing	9 (2.9)	15 (1.9)	939 (2.6)

GC, glucocorticoids; n, number.

For peer review only

Supplement 14. Sensitivity analysis: Likelihood of experiencing mental health complaints in the past two weeks of oral glucocorticoid users (n = 312) and inhalation glucocorticoid users (n = 806) compared to controls (without exclusion of participants based on psychiatric, neurological, or endocrine history or medication use)

	**Likelihood ratio test**			**Oral GC vs. controls**			**Inhalation GC vs. controls**		
	**X^2**	**P value**	**P_{FDR}**	**OR**	**95% CI**	**P value**	**OR**	**95% CI**	**P value**
**Depression**	11.1	0.0039	0.0039	1.44	1.08; 1.89	**0.010**	1.23	1.03; 1.46	**0.023**
**Disinterest**	17.8	1.4e-4	1.9e-04	1.73	1.31; 2.27	**8.5e-05**	1.21	1.00; 1.45	0.041
**Tenseness**	24.0	6.1e-06	1.2e-05	1.68	1.29; 2.16	**7.0e-05**	1.31	1.11; 1.54	**0.0014**
**Tiredness**	39.2	3.1e-09	1.2e-08	1.79	1.42; 2.27	**9.0e-07**	1.33	1.15; 1.53	**1.1e-4**

Calculated using logistic regression analysis, adjusting for age, sex, and education. P values in bold are statistically significant after Bonferroni correction for family-wise error rate of two tests ($P < 0.025$).

CI, confidence interval; GC, glucocorticoids; OR, odds ratio; P_{FDR}, Benjamini-Hochberg false discovery rate corrected P values.

For peer review only

Supplement 15. Sensitivity analysis: Imaging parameters, presented as the adjusted mean difference of patients using oral glucocorticoids (n = 222) or inhalation glucocorticoids (n = 557) compared to controls (n = 24106) (after exclusion of outlier values per group per variable)

	ANOVA			Oral GC vs. controls			Inhalation GC vs. controls		
	**F value**	**P value**	**P_{FDR}**	**AMD***	**95% CI**	**P value**	**AMD***	**95% CI**	**P value**
Volumetric measures									
Global volumes (in mm³)									
Total brain volume	16.0	1.1e-7	**4.6e-7**	-3991	-10852; 2869	0.33	3756	-565; 8076	0.10
Grey matter volume	28.8	3.4e-13	**6.1e-12**	-3143	-7081; 794	0.14	1120	-1337; 3576	0.50
White matter volume	5.4	4.6e-3	**7.1e-3**	-1861	-6349; 2626	0.55	2374	-454; 5203	0.11
Peripheral cortex	27.0	2.0e-12	**1.8e-11**	-4412	-7948; -876	**0.011**	1148	-1058; 3355	0.41
CSF volume	16.8	5.0e-8	**2.3e-7**	1437	-210; 3084	0.10	-449	-1492; 594	0.53
Subcortical volumes (in mm³)									
Accumbens	13.0	2.3e-6	**5.8e-6**	-15.6	-28.8; -2.3	**0.018**	-4.6	-13.0; 3.7	0.37
Caudate	4.7	8.8e-3	**1.1e-2**	69.4	18.4; 120.3	**0.0049**	4.5	-27.4; 36.3	0.92
Hippocampus	5.4	4.7e-3	**7.1e-3**	-17.1	-71.2; 37.0	0.70	-17	-51.3; 17.3	0.44
Pallidum	4.9	7.4e-3	**9.8e-3**	5.7	-20.5; 31.8	0.83	-9.8	-26.3; 6.7	0.32
Putamen	13.7	1.1e-6	**3.4e-6**	-63	-127.1; 1.0	0.055	-19.9	-59.7; 20.0	0.44
Thalamus	10.0	4.6e-5	**8.7e-5**	-25.6	-98.2; 46.9	0.64	-0.6	-46.2; 45.1	1.00
Regional grey matter volumes (in mm³)									
Amygdala	28.3	5.1e-13	**6.1e-12**	-17.2	-43.8; 9.4	0.26	-22.6	-39.3; -5.9	**0.01**
Caudate	12.6	3.5e-6	**8.4e-6**	138.1	67.7; 208.6	**<0.0001**	15.1	-28.8; 59.1	0.66
Cerebellum	10.3	3.3e-5	**6.6e-5**	-1.1	-42.8; 40.6	1.00	-6.6	-32.5; 19.3	0.78

For peer review only

Cingulate gyrus, anterior	3.9	2.1e-2	**2.6e-2**	110.5	-7.8; 229.0	0.071	27.1	-47.9; 102.0	0.63
Hippocampus	3.3	3.9e-2	**4.6e-2**	24.3	-22.4; 70.9	0.41	2.4	-27.0; 31.8	0.97
Insular cortex	13.1	2.0e-6	**5.5e-6**	-74.8	-143.2; -6.4	**0.029**	8.7	-34.1; 51.4	0.85
Precuneal cortex	5.2	5.4e-3	**7.5e-3**	-60.1	-213.6; 93.3	0.59	0.0	-95.6; 95.6	1.00
DTI measures									
Fractional anisotropy									
Global	22.7	1.4e-10	**1.0e-9**	-0.0043	-0.0067; -0.0018	**2.0e-4**	-0.0019	-0.0035; -3.4e-4	**0.013**
Body of corpus callosum	11.4	1.1e-5	**2.5e-5**	-0.0048	-0.0086; -0.0010	**0.0097**	-0.0021	-0.0045; 3.4e-4	0.11
Genu of corpus callosum	15.3	2.3e-7	**8.4e-7**	-0.0059	-0.010; -0.0016	**0.0048**	-0.0017	-0.0044; 0.0010	0.28
Cingulum cingulate	6.5	1.5e-3	**2.5e-3**	-0.0022	-0.0065; 0.0021	0.42	-0.0026	-0.0053; 9.7e-5	0.061
Cingulum hippocampus	7.5	5.7e-4	**9.7e-4**	-0.00012	-0.0046; 0.0044	1.00	-0.0036	-0.0064; -7.5e-4	**0.010**
Mean diffusivity									
Global	29.1	2.4e-13	**6.1e-12**	7.1e-6	3.7e-6; 1.1e-5	**<0.0001**	2.5e-6	3.1e-7; 4.7e-6	**0.022**
Body of corpus callosum	17.1	3.6e-8	**1.9e-7**	7.5e-6	2.8e-6; 1.2e-5	**7.0e-4**	3.7e-6	6.9e-7; 6.6e-6	**0.012**
Genu of corpus callosum	21.6	4.3e-10	**2.6e-9**	9.5e-6	3.9e-6; 1.5e-5	**3.0e-4**	3.6e-6	2.9e-8; 7.1e-6	**0.048**
Splenium of corpus callosum	9.9	5.2e-5	**9.4e-5**	4.6e-6	7.3e-7; 8.4e-6	**0.016**	4.2e-6	1.8e-6; 6.7e-6	**2.0e-4**
Cingulum cingulate	5.3	5.2e-3	**7.5e-3**	2.6e-6	-9.4e-7; 6.1e-6	0.19	2.6e-6	3.6e-7; 4.8e-6	**0.019**
Cingulum hippocampus	13.7	1.1e-6	**3.4e-6**	4.4e-6	2.5e-7; 8.6e-6	**0.035**	4.3e-6	1.6e-6; 6.9e-6	**6.0e-4**
Uncinate fasciculus	11.3	1.2e-5	**2.5e-5**	5.8e-6	1.9e-6; 9.7e-6	**0.0018**	2.4e-6	-8.8e-8; 4.8e-6	0.061

* Adjusted mean difference, calculated using linear models, adjusted for age, sex, education, X-, Y-, and Z-position of the head in the scanner, head size, assessment centre, and year of imaging acquisition.

P_{FDR}, Benjamini-Hochberg false discovery rate corrected P values; SE, standard error. P values in bold are statistically significant ($P < 0.05$).

For peer review only

Supplement 16. Cognitive outcome measures of oral glucocorticoid users (n = 222) and inhalation glucocorticoid users (n = 557) vs. controls (after exclusion of outlier values per group per variable)

	ANOVA			Oral GC vs. controls			Inhalation GC vs. controls			Participants with available data, n (%)		
	F value	P value	P_{FDR}	AMD*	95% CI	P value	AMD*	95% CI	P value	Oral GC	Inhalation GC	Controls
**Trail making A**	5.2	0.0057	**0.011**	-0.10	-0.25; 0.05	0.25	-0.018	-0.12; 0.09	0.88	143 (64)	286 (51)	15996 (66)
**Trail making B**	9.6	6.8e-5	**2.0e-4**	-0.16	-0.32; -0.01	**0.038**	-0.064	-0.17; 0.04	0.31	137 (62)	289 (52)	15733 (65)
**Symbol substitution**	11.6	8.9e-6	**5.3e-5**	-0.18	-0.34; -0.02	**0.021**	-0.046	-0.16; 0.06	0.55	141 (64)	295 (53)	16270 (67)

* Adjusted mean difference between patients and controls, expressed in Z scores. Calculated using linear models, adjusted for age, sex, and education. Trail making A, and trail making B were log transformed before generation of Z scores because they were non-normally distributed. Variables were transformed such that higher values indicate a better performance.

GC, glucocorticoids; P_{FDR}, Benjamini-Hochberg false discovery rate corrected P values.

For peer review only

Supplement 17. STROBE Statement – Checklist of items that should be included in reports of cohort studies

	Item No	Recommendation	Where to be found
**Title and abstract**	1	(*a*) Indicate the study's design with a commonly used term in the title or the abstract	Abstract: Design (p.2)
		(*b*) Provide in the abstract an informative and balanced summary of what was done and what was found	Abstract: Main outcome measures, Results (p.2)
**Introduction**			
Background/ rationale	2	Explain the scientific background and rationale for the investigation being reported	Introduction (p.4)
Objectives	3	State specific objectives, including any prespecified hypotheses	Introduction (p.4)
**Methods**			
Study design	4	Present key elements of study design early in the paper	Study design (p.5)
Setting	5	Describe the setting, locations, and relevant dates, including periods of recruitment, exposure, follow-up, and data collection	Study design, Data collection (p.5)
Participants	6	(*a*) Give the eligibility criteria, and the sources and methods of selection of participants. Describe methods of follow-up	Participants (pp.5-6)
		(*b*) For matched studies, give matching criteria and number of exposed and unexposed	*Not applicable*
Variables	7	Clearly define all outcomes, exposures, predictors, potential confounders, and effect modifiers. Give diagnostic criteria, if applicable	Data collection, Imaging data, Cognitive and Emotional data, Statistical analysis (pp.5-9)
Data sources/ measurement	8	For each variable of interest, give sources of data and details of methods of assessment (measurement). Describe comparability of assessment methods if there is more than one group	Data collection, Imaging data, Cognitive and Emotional data (pp.5-7)
Bias	9	Describe any efforts to address potential sources of bias	Statistical analysis (pp.7-9)
Study size	10	Explain how the study size was arrived at	Participants (pp.5-6)
Quantitative variables	11	Explain how quantitative variables were handled in the analyses. If applicable, describe which groupings were chosen and why	Statistical analysis (pp.7-9)
Statistical methods	12	(*a*) Describe all statistical methods, including those used to control for confounding	Statistical analysis (pp.7-9)
		(*b*) Describe any methods used to examine subgroups and interactions	
		(*c*) Explain how missing data were addressed	
		(*d*) If applicable, explain how loss to follow-up was addressed	
		(*e*) Describe any sensitivity analyses	

For peer review only

**Results**			
Participants	13	(a) Report numbers of individuals at each stage of study – e.g. numbers potentially eligible, examined for eligibility, confirmed eligible, included in the study, completing follow-up, and analyzed	Demographic characteristics (p.10) and Figure 1
		(b) Give reasons for non-participation at each stage	
		(c) Consider use of a flow diagram	
Descriptive data	14	(a) Give characteristics of study participants (e.g. demographic, clinical, social) and information on exposures and potential confounders	Demographic characteristics (p.10) and Table 1
		(b) Indicate number of participants with missing data for each variable of interest	
		(c) Summarize follow-up time (e.g., average and total amount)	*Not applicable*
Outcome data	15*	Report numbers of outcome events or summary measures over time	Results (pp.12-18)
Main results	16	(*a*) Give unadjusted estimates and, if applicable, confounder-adjusted estimates and their precision (e.g., 95% confidence interval). Make clear which confounders were adjusted for and why they were included	Results (pp.12-18), Tables 2-4, Supplements
		(*b*) Report category boundaries when continuous variables were categorized	Statistical analysis (p.8)
		(*c*) If relevant, consider translating estimates of relative risk into absolute risk for a meaningful time period	*Not applicable*
Other analyses	17	Report other analyses done – e.g. analyses of subgroups and interactions, and sensitivity analyses	Results (p.19), Supplements
**Discussion**			
Key results	18	Summarize key results with reference to study objectives	Discussion (pp.20-21)
Limitations	19	Discuss limitations of the study, taking into account sources of potential bias or imprecision. Discuss both direction and magnitude of any potential bias	Strengths and limitations (pp.22-23)
Interpretation	20	Give a cautious overall interpretation of results considering objectives, limitations, multiplicity of analyses, results from similar studies, and other relevant evidence	Findings in context, Potential consequences and implications (pp.20-22)
Generalizability	21	Discuss the generalizability (external validity) of the study results	Strengths and limitations (pp.22-23)
**Other information**			
Funding	22	Give the source of funding and the role of the funders for the present study and, if applicable, for the original study on which the present article is based	Funding (p.24)

For peer review only

STROBE Statement – Checklist of items that should be included in reports of cohort studies

	Item No	Recommendation	Where to be found
**Title and abstract**	1	(*a*) Indicate the study's design with a commonly used term in the title or the abstract	Abstract: Design (p.2)
		(*b*) Provide in the abstract an informative and balanced summary of what was done and what was found	Abstract: Main outcome measures, Results (p.2)
**Introduction**			
Background/ rationale	2	Explain the scientific background and rationale for the investigation being reported	Introduction (p.4)
Objectives	3	State specific objectives, including any prespecified hypotheses	Introduction (p.4)
**Methods**			
Study design	4	Present key elements of study design early in the paper	Study design (p.5)
Setting	5	Describe the setting, locations, and relevant dates, including periods of recruitment, exposure, follow-up, and data collection	Study design, Data collection (p.5)
Participants	6	(*a*) Give the eligibility criteria, and the sources and methods of selection of participants. Describe methods of follow-up	Participants (pp.5-6)
		(*b*) For matched studies, give matching criteria and number of exposed and unexposed	*Not applicable*
Variables	7	Clearly define all outcomes, exposures, predictors, potential confounders, and effect modifiers. Give diagnostic criteria, if applicable	Data collection, Imaging data, Cognitive and Emotional data, Statistical analysis (pp.5-9)
Data sources/ measurement	8	For each variable of interest, give sources of data and details of methods of assessment (measurement). Describe comparability of assessment methods if there is more than one group	Data collection, Imaging data, Cognitive and Emotional data (pp.5-7)
Bias	9	Describe any efforts to address potential sources of bias	Statistical analysis (pp.7-9)
Study size	10	Explain how the study size was arrived at	Participants (pp.5-6)
Quantitative variables	11	Explain how quantitative variables were handled in the analyses. If applicable, describe which groupings were chosen and why	Statistical analysis (pp.7-9)
Statistical methods	12	(*a*) Describe all statistical methods, including those used to control for confounding	Statistical analysis (pp.7-9)
		(*b*) Describe any methods used to examine subgroups and interactions	
		(*c*) Explain how missing data were addressed	
		(*d*) If applicable, explain how loss to follow-up was addressed	
		(*e*) Describe any sensitivity analyses	

For peer review only

**Results**			
Participants	13	(a) Report numbers of individuals at each stage of study – e.g. numbers potentially eligible, examined for eligibility, confirmed eligible, included in the study, completing follow-up, and analyzed	Demographic characteristics (p.10) and Figure 1
		(b) Give reasons for non-participation at each stage	
		(c) Consider use of a flow diagram	
Descriptive data	14	(a) Give characteristics of study participants (e.g. demographic, clinical, social) and information on exposures and potential confounders	Demographic characteristics (p.10) and Table 1
		(b) Indicate number of participants with missing data for each variable of interest	
		(c) Summarize follow-up time (e.g., average and total amount)	*Not applicable*
Outcome data	15*	Report numbers of outcome events or summary measures over time	Results (pp.12-18)
Main results	16	(*a*) Give unadjusted estimates and, if applicable, confounder-adjusted estimates and their precision (e.g., 95% confidence interval). Make clear which confounders were adjusted for and why they were included	Results (pp.12-18), Tables 2-4, Supplements
		(*b*) Report category boundaries when continuous variables were categorized	Statistical analysis (p.8)
		(*c*) If relevant, consider translating estimates of relative risk into absolute risk for a meaningful time period	*Not applicable*
Other analyses	17	Report other analyses done – e.g. analyses of subgroups and interactions, and sensitivity analyses	Results (p.19), Supplements
**Discussion**			
Key results	18	Summarize key results with reference to study objectives	Discussion (pp.20-21)
Limitations	19	Discuss limitations of the study, taking into account sources of potential bias or imprecision. Discuss both direction and magnitude of any potential bias	Strengths and limitations (pp.22-23)
Interpretation	20	Give a cautious overall interpretation of results considering objectives, limitations, multiplicity of analyses, results from similar studies, and other relevant evidence	Findings in context, Potential consequences and implications (pp.20-22)
Generalizability	21	Discuss the generalizability (external validity) of the study results	Strengths and limitations (pp.22-23)
**Other information**			
Funding	22	Give the source of funding and the role of the funders for the present study and, if applicable, for the original study on which the present article is based	Funding (p.24)

For peer review only

Association between use of systemic and inhaled glucocorticoids and changes in brain volume and white matter microstructure: a cross-sectional study using data from the UK Biobank

Journal:	*BMJ Open*
Manuscript ID	bmjopen-2022-062446.R1
Article Type:	Original research
Date Submitted by the Author:	17-Jun-2022

Complete List of Authors:	van der Meulen, Merel; Leiden University Medical Center, Department of Medicine, division of Endocrinology<br>Amaya, Jorge Miguel; Leiden University Medical Center, Department of Medicine, division of Endocrinology<br>Dekkers, Olaf; Leiden University Medical Center, Department of Medicine, division of Endocrinology<br>Meijer, Onno C.; Leiden University Medical Center, Department of Medicine, division of Endocrinology

**Primary Subject Heading**:	Diabetes and endocrinology
Secondary Subject Heading:	Mental health, Pharmacology and therapeutics, Radiology and imaging
Keywords:	Magnetic resonance imaging < RADIOLOGY & IMAGING, Anxiety disorders < PSYCHIATRY, DIABETES & ENDOCRINOLOGY, INTERNAL MEDICINE, MENTAL HEALTH, Depression & mood disorders < PSYCHIATRY

SCHOLARONE™
Manuscripts

BMJ

I, the Submitting Author has the right to grant and does grant on behalf of all authors of the Work (as defined in the below author licence), an exclusive licence and/or a non-exclusive licence for contributions from authors who are: i) UK Crown employees; ii) where BMJ has agreed a CC-BY licence shall apply, and/or iii) in accordance with the terms applicable for US Federal Government officers or employees acting as part of their official duties; on a worldwide, perpetual, irrevocable, royalty-free basis to BMJ Publishing Group Ltd ("BMJ") its licensees and where the relevant Journal is co-owned by BMJ to the co-owners of the Journal, to publish the Work in this journal and any other BMJ products and to exploit all rights, as set out in our licence.

The Submitting Author accepts and understands that any supply made under these terms is made by BMJ to the Submitting Author unless you are acting as an employee on behalf of your employer or a postgraduate student of an affiliated institution which is paying any applicable article publishing charge ("APC") for Open Access articles. Where the Submitting Author wishes to make the Work available on an Open Access basis (and intends to pay the relevant APC), the terms of reuse of such Open Access shall be governed by a Creative Commons licence – details of these licences and which Creative Commons licence will apply to this Work are set out in our licence referred to above.

Other than as permitted in any relevant BMJ Author's Self Archiving Policies, I confirm this Work has not been accepted for publication elsewhere, is not being considered for publication elsewhere and does not duplicate material already published. I confirm all authors consent to publication of this Work and authorise the granting of this licence.

For peer review only

Association between use of systemic and inhaled glucocorticoids and changes in brain volume and white matter microstructure: a cross-sectional study using data from the UK Biobank

Merel van der Meulen (0000-0002-0001-4408)[1], Jorge Miguel Amaya[1], Olaf M. Dekkers[1], Onno C. Meijer (0000-0002-8394-6859)[1]

[1] Department of Medicine, division of Endocrinology, Leiden University Medical Center, Leiden, the Netherlands

Merel van der Meulen: PhD candidate
Jorge Miguel Amaya: PhD candidate
Olaf M. Dekkers: full professor
Onno C. Meijer: full professor

Correspondence to: Merel van der Meulen, m.vandermeulen@lumc.nl, Department of Medicine, division of Endocrinology, Leiden University Medical Center, Leiden, the Netherlands

Tables: 4
Figures: 3
Word count: 5801

For peer review only

Abstract

Objective: To test the hypothesis that systemic and inhaled glucocorticoid use is associated with changes in grey matter volume (GMV) and white matter microstructure.

Design: Cross-sectional study.

Setting: UK Biobank, a prospective population-based cohort study of adults recruited in the UK between 2006 and 2010.

Participants: After exclusion based on neurological, psychiatric, or endocrinological history, and use of psychotropic medication, 222 systemic glucocorticoid users, 557 inhaled glucocorticoid users, and 24,106 controls with available T1 and diffusion MRI data were included.

Main outcome measures: Primary outcomes were differences in 22 volumetric and 14 diffusion imaging parameters between glucocorticoid users and controls, determined using linear regression analyses adjusted for potential confounders. Secondary outcomes included cognitive functioning (six tests) and emotional symptoms (four questions).

Results: Both systemic and inhaled glucocorticoid use were associated with reduced white matter integrity (lower fractional anisotropy (FA) and higher mean diffusivity (MD)) compared with controls, with larger effect sizes in systemic users (FA: adjusted mean difference (AMD)=-3.7e-3, -95% confidence interval (CI)=-6.4e-3 to 1.0e-3; MD: AMD=7.2e-6, 95%CI=3.2e-6 to 1.1e-5) than inhaled users (FA: AMD=-2.3e-3, 95%CI=-4.0e-3 to -5.7e-4; MD: AMD=2.7e-6, 95%CI=1.7e-7 to 5.2e-6). Systemic use was also associated with larger caudate GMV (AMD=178.7 mm^3, 95%CI=82.2 to 275.0), while inhaled users had smaller amygdala GMV (AMD=-23.9 mm^3, 95%CI=-41.5 to -6.2) than controls. As for secondary outcomes, systemic users performed worse on the symbol digit substitution task (AMD=-0.17 SD, 95%CI=-0.34 to -0.01), and reported more depressive symptoms (OR=1.76, 95%CI=1.25-2.43), disinterest (OR=1.84, 95%CI=1.29-2.56), tenseness/restlessness (OR=1.78, 95%CI=1.29-2.41), and tiredness/lethargy (OR=1.90, 95%CI=1.45-2.50) compared with controls. Inhaled users only reported more tiredness/lethargy (OR=1.35, 95%CI=1.14-1.60).

Conclusions: Both systemic and inhaled glucocorticoid use are associated with decreased white matter integrity and limited changes in GMV. This association may contribute to the neuropsychiatric side effects of glucocorticoid medication, especially with chronic use.

For peer review only

For peer review only - http://bmjopen.bmj.com/site/about/guidelines.xhtml

Strengths and limitations of this study

- To the best of our knowledge, this is the largest study to date assessing the association between glucocorticoid use and brain structure, and the first to investigate these associations in inhaled glucocorticoid users.
- Relatively strict exclusion criteria were used to limit the potential confounding that may arise in observational cohort studies.
- However, the cross-sectional nature of this study precludes formal conclusions on causality.
- Dose and duration of medication use were not available in the UK Biobank, making thorough analyses on dose-dependent or duration-dependent associations impossible.

For peer review only

For peer review only - http://bmjopen.bmj.com/site/about/guidelines.xhtml

Introduction

Due to their immunosuppressive properties, glucocorticoids are among the most prescribed drugs on the market, with an estimated annual prevalence of systemic glucocorticoid use between 0.5% and 3% [1-5]. Although efficacious, both systemic and local (especially inhaled) glucocorticoids are associated with many potentially serious metabolic, cardiovascular, and musculoskeletal side effects [6-9]. Besides these physical side effects, the use of synthetic glucocorticoids is also associated with neuropsychiatric symptoms and disorders, including depression, mania, delirium, and even a seven-fold increased suicide (attempt) rate [10, 11]. In addition, on an anatomical level, both preclinical and clinical studies have shown long-lasting effects of glucocorticoid overexposure on the brain. In patients with chronic endogenous glucocorticoid excess due to a pituitary tumour (Cushing disease), it has been established that long-term glucocorticoid excess is associated with global cerebral atrophy [12-18] and decreased cortical thickness and grey matter volumes in specific brain regions [13, 18-26]. Some of these effects were detected even after ten years of biochemical remission [22, 23]. Moreover, a few small studies have shown volumetric reductions in specific brain regions, including the hippocampus and amygdala, [27-31] in patients using chronic and/or high-dose synthetic systemic glucocorticoids. Besides these structural abnormalities, several studies in animal models and patients with Cushing disease have also demonstrated widespread reductions in white matter integrity throughout the brain [32-36]. In humans, this was studied using diffusion tensor imaging (DTI), showing globally decreased fractional anisotropy (FA), which represents the directionality of water diffusion through the brain and is a marker of microstructural architecture [37], and increased mean diffusivity (MD) [32-35], which represents an increase in water diffusion in all directions and is associated with disease processes such as inflammation and oedema [37].

However, most clinical studies investigating the effects of glucocorticoid overexposure on brain structure have been performed in small, selected populations with chronic glucocorticoid excess due to Cushing disease or systemic glucocorticoid use. It remains unknown whether these associations can also be observed in a broader sample of people using glucocorticoids, including inhaled glucocorticoids. We therefore used data from the UK Biobank, a large population-based cohort study, to investigate whether, at a population level, differences in brain volumes and white matter microstructure could be detected between systemic or inhaled glucocorticoid users and non-users. As secondary outcomes, we also assessed potential differences in cognitive and emotional functioning. Based on previous literature, we hypothesized that glucocorticoid use would be associated with decreased grey matter volumes in the limbic system and hippocampus, a widespread reduction in FA and increase in MD throughout the brain, and poorer cognitive and emotional outcomes.

For peer review only

Methods

Study design

The UK Biobank is a large population-based prospective cohort, comprising over 500,000 participants aged 40-69 years at the time of recruitment (between 2006 and 2010) [38]. The protocol for the UK Biobank was approved by the North-West Multi-Centre Research Ethics Committee and all participants provided written informed consent for collection, storage, and use of their data. Data for the present study were obtained under application number 59004.

Data collection

Data were collected at the assessment centres and during an online follow-up. Data used for this study included data on demographic characteristics, health and medical history, brain imaging, cognitive and emotional functioning, and body composition. Data on demographic characteristics, cognition, and emotional functioning were collected using a touch screen device at the assessment centres. If patients had indicated that they did not want to answer a question on one or more of these characteristics, we coded this as missing. Data on health and medical history, including medication use, were collected using the touch screen device and a verbal interview (self-reported data), but also using hospital episode statistics (HES). Body composition was measured using body impedance on a Tanita BC418MA body composition analyser as described in the UK Biobank documentation [39]. The imaging acquisition is described in more detail below.

Participants

For the analysis presented in this study, we selected participants who

1. Had both T1-weighted magnetic resonance imaging (MRI) and diffusion tensor imaging (DTI) data available at the same imaging visit;
2. Did not have a history of psychiatric disease based on self-reported data or HES data. However, we did include the psychiatric diseases most commonly associated with glucocorticoid use based on previous literature (anxiety, depression, mania, and delirium) [10] as we did not want to exclude patients based on potentially glucocorticoid-related outcomes;
3. Did not use psychotropic medication;
4. And did not have any neurological condition based on self-reported or HES data.

Individuals who met these criteria and used oral or parenteral glucocorticoids at the time of imaging were included in the systemic glucocorticoid patient group (n = 222), and individuals who met these criteria and used inhaled glucocorticoids (but no systemic glucocorticoids) at the time of imaging were included in the inhaled glucocorticoid group (n = 557). Among the patients using systemic glucocorticoids, 14 were also using inhaled glucocorticoids. Individuals who met these criteria but

For peer review only - http://bmjopen.bmj.com/site/about/guidelines.xhtml

For peer review only

had not used systemic or inhaled glucocorticoids at any timepoint (before and including the imaging visit) and did not have any endocrinological disorder according to self-reported or HES data, were included in the control group (n = 24,106). A flowchart of patient selection is presented in Figure 1, and Supplement 1 provides a list of all Biobank UK field codes that were used as inclusion or exclusion criteria.

Imaging data

Our study made use of imaging-derived phenotypes (IDPs) generated by an image-processing pipeline developed and run on behalf of the UK Biobank. Details on the brain imaging acquisition protocols, imaging processing and quality control, and generation of IDPs are provided by the UK Biobank [40, 41]. In short, all imaging was performed on a standard Siemens Skyra 3 Tesla scanner with a standard Siemens 32-channel radiofrequency receiver head coil. T1-weighted imaging was performed using a three-dimensional magnetization-prepared rapid acquisition with gradient echo sequence (3D MPRAGE) in the sagittal plane (voxel 1x1x1 mm; field-of-view 208x256x256 matrix). T1-weighted data were segmented using FAST (FMRIB's Automated Segmentation Tool [42]), to obtain volumes of cerebrospinal fluid (CSF), grey matter, and white matter, and to generate grey matter IDPs in 139 regions of interest (ROI). Subcortical structures were modelled using FIRST (FMRIB's Integrated Registration and Segmentation Tool [43]). For the present study, the mean volume of each bilateral structure was calculated over the two hemispheres, and the total cerebellar volume was calculated by adding up the volumes of all cerebellar lobules.

Diffusion imaging was performed using a standard Stejskal-Tanner pulse sequence to acquire 50 distinct diffusion-encoding directions for two diffusion-weighted shells with b values of 1000 and 2000 s/mm^2 (voxel 2x2x2 mm; field-of-view 104x104x72 matrix). The b = 1000 s/mm^2 data were fed into the diffusion-tensor-imaging (DTI) fitting tool (DTIFIT), which created DTI outputs including fractional anisotropy (FA) and mean diffusivity (MD). These outputs were then aligned to a standard-space white-matter skeleton using TBSS (Tract-Based Spatial Statistics [44]), and were averaged across a set of 48 standard-space tract masks defined by the John Hopkins University White Matter Atlas[45]. For the present study, the mean FA and MD of each bilateral structure of interest were calculated over the two hemispheres. Moreover, global FA and MD measures were calculated by averaging these metrics over all white matter tracts per individual. Grey matter FA or MD were not available in the UK Biobank and are therefore not included in the global FA and MD.

Cognitive and emotional data

At the assessment centres, participants also completed a series of cognitive tests and questionnaires on a touch screen. For these analyses, six cognitive tasks were selected: reaction time (to assess

For peer review only - http://bmjopen.bmj.com/site/about/guidelines.xhtml

simple processing speed; expressed as mean time to correctly identify matches), trail making A and B (to test visual attention; expressed as the duration to complete the numeric (A) or alphanumeric (B) path), fluid intelligence (to test reasoning and problem solving; expressed as a fluid intelligence score, which is the number of correct answers given to 13 questions), symbol digit substitution (to assess complex processing speed; expressed as the number of symbol digit matches made correctly within two minutes, with no maximum), and digit span (to test numeric working memory; expressed as the maximum digits remembered correctly, with a maximum of 12). For fluid intelligence, symbol digit substitution, and digit span tests, higher scores represent a better cognitive performance, while for reaction time, and trail making A and B, higher scores represent a worse cognitive performance.

Moreover, we analysed four mental health questionnaire items that specifically asked about the participant's situation in the previous two weeks, in which the glucocorticoid users were likely already using glucocorticoid medication. These questions included the frequency of a depressed mood, disinterest, tenseness/restlessness, and tiredness/lethargy in the past two weeks, and were answered using categorical answer options ('Never', 'Several days', 'More than half of the days', or 'Nearly every day'). The entire questionnaire can be found via: https://biobank.ndph.ox.ac.uk/ukb/ukb/docs/TouchscreenQuestionsMainFinal.pdf.

Statistical analysis

Demographic characteristics were presented as mean and standard deviation (SD) or number and percentage and were compared across the three groups using analysis of variance (ANOVA) or chi squared tests, respectively.

The primary outcomes of this study were the differences in imaging parameters between glucocorticoid users and controls for a selection of ROIs (22 volumetric parameters, 14 diffusion parameters), that have previously been shown to be affected by long-term glucocorticoid exposure (see Supplement 2). As secondary outcomes, potential differences in cognitive and emotional outcomes between glucocorticoid users and controls were assessed.

The statistical analysis was performed in a stepwise approach, which is visualized in Figure 2. For the imaging and cognitive outcomes, multivariable linear regression models were used. The assumption of normality of the residuals was assessed using quantile-quantile (Q-Q) plots and homogeneity of variance across the groups was tested using Levene's test and was visually assessed using scatter plots. Subsequently, ANOVA was used to assess whether any differences in outcome parameters existed between systemic glucocorticoid users, inhaled glucocorticoid users, and controls. To account for multiple testing, P values were adjusted using the Benjamini-Hochberg false discovery rate (FDR) method, for the number of comparisons tested (i.e., 36 for imaging variables, 6 for cognitive variables). For those parameters with P values < 0.05 after FDR correction, post-hoc

For peer review only - http://bmjopen.bmj.com/site/about/guidelines.xhtml

Dunnett tests were used to make pairwise comparisons between systemic glucocorticoid users vs. controls, and inhaled glucocorticoid users vs. controls.

For the multivariable linear models of the imaging parameters, covariates included age, sex, education, a measure of head size (the volumetric scaling from T1 image to standard space, corresponding to the inverse of head size), measures of head position (X-, Y-, and Z-position of the head in the scanner, and table position), assessment centre, and year of imaging acquisition. This selection was based on recommendations by the UK Biobank [46], in addition to variables that potentially meet the criteria of a confounder for this study. Because fewer than 1% of the participants had missing values for the covariates, complete case analysis was performed for the analysis of these primary outcomes, and all subsequent analyses. We considered that the very limited missing covariate data did not justify the intrinsic uncertainty that would come with imputation.

For the cognitive outcomes, variables with non-normally distributed residuals (reaction time, trail making A, trail making B) were normalized using log transformation. All cognitive outcomes were transformed such that higher values indicate better performance, and then converted into Z scores. The linear models of the cognitive outcomes were adjusted for age, sex, and education.

Since the emotional outcome parameters were categorical, logistic models were used, adjusted for age, sex, and education. Per symptom, the participants who reported a frequency of 'Several days', 'More than half of the days', or 'Nearly every day' were grouped together and were compared with participants who replied 'Never'. The likelihood ratio test was performed to determine whether the proportion of patients experiencing a mental health complaint in the past two weeks differed between the three groups. For those parameters with a statistically significant difference after FDR correction (for 4 comparisons), the odds ratio (OR) of experiencing a mental health complaint in the past two weeks was calculated for each glucocorticoid user group compared with controls. P values pertaining to the ORs were Bonferroni-corrected for multiple testing.

Use of glucocorticoids is associated with weight gain and in particular with an increased body fat percentage [7], which has been reported to affect brain volume and white matter microstructure [47]. Therefore, mediation analysis was performed to test whether the association between glucocorticoid use and brain volume and white matter microstructure were mediated by body fat percentage (as measured by body impedance). For this analysis, all three significantly different volumetric outcomes, and the two (significantly different) global diffusion imaging parameters were considered. The mediation analysis was performed using the *mediation* package, with 1000 simulations and including the same covariates for the imaging parameters as above.

Since the doses and duration of medication use are unknown in the UK Biobank, we were unable to perform subgroup analyses based on dose or duration of glucocorticoid use. Because

For peer review only - http://bmjopen.bmj.com/site/about/guidelines.xhtml

inhaled glucocorticoids are expected to cause, on average, lower systemic concentrations of glucocorticoids than orally or parenterally administered glucocorticoids [48], the inhaled glucocorticoid users likely represent a group of patients exposed to lower systemic concentrations than patients using systemic glucocorticoids and might show less pronounced effects of glucocorticoids on brain parameters. This may give an indication of a dose-dependent effect of glucocorticoids on the brain. In addition, to assess whether we could identify potential duration-dependent or cumulative dose-dependent associations of glucocorticoid use with brain parameters, we performed an additional analysis in the subgroups of glucocorticoid users who reported using glucocorticoids at two different visits (before and including the imaging visit) and therefore likely represent a group of chronic or repeated glucocorticoid users. Since the low number of participants in this group expectedly resulted in a lower power, we performed the post-hoc tests for these subgroups not only on those parameters that were statistically significant in the ANOVA, but on those parameters assessed by post-hoc tests in the main analysis, because this allowed us to gain insight into the difference in effect size compared with the main analysis.

Lastly, to assess whether outlier values, possibly resulting from poor data quality or processing problems, affected the imaging or cognitive outcomes, the analyses were repeated while excluding outlier values of all outcome parameters (per outcome per study group), defined as more than 1.5 interquartile range (IQR) below the first quartile or above the third quartile. For the cognitive parameters, the outliers were removed after transformation of the data. In addition, a sensitivity analysis of all outcome parameters was performed among all participants with imaging data available, without exclusion based on psychiatric, neurological, or endocrinological history, or medication use.

All statistical analyses and data visualization were performed in R (version 4.1.1) [49] using the packages tidyverse (version 1.3.1) [50], car (version 3.0-11) [51], emmeans (version 1.7.0; https://cran.r-project.org/package=emmeans), lmtest (0.9-38) [52], mediation (version 4.5.0) [53], fauxnaif (version 0.6.1; https://cran.r-project.org/package=fauxnaif), ggpubr (version 0.4.0; https://rpkgs.datanovia.com/ggpubr/), and cowplot (version 1.1.1; https://cran.r-project.org/package=cowplot).

Patient and public involvement

Patients and the public were not directly involved in the design or implementation of this study, since we used previously collected data.

For peer review only - http://bmjopen.bmj.com/site/about/guidelines.xhtml

Results

Demographic characteristics

In total, 222 patients using systemic glucocorticoids, 557 patients using inhaled glucocorticoids, and 24,106 controls were included. As shown in Table 1, these groups did not differ significantly with respect to sex, education, and smoking status, while the systemic glucocorticoid group was slightly older than the other groups (mean age 66.1 ± 7.2 years for systemic glucocorticoid users; 63.3 ± 7.5 years for inhaled glucocorticoid users; 63.5 ± 7.5 years for controls), and the inhaled glucocorticoid group had a higher BMI and body fat percentage (Supplement 3.1).

For peer review only

278 **Table 1. Characteristics of included patients using systemic glucocorticoids (n = 222), inhaled glucocorticoids (n = 557), and controls**

	Patients using systemic GC (n = 222)	Patients using inhaled GC (n = 557)	Controls (n = 24106)	P value (ANOVA)	Systemic GC vs. controls**		Inhaled GC vs. controls**	
					Mean difference (95% CI)	P value	Mean difference (95% CI)	P value
**Sex: male, n (%)***	111 (50.0%)	253 (45.4%)	12154 (50.4%)	0.066				
**Age at time of scanning in years, mean (SD)***	66.1 (7.2)	63.3 (7.5)	63.5 (7.5)	2.4e-6	2.6 (1.4, 3.7)	<.0001	-0.2 (-0.9, 0.5)	0.81
**Education level, n (%)**				0.66				
**College/University degree**	108 (48.6)	287 (51.5)	12058 (50.0)					
**A levels or equivalent**	26 (11.7)	66 (11.8)	2930 (12.2)					
**O levels/GCSE or equivalent**	38 (17.1)	96 (17.2)	4155 (17.2)					
**CSEs or equivalent**	9 (4.1)	17 (3.1)	879 (3.6)					
**NVQ, HND, HNC, or equivalent**	6 (2.7)	35 (6.3)	1396 (5.8)					
**Other professional**	13 (5.9)	29 (5.2)	1150 (4.8)					
**qualifications**	18 (8.1)	25 (4.5)	1311 (5.4)					
**None of the above**	4 (1.8)	2 (0.4)	227 (0.9)					
**Missing**								
**BMI in kg/m², mean (SD)**	26.2 (3.9)	26.7 (4.3)	26.1 (4.1)	1.0e-3	0.0 (-0.6, 0.7)	0.98	0.6 (0.2, 1.0)	1.3e-3
**Number (%) missing**	7 (3.2)	20 (3.6)	1325 (5.5)					
**Body fat percentage, mean (SD)**	30.9 (7.9)	32.1 (8.3)	30.2 (7.9)	3.5e-8	0.7 (-0.5, 1.9)	0.36	1.9 (1.1, 2.7)	<1.0e-4
**Number (%) missing**	7 (3.2)	20 (3.6)	1331 (5.5)					
**Smoking status, n (%)**				0.44				
**Current**	6 (2.7)	11 (2.0)	647 (2.7)					
**Previous**	76 (34.2)	200 (35.9)	7858 (32.6)					
**Never**	137 (61.7)	341 (61.2)	15380 (63.8)					
**Missing**	3 (1.4)	5 (0.9)	221 (0.9)					

For peer review only

279 * There were no missing values for the variables sex and age. BMI, body mass index; CI, confidence interval; GC, glucocorticoids; n, number; SD, standard deviation.

281 ** Calculated using post-hoc Dunnett's test, only for those variables with a statistical difference according to the ANOVA.

For peer review only

Volumetric imaging parameters

Fifteen out of 22 predefined ROIs for the volumetric imaging were significantly different across the groups according to the ANOVA (Supplement 3.2). However, none of the 'global volume' parameters reached statistical significance in the post-hoc tests (Table 2, Figure 3, Supplement 4). With respect to 'subcortical volumes', the caudate was larger in systemic glucocorticoid users compared with controls (adjusted mean difference (AMD) = 77.8 mm^3, 95% confidence interval (CI) = 24.5 to 131.1). None of the subcortical volumes (containing both grey and white matter) differed significantly between inhaled glucocorticoid users and controls. Of the 'regional grey matter volumes', the caudate was larger in systemic glucocorticoid users compared with controls (AMD = 178.7 mm^3, 95% CI = 82.2 to 275.0), and inhaled glucocorticoid users had smaller grey matter volumes in the amygdala (AMD = -23.9 mm^3, 95% CI =-41.5 to -6.2).

To assess whether chronic or repeated glucocorticoid exposure was associated with greater changes in imaging parameters, subgroup analyses among chronic systemic glucocorticoid users (n = 42) and chronic inhaled glucocorticoid users (n = 305) were performed (demographic characteristics are presented in Supplement 5). As expected, only few of the investigated imaging parameters reached statistical significance, potentially due to the lower power resulting from the smaller group sizes than in the main analysis (Supplement 3.3). Nevertheless, in chronic systemic glucocorticoid users, global volumes showed the same patterns of reduction as in the main analysis, and the caudate showed a larger increase in subcortical volume, but a smaller increase in grey matter volume. For chronic inhaled glucocorticoid users, the patterns were like those in the main analysis, with no striking differences in effect sizes (Supplements 3.3, 6, and 7).

For peer review only

Table 2. Imaging parameters, presented as the adjusted mean difference of patients using systemic glucocorticoids (n = 222) or inhaled glucocorticoids (n = 557) compared with controls (n = 24106)

	ANOVA			Systemic GC vs. controls			Inhaled GC vs. controls		
	F value	P value	P_{FDR}	AMD*	95% CI	P value	AMD*	95% CI	P value
Volumetric measures									
Global volumes (in mm^3)									
Total brain volume	19.7	2.8e-9	**1.0e-8**	-3688	-10627; 3252	0.39	3374	-1012; 7760	0.16
Grey matter volume	23.7	5.4e-11	**6.5e-10**	-1968	-5904; 1968	0.43	1012	-1476; 3500	0.57
White matter volume	6.7	1.2e-3	**2.0e-3**	-1720	-6273; 2833	0.61	2362	-516; 5240	0.13
Peripheral cortex	21.1	6.9e-10	**6.2e-9**	-3303	-6843; 237	0.072	1033	-1205; 3270	0.49
CSF volume	10.1	4.2e-5	**9.5e-5**	1215	-824; 3254	0.32	78	-1211; 1367	0.98
Subcortical volumes (in mm^3)									
Accumbens	12.0	6.0e-6	**1.7e-5**	-13.1	-26.7; 0.5	0.062	-6.5	-15.1; 2.1	0.17
Caudate	6.7	1.3e-3	**2.0e-3**	77.8	24.5; 131.1	**0.0023**	-2.7	-36.4; 30.9	0.97
Pallidum	7.7	4.5e-4	**7.8e-4**	0.8	-29.9; 31.4	1.00	-18.0	-37.3; 1.4	*0.074*
Putamen	10.9	1.8e-5	**4.6e-5**	-31.3	-98.2; 35.6	0.48	-27.9	-70.2; 14.4	0.25
Thalamus	8.2	2.7e-4	**4.9e-4**	3.6	-74.0; 81.1	0.99	-6.4	-55.4; 42.6	0.93
Regional grey matter volumes (in mm^3)									
Amygdala	23.8	5.0e-11	**6.5e-10**	-4.0	-31.9; 23.8	0.91	-23.9	-41.5; -6.2	**5.2e-3**
Caudate	13.0	2.3e-6	**7.5e-6**	178.7	82.2; 275.0	**1.0e-4**	41.2	-19.8; 102.0	0.24
Cerebellum	10.8	2.0e-5	**4.8e-5**	25.1	-18.4; 68.5	0.34	-12.2	-39.7; 15.3	0.51
Insular cortex	8.5	2.0e-4	**3.9e-4**	-36.2	-108.4; 36.0	0.43	5.0	-40.6; 50.7	0.95

For peer review only

For peer review only - http://bmjopen.bmj.com/site/about/guidelines.xhtml

Precuneal cortex	5.5	4.3e-3	**5.6e-3**	-21.5	-179.0; 136.3	0.92	-7.4	-107.0; 92.4	0.97
DTI measures									
Fractional anisotropy									
Global	19.2	4.6e-9	**2.8e-8**	-0.0037	-0.0064; -0.0010	**4.2e-3**	-0.0023	-0.0040; -5.7e-4	**5.7e-3**
Body of corpus callosum	10.0	4.7e-5	**1.0e-4**	-0.0043	-0.0084; -1.2e-4	**0.043**	-0.0023	-0.0049; 3.0e-4	0.092
Genu of corpus callosum	16.8	5.4e-8	**2.1e-7**	-0.0064	-0.011; -0.0017	**5.0e-3**	-0.0019	-0.0049; 0.0011	0.27
Splenium of corpus callosum	5.4	4.4e-3	**5.6e-3**	-0.0021	-0.0053; 0.0012	0.27	-0.0032	-0.0052; -0.0012	**1.0e-3**
Cingulum cingulate	6.1	2.4e-3	**3.4e-3**	-0.0017	-0.0062; 0.0028	0.61	-0.0028	-0.0057; 8.9e-6	*0.051*
Cingulum hippocampus	6.4	1.7e-3	**2.5e-3**	6.5e-5	-0.0046; 0.0048	1.00	-3.4e-3	-0.0063; -3.8e-4	**0.024**
Mean diffusivity									
Global	25.9	5.8e-12	**2.1e-10**	7.2e-6	3.2e-6; 1.1e-5	**1.0e-4**	2.7e-6	1.7e-7; 5.2e-6	**0.034**
Body of corpus callosum	15.5	2.0e-7	**7.0e-7**	6.9e-6	1.7e-6; 1.2e-5	**6.0e-3**	4.8e-6	1.6e-6; 8.1e-6	**2.0e-3**
Genu of corpus callosum	18.0	1.6e-8	**7.0e-8**	8.4e-6	2.2e-6; 1.5e-5	**4.9e-3**	4.1e-6	1.7e-7; 8.0e-6	**0.039**
Splenium of corpus callosum	9.7	6.2e-5	**1.2e-4**	4.4e-6	-3.8e-8; 8.9e-6	0.050	5.3e-6	2.4e-6; 8.1e-6	**1.0e-4**
Cingulum cingulate	5.4	4.3e-3	**5.6e-3**	2.9e-6	-8.5e-7; 6.6e-6	0.16	2.8e-6	4.7e-7; 5.2e-6	**0.015**
Cingulum hippocampus	18.5	9.1e-9	**4.7e-8**	5.0e-6	4.2e-7; 9.5e-6	**0.029**	5.6e-6	2.8e-6; 8.5e-6	**<1.03-4**
Uncinate fasciculus	12.1	5.4e-6	**1.6e-5**	6.4e-6	2.2e-6; 1.1e-5	**1.4e-3**	2.2e-6	-4.4e-7; 4.9e-6	0.12

* Adjusted mean difference, calculated using linear models, adjusted for age, sex, education, X-, Y-, and Z-position of the head in the scanner, head size, assessment centre, and year of imaging acquisition; significance was determined using a post-hoc Dunnett's test.

CI, confidence interval; P_{FDR}, Benjamini-Hochberg false discovery rate corrected P values. P values in bold are statistically significant ($P < 0.05$).

For peer review only

Diffusion imaging parameters

All but one of the diffusion imaging parameters differed significantly across the groups. Post-hoc tests showed that systemic glucocorticoid use was associated with reduced global FA (AMD = -3.7e-3, 95% CI = -6.4e-3 to 1.0e-3), and reductions in regional FA were observed in the body and genu of the corpus callosum (Table 2, Figure 3, Supplement 4). Similarly, inhaled glucocorticoid use was associated with reduced global FA (AMD = -2.3e-3, 95% CI = -4.0e-3 to -5.7e-4), and the splenium of the corpus callosum and the cingulum of the hippocampus also showed a lower FA. For most ROIs, reductions in FA were smaller in inhaled glucocorticoid users than in systemic glucocorticoid users.

Furthermore, global MD was higher in systemic glucocorticoid users (AMD = 7.2e-6, 95% CI = 3.2e-6 to 1.1e-5) and inhaled glucocorticoid users compared with controls (AMD = 2.7e-6, 95% CI = 1.7e-7 to 5.2e-6). Systemic glucocorticoid was associated with higher regional MD in the body and genu of the corpus callosum, the cingulum of the hippocampus, and the uncinate gyrus. Inhaled glucocorticoid use showed significant associations with increased MD in the body, genu, and splenium of the corpus callosum, the cingulum of the cingulate cortex, and the cingulum of the hippocampus. Again, effect sizes were similar or smaller for most tracts compared with the associations observed in systemic glucocorticoid users.

For chronic glucocorticoid users, the tendencies of FA and MD outcomes were in the same direction as the main analysis for all ROIs. Almost all associations with global and regional FA and MD showed a greater effect size among chronic systemic glucocorticoid users than in the main analysis, although only the global FA and MD measures, and FA and MD in the genu of the corpus callosum reached significance. In chronic inhaled glucocorticoid users, however, the effect sizes were not remarkably different from those observed in the main analysis (Supplements 3.3, 6, and 7).

Cognitive and emotional outcomes

ANOVA showed differences between the groups on three cognitive tasks: trail making A, trail making B, and symbol substitution (Supplement 3.4). Post-hoc testing revealed that systemic glucocorticoid users performed significantly worse on the symbol digit substitution task compared with controls (AMD = -0.17 SD, 95% CI = -0.34 to -0.01; Table 3). With regards to the emotional outcomes, between-group differences were observed in the frequency of depressive symptoms ($P = 0.0049$), disinterest ($P = 0.0049$), tenseness/restlessness ($P = 0.0025$) and tiredness/lethargy ($P = 3.7e-7$) (Supplements 3.5 and 8). Pairwise comparisons using logistic regression analysis revealed that systemic glucocorticoid users experienced more depressive symptoms (OR = 1.76, 95% CI = 1.25 to 2.43), disinterest (OR = 1.84, 95% CI = 1.29 to 2.56), tenseness/restlessness (OR = 1.78, 95% CI = 1.29 to 2.41), and tiredness/lethargy (OR = 1.90, 95% CI = 1.45 to 2.50) compared with controls (Table 4),

For peer review only - http://bmjopen.bmj.com/site/about/guidelines.xhtml

while inhaled glucocorticoid users only reported more tiredness/lethargy than controls (OR = 1.35, 95% CI = 1.14 to 1.60).

For the chronic users, none of the cognitive outcomes was significantly different in systemic or inhaled glucocorticoid users compared with controls in the post-hoc analysis. Effect sizes for chronic systemic glucocorticoid users were even smaller than in the entire cohort, while two out of three were slightly larger in the chronic inhaled glucocorticoid users compared with the entire cohort (Supplements 3.6 and 9). Likewise, the emotional outcome parameters did not differ significantly, except for tiredness/lethargy which was more common in inhaled glucocorticoid users compared with controls. Remarkably, most odds ratios were lower than in the main analysis (Supplements 3.7 and 10).

For peer review only

Table 3. Cognitive outcome measures of patients using systemic glucocorticoids (n = 222) or inhaled glucocorticoids (n = 557) compared with controls

	ANOVA			Systemic GC vs. controls			Inhaled GC vs. controls			Participants with available data, n (%)		
	F value	P value	P_{FDR}	AMD*	95% CI	P value	AMD*	95% CI	P value	Systemic GC	Inhaled GC	Controls
**Trail making A**	5.6	0.0036	**7.3e-3**	-0.11	-0.28; 0.06	0.25	-0.031	-0.15; 0.09	0.78	149 (67)	296 (53)	16419 (68)
**Trail making B**	6.1	0.0023	**6.8e-3**	-0.12	-0.30; 0.05	0.19	-0.0077	-0.13; 0.11	0.98	139 (63)	291 (52)	16071 (67)
**Symbol substitution**	10.3	3.5e-5	**2.1e-4**	-0.17	-0.34; -0.01	**0.04**	-0.035	-0.15; 0.08	0.72	146 (66)	298 (54)	16442 (68)

* Adjusted mean difference between patients and controls, expressed in Z scores. Calculated using linear models, adjusting for age, sex, and education. Trail making A, and trail making B were log transformed before generation of Z scores because they were non-normally distributed. Variables were transformed such that higher values indicate a better performance. Significance was determined using a post-hoc Dunnett's test.

CI, confidence interval; GC, glucocorticoids; n, number; P_{FDR}, Benjamini-Hochberg false discovery rate corrected P values.

P values in bold are statistically significant (P < 0.05).

For peer review only

369 **Table 4. Likelihood of experiencing mental health complaints in the past two weeks of systemic**
370 **glucocorticoid users (n = 222) and inhaled glucocorticoid users (n = 557) compared with controls**
371

	Likelihood ratio test			Systemic GC vs. controls			Inhaled GC vs. controls		
	X^2	P value	P_{FDR}	OR	95% CI	P value	OR	95% CI	P value
**Depression**	10.6	0.0049	0.0049	1.76	1.25; 2.43	**8.2e-4**	1.10	0.87; 1.38	0.43
**Disinterest**	10.9	0.0043	0.0049	1.84	1.29; 2.56	**5.1e-4**	1.06	0.82; 1.36	0.64
**Tenseness**	13.4	0.0012	0.0025	1.78	1.29; 2.41	**3.0e-4**	1.16	0.92; 1.43	0.19
**Tiredness**	32.4	9.2e-8	3.7e-7	1.90	1.45; 2.50	**4.4e-6**	1.35	1.14; 1.60	**6.3e-4**

372
373 Calculated using logistic regression analysis, adjusting for age, sex, and education.
374 P values in bold are statistically significant after Bonferroni correction for family-wise error rate of
375 two tests (P < 0.025).
376 CI, confidence interval; GC, glucocorticoids; OR, odds ratio; P_{FDR}, Benjamini-Hochberg false discovery
377 rate corrected P values.
378
379

For peer review only

Sensitivity analyses

In the first sensitivity analysis we included the subjects that were previously excluded based on neurological, psychiatric, or endocrine history or medication use. The imaging outcomes were comparable to those of the main analysis, with similar ROIs showing significant differences between the groups (Supplements 3.8 to 3.10, and Supplements 11 to 15), although the differences in diffusion parameters between glucocorticoid users and controls were more pronounced in the main analysis than in the unselected group. The same was observed for the cognitive and emotional outcomes.

For the second sensitivity analysis, outliers of the imaging and cognitive outcomes (<3% for most parameters) were excluded (Supplements 3.11 to 3.14, and Supplements 16 and 17), which led to the same conclusions for the imaging outcomes, except for a small number of regions that had shown a tendency in the main analysis and reached significance after exclusion of outlier values (subcortical accumbens volume, insular grey matter volume, and MD in the splenium of the corpus callosum; all in systemic glucocorticoid users). For the cognitive outcomes, exclusion of outliers resulted in not only a significant reduced score on the symbol digit substitution test, but also on the trail making B test for the systemic glucocorticoid users.

Mediation analyses

To assess whether total body fat percentage could have mediated the association between glucocorticoid use and brain volume and white matter microstructure, mediation analysis was performed. For none of the investigated imaging outcomes was a significant mediation effect by body fat percentage found (Supplement 3.15), suggesting that the observed associations were independent of body fat.

For peer review only

Discussion

This study shows that in the large population-based cohort of the UK Biobank, the use of not only systemic glucocorticoids but also inhaled glucocorticoids is associated with changes in several brain imaging parameters. Most notably, the previously reported glucocorticoid effects on white matter microstructure [32] were also detected in this population and are therefore likely to be widespread amongst glucocorticoid users. Subgroup analyses among people using chronic glucocorticoids suggested a potential dose-dependent or duration-dependent effect of glucocorticoids on white matter microstructure, with smallest effect sizes in inhaled glucocorticoid users, larger effect sizes in systemic glucocorticoid users, and the largest effect sizes in chronic systemic glucocorticoid users. While it remains unclear whether the observed effect sizes have clinical consequences for the population of glucocorticoid users as a whole, these findings are remarkable given the common neuropsychiatric side effects of synthetic glucocorticoids, and the observed changes may play a role in those patients suffering from these side effects.

Findings in context

Previous studies in people exposed to high levels of endogenous glucocorticoids due to Cushing disease or high-dose synthetic systemic glucocorticoids have shown that glucocorticoid overexposure is associated with global cerebral atrophy and cortical thinning, as well as volumetric changes in specific brain areas. For example, reductions of grey matter volume have been observed in the hippocampus [14, 24, 25, 27, 30, 31, 54], amygdala [18, 28, 55], cingulate cortex [13, 22, 23], insula [13], caudate [19], and cerebellum [17, 25, 26], which have all been implicated in cognitive processes and emotional regulation [56-61]. However, not all findings were consistent across studies, which may in part be due to differences between patient populations (e.g., with respect to duration and type of glucocorticoid exposure), the small sample sizes of the studies, and the different analysis methods used, with some studies only focussing on one specific brain region, and others performing whole-brain analysis. In general, studies have mainly been dedicated to structural imaging with a specific interest in grey matter volume, while diffusion imaging has only been performed by a few studies in patients with Cushing disease [32-35].

The present study extends these findings by investigating brain volumes and white matter microstructure in not only systemic glucocorticoid users, but also inhaled glucocorticoid users, in whom neuropsychiatric side effects have been reported too [62]. The most remarkable and consistent associations were observed in white matter integrity, as both systemic and inhaled glucocorticoid use was associated with widespread reductions in FA and increases in MD. Although these associations were only about 10% of the effect sizes previously found in Cushing patients [32], this adds to the growing body of literature suggesting that glucocorticoids have important impact on white

For peer review only - http://bmjopen.bmj.com/site/about/guidelines.xhtml

matter, and that non-neuronal cells such as oligodendrocytes are very sensitive to glucocorticoids. Animal studies have shown that glucocorticoid exposure inhibits proliferation of oligodendrocyte progenitor cells throughout the white matter [63], and induce changes in the expression of myelin basic protein (MBP), an oligodendrocyte marker [36, 64]. Since oligodendrocytes are responsible for myelin production, glucocorticoid-induced changes in oligodendrocytes may underly the reduced white matter microstructure observed in patients using glucocorticoids. Besides oligodendrocytes, other glia cells including microglia and astrocytes are also affected by glucocorticoids, with multiple reports of decreased cell viability, proliferation, and immunoreactivity of microglia and astrocytes in response to glucocorticoids [65-68].

Although we observed some patterns in global and regional brain volumes in glucocorticoid users, most of these did not reach significance. Rather surprisingly, although none of the global volumes was significantly different between patients and controls, the direction of change for all the areas was different for systemic (decreased volumes) vs. inhaled glucocorticoid users (increased volumes). We did observe a significant association between inhaled glucocorticoid use and decreased grey matter volume of the amygdala, and systemic glucocorticoid use was associated with an increase in total and grey matter volume of the caudate nucleus. Decreased amygdala volumes have previously been reported in chronic systemic glucocorticoid users [18, 28, 55]. However, the increase in caudate volume contrasts with two previous studies that found larger caudate volumes after treatment of Cushing disease compared with during active disease [19, 20], while one other study reported an increased caudate volume in remitted patients compared with controls, but no differences in patients with active Cushing disease compared with controls [21]. Those findings suggest that cortisol excess caused a decreased caudate volume in these patients and/or that the caudate volume increased in response to normalization of cortisol levels. The modest association of glucocorticoid use with brain volumes in the present population-based cohort study could indicate that white matter integrity is more sensitive to glucocorticoids than grey matter volume, and that longer or higher glucocorticoid exposure is needed to also induce volumetric changes.

It is tempting to relate these findings to glucocorticoid (GR) and mineralocorticoid receptor (MR) expression profiles in the brain. Previously, our group correlated the expression of GR and MR in several brain areas (data from the Allen Brain Atlas [69]) to the changes in brain volume observed in the extreme hypercortisolism caused by Cushing disease. [23] We then concluded that, although a high expression of these receptors was seen in the key brain areas such as the hippocampus, anterior cingulate cortex, and amygdala, there was no clear correlation between receptor expression profiles and brain areas affected by hypercortisolism. Receptor expression appears necessary but not predictive in this case. One might speculate that whether an area is affected by glucocorticoids may be more related to the densities of specific cell types that are responsive to glucocorticoids than the

For peer review only

expression of receptors per se. Perhaps the density of oligodendrocytes, which are increasingly recognized as glucocorticoid-responsive, could be an important factor determining the responsiveness of different brain areas to glucocorticoids.

Potential consequences and implications

It is well-known that exogenous glucocorticoids are associated with neuropsychiatric side effects, including not only potentially severe mood disturbances such as depression and mania, but also cognitive impairment such as concentration and memory problems [10]. In the present study, glucocorticoid users reported a higher frequency of several mental health complaints, while their cognitive performance was not significantly different, except for worse scores on the symbol digit substitution task in systemic glucocorticoid users. It should be noted that only a few mood-related parameters assessed by the UK Biobank were selected for this study, because these were the only parameters that applied specifically to the previous two weeks, in which the glucocorticoid users were likely already using their medication. Ideally, more aspects of mood would have been assessed to get a more comprehensive view on the glucocorticoid users' psychological functioning. Furthermore, the observed mood-related effects may not be caused by glucocorticoid use per se but could also be related to the condition for which glucocorticoids were prescribed. For example, autoimmune and inflammatory diseases commonly treated with glucocorticoids, such as rheumatoid arthritis and chronic obstructive pulmonary disorder, have also been associated with mental health impairment and reduced quality of life [70, 71].

Nevertheless, awareness for the potential of glucocorticoids to affect the brain and cause neuropsychiatric symptoms is important, since these medications are prescribed for a wide range of conditions by many different medical specialties and are used by a substantial proportion of the population. Moreover, further research into the underlying mechanisms, reversibility, and risk factors for development of neuropsychiatric side effects of glucocorticoids is warranted, ideally considering dose and duration of glucocorticoids, as well as single nucleotide polymorphisms (SNPs) in the glucocorticoid receptor gene (NR3C1) that affect glucocorticoid sensitivity. For those patients experiencing side effects, alternative treatment options should also be investigated. One promising direction is the development of selective GR modulators, since these (ideally) only activate the desired downstream signalling pathways in the desired cell types, limiting the potential side effects [72, 73].

Strengths and limitations

To the best of our knowledge, this is the largest study to date assessing the association between glucocorticoid use and brain structure, and the first to investigate these associations in inhaled

For peer review only - http://bmjopen.bmj.com/site/about/guidelines.xhtml

glucocorticoid users. For the selection of patients and controls, we applied relatively strict exclusion criteria to limit the potential confounding that may arise in observational cohort studies. Although not all neurological disorders, especially peripheral disorders, may have a clear impact on brain volume or white matter microstructure, UK Biobank participants with these conditions were excluded to prevent any confounding by these comorbidities. Our sensitivity analysis suggested that these conditions did not have a large impact on the results. However, we decided not to exclude patients with a history of depression, anxiety, mania, or delirium, because these are known possible consequences of glucocorticoid use [10], and we did not want to exclude patients based on potentially glucocorticoid-related outcomes.

Another method used to limit confounding was adjustment of the regression analyses for relevant confounding variables, including demographic variables and variables related to the imaging visits (e.g., assessment centre, position of the head in the scanner). For both the volumetric and diffusion parameters, head size was used as covariate, because previous research not only found a relation between head size and brain volume, but also between head size and DTI parameters [74, 75]. The use of this variable as covariate is also recommended by the UK Biobank [46]. We decided not to include a measure of body weight or body composition as covariate, because it is known that glucocorticoids can cause obesity [7], which is therefore more likely to be in the causal pathway than to be a confounder. Our mediation analysis, however, suggested that body fat percentage did not mediate the associations identified. Nevertheless, despite the correction for a wide range of potential confounders, it should be noted that the possibility of residual confounding cannot be excluded.

In addition, although a causal relation between glucocorticoid use and changes in the brain is likely based on the present and previous studies, the cross-sectional nature of this study does not allow for formal conclusions on causality. Demonstrating a dose-response effect of glucocorticoid on imaging parameters would have increased the likelihood of a causal relation, but unfortunately, dose and duration of medication use were not available in the UK Biobank. We were therefore only able to give an indication of a dose-response effect by performing separate analyses in systemic glucocorticoid users, inhaled glucocorticoid users (representing a group exposed to lower systemic concentration of glucocorticoids), and subgroups of patients using systemic or inhaled glucocorticoid chronically (representing groups with a longer duration and larger cumulative dose of glucocorticoid use). The fact that the effect sizes of the associations between glucocorticoid use and diffusion imaging parameters are generally largest in the chronic systemic glucocorticoid group, and smallest in the inhaled glucocorticoid group, indicates that a dose-dependent or duration-dependent effect may exist, although the expected lower power of the small chronic systemic glucocorticoid group likely precluded most associations from reaching significance. Moreover, while the association effect

For peer review only - http://bmjopen.bmj.com/site/about/guidelines.xhtml

size estimates were larger in chronic systemic glucocorticoid users compared with the main group using systemic glucocorticoids, this difference was not observed among inhaled glucocorticoid users. A potential explanation may be that inhaled glucocorticoids are generally prescribed for a longer duration than systemic glucocorticoids, which is also reflected by the high percentage of inhaled glucocorticoid users (326/592, 55%) that could be included in the subgroup of chronic users, compared with the lower percentage of chronic systemic glucocorticoid users (48/234, 21%).

Another limitation is that we could not differentiate between oral and parenteral glucocorticoids because of the medication names used by the UK Biobank. We were therefore unable to conduct separate analyses for these groups and analysed them together as systemic glucocorticoid users. Also, 14 participants used both inhaled and systemic glucocorticoids. Since this group was too small to analyse separately in a meaningful way, these participants were included in the systemic group. Although simultaneous use of different glucocorticoids might be associated with more profound changes in the brain, we do not expect that this association is larger than the effect size differences that may exist because of differences in dosages of the systemic glucocorticoids. Lastly, some seasonal patterns in glucocorticoid use may exist depending on the indications, which we were unable to adjust for in the analyses.

Conclusion

This study shows that both systemic and inhaled glucocorticoids are associated with an apparently widespread reduction in white matter integrity, which may in part underly the neuropsychiatric side effects observed in patients using glucocorticoids. Since these medications are widely used, awareness of these associations is necessary across medical specialties, and research into alternative treatment options is warranted.

For peer review only

Declarations

Contributors: JMA and OCM contributed to the study conception. MvdM designed the study, performed the analyses, and wrote the first version of the manuscript. OMD contributed to the statistical analyses. All authors read and commented on the manuscript and approved the final version of the manuscript. MvdM and OCM are the guarantors of the manuscript and accept full responsibility for the work and conduct of the study, had access to the data, and controlled the decision to publish. The corresponding author attests that all listed authors meet authorship criteria and that no others meeting the criteria have been omitted.

Acknowledgments: We would like to thank dr. Roula Tsonaka for her advice regarding the statistical analysis, and dr. Steven van der Werff for his suggestion for this study.

Competing interests: All authors have completed the ICMJE uniform disclosure form at http://www.icmje.org/disclosure-of-interest/ and declare: MvdM received financial support from the MD/PhD grant of the Leiden University Medical Center, and JMA received financial support from CONACyT (the National Council for Science and Technology-Government of Mexico) for the submitted work; OCM has received research grants and honorariums from Corcept Therapeutics, and a speakers' fee from Ipsen; no other relationships or activities that could appear to have influenced the submitted work.

Funding: The UK Biobank was established by the Wellcome Trust, Medical Research Council, Department of Health, Scottish government, and Northwest Regional Development Agency. It also received funding from the Welsh Government, the British Heart Foundation, Cancer Research UK, and Diabetes UK. For the analyses presented in this manuscript, MvdM received a personal MD/PhD grant of the Leiden University Medical Center, and JMA received support from CONACyT (the National Council for Science and Technology-Government of Mexico).

Role of the funding source: The funding sources had no role in the study conduct, data collection, analyses, data interpretation, and the decision to submit the manuscript.

Ethics approval: This study was performed under the ethical approval obtained by UK Biobank from the National Health Service National Research Ethics Service (ref 11/NW/0382, 17 June 2011). Data for the present study were obtained from the UK Biobank under application number 59004.

For peer review only

Transparency declaration: The manuscript's guarantors (MvdM and OCM) affirm that the manuscript is an honest, accurate, and transparent account of the study being reported; that no important aspects of the study have been omitted; and that any discrepancies from the study as planned have been explained.

Data availability statement: Data used for this study are available via application to the UK Biobank.

Dissemination to participants and related patient and public communities: Results of the study will be disseminated via the UK Biobank website, accessible for research participants and relevant patient and public communities.

Checklist: The STROBE checklist for observational studies can be found in Supplement 18.

For peer review only

References

1. Laugesen K, Jørgensen JOL, Petersen I, Sørensen HT. Fifteen-year nationwide trends in systemic glucocorticoid drug use in Denmark. Eur J Endocrinol. 2019;181(3):267-73.
2. Fardet L, Petersen I, Nazareth I. Prevalence of long-term oral glucocorticoid prescriptions in the UK over the past 20 years. Rheumatology (Oxford). 2011;50(11):1982-90.
3. Overman RA, Yeh JY, Deal CL. Prevalence of oral glucocorticoid usage in the United States: a general population perspective. Arthritis Care Res (Hoboken). 2013;65(2):294-8.
4. van Staa TP, Leufkens HG, Abenhaim L, Begaud B, Zhang B, Cooper C. Use of oral corticosteroids in the United Kingdom. Qjm. 2000;93(2):105-11.
5. Walsh LJ, Wong CA, Pringle M, Tattersfield AE. Use of oral corticosteroids in the community and the prevention of secondary osteoporosis: a cross sectional study. Bmj. 1996;313(7053):344-6.
6. Rhen T, Cidlowski JA. Antiinflammatory action of glucocorticoids--new mechanisms for old drugs. N Engl J Med. 2005;353(16):1711-23.
7. Fardet L, Fève B. Systemic glucocorticoid therapy: a review of its metabolic and cardiovascular adverse events. Drugs. 2014;74(15):1731-45.
8. Chalitsios CV, Shaw DE, McKeever TM. Risk of osteoporosis and fragility fractures in asthma due to oral and inhaled corticosteroids: two population-based nested case-control studies. Thorax. 2021;76(1):21-8.
9. Savas M, Muka T, Wester VL, van den Akker ELT, Visser JA, Braunstahl GJ, et al. Associations Between Systemic and Local Corticosteroid Use With Metabolic Syndrome and Body Mass Index. J Clin Endocrinol Metab. 2017;102(10):3765-74.
10. Judd LL, Schettler PJ, Brown ES, Wolkowitz OM, Sternberg EM, Bender BG, et al. Adverse consequences of glucocorticoid medication: psychological, cognitive, and behavioral effects. Am J Psychiatry. 2014;171(10):1045-51.
11. Fardet L, Petersen I, Nazareth I. Suicidal behavior and severe neuropsychiatric disorders following glucocorticoid therapy in primary care. Am J Psychiatry. 2012;169(5):491-7.
12. Bourdeau I, Bard C, Noël B, Leclerc I, Cordeau MP, Bélair M, et al. Loss of brain volume in endogenous Cushing's syndrome and its reversibility after correction of hypercortisolism. J Clin Endocrinol Metab. 2002;87(5):1949-54.
13. Hou B, Gao L, Shi L, Luo Y, Guo X, Young GS, et al. Reversibility of impaired brain structures after transsphenoidal surgery in Cushing's disease: a longitudinal study based on an artificial intelligence-assisted tool. J Neurosurg. 2020:1-10.
14. Chen Y, Zhang J, Tan H, Li J, Yu Y. Detrimental effects of hypercortisolism on brain structure and related risk factors. Sci Rep. 2020;10(1):12708.
15. Santos A, Resmini E, Gómez-Ansón B, Crespo I, Granell E, Valassi E, et al. Cardiovascular risk and white matter lesions after endocrine control of Cushing's syndrome. Eur J Endocrinol. 2015;173(6):765-75.
16. Simmons NE, Do HM, Lipper MH, Laws ER, Jr. Cerebral atrophy in Cushing's disease. Surg Neurol. 2000;53(1):72-6.
17. Momose KJ, Kjellberg RN, Kliman B. High incidence of cortical atrophy of the cerebral and cerebellar hemispheres in Cushing's disease. Radiology. 1971;99(2):341-8.
18. Merke DP, Giedd JN, Keil MF, Mehlinger SL, Wiggs EA, Holzer S, et al. Children experience cognitive decline despite reversal of brain atrophy one year after resolution of Cushing syndrome. J Clin Endocrinol Metab. 2005;90(5):2531-6.
19. Starkman MN, Giordani B, Gebarski SS, Berent S, Schork MA, Schteingart DE. Decrease in cortisol reverses human hippocampal atrophy following treatment of Cushing's disease. Biol Psychiatry. 1999;46(12):1595-602.
20. Starkman MN, Giordani B, Gebarski SS, Schteingart DE. Improvement in learning associated with increase in hippocampal formation volume. Biol Psychiatry. 2003;53(3):233-8.

For peer review only - http://bmjopen.bmj.com/site/about/guidelines.xhtml

21. Jiang H, Ren J, He NY, Liu C, Sun YH, Jian FF, et al. Volumetric magnetic resonance imaging analysis in patients with short-term remission of Cushing's disease. Clin Endocrinol (Oxf). 2017;87(4):367-74.
22. Bauduin S, van der Pal Z, Pereira AM, Meijer OC, Giltay EJ, van der Wee NJA, et al. Cortical thickness abnormalities in long-term remitted Cushing's disease. Transl Psychiatry. 2020;10(1):293.
23. Andela CD, van der Werff SJ, Pannekoek JN, van den Berg SM, Meijer OC, van Buchem MA, et al. Smaller grey matter volumes in the anterior cingulate cortex and greater cerebellar volumes in patients with long-term remission of Cushing's disease: a case-control study. Eur J Endocrinol. 2013;169(6):811-9.
24. Toffanin T, Nifosì F, Follador H, Passamani A, Zonta F, Ferri G, et al. Volumetric MRI analysis of hippocampal subregions in Cushing's disease: a model for glucocorticoid neural modulation. Eur Psychiatry. 2011;26(1):64-7.
25. Burkhardt T, Lüdecke D, Spies L, Wittmann L, Westphal M, Flitsch J. Hippocampal and cerebellar atrophy in patients with Cushing's disease. Neurosurg Focus. 2015;39(5):E5.
26. Santos A, Resmini E, Crespo I, Pires P, Vives-Gilabert Y, Granell E, et al. Small cerebellar cortex volume in patients with active Cushing's syndrome. Eur J Endocrinol. 2014;171(4):461-9.
27. Brown ES, D JW, Frol A, Bobadilla L, Khan DA, Hanczyc M, et al. Hippocampal volume, spectroscopy, cognition, and mood in patients receiving corticosteroid therapy. Biol Psychiatry. 2004;55(5):538-45.
28. Desai S, Khanani S, Shad MU, Brown ES. Attenuation of amygdala atrophy with lamotrigine in patients receiving corticosteroid therapy. J Clin Psychopharmacol. 2009;29(3):284-7.
29. Brown ES, Kulikova A, Van Enkevort E, Nakamura A, Ivleva EI, Tustison NJ, et al. A randomized trial of an NMDA receptor antagonist for reversing corticosteroid effects on the human hippocampus. Neuropsychopharmacology. 2019;44(13):2263-7.
30. Nguyen DM, Yassa MA, Tustison NJ, Roberts JM, Kulikova A, Nakamura A, et al. The Relationship Between Cumulative Exogenous Corticosteroid Exposure and Volumes of Hippocampal Subfields and Surrounding Structures. J Clin Psychopharmacol. 2019;39(6):653-7.
31. Brown ES, Jeon-Slaughter H, Lu H, Jamadar R, Issac S, Shad M, et al. Hippocampal volume in healthy controls given 3-day stress doses of hydrocortisone. Neuropsychopharmacology. 2015;40(5):1216-21.
32. van der Werff SJ, Andela CD, Nienke Pannekoek J, Meijer OC, van Buchem MA, Rombouts SA, et al. Widespread reductions of white matter integrity in patients with long-term remission of Cushing's disease. Neuroimage Clin. 2014;4:659-67.
33. Pires P, Santos A, Vives-Gilabert Y, Webb SM, Sainz-Ruiz A, Resmini E, et al. White matter alterations in the brains of patients with active, remitted, and cured cushing syndrome: a DTI study. AJNR Am J Neuroradiol. 2015;36(6):1043-8.
34. Jiang H, He NY, Sun YH, Jian FF, Bian LG, Shen JK, et al. Altered gray and white matter microstructure in Cushing's disease: A diffusional kurtosis imaging study. Brain Res. 2017;1665:80-7.
35. Pires P, Santos A, Vives-Gilabert Y, Webb SM, Sainz-Ruiz A, Resmini E, et al. White matter involvement on DTI-MRI in Cushing's syndrome relates to mood disturbances and processing speed: a case-control study. Pituitary. 2017;20(3):340-8.
36. Amaya JM, Suidgeest E, Sahut-Barnola I, Dumontet T, Montanier N, Pagès G, et al. Effects of Long-Term Endogenous Corticosteroid Exposure on Brain Volume and Glial Cells in the AdKO Mouse. Front Neurosci. 2021;15:604103.
37. Alexander AL, Lee JE, Lazar M, Field AS. Diffusion tensor imaging of the brain. Neurotherapeutics. 2007;4(3):316-29.
38. Sudlow C, Gallacher J, Allen N, Beral V, Burton P, Danesh J, et al. UK Biobank: an open access resource for identifying the causes of a wide range of complex diseases of middle and old age. PLoS Med. 2015;12(3):e1001779.
39. UK Biobank. UK Biobank Body Composition Measurement Version 1.0 2011 [Available from: https://biobank.ndph.ox.ac.uk/ukb/ukb/docs/body_composition.pdf.

For peer review only - http://bmjopen.bmj.com/site/about/guidelines.xhtml

40. Alfaro-Almagro F, Jenkinson M, Bangerter NK, Andersson JLR, Griffanti L, Douaud G, et al. Image processing and Quality Control for the first 10,000 brain imaging datasets from UK Biobank. Neuroimage. 2018;166:400-24.

41. Smith SM, Alfaro-Almagro F, Miller KL. UK Biobank Brain Imaging Documentation 2020 [cited 2022. Available from: https://biobank.ndph.ox.ac.uk/showcase/ukb/docs/brain_mri.pdf.

42. Zhang Y, Brady M, Smith S. Segmentation of brain MR images through a hidden Markov random field model and the expectation-maximization algorithm. IEEE Trans Med Imaging. 2001;20(1):45-57.

43. Patenaude B, Smith SM, Kennedy DN, Jenkinson M. A Bayesian model of shape and appearance for subcortical brain segmentation. Neuroimage. 2011;56(3):907-22.

44. Smith SM, Jenkinson M, Johansen-Berg H, Rueckert D, Nichols TE, Mackay CE, et al. Tract-based spatial statistics: voxelwise analysis of multi-subject diffusion data. Neuroimage. 2006;31(4):1487-505.

45. Mori S, Wakana S, Van Zijl PC, Nagae-Poetscher L. MRI atlas of human white matter: Elsevier; 2005.

46. Alfaro-Almagro F, McCarthy P, Afyouni S, Andersson JLR, Bastiani M, Miller KL, et al. Confound modelling in UK Biobank brain imaging. Neuroimage. 2021;224:117002.

47. Dekkers IA, Jansen PR, Lamb HJ. Obesity, Brain Volume, and White Matter Microstructure at MRI: A Cross-sectional UK Biobank Study. Radiology. 2019;292(1):270.

48. Daley-Yates PT. Inhaled corticosteroids: potency, dose equivalence and therapeutic index. Br J Clin Pharmacol. 2015;80(3):372-80.

49. R Core Team. R: A language and environment for statistical computing. Vienna, Austria: R Foundation for Statistical Computing; 2020 [Available from: https://www.R-project.org/.

50. Wickham H AM, Bryan J, Chang W, McGowan LD, François R, Grolemund G, Hayes A, Henry L, Hester J, Kuhn M, Pedersen TL, Miller E, Bache SM, Müller K, Ooms J, Robinson D, Seidel DP, Spinu V, Takahashi K, Vaughan D, Wilke C, Woo K, Yutani H,. Welcome to the tidyverse. Journal of Open Source Software. 2019;4:1686.

51. Fox J, Weisberg S. An R Companion to Applied Regression. Third ed. Thousand Oaks CA: Sage; 2019.

52. Zeileis A, Hothorn T. Diagnostic Checking in Regression Relationships. R News. 2002;2(3):7-10.

53. Tingley D, Yamamoto T, Hirose K, Keele L, Imai K. mediation: R Package for Causal Mediation Analysis. Journal of Statistical Software. 2014;59(5):1-38.

54. Resmini E, Santos A, Gómez-Anson B, Vives Y, Pires P, Crespo I, et al. Verbal and visual memory performance and hippocampal volumes, measured by 3-Tesla magnetic resonance imaging, in patients with Cushing's syndrome. J Clin Endocrinol Metab. 2012;97(2):663-71.

55. Brown ES, Woolston DJ, Frol AB. Amygdala volume in patients receiving chronic corticosteroid therapy. Biol Psychiatry. 2008;63(7):705-9.

56. Bartsch T, Wulff P. The hippocampus in aging and disease: From plasticity to vulnerability. Neuroscience. 2015;309:1-16.

57. Pessoa L. Emotion and cognition and the amygdala: from "what is it?" to "what's to be done?". Neuropsychologia. 2010;48(12):3416-29.

58. Rolls ET. The cingulate cortex and limbic systems for emotion, action, and memory. Brain Struct Funct. 2019;224(9):3001-18.

59. Gasquoine PG. Contributions of the insula to cognition and emotion. Neuropsychol Rev. 2014;24(2):77-87.

60. Grahn JA, Parkinson JA, Owen AM. The role of the basal ganglia in learning and memory: neuropsychological studies. Behav Brain Res. 2009;199(1):53-60.

61. Schmahmann JD, Caplan D. Cognition, emotion and the cerebellum. Brain. 2006;129(2):290-2.

For peer review only

For peer review only - http://bmjopen.bmj.com/site/about/guidelines.xhtml

62. Savas M, Vinkers CH, Rosmalen JGM, Hartman CA, Wester VL, van den Akker ELT, et al. Systemic and Local Corticosteroid Use Is Associated with Reduced Executive Cognition, and Mood and Anxiety Disorders. Neuroendocrinology. 2020;110(3-4):282-91.
63. Alonso G. Prolonged corticosterone treatment of adult rats inhibits the proliferation of oligodendrocyte progenitors present throughout white and gray matter regions of the brain. Glia. 2000;31(3):219-31.
64. Miguel-Hidalgo JJ, Carter K, Deloach PH, Sanders L, Pang Y. Glucocorticoid-Induced Reductions of Myelination and Connexin 43 in Mixed Central Nervous System Cell Cultures Are Prevented by Mifepristone. Neuroscience. 2019;411:255-69.
65. Unemura K, Kume T, Kondo M, Maeda Y, Izumi Y, Akaike A. Glucocorticoids decrease astrocyte numbers by reducing glucocorticoid receptor expression in vitro and in vivo. J Pharmacol Sci. 2012;119(1):30-9.
66. Zhang H, Zhao Y, Wang Z. Chronic corticosterone exposure reduces hippocampal astrocyte structural plasticity and induces hippocampal atrophy in mice. Neurosci Lett. 2015;592:76-81.
67. van Olst L, Bielefeld P, Fitzsimons CP, de Vries HE, Schouten M. Glucocorticoid-mediated modulation of morphological changes associated with aging in microglia. Aging Cell. 2018;17(4):e12790.
68. Freitas AE, Egea J, Buendia I, Gómez-Rangel V, Parada E, Navarro E, et al. Agmatine, by Improving Neuroplasticity Markers and Inducing Nrf2, Prevents Corticosterone-Induced Depressive-Like Behavior in Mice. Mol Neurobiol. 2016;53(5):3030-45.
69. Allen Institute for Brain Science. Allen Brain Map 2020 [updated 2022; cited 2020. Available from: https://portal.brain-map.org.
70. Machin AR, Babatunde O, Haththotuwa R, Scott I, Blagojevic-Bucknall M, Corp N, et al. The association between anxiety and disease activity and quality of life in rheumatoid arthritis: a systematic review and meta-analysis. Clin Rheumatol. 2020;39(5):1471-82.
71. Pelgrim CE, Peterson JD, Gosker HR, Schols A, van Helvoort A, Garssen J, et al. Psychological co-morbidities in COPD: Targeting systemic inflammation, a benefit for both? Eur J Pharmacol. 2019;842:99-110.
72. Meijer OC, Koorneef LL, Kroon J. Glucocorticoid receptor modulators. Ann Endocrinol (Paris). 2018;79(3):107-11.
73. Van Moortel L, Gevaert K, De Bosscher K. Improved Glucocorticoid Receptor Ligands: Fantastic Beasts, but How to Find Them? Front Endocrinol (Lausanne). 2020;11:559673.
74. Takao H, Hayashi N, Inano S, Ohtomo K. Effect of head size on diffusion tensor imaging. Neuroimage. 2011;57(3):958-67.
75. Takao H, Hayashi N, Ohtomo K. Sex dimorphism in the white matter: fractional anisotropy and brain size. J Magn Reson Imaging. 2014;39(4):917-23.

For peer review only

Figures

Figure 1. Flowchart of participant inclusion

DWI, diffusion-weighted imaging; GC, glucocorticoids; n, number; T1, T1-weighted magnetic resonance imaging (MRI).

Figure 2. Stepwise statistical analysis

ANOVA, analysis of variance; C, controls; FDR, false discovery rate correction; I, inhaled glucocorticoid users; n, number; S, systemic glucocorticoid users.

Figure 3. Bar plots showing the adjusted mean difference (with 95% confidence interval) of all imaging parameters for patients using systemic glucocorticoids (GC) (n = 222) or inhaled GC (n = 557), and subgroups of chronic systemic GC users (n = 42), or chronic inhaled GC users (n = 305) vs. controls (n = 24106)

Significance levels compared with controls: * $P < 0.05$, ** $P < 0.01$, *** $P < 0.001$. AU, arbitrary unit.

For peer review only

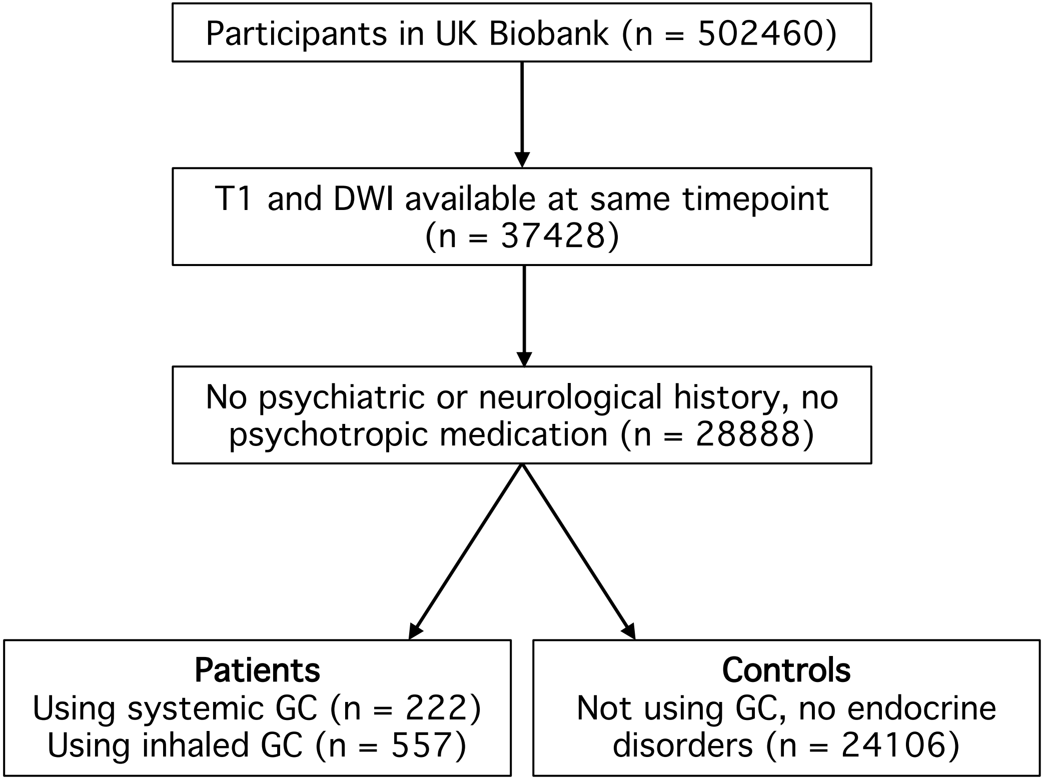
Participants in UK Biobank (n = 502460)
T1 and DWI available at same timepoint (n = 37428)
No psychiatric or neurological history, no psychotropic medication (n = 28888)
Patients
Using systemic GC (n = 222)
Using inhaled GC (n = 557)
Controls
Not using GC, no endocrine disorders (n = 24106)

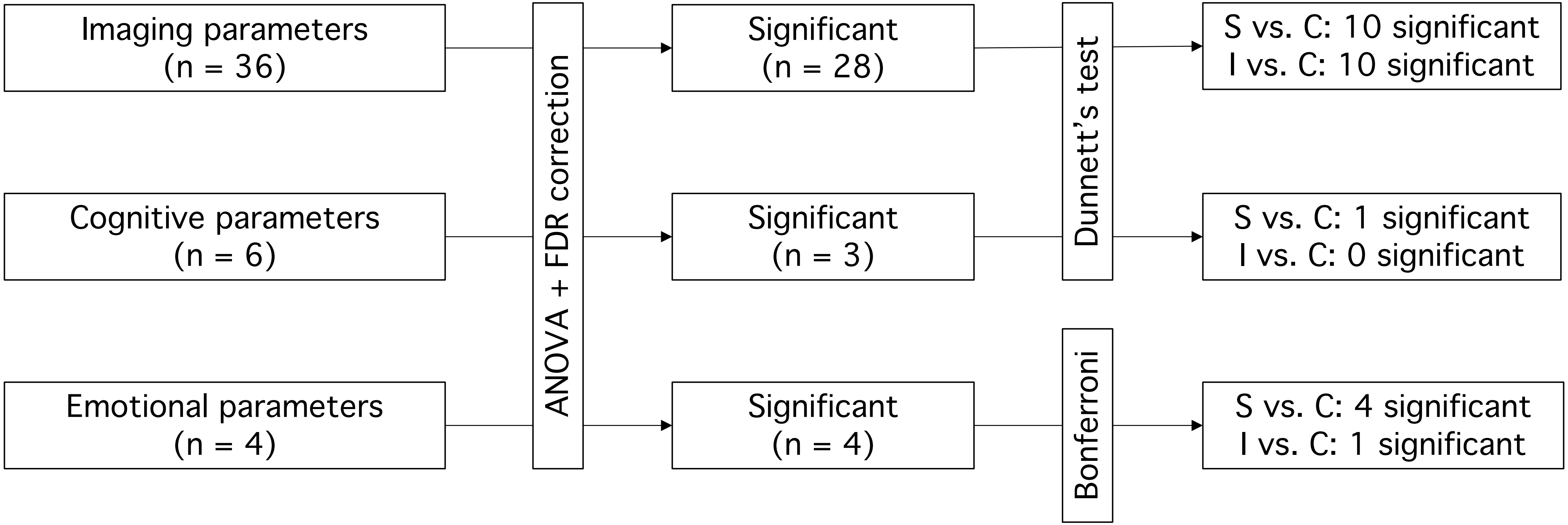
Imaging parameters
(n = 36)
Cognitive parameters
(n = 6)
Emotional parameters
(n = 4)
ANOVA + FDR correction
Significant
(n = 28)
Significant
(n = 3)
Significant
(n = 4)
Dunnett's test
Bonferroni
S vs. C: 10 significant
I vs. C: 10 significant
S vs. C: 1 significant
I vs. C: 0 significant
S vs. C: 4 significant
I vs. C: 1 significant

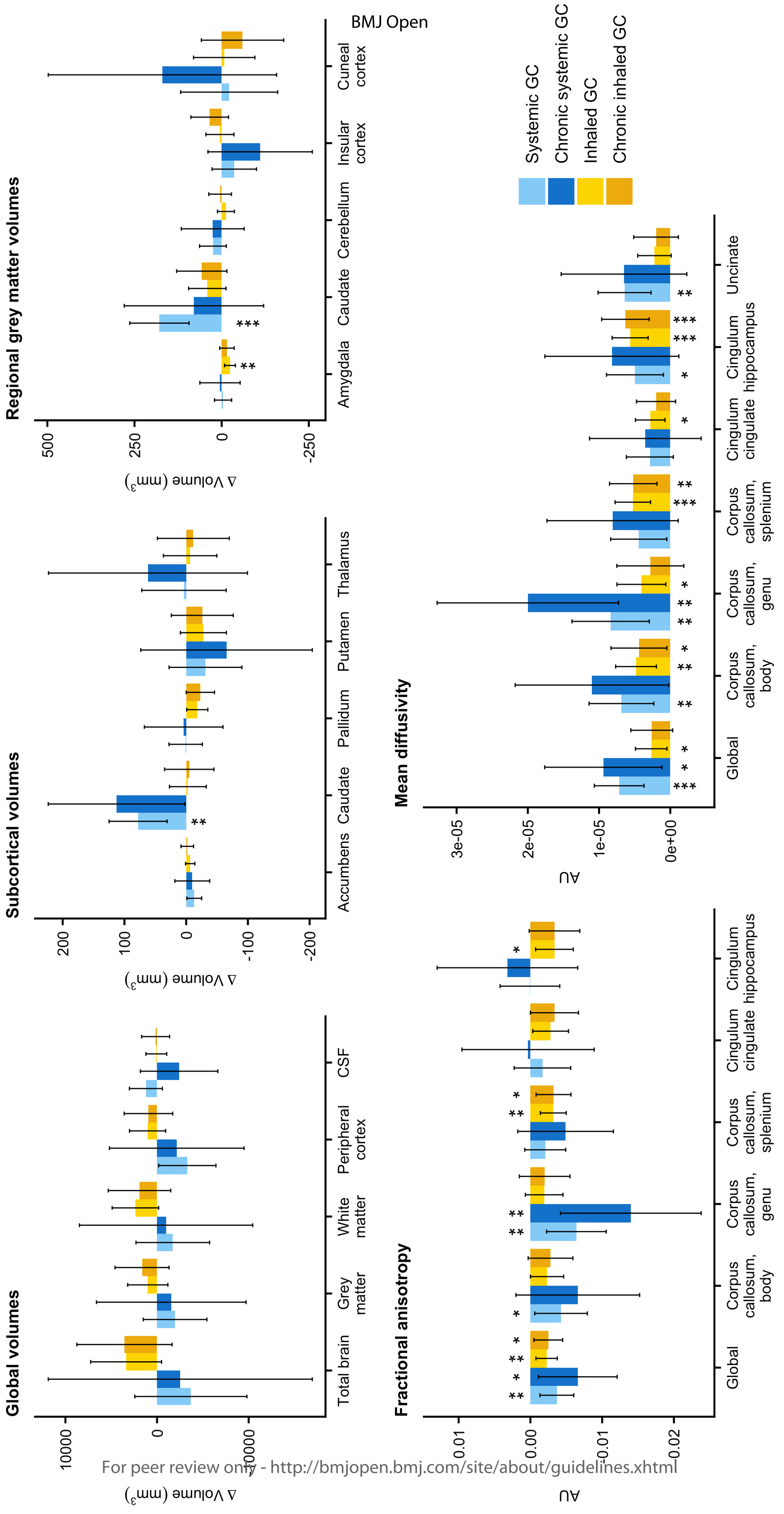

For peer review only - http://bmjopen.bmj.com/site/about/guidelines.xhtml

This file contains all UK Biobank codes used for the selection (inclusion/exclusion) of participants. Patients were included based on systemic or inhalation glucocorticoid (GC) use and availability of imaging parameters; and were excluded based on psychiatric, neurological or endocrinological comorbidity and use of psychotropic drugs. Moreover, this file lists all outcome variables used in the analyses presented in this study (imaging parameters, cognitive parameters, emotional parameters, and demographics and confounds)

For peer review only

Field ID	Code	Description
20003	1140874790	betamethasone
20003	1140874816	dexamethasone
20003	1140874954	hydrocortistab 20mg tablet
20003	1140874956	hydrocortone 10mg tablet
20003	1140874978	medrone 2mg tablet
20003	1140874976	methylprednisolone
20003	1140874950	prednesol 5mg tablet
20003	1140874930	prednisolone
20003	1141157402	prednisolone product
20003	1140868364	prednisone
20003	1140884704	cortisone product
20003	1140874896	hydrocortisone
20003	1140910424	hc - hydrocortisone
20003	1141157294	hydrocortisone product
20003	1141173346	cortisone
20003	1140874944	precortisyl 1mg tablet
20003	1140857532	cortelan 25mg tablet
20003	1140857534	oradexon 500micrograms tablet
20003	1140868370	decortisyl 5mg tablet
20003	1140868426	triamcinolone
20003	1140868434	ledercort 2mg tablet
20003	1140851210	cortenema 100mg/60ml enema
20003	1140874792	betnelan 500mcg tablet
20003	1140874794	betnesol 500mcg soluble tablet
20003	1140874810	cortistab 5mg tablet
20003	1140874814	cortisyl 25mg tablet
20003	1140874822	decadron 500micrograms tablet
20003	1140874936	deltacortril enteric 2.5mg e/c tablet
20003	1140874940	deltastab 1mg tablet
20003	1140910634	deltahydrocortisone
20003	1140910484	cortisol product

For peer review only

Field ID	Code	Description
20003	1140855466	bextasol 100micrograms inhaler
20003	1140881922	becodisks 100micrograms disks+diskhaler
20003	1140881938	beclomethasone dipropionate+salbutamol
20003	1141167594	qvar 50 inhaler
20003	1141174512	budesonide+eformoterol
20003	1141174520	symbicort 100/6 turbohaler
20003	1140862572	budesonide
20003	1140862574	pulmicort ls 50micrograms inhaler
20003	1140862584	pulmicort ls 50micrograms spacer inhaler
20003	1140862476	beclazone 50 inhaler
20003	1140862380	becloforte 250micrograms inhaler
20003	1140862474	aerobec 50mcg autohaler
20003	1140862382	becotide 50 inhaler
20003	1140888098	fluticasone
20003	1141164086	salmeterol+fluticasone propionate
20003	1141176832	seretide 50 evohaler
20003	1141176842	dexsol 2mg/5ml oral solution
20003	1140864286	flixotide 25micrograms inhaler
20003	1141191818	asmanex twisthaler 200mcg breath-actuated dry powder inhaler
20003	1140884654	beclomethasone
20003	1140909786	beclometasone
20003	1141179072	pulvinal beclomethasone diprop 100mcg breath-act dry pdr inh

For peer review only

Field ID	Description
25010	Volume of brain, grey+white matter
25008	Volume of white matter
25006	Volume of grey matter
25002	Volume of peripheral cortical grey matter
25004	Volume of ventricular cerebrospinal fluid
25888	Volume of grey matter in Amygdala (left)
25889	Volume of grey matter in Amygdala (right)
25880	Volume of grey matter in Caudate (left)
25881	Volume of grey matter in Caudate (right)
25838	Volume of grey matter in Cingulate Gyrus, anterior division (left)
25839	Volume of grey matter in Cingulate Gyrus, anterior division (right)
25840	Volume of grey matter in Cingulate Gyrus, posterior division (left)
25841	Volume of grey matter in Cingulate Gyrus, posterior division (right)
25886	Volume of grey matter in Hippocampus (left)
25887	Volume of grey matter in Hippocampus (right)
25784	Volume of grey matter in Insular Cortex (left)
25785	Volume of grey matter in Insular Cortex (right)
25788	Volume of grey matter in Middle Frontal Gyrus (left)
25789	Volume of grey matter in Middle Frontal Gyrus (right)
25844	Volume of grey matter in Cuneal Cortex (left)
25845	Volume of grey matter in Cuneal Cortex (right)
25842	Volume of grey matter in Precuneous Cortex (left)
25843	Volume of grey matter in Precuneous Cortex (right)
25900	Volume of grey matter in Crus I Cerebellum (left)
25902	Volume of grey matter in Crus I Cerebellum (right)
25901	Volume of grey matter in Crus I Cerebellum (vermis)
25903	Volume of grey matter in Crus II Cerebellum (left)
25905	Volume of grey matter in Crus II Cerebellum (right)
25904	Volume of grey matter in Crus II Cerebellum (vermis)
25893	Volume of grey matter in I-IV Cerebellum (left)
25894	Volume of grey matter in I-IV Cerebellum (right)
25915	Volume of grey matter in IX Cerebellum (left)
25917	Volume of grey matter in IX Cerebellum (right)
25916	Volume of grey matter in IX Cerebellum (vermis)
25895	Volume of grey matter in V Cerebellum (left)
25896	Volume of grey matter in V Cerebellum (right)
25897	Volume of grey matter in VI Cerebellum (left)
25899	Volume of grey matter in VI Cerebellum (right)
25898	Volume of grey matter in VI Cerebellum (vermis)
25909	Volume of grey matter in VIIIa Cerebellum (left)
25911	Volume of grey matter in VIIIa Cerebellum (right)
25910	Volume of grey matter in VIIIa Cerebellum (vermis)
25912	Volume of grey matter in VIIIb Cerebellum (left)
25914	Volume of grey matter in VIIIb Cerebellum (right)
25913	Volume of grey matter in VIIIb Cerebellum (vermis)

For peer review only

25906	Volume of grey matter in VIIb Cerebellum (left)
25908	Volume of grey matter in VIIb Cerebellum (right)
25907	Volume of grey matter in VIIb Cerebellum (vermis)
25890	Volume of grey matter in Ventral Striatum (left)
25891	Volume of grey matter in Ventral Striatum (right)
25918	Volume of grey matter in X Cerebellum (left)
25919	Volume of grey matter in X Cerebellum (vermis)
25920	Volume of grey matter in X Cerebellum (right)
25023	Volume of accumbens (left)
25024	Volume of accumbens (right)
25021	Volume of amygdala (left)
25022	Volume of amygdala (right)
25013	Volume of caudate (left)
25014	Volume of caudate (right)
25019	Volume of hippocampus (left)
25020	Volume of hippocampus (right)
25017	Volume of pallidum (left)
25018	Volume of pallidum (right)
25015	Volume of putamen (left)
25016	Volume of putamen (right)
25011	Volume of thalamus (left)
25012	Volume of thalamus (right)
25059	Mean FA in body of corpus callosum on FA skeleton
25091	Mean FA in cingulum cingulate gyrus on FA skeleton (left)
25090	Mean FA in cingulum cingulate gyrus on FA skeleton (right)
25093	Mean FA in cingulum hippocampus on FA skeleton (left)
25092	Mean FA in cingulum hippocampus on FA skeleton (right)
25058	Mean FA in genu of corpus callosum on FA skeleton
25060	Mean FA in splenium of corpus callosum on FA skeleton
25101	Mean FA in uncinate fasciculus on FA skeleton (left)
25100	Mean FA in uncinate fasciculus on FA skeleton (right)
25107	Mean MD in body of corpus callosum on FA skeleton
25139	Mean MD in cingulum cingulate gyrus on FA skeleton (left)
25138	Mean MD in cingulum cingulate gyrus on FA skeleton (right)
25141	Mean MD in cingulum hippocampus on FA skeleton (left)
25140	Mean MD in cingulum hippocampus on FA skeleton (right)
25106	Mean MD in genu of corpus callosum on FA skeleton
25108	Mean MD in splenium of corpus callosum on FA skeleton
25149	Mean MD in uncinate fasciculus on FA skeleton (left)
25148	Mean MD in uncinate fasciculus on FA skeleton (right)

For the calculation of the global FA and global MD values, all available mean FA and mean MD were

**Field ID**	**Description**
25079	Mean FA in anterior corona radiata on FA skeleton (left)

For peer review only

25078 Mean FA in anterior corona radiata on FA skeleton (right)
25073 Mean FA in anterior limb of internal capsule on FA skeleton (left)
25072 Mean FA in anterior limb of internal capsule on FA skeleton (right)
25059 Mean FA in body of corpus callosum on FA skeleton
25071 Mean FA in cerebral peduncle on FA skeleton (left)
25070 Mean FA in cerebral peduncle on FA skeleton (right)
25091 Mean FA in cingulum cingulate gyrus on FA skeleton (left)
25090 Mean FA in cingulum cingulate gyrus on FA skeleton (right)
25093 Mean FA in cingulum hippocampus on FA skeleton (left)
25092 Mean FA in cingulum hippocampus on FA skeleton (right)
25063 Mean FA in corticospinal tract on FA skeleton (left)
25062 Mean FA in corticospinal tract on FA skeleton (right)
25089 Mean FA in external capsule on FA skeleton (left)
25088 Mean FA in external capsule on FA skeleton (right)
25095 Mean FA in fornix cres+stria terminalis on FA skeleton (left)
25094 Mean FA in fornix cres+stria terminalis on FA skeleton (right)
25061 Mean FA in fornix on FA skeleton
25058 Mean FA in genu of corpus callosum on FA skeleton
25067 Mean FA in inferior cerebellar peduncle on FA skeleton (left)
25066 Mean FA in inferior cerebellar peduncle on FA skeleton (right)
25065 Mean FA in medial lemniscus on FA skeleton (left)
25064 Mean FA in medial lemniscus on FA skeleton (right)
25056 Mean FA in middle cerebellar peduncle on FA skeleton
25057 Mean FA in pontine crossing tract on FA skeleton
25083 Mean FA in posterior corona radiata on FA skeleton (left)
25082 Mean FA in posterior corona radiata on FA skeleton (right)
25075 Mean FA in posterior limb of internal capsule on FA skeleton (left)
25074 Mean FA in posterior limb of internal capsule on FA skeleton (right)
25085 Mean FA in posterior thalamic radiation on FA skeleton (left)
25084 Mean FA in posterior thalamic radiation on FA skeleton (right)
25077 Mean FA in retrolenticular part of internal capsule on FA skeleton (left)
25076 Mean FA in retrolenticular part of internal capsule on FA skeleton (right)
25087 Mean FA in sagittal stratum on FA skeleton (left)
25086 Mean FA in sagittal stratum on FA skeleton (right)
25060 Mean FA in splenium of corpus callosum on FA skeleton
25069 Mean FA in superior cerebellar peduncle on FA skeleton (left)
25068 Mean FA in superior cerebellar peduncle on FA skeleton (right)
25081 Mean FA in superior corona radiata on FA skeleton (left)
25080 Mean FA in superior corona radiata on FA skeleton (right)
25099 Mean FA in superior fronto-occipital fasciculus on FA skeleton (left)
25098 Mean FA in superior fronto-occipital fasciculus on FA skeleton (right)
25097 Mean FA in superior longitudinal fasciculus on FA skeleton (left)
25096 Mean FA in superior longitudinal fasciculus on FA skeleton (right)
25103 Mean FA in tapetum on FA skeleton (left)
25102 Mean FA in tapetum on FA skeleton (right)
25101 Mean FA in uncinate fasciculus on FA skeleton (left)

For peer review only

25100 Mean FA in uncinate fasciculus on FA skeleton (right)

25127 Mean MD in anterior corona radiata on FA skeleton (left)

25126 Mean MD in anterior corona radiata on FA skeleton (right)

25121 Mean MD in anterior limb of internal capsule on FA skeleton (left)

25120 Mean MD in anterior limb of internal capsule on FA skeleton (right)

25107 Mean MD in body of corpus callosum on FA skeleton

25119 Mean MD in cerebral peduncle on FA skeleton (left)

25118 Mean MD in cerebral peduncle on FA skeleton (right)

25139 Mean MD in cingulum cingulate gyrus on FA skeleton (left)

25138 Mean MD in cingulum cingulate gyrus on FA skeleton (right)

25141 Mean MD in cingulum hippocampus on FA skeleton (left)

25140 Mean MD in cingulum hippocampus on FA skeleton (right)

25111 Mean MD in corticospinal tract on FA skeleton (left)

25110 Mean MD in corticospinal tract on FA skeleton (right)

25137 Mean MD in external capsule on FA skeleton (left)

25136 Mean MD in external capsule on FA skeleton (right)

25143 Mean MD in fornix cres+stria terminalis on FA skeleton (left)

25142 Mean MD in fornix cres+stria terminalis on FA skeleton (right)

25109 Mean MD in fornix on FA skeleton

25106 Mean MD in genu of corpus callosum on FA skeleton

25115 Mean MD in inferior cerebellar peduncle on FA skeleton (left)

25114 Mean MD in inferior cerebellar peduncle on FA skeleton (right)

25113 Mean MD in medial lemniscus on FA skeleton (left)

25112 Mean MD in medial lemniscus on FA skeleton (right)

25104 Mean MD in middle cerebellar peduncle on FA skeleton

25105 Mean MD in pontine crossing tract on FA skeleton

25131 Mean MD in posterior corona radiata on FA skeleton (left)

25130 Mean MD in posterior corona radiata on FA skeleton (right)

25123 Mean MD in posterior limb of internal capsule on FA skeleton (left)

25122 Mean MD in posterior limb of internal capsule on FA skeleton (right)

25133 Mean MD in posterior thalamic radiation on FA skeleton (left)

25132 Mean MD in posterior thalamic radiation on FA skeleton (right)

25125 Mean MD in retrolenticular part of internal capsule on FA skeleton (left)

25124 Mean MD in retrolenticular part of internal capsule on FA skeleton (right)

25135 Mean MD in sagittal stratum on FA skeleton (left)

25134 Mean MD in sagittal stratum on FA skeleton (right)

25108 Mean MD in splenium of corpus callosum on FA skeleton

25117 Mean MD in superior cerebellar peduncle on FA skeleton (left)

25116 Mean MD in superior cerebellar peduncle on FA skeleton (right)

25129 Mean MD in superior corona radiata on FA skeleton (left)

25128 Mean MD in superior corona radiata on FA skeleton (right)

25147 Mean MD in superior fronto-occipital fasciculus on FA skeleton (left)

25146 Mean MD in superior fronto-occipital fasciculus on FA skeleton (right)

25145 Mean MD in superior longitudinal fasciculus on FA skeleton (left)

25144 Mean MD in superior longitudinal fasciculus on FA skeleton (right)

25151 Mean MD in tapetum on FA skeleton (left)

For peer review only

25150 Mean MD in tapetum on FA skeleton (right)
25149 Mean MD in uncinate fasciculus on FA skeleton (left)
25148 Mean MD in uncinate fasciculus on FA skeleton (right)

For peer review only

For peer review only

averaged, respectively:

For peer review only

Codes for the psychi

Field ID	Code
20002	1081
20002	1082
20002	1083
20002	1086
20002	1240
20002	1243
20002	1244
20002	1245
20002	1246
20002	1247
20002	1248
20002	1249
20002	1250
20002	1251
20002	1252
20002	1254
20002	1255
20002	1256
20002	1257
20002	1258
20002	1259
20002	1260
20002	1261
20002	1262
20002	1263
20002	1264
20002	1265
20002	1266
20002	1267
20002	1289
20002	1394
20002	1397
20002	1408
20002	1409
20002	1410
20002	1425
20002	1433
20002	1436
20002	1437
20002	1469
20002	1470
20002	1491

For peer review only

20002	1531
20002	1659
20001	1031
20001	1032
20001	1033
41270	C700
41270	C701
41270	C709
41270	C710
41270	C711
41270	C712
41270	C713
41270	C714
41270	C715
41270	C716
41270	C717
41270	C718
41270	C719
41270	C720
41270	C721
41270	C722
41270	C723
41270	C724
41270	C725
41270	C728
41270	C729
41270	F000
41270	F001
41270	F002
41270	F009
41270	F010
41270	F011
41270	F012
41270	F013
41270	F018
41270	F019
41270	F020
41270	F021
41270	F022
41270	F023
41270	F024
41270	F028
41270	F03
41270	F04
41270	F051
41270	F060

For peer review only

41270 F061
41270 F062
41270 F065
41270 F066
41270 F067
41270 F068
41270 F069
41270 F070
41270 F071
41270 F072
41270 F078
41270 F079
41270 F09
41270 F100
41270 F101
41270 F102
41270 F103
41270 F104
41270 F105
41270 F106
41270 F107
41270 F108
41270 F109
41270 F110
41270 F111
41270 F112
41270 F113
41270 F114
41270 F115
41270 F117
41270 F119
41270 F120
41270 F121
41270 F122
41270 F123
41270 F125
41270 F128
41270 F129
41270 F130
41270 F131
41270 F132
41270 F133
41270 F134
41270 F139
41270 F140
41270 F141

For peer review only

41270	F142
41270	F145
41270	F149
41270	F150
41270	F151
41270	F152
41270	F153
41270	F155
41270	F158
41270	F159
41270	F161
41270	F162
41270	F163
41270	F165
41270	F167
41270	F168
41270	F169
41270	F170
41270	F171
41270	F172
41270	F173
41270	F174
41270	F177
41270	F179
41270	F181
41270	F182
41270	F183
41270	F185
41270	F189
41270	F190
41270	F191
41270	F192
41270	F193
41270	F194
41270	F195
41270	F198
41270	F199
41270	F200
41270	F201
41270	F202
41270	F203
41270	F204
41270	F205
41270	F206
41270	F208
41270	F209

For peer review only

41270 F21
41270 F220
41270 F228
41270 F229
41270 F230
41270 F231
41270 F232
41270 F233
41270 F238
41270 F239
41270 F24
41270 F250
41270 F251
41270 F252
41270 F258
41270 F259
41270 F28
41270 F29
41270 F431
41270 F432
41270 F438
41270 F439
41270 F440
41270 F441
41270 F442
41270 F443
41270 F444
41270 F445
41270 F446
41270 F447
41270 F448
41270 F449
41270 F450
41270 F451
41270 F452
41270 F453
41270 F454
41270 F458
41270 F459
41270 F480
41270 F481
41270 F488
41270 F489
41270 F500
41270 F501
41270 F502

For peer review only

41270 F505
41270 F508
41270 F509
41270 F530
41270 F531
41270 F538
41270 F539
41270 F54
41270 F55
41270 F59
41270 F600
41270 F601
41270 F602
41270 F603
41270 F604
41270 F605
41270 F606
41270 F607
41270 F608
41270 F609
41270 F61
41270 F620
41270 F621
41270 F628
41270 F629
41270 F630
41270 F631
41270 F632
41270 F633
41270 F638
41270 F639
41270 F680
41270 F681
41270 F688
41270 F69
41270 F700
41270 F701
41270 F708
41270 F709
41270 F711
41270 F719
41270 F729
41270 F780
41270 F789
41270 F790
41270 F798

For peer review only

41270 F799
41270 F800
41270 F801
41270 F802
41270 F803
41270 F809
41270 F810
41270 F812
41270 F819
41270 F82
41270 F83
41270 F840
41270 F841
41270 F843
41270 F844
41270 F845
41270 F849
41270 F89
41270 F900
41270 F909
41270 F911
41270 F918
41270 F919
41270 F920
41270 F929
41270 F930
41270 F940
41270 F941
41270 F950
41270 F951
41270 F952
41270 F958
41270 F959
41270 F981
41270 F985
41270 F986
41270 F988
41270 F99
41270 G000
41270 G001
41270 G002
41270 G003
41270 G008
41270 G009
41270 G01
41270 G020

For peer review only

41270 G021
41270 G030
41270 G031
41270 G032
41270 G038
41270 G039
41270 G040
41270 G042
41270 G048
41270 G049
41270 G050
41270 G051
41270 G052
41270 G058
41270 G060
41270 G061
41270 G062
41270 G07
41270 G08
41270 G09
41270 G10
41270 G110
41270 G111
41270 G112
41270 G113
41270 G114
41270 G118
41270 G119
41270 G120
41270 G121
41270 G122
41270 G128
41270 G129
41270 G130
41270 G131
41270 G138
41270 G14
41270 G20
41270 G210
41270 G211
41270 G212
41270 G213
41270 G214
41270 G218
41270 G219
41270 G22

For peer review only

41270 G230
41270 G231
41270 G232
41270 G233
41270 G238
41270 G239
41270 G240
41270 G241
41270 G242
41270 G243
41270 G244
41270 G245
41270 G248
41270 G249
41270 G250
41270 G251
41270 G252
41270 G253
41270 G254
41270 G255
41270 G258
41270 G259
41270 G300
41270 G301
41270 G308
41270 G309
41270 G310
41270 G311
41270 G312
41270 G318
41270 G319
41270 G320
41270 G328
41270 G35
41270 G360
41270 G368
41270 G369
41270 G370
41270 G371
41270 G372
41270 G373
41270 G374
41270 G378
41270 G379
41270 G400
41270 G401

For peer review only

41270 G402
41270 G403
41270 G404
41270 G405
41270 G406
41270 G407
41270 G408
41270 G409
41270 G410
41270 G411
41270 G412
41270 G418
41270 G419
41270 G430
41270 G431
41270 G432
41270 G433
41270 G438
41270 G439
41270 G440
41270 G441
41270 G442
41270 G443
41270 G444
41270 G448
41270 G450
41270 G451
41270 G452
41270 G453
41270 G454
41270 G458
41270 G459
41270 G460
41270 G461
41270 G462
41270 G463
41270 G464
41270 G465
41270 G466
41270 G467
41270 G468
41270 G474
41270 G500
41270 G501
41270 G508
41270 G509

For peer review only

41270 G510
41270 G511
41270 G512
41270 G513
41270 G514
41270 G518
41270 G519
41270 G520
41270 G521
41270 G522
41270 G523
41270 G527
41270 G528
41270 G529
41270 G530
41270 G531
41270 G532
41270 G533
41270 G538
41270 G540
41270 G541
41270 G542
41270 G543
41270 G544
41270 G545
41270 G546
41270 G547
41270 G548
41270 G549
41270 G550
41270 G551
41270 G552
41270 G553
41270 G558
41270 G560
41270 G561
41270 G562
41270 G563
41270 G564
41270 G568
41270 G569
41270 G570
41270 G571
41270 G572
41270 G573
41270 G574

For peer review only

41270 G575
41270 G576
41270 G578
41270 G579
41270 G580
41270 G587
41270 G588
41270 G589
41270 G590
41270 G598
41270 G600
41270 G602
41270 G603
41270 G608
41270 G609
41270 G610
41270 G611
41270 G618
41270 G619
41270 G620
41270 G621
41270 G622
41270 G628
41270 G629
41270 G630
41270 G631
41270 G632
41270 G633
41270 G634
41270 G635
41270 G636
41270 G638
41270 G64
41270 G700
41270 G702
41270 G708
41270 G709
41270 G710
41270 G711
41270 G712
41270 G713
41270 G718
41270 G719
41270 G720
41270 G721
41270 G722

For peer review only

41270 G723
41270 G724
41270 G728
41270 G729
41270 G730
41270 G731
41270 G735
41270 G736
41270 G737
41270 G800
41270 G801
41270 G802
41270 G803
41270 G808
41270 G809
41270 G810
41270 G811
41270 G819
41270 G820
41270 G821
41270 G822
41270 G823
41270 G824
41270 G825
41270 G830
41270 G831
41270 G832
41270 G833
41270 G834
41270 G835
41270 G838
41270 G839
41270 G900
41270 G901
41270 G902
41270 G903
41270 G904
41270 G908
41270 G909
41270 G910
41270 G911
41270 G912
41270 G913
41270 G918
41270 G919
41270 G92

For peer review only

41270 G930
41270 G931
41270 G932
41270 G933
41270 G934
41270 G935
41270 G936
41270 G937
41270 G938
41270 G939
41270 G940
41270 G941
41270 G942
41270 G948
41270 G950
41270 G951
41270 G952
41270 G958
41270 G959
41270 G960
41270 G961
41270 G968
41270 G969
41270 G970
41270 G971
41270 G972
41270 G978
41270 G979
41270 G98
41270 G990
41270 G991
41270 G992
41270 G998
41270 S0200
41270 S0201
41270 S0210
41270 S0211
41270 S0270
41270 S0271
41270 S0280
41270 S9281
41270 S0290
41270 S0291
41270 S040
41270 S041
41270 S042

For peer review only

41270 S043
41270 S045
41270 S048
41270 S049
41270 S0600
41270 S0601
41270 S0610
41270 S0620
41270 S0621
41270 S0630
41270 S0631
41270 S0640
41270 S0641
41270 S0650
41270 S0651
41270 S0660
41270 S0661
41270 S0670
41270 S0680
41270 S0681
41270 S0690
41270 S0691
41270 S097
41270 S099
41270 T901
41270 T902
41270 T903
41270 T904
41270 T908
41270 T909
41270 T913

For peer review only

atric and neurological conditions used as exclusion criteria

**Description**
stroke
transient ischaemic attack (tia)
subdural haemorrhage/haematoma
subarachnoid haemorrhage
neurological injury/trauma
psychological/psychiatric problem
infection of nervous system
brain abscess/intracranial abscess
encephalitis
meningitis
spinal abscess
cranial nerve problem/palsy
bell's palsy/facial nerve palsy
spinal cord disorder
paraplegia
peripheral nerve disorder
peripheral neuropathy
acute infective polyneuritis/guillain-barre syndrome
trapped nerve/compressed nerve
chronic/degenerative neurological problem
motor neuron disease
myasthenia gravis
multiple sclerosis
Parkinson’s disease
dementia/Alzheimer’s/cognitive impairment
epilepsy
migraine
head injury
spinal injury
schizophrenia
peripheral nerve injury
other demyelinating disease (not multiple sclerosis)
alcohol dependency
opioid dependency
other substance abuse/dependency
cerebral aneurysm
cerebral palsy
headaches (not migraine)
myasthenia gravis
post-traumatic stress disorder
anorexia/bulimia/other eating disorder
brain haemorrhage

For peer review only

post-natal depression
meningioma / benign meningeal tumour
meningeal cancer / malignant meningioma
brain cancer / primary malignant brain tumour
spinal cord or cranial nerve cancer
C70.0 Cerebral meninges
C70.1 Spinal meninges
C70.9 Meninges, unspecified
C71.0 Cerebrum, except lobes and ventricles
C71.1 Frontal lobe
C71.2 Temporal lobe
C71.3 Parietal lobe
C71.4 Occipital lobe
C71.5 Cerebral ventricle
C71.6 Cerebellum
C71.7 Brain stem
C71.8 Overlapping lesion of brain
C71.9 Brain, unspecified
C72.0 Spinal cord
C72.1 Cauda equina
C72.2 Olfactory nerve
C72.3 Optic nerve
C72.4 Acoustic nerve
C72.5 Other and unspecified cranial nerves
C72.8 Overlapping lesion of brain and other parts of central nervous system
C72.9 Central nervous system, unspecified
F00.0 Dementia in Alzheimer's disease with early onset
F00.1 Dementia in Alzheimer's disease with late onset
F00.2 Dementia in Alzheimer's disease, atypical or mixed type
F00.9 Dementia in Alzheimer's disease, unspecified
F01.0 Vascular dementia of acute onset
F01.1 Multi-infarct dementia
F01.2 Subcortical vascular dementia
F01.3 Mixed cortical and subcortical vascular dementia
F01.8 Other vascular dementia
F01.9 Vascular dementia, unspecified
F02.0 Dementia in Pick's disease
F02.1 Dementia in Creutzfeldt-Jakob disease
F02.2 Dementia in Huntington's disease
F02.3 Dementia in Parkinson's disease
F02.4 Dementia in human immunodeficiency virus [HIV] disease
F02.8 Dementia in other specified diseases classified elsewhere
F03 Unspecified dementia
F04 Organic amnesic syndrome, not induced by alcohol and other psychoactive substances
F05.1 Delirium superimposed on dementia
F06.0 Organic hallucinosis

For peer review only

F06.1 Organic catatonic disorder
F06.2 Organic delusional [schizophrenia-like] disorder
F06.5 Organic dissociative disorder
F06.6 Organic emotionally labile [asthenic] disorder
F06.7 Mild cognitive disorder
F06.8 Other specified mental disorders due to brain damage and dysfunction and to physical disease
F06.9 Unspecified mental disorder due to brain damage and dysfunction and to physical disease
F07.0 Organic personality disorder
F07.1 Postencephalitic syndrome
F07.2 Postconcussional syndrome
F07.8 Other organic personality and behavioural disorders due to brain disease, damage and dysfunct
F07.9 Unspecified organic personality and behavioural disorder due to brain disease, damage and dys
F09 Unspecified organic or symptomatic mental disorder
F10.0 Acute intoxication
F10.1 Harmful use
F10.2 Dependence syndrome
F10.3 Withdrawal state
F10.4 Withdrawal state with delirium
F10.5 Psychotic disorder
F10.6 Amnesic syndrome
F10.7 Residual and late-onset psychotic disorder
F10.8 Other mental and behavioural disorders
F10.9 Unspecified mental and behavioural disorder
F11.0 Acute intoxication
F11.1 Harmful use
F11.2 Dependence syndrome
F11.3 Withdrawal state
F11.4 Withdrawal state with delirium
F11.5 Psychotic disorder
F11.7 Residual and late-onset psychotic disorder
F11.9 Unspecified mental and behavioural disorder
F12.0 Acute intoxication
F12.1 Harmful use
F12.2 Dependence syndrome
F12.3 Withdrawal state
F12.5 Psychotic disorder
F12.8 Other mental and behavioural disorders
F12.9 Unspecified mental and behavioural disorder
F13.0 Acute intoxication
F13.1 Harmful use
F13.2 Dependence syndrome
F13.3 Withdrawal state
F13.4 Withdrawal state with delirium
F13.9 Unspecified mental and behavioural disorder
F14.0 Acute intoxication
F14.1 Harmful use

For peer review only

F14.2 Dependence syndrome
F14.5 Psychotic disorder
F14.9 Unspecified mental and behavioural disorder
F15.0 Acute intoxication
F15.1 Harmful use
F15.2 Dependence syndrome
F15.3 Withdrawal state
F15.5 Psychotic disorder
F15.8 Other mental and behavioural disorders
F15.9 Unspecified mental and behavioural disorder
F16.1 Harmful use
F16.2 Dependence syndrome
F16.3 Withdrawal state
F16.5 Psychotic disorder
F16.7 Residual and late-onset psychotic disorder
F16.8 Other mental and behavioural disorders
F16.9 Unspecified mental and behavioural disorder
F17.0 Acute intoxication
F17.1 Harmful use
F17.2 Dependence syndrome
F17.3 Withdrawal state
F17.4 Withdrawal state with delirium
F17.7 Residual and late-onset psychotic disorder
F17.9 Unspecified mental and behavioural disorder
F18.1 Harmful use
F18.2 Dependence syndrome
F18.3 Withdrawal state
F18.5 Psychotic disorder
F18.9 Unspecified mental and behavioural disorder
F19.0 Acute intoxication
F19.1 Harmful use
F19.2 Dependence syndrome
F19.3 Withdrawal state
F19.4 Withdrawal state with delirium
F19.5 Psychotic disorder
F19.8 Other mental and behavioural disorders
F19.9 Unspecified mental and behavioural disorder
F20.0 Paranoid schizophrenia
F20.1 Hebephrenic schizophrenia
F20.2 Catatonic schizophrenia
F20.3 Undifferentiated schizophrenia
F20.4 Postschizophrenic depression
F20.5 Residual schizophrenia
F20.6 Simple schizophrenia
F20.8 Other schizophrenia
F20.9 Schizophrenia, unspecified

For peer review only

F21 Schizotypal disorder
F22.0 Delusional disorder
F22.8 Other persistent delusional disorders
F22.9 Persistent delusional disorder, unspecified
F23.0 Acute polymorphic psychotic disorder without symptoms of schizophrenia
F23.1 Acute polymorphic psychotic disorder with symptoms of schizophrenia
F23.2 Acute schizophrenia-like psychotic disorder
F23.3 Other acute predominantly delusional psychotic disorders
F23.8 Other acute and transient psychotic disorders
F23.9 Acute and transient psychotic disorder, unspecified
F24 Induced delusional disorder
F25.0 Schizoaffective disorder, manic type
F25.1 Schizoaffective disorder, depressive type
F25.2 Schizoaffective disorder, mixed type
F25.8 Other schizoaffective disorders
F25.9 Schizoaffective disorder, unspecified
F28 Other nonorganic psychotic disorders
F29 Unspecified nonorganic psychosis
F43.1 Posttraumatic stress disorder
F43.2 Adjustment disorders
F43.8 Other reactions to severe stress
F43.9 Reaction to severe stress, unspecified
F44.0 Dissociative amnesia
F44.1 Dissociative fugue
F44.2 Dissociative stupor
F44.3 Trance and possession disorders
F44.4 Dissociative motor disorders
F44.5 Dissociative convulsions
F44.6 Dissociative anaesthesia and sensory loss
F44.7 Mixed dissociative [conversion] disorders
F44.8 Other dissociative [conversion] disorders
F44.9 Dissociative [conversion] disorder, unspecified
F45.0 Somatisation disorder
F45.1 Undifferentiated somatoform disorder
F45.2 Hypochondriacal disorder
F45.3 Somatoform autonomic dysfunction
F45.4 Persistent somatoform pain disorder
F45.8 Other somatoform disorders
F45.9 Somatoform disorder, unspecified
F48.0 Neurasthenia
F48.1 Depersonalisation-derealisation syndrome
F48.8 Other specified neurotic disorders
F48.9 Neurotic disorder, unspecified
F50.0 Anorexia nervosa
F50.1 Atypical anorexia nervosa
F50.2 Bulimia nervosa

For peer review only

F50.5 Vomiting associated with other psychological disturbances
F50.8 Other eating disorders
F50.9 Eating disorder, unspecified
F53.0 Mild mental and behavioural disorders associated with the puerperium, not elsewhere classifie
F53.1 Severe mental and behavioural disorders associated with the puerperium, not elsewhere classif
F53.8 Other mental and behavioural disorders associated with the puerperium, not elsewhere classifi
F53.9 Puerperal mental disorder, unspecified
F54 Psychological and behavioural factors associated with disorders or diseases classified elsewhere
F55 Abuse of non-dependence-producing substances
F59 Unspecified behavioural syndromes associated with physiological disturbances and physical facto
F60.0 Paranoid personality disorder
F60.1 Schizoid personality disorder
F60.2 Dissocial personality disorder
F60.3 Emotionally unstable personality disorder
F60.4 Histrionic personality disorder
F60.5 Anankastic personality disorder
F60.6 Anxious [avoidant] personality disorder
F60.7 Dependent personality disorder
F60.8 Other specific personality disorders
F60.9 Personality disorder, unspecified
F61 Mixed and other personality disorders
F62.0 Enduring personality change after catastrophic experience
F62.1 Enduring personality change after psychiatric illness
F62.8 Other enduring personality changes
F62.9 Enduring personality change, unspecified
F63.0 Pathological gambling
F63.1 Pathological fire-setting [pyromania]
F63.2 Pathological stealing [kleptomania]
F63.3 Trichotillomania
F63.8 Other habit and impulse disorders
F63.9 Habit and impulse disorder, unspecified
F68.0 Elaboration of physical symptoms for psychological reasons
F68.1 Intentional production or feigning of symptoms or disabilities, either physical or psychological [
F68.8 Other specified disorders of adult personality and behaviour
F69 Unspecified disorder of adult personality and behaviour
F70.0 Mild mental retardation (With the statement of no, or minimal, impairment of behaviour)
F70.1 Mild mental retardation (Significant impairment of behaviour requiring attention or treatment)
F70.8 Mild mental retardation (Other impairments of behaviour)
F70.9 Mild mental retardation (Without mention of impairment of behaviour)
F71.1 Moderate mental retardation (Significant impairment of behaviour requiring attention or treatr
F71.9 Moderate mental retardation (Without mention of impairment of behaviour)
F72.9 Severe mental retardation (Without mention of impairment of behaviour)
F78.0 Other mental retardation (With the statement of no, or minimal, impairment of behaviour)
F78.9 Other mental retardation (Without mention of impairment of behaviour)
F79.0 Unspecified mental retardation (With the statement of no, or minimal, impairment of behaviou
F79.8 Unspecified mental retardation (Other impairments of behaviour)

For peer review only

F79.9 Unspecified mental retardation (Without mention of impairment of behaviour)
F80.0 Specific speech articulation disorder
F80.1 Expressive language disorder
F80.2 Receptive language disorder
F80.3 Acquired aphasia with epilepsy [Landau-Kleffner]
F80.9 Developmental disorder of speech and language, unspecified
F81.0 Specific reading disorder
F81.2 Specific disorder of arithmetical skills
F81.9 Developmental disorder of scholastic skills, unspecified
F82 Specific developmental disorder of motor function
F83 Mixed specific developmental disorders
F84.0 Childhood autism
F84.1 Atypical autism
F84.3 Other childhood disintegrative disorder
F84.4 Overactive disorder associated with mental retardation and stereotyped movements
F84.5 Asperger's syndrome
F84.9 Pervasive developmental disorder, unspecified
F89 Unspecified disorder of psychological development
F90.0 Disturbance of activity and attention
F90.9 Hyperkinetic disorder, unspecified
F91.1 Unsocialised conduct disorder
F91.8 Other conduct disorders
F91.9 Conduct disorder, unspecified
F92.0 Depressive conduct disorder
F92.9 Mixed disorder of conduct and emotions, unspecified
F93.0 Separation anxiety disorder of childhood
F94.0 Elective mutism
F94.1 Reactive attachment disorder of childhood
F95.0 Transient tic disorder
F95.1 Chronic motor or vocal tic disorder
F95.2 Combined vocal and multiple motor tic disorder [de la Tourette]
F95.8 Other tic disorders
F95.9 Tic disorder, unspecified
F98.1 Nonorganic encopresis
F98.5 Stuttering [stammering]
F98.6 Cluttering
F98.8 Other specified behavioural and emotional disorders with onset usually occurring in childhood [illegible]
F99 Mental disorder, not otherwise specified
G00.0 Haemophilus meningitis
G00.1 Pneumococcal meningitis
G00.2 Streptococcal meningitis
G00.3 Staphylococcal meningitis
G00.8 Other bacterial meningitis
G00.9 Bacterial meningitis, unspecified
G01 Meningitis in bacterial diseases classified elsewhere
G02.0 Meningitis in viral diseases classified elsewhere

For peer review only

G02.1 Meningitis in mycoses
G03.0 Nonpyogenic meningitis
G03.1 Chronic meningitis
G03.2 Benign recurrent meningitis [Mollaret]
G03.8 Meningitis due to other specified causes
G03.9 Meningitis, unspecified
G04.0 Acute disseminated encephalitis
G04.2 Bacterial meningoencephalitis and meningomyelitis, not elsewhere classified
G04.8 Other encephalitis, myelitis and encephalomyelitis
G04.9 Encephalitis, myelitis and encephalomyelitis, unspecified
G05.0 Encephalitis, myelitis and encephalomyelitis in bacterial diseases classified elsewhere
G05.1 Encephalitis, myelitis and encephalomyelitis in viral diseases classified elsewhere
G05.2 Encephalitis, myelitis and encephalomyelitis in other infectious and parasitic diseases classified
G05.8 Encephalitis, myelitis and encephalomyelitis in other diseases classified elsewhere
G06.0 Intracranial abscess and granuloma
G06.1 Intraspinal abscess and granuloma
G06.2 Extradural and subdural abscess, unspecified
G07 Intracranial and intraspinal abscess and granuloma in diseases classified elsewhere
G08 Intracranial and intraspinal phlebitis and thrombophlebitis
G09 Sequelae of inflammatory diseases of central nervous system
G10 Huntington's disease
G11.0 Congenital nonprogressive ataxia
G11.1 Early-onset cerebellar ataxia
G11.2 Late-onset cerebellar ataxia
G11.3 Cerebellar ataxia with defective DNA repair
G11.4 Hereditary spastic paraplegia
G11.8 Other hereditary ataxias
G11.9 Hereditary ataxia, unspecified
G12.0 Infantile spinal muscular atrophy, type I [Werdnig-Hoffman]
G12.1 Other inherited spinal muscular atrophy
G12.2 Motor neuron disease
G12.8 Other spinal muscular atrophies and related syndromes
G12.9 Spinal muscular atrophy, unspecified
G13.0 Paraneoplastic neuromyopathy and neuropathy
G13.1 Other systemic atrophy primarily affecting central nervous system in neoplastic disease
G13.8 Systemic atrophy primarily affecting central nervous system in other diseases classified elsewh
G14 Postpolio syndrome
G20 Parkinson's disease
G21.0 Malignant neuroleptic syndrome
G21.1 Other drug-induced secondary Parkinsonism
G21.2 Secondary Parkinsonism due to other external agents
G21.3 Postencephalitic Parkinsonism
G21.4 Vascular parkinsonism
G21.8 Other secondary Parkinsonism
G21.9 Secondary Parkinsonism, unspecified
G22 Parkinsonism in diseases classified elsewhere

For peer review only

G23.0 Hallervorden-Spatz disease
G23.1 Progressive supranuclear ophthalmoplegia [Steele-Richardson-Olszewski]
G23.2 Striatonigral degeneration
G23.3 Multiple system atrophy, cerebellar type
G23.8 Other specified degenerative diseases of basal ganglia
G23.9 Degenerative disease of basal ganglia, unspecified
G24.0 Drug-induced dystonia
G24.1 Idiopathic familial dystonia
G24.2 Idiopathic nonfamilial dystonia
G24.3 Spasmodic torticollis
G24.4 Idiopathic orofacial dystonia
G24.5 Blepharospasm
G24.8 Other dystonia
G24.9 Dystonia, unspecified
G25.0 Essential tremor
G25.1 Drug-induced tremor
G25.2 Other specified forms of tremor
G25.3 Myoclonus
G25.4 Drug-induced chorea
G25.5 Other chorea
G25.8 Other specified extrapyramidal and movement disorders
G25.9 Extrapyramidal and movement disorder, unspecified
G30.0 Alzheimer's disease with early onset
G30.1 Alzheimer's disease with late onset
G30.8 Other Alzheimer's disease
G30.9 Alzheimer's disease, unspecified
G31.0 Circumscribed brain atrophy
G31.1 Senile degeneration of brain, not elsewhere classified
G31.2 Degeneration of nervous system due to alcohol
G31.8 Other specified degenerative diseases of nervous system
G31.9 Degenerative disease of nervous system, unspecified
G32.0 Subacute combined degeneration of spinal cord in diseases classified elsewhere
G32.8 Other specified degenerative disorders of nervous system in diseases classified elsewhere
G35 Multiple sclerosis
G36.0 Neuromyelitis optica [Devic]
G36.8 Other specified acute disseminated demyelination
G36.9 Acute disseminated demyelination, unspecified
G37.0 Diffuse sclerosis
G37.1 Central demyelination of corpus callosum
G37.2 Central pontine myelinolysis
G37.3 Acute transverse myelitis in demyelinating disease of central nervous system
G37.4 Subacute necrotising myelitis
G37.8 Other specified demyelinating diseases of central nervous system
G37.9 Demyelinating disease of central nervous system, unspecified
G40.0 Localisation-related (focal) (partial) idiopathic epilepsy and epileptic syndromes with seizures o
G40.1 Localisation-related (focal) (partial) symptomatic epilepsy and epileptic syndromes with simple

For peer review only

G40.2 Localisation-related (focal) (partial) symptomatic epilepsy and epileptic syndromes with comple
G40.3 Generalised idiopathic epilepsy and epileptic syndromes
G40.4 Other generalised epilepsy and epileptic syndromes
G40.5 Special epileptic syndromes
G40.6 Grand mal seizures, unspecified (with or without petit mal)
G40.7 Petit mal, unspecified, without grand mal seizures
G40.8 Other epilepsy
G40.9 Epilepsy, unspecified
G41.0 Grand mal status epilepticus
G41.1 Petit mal status epilepticus
G41.2 Complex partial status epilepticus
G41.8 Other status epilepticus
G41.9 Status epilepticus, unspecified
G43.0 Migraine without aura [common migraine]
G43.1 Migraine with aura [classical migraine]
G43.2 Status migrainosus
G43.3 Complicated migraine
G43.8 Other migraine
G43.9 Migraine, unspecified
G44.0 Cluster headache syndrome
G44.1 Vascular headache, not elsewhere classified
G44.2 Tension-type headache
G44.3 Chronic posttraumatic headache
G44.4 Drug-induced headache, not elsewhere classified
G44.8 Other specified headache syndromes
G45.0 Vertebro-basilar artery syndrome
G45.1 Carotid artery syndrome (hemispheric)
G45.2 Multiple and bilateral precerebral artery syndromes
G45.3 Amaurosis fugax
G45.4 Transient global amnesia
G45.8 Other transient cerebral ischaemic attacks and related syndromes
G45.9 Transient cerebral ischaemic attack, unspecified
G46.0 Middle cerebral artery syndrome
G46.1 Anterior cerebral artery syndrome
G46.2 Posterior cerebral artery syndrome
G46.3 Brain stem stroke syndrome
G46.4 Cerebellar stroke syndrome
G46.5 Pure motor lacunar syndrome
G46.6 Pure sensory lacunar syndrome
G46.7 Other lacunar syndromes
G46.8 Other vascular syndromes of brain in cerebrovascular diseases
G47.4 Narcolepsy and cataplexy
G50.0 Trigeminal neuralgia
G50.1 Atypical facial pain
G50.8 Other disorders of trigeminal nerve
G50.9 Disorder of trigeminal nerve, unspecified

For peer review only

G51.0 Bell's palsy
G51.1 Geniculate ganglionitis
G51.2 Melkersson's syndrome
G51.3 Clonic hemifacial spasm
G51.4 Facial myokymia
G51.8 Other disorders of facial nerve
G51.9 Disorder of facial nerve, unspecified
G52.0 Disorders of olfactory nerve
G52.1 Disorders of glossopharyngeal nerve
G52.2 Disorders of vagus nerve
G52.3 Disorders of hypoglossal nerve
G52.7 Disorders of multiple cranial nerves
G52.8 Disorders of other specified cranial nerves
G52.9 Cranial nerve disorder, unspecified
G53.0 Postzoster neuralgia
G53.1 Multiple cranial nerve palsies in infectious and parasitic diseases classified elsewhere
G53.2 Multiple cranial nerve palsies in sarcoidosis
G53.3 Multiple cranial nerve palsies in neoplastic disease
G53.8 Other cranial nerve disorders in other diseases classified elsewhere
G54.0 Brachial plexus disorders
G54.1 Lumbosacral plexus disorders
G54.2 Cervical root disorders, not elsewhere classified
G54.3 Thoracic root disorders, not elsewhere classified
G54.4 Lumbosacral root disorders, not elsewhere classified
G54.5 Neuralgic amyotrophy
G54.6 Phantom limb syndrome with pain
G54.7 Phantom limb syndrome without pain
G54.8 Other nerve root and plexus disorders
G54.9 Nerve root and plexus disorder, unspecified
G55.0 Nerve root and plexus compressions in neoplastic disease
G55.1 Nerve root and plexus compressions in intervertebral disk disorders
G55.2 Nerve root and plexus compressions in spondylosis
G55.3 Nerve root and plexus compressions in other dorsopathies
G55.8 Nerve root and plexus compressions in other diseases classified elsewhere
G56.0 Carpal tunnel syndrome
G56.1 Other lesions of median nerve
G56.2 Lesion of ulnar nerve
G56.3 Lesion of radial nerve
G56.4 Causalgia
G56.8 Other mononeuropathies of upper limb
G56.9 Mononeuropathy of upper limb, unspecified
G57.0 Lesion of sciatic nerve
G57.1 Meralgia paraesthetica
G57.2 Lesion of femoral nerve
G57.3 Lesion of lateral popliteal nerve
G57.4 Lesion of medial popliteal nerve

For peer review only

G57.5 Tarsal tunnel syndrome
G57.6 Lesion of plantar nerve
G57.8 Other mononeuropathies of lower limb
G57.9 Mononeuropathy of lower limb, unspecified
G58.0 Intercostal neuropathy
G58.7 Mononeuritis multiplex
G58.8 Other specified mononeuropathies
G58.9 Mononeuropathy, unspecified
G59.0 Diabetic mononeuropathy
G59.8 Other mononeuropathies in diseases classified elsewhere
G60.0 Hereditary motor and sensory neuropathy
G60.2 Neuropathy in association with hereditary ataxia
G60.3 Idiopathic progressive neuropathy
G60.8 Other hereditary and idiopathic neuropathies
G60.9 Hereditary and idiopathic neuropathy, unspecified
G61.0 Guillain-Barre syndrome
G61.1 Serum neuropathy1
G61.8 Other inflammatory polyneuropathies
G61.9 Inflammatory polyneuropathy, unspecified
G62.0 Drug-induced polyneuropathy
G62.1 Alcoholic polyneuropathy
G62.2 Polyneuropathy due to other toxic agents
G62.8 Other specified polyneuropathies
G62.9 Polyneuropathy, unspecified
G63.0 Polyneuropathy in infectious and parasitic diseases classified elsewhere
G63.1 Polyneuropathy in neoplastic disease
G63.2 Diabetic polyneuropathy
G63.3 Polyneuropathy in other endocrine and metabolic diseases
G63.4 Polyneuropathy in nutritional deficiency
G63.5 Polyneuropathy in systemic connective tissue disorders
G63.6 Polyneuropathy in other musculoskeletal disorders
G63.8 Polyneuropathy in other diseases classified elsewhere
G64 Other disorders of peripheral nervous system
G70.0 Myasthenia gravis
G70.2 Congenital and developmental myasthenia
G70.8 Other specified myoneural disorders
G70.9 Myoneural disorder, unspecified
G71.0 Muscular dystrophy
G71.1 Myotonic disorders
G71.2 Congenital myopathies
G71.3 Mitochondrial myopathy, not elsewhere classified
G71.8 Other primary disorders of muscles
G71.9 Primary disorder of muscle, unspecified
G72.0 Drug-induced myopathy
G72.1 Alcoholic myopathy
G72.2 Myopathy due to other toxic agents

For peer review only

G72.3 Periodic paralysis
G72.4 Inflammatory myopathy, not elsewhere classified
G72.8 Other specified myopathies
G72.9 Myopathy, unspecified
G73.0 Myasthenic syndromes in endocrine diseases
G73.1 Eaton-Lambert syndrome
G73.5 Myopathy in endocrine diseases
G73.6 Myopathy in metabolic diseases
G73.7 Myopathy other diseases classified elsewhere
G80.0 Spastic cerebral palsy
G80.1 Spastic diplegia
G80.2 Infantile hemiplegia
G80.3 Dyskinetic cerebral palsy
G80.8 Other infantile cerebral palsy
G80.9 Infantile cerebral palsy, unspecified
G81.0 Flaccid hemiplegia
G81.1 Spastic hemiplegia
G81.9 Hemiplegia, unspecified
G82.0 Flaccid paraplegia
G82.1 Spastic paraplegia
G82.2 Paraplegia, unspecified
G82.3 Flaccid tetraplegia
G82.4 Spastic tetraplegia
G82.5 Tetraplegia, unspecified
G83.0 Diplegia of upper limbs
G83.1 Monoplegia of lower limb
G83.2 Monoplegia of upper limb
G83.3 Monoplegia, unspecified
G83.4 Cauda equina syndrome
G83.5 Locked-in syndrome
G83.8 Other specified paralytic syndromes
G83.9 Paralytic syndrome, unspecified
G90.0 Idiopathic peripheral autonomic neuropathy
G90.1 Familial dysautonomia [Riley-Day]
G90.2 Horner's syndrome
G90.3 Multisystem degeneration
G90.4 Autonomic dysreflexia
G90.8 Other disorders of autonomic nervous system
G90.9 Disorder of autonomic nervous system, unspecified
G91.0 Communicating hydrocephalus
G91.1 Obstructive hydrocephalus
G91.2 Normal-pressure hydrocephalus
G91.3 Posttraumatic hydrocephalus, unspecified
G91.8 Other hydrocephalus
G91.9 Hydrocephalus, unspecified
G92 Toxic encephalopathy

For peer review only

G93.0 Cerebral cysts
G93.1 Anoxic brain damage, not elsewhere classified
G93.2 Benign intracranial hypertension
G93.3 Postviral fatigue syndrome
G93.4 Encephalopathy, unspecified
G93.5 Compression of brain
G93.6 Cerebral oedema
G93.7 Reye's syndrome
G93.8 Other specified disorders of brain
G93.9 Disorder of brain, unspecified
G94.0 Hydrocephalus in infectious and parasitic diseases classified elsewhere
G94.1 Hydrocephalus in neoplastic disease
G94.2 Hydrocephalus in other diseases classified elsewhere
G94.8 Other specified disorders of brain in diseases classified elsewhere
G95.0 Syringomyelia and syringobulbia
G95.1 Vascular myelopathies
G95.2 Cord compression, unspecified
G95.8 Other specified diseases of spinal cord
G95.9 Disease of spinal cord, unspecified
G96.0 Cerebrospinal fluid leak
G96.1 Disorders of meninges, not elsewhere classified
G96.8 Other specified disorders of central nervous system
G96.9 Disorder of central nervous system, unspecified
G97.0 Cerebrospinal fluid leak from spinal puncture
G97.1 Other reaction to spinal and lumbar puncture
G97.2 Intracranial hypotension following ventricular shunting
G97.8 Other postprocedural disorders of nervous system
G97.9 Postprocedural disorder of nervous system, unspecified
G98 Other disorders of nervous system, not elsewhere classified
G99.0 Autonomic neuropathy in endocrine and metabolic diseases
G99.1 Other disorders of autonomic nervous system in other diseases classified elsewhere
G99.2 Myelopathy in diseases classified elsewhere
G99.8 Other specified disorders of nervous system in diseases classified elsewhere
S02.00 Fracture of vault of skull (closed)
S02.01 Fracture of vault of skull (open)
S02.10 Fracture of base of skull (closed)
S02.11 Fracture of base of skull (open)
S02.70 Multiple fractures involving skull and facial bones (closed)
S02.71 Multiple fractures involving skull and facial bones (open)
S02.80 Fractures of other skull and facial bones (closed)
S02.81 Fractures of other skull and facial bones (open)
S02.90 Fracture of skull and facial bones, part unspecified (closed)
S02.91 Fracture of skull and facial bones, part unspecified (open)
S04.0 Injury of optic nerve and pathways
S04.1 Injury of oculomotor nerve
S04.2 Injury of trochlear nerve

For peer review only

S04.3 Injury of trigeminal nerve
S04.5 Injury of facial nerve
S04.8 Injury of other cranial nerves
S04.9 Injury of unspecified cranial nerve
S06.00 Concussion (without open intracranial wound)
S06.01 Concussion (with open intracranial wound)
S06.10 Traumatic cerebral oedema (without open intracranial wound)
S06.20 Diffuse brain injury (without open intracranial wound)
S06.21 Diffuse brain injury (with open intracranial wound)
S06.30 Focal brain injury (without open intracranial wound)
S06.31 Focal brain injury (with open intracranial wound)
S06.40 Epidural haemorrhage (without open intracranial wound)
S06.41 Epidural haemorrhage (with open intracranial wound)
S06.50 Traumatic subdural haemorrhage (without open intracranial wound)
S06.51 Traumatic subdural haemorrhage (with open intracranial wound)
S06.60 Traumatic subarachnoid haemorrhage (without open intracranial wound)
S06.61 Traumatic subarachnoid haemorrhage (with open intracranial wound)
S06.70 Intracranial injury with prolonged coma (without open intracranial wound)
S06.80 Other intracranial injuries (without open intracranial wound)
S06.81 Other intracranial injuries (with open intracranial wound)
S06.90 Intracranial injury, unspecified (without open intracranial wound)
S06.91 Intracranial injury, unspecified (with open intracranial wound)
S09.7 Multiple injuries of head
S09.9 Unspecified injury of head
T90.1 Sequelae of open wound of head
T90.2 Sequelae of fracture of skull and facial bones
T90.3 Sequelae of injury of cranial nerves
T90.5 Sequelae of intracranial injury
T90.8 Sequelae of other specified injuries of head
T90.9 Sequelae of unspecified injury of head
T91.3 Sequelae of injury of spinal cord

For peer review only

Codes for the psychotropic medications used as exclusion criteria

Field ID	Code	Description
20003	1140921600	citalopram
20003	1141180212	escitalopram
20003	1141190158	cipralex 5mg tablet
20003	1141151946	cipramil 10mg tablet
20003	1140879540	fluoxetine
20003	1140867876	prozac 20mg capsule
20003	1140867878	sertraline
20003	1140879544	fluvoxamine
20003	1140867860	faverin 50mg tablet
20003	1140867888	paroxetine
20003	1140882236	seroxat 20mg tablet
20003	1140867884	lustral 50mg tablet
20003	1140916282	venlafaxine
20003	1141200564	duloxetine
20003	1141201834	cymbalta 30mg gastro-resistant capsule
20003	1140916288	efexor 37.5mg tablet
20003	1140917460	nefazodone
20003	1140917466	dutonin 100mg tablet
20003	1140879634	trazodone
20003	1141151978	reboxetine
20003	1141151982	edronax 4mg tablet
20003	1140879688	viloxazine
20003	1140867770	vivalan 50mg tablet
20003	1141199446	atomoxetine
20003	1141199460	strattera 10mg capsule
20003	1141176854	bupropion
20003	1140879616	amitriptyline
20003	1140867938	amitriptyline+chlordiazepoxide 12.5mg/5mg capsule
20003	1140867948	amitriptyline hydrochloride+perphenazine 10mg/2mg tablet
20003	1140867658	elavil 10mg tablet
20003	1140879620	clomipramine
20003	1140867690	anafranil 10mg capsule
20003	1140879624	desipramine
20003	1140909806	dosulepin
20003	1140867624	prothiaden 25mg capsule
20003	1141168396	doxepin hydrochloride 5% cream
20003	1140882312	sinequan 10mg capsule
20003	1140879630	imipramine
20003	1140867712	tofranil 10mg tablet
20003	1140867726	lofepramine
20003	1141146062	lomont 70mg/5ml s/f suspension
20003	1140882310	gamanil 70mg tablet

For peer review only

20003	1140867818	nortriptyline
20003	1140867940	fluphenazine hydrochloride+nortriptyline 1.5mg/30mg tablet
20003	1140867942	fluphenazine hcl+nortriptyline 500micrograms/10mg tablet
20003	1140867824	aventyl 10mg capsule
20003	1140879632	protriptyline
20003	1140867756	trimipramine
20003	1140867758	surmontil 10mg tablet
20003	1140867720	iprindole
20003	1140867722	prondol 15mg tablet
20003	1140867734	concordin 5mg tablet
20003	1140856074	butriptyline
20003	1140856076	evadyne 25mg tablet
20003	1140867774	amoxapine
20003	1140867840	asendis 25mg tablet
20003	1140879552	maprotiline
20003	1140867784	ludiomil 10mg tablet
20003	1140879556	mianserin
20003	1141152732	mirtazapine
20003	1140867856	isocarboxazid
20003	1140867858	marplan 10mg tablet
20003	1140910704	maoi - phenelzine
20003	140867850	phenelzine
20003	1140867852	nardil 15mg tablet
20003	1140867914	tranylcypromine
20003	1140867916	parnate 10mg tablet
20003	1140879668	selegiline
20003	1140872348	eldepryl 5mg tablet
20003	1141169666	zelapar 1.25mg tablet
20003	1140867920	moclobemide
20003	1140867922	manerix 150mg tablet
20003	1140867960	tryptophan product
20003	1140879674	pipothiazine
20003	1141153490	amisulpride
20003	1141184742	solian 100mg/ml s/f oral solution
20003	1141195974	aripiprazole
20003	1141202024	abilify 5mg tablet
20003	1140928916	olanzapine
20003	1141167976	zyprexa 2.5mg tablet
20003	1141152848	quetiapine
20003	1141152860	seroquel 25mg tablet
20003	1140867444	risperidone
20003	1141177762	risperdal 0.5mg tablet
20003	1140867078	benperidol
20003	1140867084	droperidol
20003	1140867080	anquil 250micrograms tablet
20003	1140867086	droleptan 10mg tablet

For peer review only

20003	1140867168	haloperidol
20003	1140867180	dozic 1mg/ml oral liquid
20003	1140867184	haldol 5mg tablet
20003	1140867092	serenace 500micrograms capsule
20003	1140867546	fluspirilene
20003	1140867548	redeptin 2mg/1ml injection
20003	1140867218	pimozide
20003	1140879658	chlorpromazine
20003	1140910358	cpz - chlorpromazine
20003	1140867398	fluphenazine decanoate
20003	1140867940	fluphenazine hydrochloride+nortriptyline 1.5mg/30mg tablet
20003	1140867942	fluphenazine hcl+nortriptyline 500micrograms/10mg tablet
20003	1140867944	tranylcypromine+trifluoperazine 10mg/1mg tablet
20003	1140867948	amitriptyline hydrochloride+perphenazine 10mg/2mg tablet
20003	1140909802	levomepromazine
20003	1140867122	nozinan 25mg tablet
20003	1140867134	pericyazine
20003	1140867136	neulactil 2.5mg tablet
20003	1140867208	perphenazine
20003	1140867210	fentazin 2mg tablet
20003	1140909804	pipotiazine
20003	1140867572	piportil depot 50mg/1ml oily injection
20003	1140868170	prochlorperazine
20003	1140868172	stemetil 5mg tablet
20003	1140879746	promazine
20003	1140867288	sparine 50mg/5ml suspension
20003	1140882082	promethazine product
20003	1140879750	thioridazine
20003	1140867312	melleril 10mg tablet
20003	1140867304	sulpiride
20003	1140868120	trifluoperazine
20003	1140867244	stelazine 1mg tablet
20003	1140856052	chlorprothixene
20003	1140882100	zuclopenthixol
20003	1140867150	flupenthixol
20003	1140867152	depixol 3mg tablet
20003	1140867156	moditen 1mg tablet
20003	1140867342	clopixol 2mg tablet
20003	1140867306	dolmatil 200mg tablet
20003	1141185130	sulpor 200mg/5ml oral solution
20003	1140867406	loxapine
20003	1140867414	loxapac 10mg capsule
20003	1140867420	clozapine
20003	1140879704	remoxipride
20003	1140867432	roxiam 150mg m/r capsule
20003	1140882320	clozaril 25mg tablet

For peer review only

20003	1141169714	zotepine
20003	1141169722	zoleptil 25mg tablet
20003	1140927956	sertindole
20003	1140927970	serdolect 4mg tablet
20003	1140855960	fortunan 500micrograms tablet
20003	1141200004	pregabalin
20003	1141200072	lyrica 25mg capsule
20003	1140883656	hydroxyzine
20003	1140863286	atarax 10mg tablet
20003	1140863292	ucerax 25mg tablet
20003	1140863302	lorazepam
20003	1140863308	alprazolam
20003	1140863310	xanax 250mcg tablet
20003	1140863318	bromazepam
20003	1140863320	lexotan 1.5mg tablet
20003	1140863364	ativan 1mg tablet
20003	1140863372	medazepam
20003	1140863374	nobrium 5mg capsule
20003	1140863268	clobazam
20003	1140863272	frisium 10mg capsule
20003	1140863274	potassium clorazepate
20003	1140863276	tranxene 7.5mg capsule
20003	1140863152	diazepam
20003	1140863164	rimapam 2mg tablet
20003	1140863170	diazemuls 10mg/2ml injection
20003	1140863172	dialar 2mg/5ml syrup
20003	1140863234	stesolid 5mg rectal solution
20003	1140863238	tensium 2mg tablet
20003	1140863244	valium 2mg tablet
20003	1140863250	valium 2mg/5ml syrup
20003	1140863442	oxazepam
20003	1140863454	buspar 5mg tablet
20003	1140879730	buspirone
20003	1140855944	prazepam
20003	1140855946	centrax 10mg tablet
20003	1140863328	chlordiazepoxide
20003	1140863350	librium 5mg tablet
20003	1140872150	clonazepam
20003	1140872152	rivotril 500mcg tablet
20003	1140856092	pacitron 500mg tablet
20003	1140856108	villescon tablet

For peer review only

Codes for the endoc

Field ID	Code
20002	1214
20002	1220
20002	1221
20002	1222
20002	1223
20002	1224
20002	1225
20002	1226
20002	1228
20002	1229
20002	1230
20002	1232
20002	1233
20002	1234
20002	1235
20002	1236
20002	1237
20002	1238
20002	1239
20002	1350
20002	1428
20002	1429
20002	1430
20002	1431
20002	1432
20002	1521
20002	1522
20002	1610
20002	1611
20002	1682
41270	C73
41270	C740
41270	C741
41270	C749
41270	C750
41270	C751
41270	C752
41270	C753
41270	C754
41270	C755
41270	C758
41270	C759

For peer review only

41270 E011
41270 E018
41270 E02
41270 E030
41270 E031
41270 E032
41270 E033
41270 E034
41270 E035
41270 E038
41270 E039
41270 E040
41270 E041
41270 E042
41270 E048
41270 E049
41270 E050
41270 E051
41270 E052
41270 E053
41270 E054
41270 E055
41270 E058
41270 E059
41270 E060
41270 E061
41270 E062
41270 E063
41270 E064
41270 E065
41270 E069
41270 E070
41270 E071
41270 E078
41270 E079
41270 E100
41270 E101
41270 E102
41270 E103
41270 E104
41270 E105
41270 E106
41270 E107
41270 E108
41270 E109
41270 E110

For peer review only

41270 E111
41270 E112
41270 E113
41270 E114
41270 E115
41270 E116
41270 E117
41270 E118
41270 E119
41270 E121
41270 E123
41270 E125
41270 E128
41270 E129
41270 E130
41270 E131
41270 E132
41270 E133
41270 E134
41270 E135
41270 E136
41270 E137
41270 E138
41270 E139
41270 E140
41270 E141
41270 E142
41270 E143
41270 E144
41270 E145
41270 E146
41270 E147
41270 E148
41270 E149
41270 E15
41270 E160
41270 E161
41270 E162
41270 E163
41270 E164
41270 E168
41270 E169
41270 E200
41270 E201
41270 E208
41270 E209

For peer review only

41270 E210
41270 E211
41270 E212
41270 E213
41270 E214
41270 E215
41270 E220
41270 E221
41270 E222
41270 E228
41270 E229
41270 E230
41270 E231
41270 E232
41270 E233
41270 E236
41270 E237
41270 E240
41270 E241
41270 E242
41270 E243
41270 E244
41270 E248
41270 E249
41270 E250
41270 E258
41270 E259
41270 E270
41270 E271
41270 E272
41270 E273
41270 E274
41270 E275
41270 E278
41270 E279
41270 E281
41270 E282
41270 E283
41270 E288
41270 E289
41270 E290
41270 E291
41270 E298
41270 E299
41270 E351
41270 E340

For peer review only

rine conditions used as exclusion criteria for the control group

Description
testicular problems (not cancer)
diabetes
gestational diabetes
type 1 diabetes
type 2 diabetes
thyroid problem (not cancer)
hyperthyroidism/thyrotoxicosis
hypothyroidism/myxoedema
thyroid radioablation therapy
parathyroid gland problem (not cancer)
parathyroid hyperplasia/adenoma
disorder of adrenal gland
adrenal tumour
adrenocortical insufficiency/addison's disease
hyperaldosteronism/conn's syndrome
phaeochromocytoma
disorder or pituitary gland
pituitary adenoma/tumour
cushings syndrome
polycystic ovaries/polycystic ovarian syndrome
thyroiditis
acromegaly
hypopituitarism
hyperprolactinaemia
carcinoid syndrome/tumour
diabetes insipidus
grave's disease
thyroid goitre
hyperparathyroidism
benign insulinoma
C73 Malignant neoplasm of thyroid gland
C74.0 Cortex of adrenal gland
C74.1 Medulla of adrenal gland
C74.9 Adrenal gland, unspecified
C75.0 Parathyroid gland
C75.1 Pituitary gland
C75.2 Craniopharyngeal duct
C75.3 Pineal gland
C75.4 Carotid body
C75.5 Aortic body and other paraganglia
C75.8 Pluriglandular involvement, unspecified
C75.9 Endocrine gland, unspecified

For peer review only

E01.1 Iodine-deficiency-related multinodular (endemic) goitre
E01.8 Other iodine-deficiency-related thyroid disorders and allied conditions
E02 Subclinical iodine-deficiency hypothyroidism
E03.0 Congenital hypothyroidism with diffuse goitre
E03.1 Congenital hypothyroidism without goitre
E03.2 Hypothyroidism due to medicaments and other exogenous substances
E03.3 Postinfectious hypothyroidism
E03.4 Atrophy of thyroid (acquired)
E03.5 Myxoedema coma
E03.8 Other specified hypothyroidism
E03.9 Hypothyroidism, unspecified
E04.0 Non-toxic diffuse goitre
E04.1 Non-toxic single thyroid nodule
E04.2 Non-toxic multinodular goitre
E04.8 Other specified non-toxic goitre
E04.9 Non-toxic goitre, unspecified
E05.0 Thyrotoxicosis with diffuse goitre
E05.1 Thyrotoxicosis with toxic single thyroid nodule
E05.2 Thyrotoxicosis with toxic multinodular goitre
E05.3 Thyrotoxicosis from ectopic thyroid tissue
E05.4 Thyrotoxicosis factitia
E05.5 Thyroid crisis or storm
E05.8 Other thyrotoxicosis
E05.9 Thyrotoxicosis, unspecified
E06.0 Acute thyroiditis
E06.1 Subacute thyroiditis
E06.2 Chronic thyroiditis with transient thyrotoxicosis
E06.3 Autoimmune thyroiditis
E06.4 Drug-induced thyroiditis
E06.5 Other chronic thyroiditis
E06.9 Thyroiditis, unspecified
E07.0 Hypersecretion of calcitonin
E07.1 Dyshormogenetic goitre
E07.8 Other specified disorders of thyroid
E07.9 Disorder of thyroid, unspecified
E10.0 With coma
E10.1 With ketoacidosis
E10.2 With renal complications
E10.3 With ophthalmic complications
E10.4 With neurological complications
E10.5 With peripheral circulatory complications
E10.6 With other specified complications
E10.7 With multiple complications
E10.8 With unspecified complications
E10.9 Without complications
E11.0 With coma

For peer review only

E11.1 With ketoacidosis
E11.2 With renal complications
E11.3 With ophthalmic complications
E11.4 With neurological complications
E11.5 With peripheral circulatory complications
E11.6 With other specified complications
E11.7 With multiple complications
E11.8 With unspecified complications
E11.9 Without complications
E12.1 With ketoacidosis
E12.3 With ophthalmic complications
E12.5 With peripheral circulatory complications
E12.8 With unspecified complications
E12.9 Without complications
E13.0 With coma
E13.1 With ketoacidosis
E13.2 With renal complications
E13.3 With ophthalmic complications
E13.4 With neurological complications
E13.5 With peripheral circulatory complications
E13.6 With other specified complications
E13.7 With multiple complications
E13.8 With unspecified complications
E13.9 Without complications
E14.0 With coma
E14.1 With ketoacidosis
E14.2 Withrenal complications
E14.3 With ophthalmic complications
E14.4 With neurological complications
E14.5 With peripheral circulatory complications
E14.6 With other specified complications
E14.7 With multiple complications
E14.8 With unspecified complications
E14.9 Without complications
E15 Nondiabetic hypoglycaemic coma
E16.0 Drug-induced hypoglycaemia without coma
E16.1 Other hypoglycaemia
E16.2 Hypoglycaemia, unspecified
E16.3 Increased secretion of glucagon
E16.4 Abnormal secretion of gastrin
E16.8 Other specified disorders of pancreatic internal secretion
E16.9 Disorder of pancreatic internal secretion, unspecified
E20.0 Idiopathic hypoparathyroidism
E20.1 Pseudohypoparathyroidism
E20.8 Other hypoparathyroidism
E20.9 Hypoparathyroidism, unspecified

For peer review only

E21.0 Primary hyperparathyroidism
E21.1 Secondary hyperparathyroidism, not elsewhere classified
E21.2 Other hyperparathyroidism
E21.3 Hyperparathyroidism, unspecified
E21.4 Other specified disorders of parathyroid gland
E21.5 Disorder of parathyroid gland, unspecified
E22.0 Acromegaly and pituitary gigantism
E22.1 Hyperprolactinaemia
E22.2 Syndrome of inappropriate secretion of antidiuretic hormone
E22.8 Other hyperfunction of pituitary gland
E22.9 Hyperfunction of pituitary gland, unspecified
E23.0 Hypopituitarism
E23.1 Drug-induced hypopituitarism
E23.2 Diabetes insipidus
E23.3 Hypothalamic dysfunction, not elsewhere classified
E23.6 Other disorders of pituitary gland
E23.7 Disorder of pituitary gland, unspecified
E24.0 Pituitary-dependent Cushing's disease
E24.1 Nelson's syndrome
E24.2 Drug-induced Cushing's syndrome
E24.3 Ectopic ACTH syndrome
E24.4 Alcohol-induced pseudo-Cushing's syndrome
E24.8 Other Cushing's syndrome
E24.9 Cushing's syndrome, unspecified
E25.0 Congenital adrenogenital disorders associated with enzyme deficiency
E25.8 Other adrenogenital disorders
E25.9 Adrenogenital disorder, unspecified
E27.0 Other adrenocortical overactivity
E27.1 Primary adrenocortical insufficiency
E27.2 Addisonian crisis
E27.3 Drug-induced adrenocortical insufficiency
E27.4 Other and unspecified adrenocortical insufficiency
E27.5 Adrenomedullary hyperfunction
E27.8 Other specified disorders of adrenal gland
E27.9 Disorder of adrenal gland, unspecified
E28.1 Androgen excess
E28.2 Polycystic ovarian syndrome
E28.3 Primary ovarian failure
E28.8 Other ovarian dysfunction
E28.9 Ovarian dysfunction, unspecified
E29.0 Testicular hyperfunction
E29.1 Testicular hypofunction
E29.8 Other testicular dysfunction
E29.9 Testicular dysfunction, unspecified
E35.1 Disorders of adrenal glands in diseases classified elsewhere
E34.0 Carcinoid syndrome

For peer review only

Field ID	Description
20023	Mean time to correctly identify matches (Reaction time)
20016	Fluid intelligence score (Fluid intelligence)
6348	Duration to complete numeric path (trail #1) (Trail making A)
6350	Duration to complete alphanumeric path (trail #2) (Trail making B)
23324	Number of symbol digit matches made correctly (Symbol digit substitution)
4282	Maximum digits remembered correctly (Digit span)

For peer review only

Field ID	Description
2050	Frequency of depressed mood in last 2 weeks
2060	Frequency of unenthusiasm / disinterest in last 2 weeks
2070	Frequency of tenseness / restlessness in last 2 weeks
2080	Frequency of tiredness / lethargy in last 2 weeks

For peer review only

Field ID	Description
31	Sex
21003	Age when attended assessment center
6138	Qualifications (education)
20116	Smoking status
53	Year of imaging
54	Assessment center
25756	Scanner lateral (X) brain position
25757	Scanner transverse (Y) brain position
25758	Scanner longitudinal (Z) brain position
25759	Scanner table position
25000	Volumetric scaling from T1 head image to standard space (measure of head size)
23104	Body mass index measured by body impedance
23099	Total body fat percentage measured by body impedance

For peer review only

Supplement 2. Structural or diffusion MRI studies in patients with Cushing disease or exogenous glucocorticoid use

	Cohort	Imaging modality	Findings
**Cushing**			
Andela 2013 [1]	25 CD patients with long-term remission, 25 healthy controls	Volumetric MRI	Patients had reduced GMV in ACC and increased GMV in left posterior lobe of cerebellum. Patients reported more psychological and cognitive symptoms than controls, but these were not associated with GMV changes.
Bauduin 2020 [2]	25 CD patients with long-term remission, 25 healthy controls	Volumetric MRI	Patients had reduced cortical thickness in left caudal ACC, right rostral ACC, left cuneus, left PCC, and bilateral precuneus. Cortical thickness in left caudal ACC and left cuneus were inversely associated with anxiety, depressive symptoms, and disease duration.
Bourdeau 2002 [3]	38 patients with active CS (21 with CD 17 with adrenal CS), 18 patients with other non-ACTH-secreting sellar tumors, 20 normal controls	Volumetric MRI	Overall loss of brain volume and increased ventricle diameters in CS patients. Re-imaging in 22 CS patients at 40 months after correction of hypercortisolism showed a decrease in ventricle diameters and increase in brain volume compared to active disease.
Burkhardt 2015 [4]	19 patients with active untreated CD, 40 healthy controls	Volumetric MRI	CD patients had reduced GMV in hippocampus and cerebellum compared to controls.
Chen 2020 [5]	101 patients with active untreated CD, and 95 patients with NFA (controls)	Volumetric MRI	CD patients had more cortical and subcortical atrophy, more white matter hyperintensities, and decreased hippocampal height. Follow-up of 14 CD patients showed partial reversion of brain atrophy and white matter hyperintensities after correction of hypercortisolism.
Crespo 2014 [6]	35 patients with CS (27 cured, 8 medically treated), 35 controls	Volumetric MRI	No differences were found between cured and treated CS patients. Patients had decreased cortical thickness in the left superior frontal cortex, precentral cortex, left insular cortex, left and right rostral ACC, and right caudal middle frontal cortex compared to controls. Patients also had altered decision-making strategies.
Hou [7]	50 patients with active CD, 36 healthy controls	Volumetric MRI	Patients had reductions in total GMV and frontal, parietal, occipital, and temporal lobes; insula; cingulate lobe; and enlargement of lateral and third ventricles. All affected brain regions improved significantly after TSS. No differences in volume of hippocampus or amygdala.
Jiang 2017 [8]	34 patients with CD (14 with short-term remission, 20 with active CD), 34 controls	Volumetric MRI	Remitted CD patients had greater GMV in bilateral caudate; no differences in GMV of MFG or cerebellum compared to controls. Active CD patients had smaller GMV in MFG and cerebellum compared to controls and remitted patients.
Jiang 2017 [9]	15 patients with active CD, 15 healthy controls	DKI	White matter: increased MD in the splenium of the corpus callosum, bilateral frontal lobe, and left temporal lobe. AD was mainly increased in the bilateral

For peer review only

			frontal lobe, and RD mainly in the left temporal lobe. FA was mainly decreased in the splenium of the corpus callosum and the left temporal lobe.<br>Gray matter: increased MD, RD, and AD in the left hippocampus/parahippocampal gyrus and the left temporal lobe, increased radial kurtosis in the right cerebellar hemisphere, decreased axial kurtosis in the left frontal lobe and decreased mean kurtosis in left cerebellar hemisphere.
Merke 2005 [10]	11 pediatric patients with active CS, 10 healthy controls	Volumetric MRI	CD patients had smaller cerebral volumes, larger ventricles, and a smaller amygdala. One year after surgical cure, cerebral atrophy was reversed, but children showed a decline in IQ and school performance.
Momose 1971 [11]	31 patients with active CD, 64 patients with acromegaly, 36 patients with chromophobe adenoma	Pneumoencephalography	Cerebral cortical atrophy was present in 90% and cerebellar cortical atrophy in 74% of patients with CD. In controls with acromegaly, this was 30% and 3%, respectively. In controls with a chromophobe adenoma, this was 58% and 20%, respectively.
Pires 2015 [12]	35 patients with CS (8 active hypercortisolism, 7 medication-remitted cortisol, 20 surgically cured), 35 healthy controls	DTI	Patients had widespread alteration in white matter integrity (increased FA, decreased MD, RD, AD) compared to controls. Both active and cured CS patients showed increased FA, and decreased MD, RD, and AD; medically treated CS patients did not have significantly different AD values.
Pires 2017 [13]	35 patients with CS (8 active hypercortisolism, 7 medication-remitted cortisol, 20 surgically cured), 35 healthy controls	DTI	All patient groups had more depression and anxiety than controls. Depression scores correlated negatively to FA (in right corticospinal tract (CST), forceps major, forceps minor, left inferior fronto-occipital fasciculus (IFOF) (frontal part), right IFOF, right inferior longitudinal fasciculus (ILF), left superior longitudinal fasciculus (SLF) (anterior part) and right SLF), and positively to RD values (in frontal regions of the forceps minor and frontal areas of bilateral IFOFs). Although processing speed did not differ between groups, Symbol Digit Modalities Test scores correlated positively to both FA and AD values.
Resmini 2012 [14]	33 patients with CS (11 active, 22 cured), 34 controls	Volumetric MRI	Patients had reduced total and cortical brain gray matter volumes compared with controls. Subcortical gray matter (which includes hippocampal volume) was reduced only in 12 patients with severe memory impairment. No differences in hippocampal volume were reported between patients with active or cured CS.
Santos 2014 [15]	36 patients with CS (15 active, 21 in remission), 36 controls	Volumetric MRI	Patients with active CS had smaller cerebellar cortex volumes, and patients with remitted CS showed a similar trend. Cerebellar white matter volume showed no differences.
Santos 2015 [16]	38 patients with CS (15 active, 23 in remission), 38 controls	Volumetric MRI	Patients in remission had more white matter lesions than controls and active patients. Both CS groups had reduced total brain volume and GMV. No differences were found in white matter volume.

For peer review only

Santos 2017 [17]	39 patients with CS (16 active, 23 in remission), 39 healthy controls	Volumetric MRI	Active CS patients had smaller right amygdala volumes. Left amygdala volume was associated with depression and anxiety scores. No differences were found between patients in remission and controls.
Simmons 2000 [18]	63 patients with CD (all after surgical treatment), 63 controls with sellar pathology other than ACTH-secreting tumors	Volumetric MRI	CD patients had higher degrees of cerebral atrophy than controls.
Starkman 1992 [19]	12 patients with CS	Volumetric MRI	For 27% of patients, hippocampal volume fell outside the 95% confidence interval of the population. Plasma cortisol was negatively correlated with hippocampal volume.
Starkman 1999 [20]	22 patients with active CD	Volumetric MRI	Sixteen months after TSS, hippocampus volume increased up to 10%, and a smaller increase was observed in caudate volume.
Starkman 2003 [21]	24 patients with active CD	Volumetric MRI	Sixteen months after TSS, all patients showed an increase in hippocampal volume (which was significantly correlated with lower cortisol levels, and with one neuropsychological test), and 18 patients had an increase in caudate head volume.
Tirosh 2020 [22]	29 patients with CS (8 active, 21 recovering), 8 controls	Volumetric MRI	Patients with persistent disease had increased white matter volume and decreased cortex thickness and white matter intensity compared with patients achieving remission of CS, mainly in frontal and parietal lobes (but not FDR-corrected). Compared to healthy controls, patients recovering from CS had a decrease in subcortical GM volume, an increase in cortical thickness, and a decrease in white matter volume in multiple sites (including accumbens).<br>In all patients together, 24h UFC correlated negatively with intensity in caudate, hippocampus, accumbens, and corpus callosum; correlated negatively with white matter intensity in frontal and parietal lobes; and positively with lateral ventricles volumes. Changes in 24h UFC correlated negatively with change in total brain volume, supratentorium, cerebellar cortex, and putamen.
Toffanin 2011 [23]	10 patients with active CD	Volumetric MRI	After TSS, the volume of the hippocampal head increased significantly, but no change in hippocampal body or tail, nor in whole brain volume was observed.
Van der Werff 2014 [24]	22 patients with long-term remission of CD, 22 healthy controls	DTI	Patients had widespread FA reductions in whole brain analysis. ROI analysis revealed reduced FA in the bilateral cingulate cingulum, bilateral uncinate fasciculus and corpus callosum. No significant differences were found in tracts in the inferior parts of the brainstem, the white matter in the bilateral cerebellum, the bilateral hippocampal cingulum, the left inferior fronto-occipital fasciculus, and parts of the bilateral superior longitudinal fasciculus. Patients also had increased radial and mean diffusivity, but no difference in axial diffusivity.

For peer review only

Exogenous GC			
Bentson 1978 [25]	15 long-term GC users	CT	Patients showed varying degrees of apparent cerebral atrophy. Some correlation between dosage and degree of atrophy appeared to be present.
Brown 2004 [26]	17 chronic (>6 months) GC (prednisone) users, 15 controls	Volumetric MRI, PMRS	GC users had smaller hippocampal volume, lower N-acetyl aspartate ratios, more mood symptoms and poorer cognitive function.
Brown 2015 [27]	17 healthy adults who received hydrocortisone (160 mg/day)/placebo, phenytoin/placebo, hydrocortisone/phenytoin, or placebo/placebo, in a randomized, blinded, cross-over trial with 21-day washout between conditions.	Volumetric MRI	Hydrocortisone use was not associated with difference in total brain volume but was associated with a 1.69% reduction in total hippocampal volume compared to placebo. Phenytoin blocked this hippocampal volume reduction by hydrocortisone.
Brown 2019 [28]	46 chronic GC users, randomized to memantine or placebo in blinded, cross-over trial (two 24-week treatment periods, separated by four-week washout)	Volumetric MRI	Hippocampal volume decreased significantly from baseline to week 52 and from week 24 to week 52, without significant difference between baseline and week 24. Following 24 weeks of memantine, left dentate gyrus/CA3 volume was significantly larger than after placebo; a similar trend was observed in the right CA1. Subiculum showed no significant differences.
Brown 2008 [29]	15 chronic (>6 months) GC (prednisone) users, 13 controls	Volumetric MRI	GC users had significantly smaller amygdala volumes compared to controls. Duration of GC therapy correlated negatively with right amygdala volume.
Desai 2009 [30]	28 chronic (>6 months) GC (prednisone) users, randomized to 24 weeks of lamotrigine (n = 16) or placebo (n = 12) in blinded trial	Volumetric MRI	After 24 weeks, amygdala volume was reduced in both groups, but right amygdala volume was significantly less reduced in the lamotrigine group than in the placebo group.
Nguyn 2019 [31]	81 chronic (>6 months) GC (prednisone) users	Volumetric MRI	Cumulative GC exposure negatively associated with the volumes of the left and right hippocampal dentate gyrus/CA3; no associations were found for entorhinal, perirhinal, or parahippocampal gyri, subiculum, or CA1.

AD, axial diffusivity, ACC, anterior cingulate cortex; CD, Cushing disease; CS, Cushing syndrome; CT, computed tomography; FA, fractional anisotropy; DKI, diffusional kurtosis imaging; DMN, default mode network; GC, glucocorticoids; GMV, grey matter volume; MD, mean diffusivity; MFG, medial frontal gyrus; NFA, non-functioning pituitary adenoma; PCC, posterior cingulate cortex; PMRS, proton magnetic resonance spectroscopy; RD, radial diffusivity; RSFC, resting-state functional connectivity; TSS, transsphenoidal surgery; 24h UFC, 24-hour urinary free cortisol.

For peer review only

References

1. Andela, C.D., van der Werff, S.J., Pannekoek, J.N., van den Berg, S.M., Meijer, O.C., van Buchem, M.A., Rombouts, S.A., van der Mast, R.C., Romijn, J.A., Tiemensma, J., Biermasz, N.R., van der Wee, N.J., Pereira, A.M.: Smaller grey matter volumes in the anterior cingulate cortex and greater cerebellar volumes in patients with long-term remission of Cushing's disease: a case-control study. Eur J Endocrinol **169**(6), 811-819 (2013). doi:10.1530/eje-13-0471
2. Bauduin, S., van der Pal, Z., Pereira, A.M., Meijer, O.C., Giltay, E.J., van der Wee, N.J.A., van der Werff, S.J.A.: Cortical thickness abnormalities in long-term remitted Cushing's disease. Transl Psychiatry **10**(1), 293 (2020). doi:10.1038/s41398-020-00980-6
3. Bourdeau, I., Bard, C., Noël, B., Leclerc, I., Cordeau, M.P., Bélair, M., Lesage, J., Lafontaine, L., Lacroix, A.: Loss of brain volume in endogenous Cushing's syndrome and its reversibility after correction of hypercortisolism. J Clin Endocrinol Metab **87**(5), 1949-1954 (2002). doi:10.1210/jcem.87.5.8493
4. Burkhardt, T., Lüdecke, D., Spies, L., Wittmann, L., Westphal, M., Flitsch, J.: Hippocampal and cerebellar atrophy in patients with Cushing's disease. Neurosurg Focus **39**(5), E5 (2015). doi:10.3171/2015.8.Focus15324
5. Chen, Y., Zhang, J., Tan, H., Li, J., Yu, Y.: Detrimental effects of hypercortisolism on brain structure and related risk factors. Sci Rep **10**(1), 12708 (2020). doi:10.1038/s41598-020-68166-0
6. Crespo, I., Esther, G.M., Santos, A., Valassi, E., Yolanda, V.G., De Juan-Delago, M., Webb, S.M., Gómez-Ansón, B., Resmini, E.: Impaired decision-making and selective cortical frontal thinning in Cushing's syndrome. Clinical endocrinology **81**(6), 826-833 (2014).
7. Hou, B., Gao, L., Shi, L., Luo, Y., Guo, X., Young, G.S., Qin, L., Zhu, H., Lu, L., Wang, Z., Feng, M., Bao, X., Wang, R., Xing, B., Feng, F.: Reversibility of impaired brain structures after transsphenoidal surgery in Cushing's disease: a longitudinal study based on an artificial intelligence-assisted tool. J Neurosurg, 1-10 (2020). doi:10.3171/2019.10.Jns191400
8. Jiang, H., Ren, J., He, N.Y., Liu, C., Sun, Y.H., Jian, F.F., Bian, L.G., Shen, J.K., Yan, F.H., Pan, S.J., Sun, Q.F.: Volumetric magnetic resonance imaging analysis in patients with short-term remission of Cushing's disease. Clin Endocrinol (Oxf) **87**(4), 367-374 (2017). doi:10.1111/cen.13381
9. Jiang, H., He, N.Y., Sun, Y.H., Jian, F.F., Bian, L.G., Shen, J.K., Yan, F.H., Pan, S.J., Sun, Q.F.: Altered gray and white matter microstructure in Cushing's disease: A diffusional kurtosis imaging study. Brain Res **1665**, 80-87 (2017). doi:10.1016/j.brainres.2017.04.007
10. Merke, D.P., Giedd, J.N., Keil, M.F., Mehlinger, S.L., Wiggs, E.A., Holzer, S., Rawson, E., Vaituzis, A.C., Stratakis, C.A., Chrousos, G.P.: Children experience cognitive decline despite reversal of brain atrophy one year after resolution of Cushing syndrome. J Clin Endocrinol Metab **90**(5), 2531-2536 (2005). doi:10.1210/jc.2004-2488
11. Momose, K.J., Kjellberg, R.N., Kliman, B.: High incidence of cortical atrophy of the cerebral and cerebellar hemispheres in Cushing's disease. Radiology **99**(2), 341-348 (1971). doi:10.1148/99.2.341
12. Pires, P., Santos, A., Vives-Gilabert, Y., Webb, S.M., Sainz-Ruiz, A., Resmini, E., Crespo, I., de Juan-Delago, M., Gómez-Anson, B.: White matter alterations in the brains of patients with active, remitted, and cured cushing syndrome: a DTI study. AJNR Am J Neuroradiol **36**(6), 1043-1048 (2015). doi:10.3174/ajnr.A4322

For peer review only

13. Pires, P., Santos, A., Vives-Gilabert, Y., Webb, S.M., Sainz-Ruiz, A., Resmini, E., Crespo, I., de Juan-Delago, M., Gómez-Anson, B.: White matter involvement on DTI-MRI in Cushing's syndrome relates to mood disturbances and processing speed: a case-control study. Pituitary **20**(3), 340-348 (2017). doi:10.1007/s11102-017-0793-y
14. Resmini, E., Santos, A., Gómez-Anson, B., Vives, Y., Pires, P., Crespo, I., Portella, M.J., de Juan-Delago, M., Barahona, M.J., Webb, S.M.: Verbal and visual memory performance and hippocampal volumes, measured by 3-Tesla magnetic resonance imaging, in patients with Cushing's syndrome. J Clin Endocrinol Metab **97**(2), 663-671 (2012). doi:10.1210/jc.2011-2231
15. Santos, A., Resmini, E., Crespo, I., Pires, P., Vives-Gilabert, Y., Granell, E., Valassi, E., Gómez-Anson, B., Martínez-Momblán, M.A., Mataró, M., Webb, S.M.: Small cerebellar cortex volume in patients with active Cushing's syndrome. Eur J Endocrinol **171**(4), 461-469 (2014). doi:10.1530/eje-14-0371
16. Santos, A., Resmini, E., Gómez-Ansón, B., Crespo, I., Granell, E., Valassi, E., Pires, P., Vives-Gilabert, Y., Martínez-Momblán, M.A., de Juan, M., Mataró, M., Webb, S.M.: Cardiovascular risk and white matter lesions after endocrine control of Cushing's syndrome. Eur J Endocrinol **173**(6), 765-775 (2015). doi:10.1530/eje-15-0600
17. Santos, A., Granell, E., Gómez-Ansón, B., Crespo, I., Pires, P., Vives-Gilabert, Y., Valassi, E., Webb, S.M., Resmini, E.: Depression and Anxiety Scores Are Associated with Amygdala Volume in Cushing's Syndrome: Preliminary Study. Biomed Res Int **2017**, 2061935 (2017). doi:10.1155/2017/2061935
18. Simmons, N.E., Do, H.M., Lipper, M.H., Laws, E.R., Jr.: Cerebral atrophy in Cushing's disease. Surg Neurol **53**(1), 72-76 (2000). doi:10.1016/s0090-3019(99)00197-4
19. Starkman, M.N., Gebarski, S.S., Berent, S., Schteingart, D.E.: Hippocampal formation volume, memory dysfunction, and cortisol levels in patients with Cushing's syndrome. Biological psychiatry **32**(9), 756-765 (1992).
20. Starkman, M.N., Giordani, B., Gebarski, S.S., Berent, S., Schork, M.A., Schteingart, D.E.: Decrease in cortisol reverses human hippocampal atrophy following treatment of Cushing's disease. Biol Psychiatry **46**(12), 1595-1602 (1999). doi:10.1016/s0006-3223(99)00203-6
21. Starkman, M.N., Giordani, B., Gebarski, S.S., Schteingart, D.E.: Improvement in learning associated with increase in hippocampal formation volume. Biol Psychiatry **53**(3), 233-238 (2003). doi:10.1016/s0006-3223(02)01750-x
22. Tirosh, A., RaviPrakash, H., Papadakis, G.Z., Tatsi, C., Belyavskaya, E., Charalampos, L., Lodish, M.B., Bagci, U., Stratakis, C.A.: Computerized Analysis of Brain MRI Parameter Dynamics in Young Patients With Cushing Syndrome-A Case-Control Study. J Clin Endocrinol Metab **105**(5), e2069-2077 (2020). doi:10.1210/clinem/dgz303
23. Toffanin, T., Nifosì, F., Follador, H., Passamani, A., Zonta, F., Ferri, G., Scanarini, M., Amistà, P., Pigato, G., Scaroni, C., Mantero, F., Carollo, C., Perini, G.I.: Volumetric MRI analysis of hippocampal subregions in Cushing's disease: a model for glucocorticoid neural modulation. Eur Psychiatry **26**(1), 64-67 (2011). doi:10.1016/j.eurpsy.2010.09.003
24. van der Werff, S.J., Andela, C.D., Nienke Pannekoek, J., Meijer, O.C., van Buchem, M.A., Rombouts, S.A., van der Mast, R.C., Biermasz, N.R., Pereira, A.M., van der Wee, N.J.: Widespread reductions of white matter integrity in patients with long-term remission of Cushing's disease. Neuroimage Clin **4**, 659-667 (2014). doi:10.1016/j.nicl.2014.01.017
25. Bentson, J., Reza, M., Winter, J., Wilson, G.: Steroids and apparent cerebral atrophy on computed tomography scans. J Comput Assist Tomogr **2**(1), 16-23 (1978). doi:10.1097/00004728-197801000-00003

For peer review only

26. Brown, E.S., D, J.W., Frol, A., Bobadilla, L., Khan, D.A., Hanczyc, M., Rush, A.J., Fleckenstein, J., Babcock, E., Cullum, C.M.: Hippocampal volume, spectroscopy, cognition, and mood in patients receiving corticosteroid therapy. Biol Psychiatry **55**(5), 538-545 (2004). doi:10.1016/j.biopsych.2003.09.010
27. Brown, E.S., Jeon-Slaughter, H., Lu, H., Jamadar, R., Issac, S., Shad, M., Denniston, D., Tamminga, C., Nakamura, A., Thomas, B.P.: Hippocampal volume in healthy controls given 3-day stress doses of hydrocortisone. Neuropsychopharmacology **40**(5), 1216-1221 (2015). doi:10.1038/npp.2014.307
28. Brown, E.S., Kulikova, A., Van Enkevort, E., Nakamura, A., Ivleva, E.I., Tustison, N.J., Roberts, J., Yassa, M.A., Choi, C., Frol, A., Khan, D.A., Vazquez, M., Holmes, T., Malone, K.: A randomized trial of an NMDA receptor antagonist for reversing corticosteroid effects on the human hippocampus. Neuropsychopharmacology **44**(13), 2263-2267 (2019). doi:10.1038/s41386-019-0430-8
29. Brown, E.S., Woolston, D.J., Frol, A.B.: Amygdala volume in patients receiving chronic corticosteroid therapy. Biol Psychiatry **63**(7), 705-709 (2008). doi:10.1016/j.biopsych.2007.09.014
30. Desai, S., Khanani, S., Shad, M.U., Brown, E.S.: Attenuation of amygdala atrophy with lamotrigine in patients receiving corticosteroid therapy. J Clin Psychopharmacol **29**(3), 284-287 (2009). doi:10.1097/JCP.0b013e3181a3e2a7
31. Nguyen, D.M., Yassa, M.A., Tustison, N.J., Roberts, J.M., Kulikova, A., Nakamura, A., Ivleva, E.I., Van Enkevort, E., Brown, E.S.: The Relationship Between Cumulative Exogenous Corticosteroid Exposure and Volumes of Hippocampal Subfields and Surrounding Structures. J Clin Psychopharmacol **39**(6), 653-657 (2019). doi:10.1097/jcp.0000000000001120

For peer review only

Abbreviation	Meaning
GC	Glucocorticoids
FDR	False discovery rate
Lower CI	Lower end of the 95% confidence interval
Upper	Higher end of the 95% confidence interval

For peer review only

Post-hoc tests of demographic variables

		ANOVA of continuous demo	
		**F value**	**P value**
**Main population**	**Age**	13.0	2.4E-06
	**BMI**	5.8	2.9E-03
	**Body fat percentage**	15.3	2.2E-07
**Chronic users**	**Body fat percentage**	7.7	4.6E-04
**Population without exclusion criteria**	**Age**	21.8	3.6E-10
	**BMI**	8.4	2.2E-04
	**Body fat percentage**	14.4	5.5E-07

For peer review only

ographic variables	Post-hoc Dunnett's test			
	Systemic GC vs. controls			
	Estimate	Lower CI	Upper CI	P value
	2.6	1.4	3.7	**<.0001**
	0.03	-0.6	0.7	0.98
	0.7	-0.5	1.9	0.36
	-0.2	-3.0	2.6	0.97
	2.4	1.5	3.4	**<.0001**
	0.2	-0.4	0.7	0.67
	0.8	-0.2	1.8	0.16

For peer review only

Inhaled GC vs. controls

Estimate	Lower CI	Upper CI	P value
-0.2	-0.9	0.5	0.81
0.6	0.2	1.0	**1.3E-03**
1.9	1.1	2.7	**<.0001**
1.8	0.8	2.9	**2.0E-04**
-0.9	-1.5	-0.3	**2.0E-03**
0.6	0.3	1.0	**1.0E-04**
1.5	0.9	2.2	**<.0001**

For peer review only

Primary comparison: systemic GC / inhaled GC vs. controls

ANOVA of imaging parameters

	F value	P value	P_FDR
**Volumetric measures**			
Global volumes			
Total brain volume	19.7	2.8E-09	**2.0E-08**
Grey matter volume	23.7	5.4E-11	**6.5E-10**
White matter volume	6.7	1.2E-03	**2.0E-03**
Peripheral cortex	21.1	6.9E-10	**6.2E-09**
CSF volume	10.1	4.2E-05	**9.5E-05**
Subcortical volumes			
Accumbens	12.0	6.0E-06	**1.7E-05**
Amygdala	3.1	4.4E-02	5.4E-02
Caudate	6.7	1.3E-03	**2.0E-03**
Hippocampus	2.5	8.0E-02	8.7E-02
Pallidum	7.7	4.5E-04	**7.8E-04**
Putamen	10.9	1.8E-05	**4.6E-05**
Thalamus	8.2	2.7E-04	**4.9E-04**
Regional grey matter volumes			
Amygdala	23.8	5.0E-11	**6.5E-10**
Caudate	13.0	2.3E-06	**7.5E-06**
Cerebellum	10.8	2.0E-05	**4.8E-05**
Cingulate gyrus, anterior	2.6	7.4E-02	8.3E-02
Cingulate gyrus, posterior	1.2	3.1E-01	3.2E-01
Cuneal cortex	2.4	9.1E-02	9.6E-02
Hippocampus	2.7	6.8E-02	7.9E-02
Insular cortex	8.5	2.0E-04	**3.9E-04**
Medial frontal gyrus	0.5	6.1E-01	6.1E-01
Precuneal cortex	5.5	4.3E-03	**5.6E-03**
**DTI measures**			
Fractional anisotropy			
Global	19.2	4.6E-09	**2.8E-08**
Body of corpus callosum	10.0	4.7E-05	**1.0E-04**
Genu of corpus callosum	16.8	5.3E-08	**2.1E-07**
Splenium of corpus callosum	5.4	4.4E-03	**5.6E-03**
Cingulum cingulate	6.1	2.4E-03	**3.4E-03**
Cingulum hippocampus	6.4	1.7E-03	**2.5E-03**
Uncinate	2.8	6.1E-02	7.3E-02
Mean diffusivity			
Global	25.9	5.8E-12	**2.1E-10**
Body of corpus callosum	15.5	2.0E-07	**7.0E-07**
Genu of corpus callosum	18.0	1.6E-08	**7.0E-08**
Splenium of corpus callosum	9.7	6.2E-05	**1.2E-04**
Cingulum cingulate	5.4	4.3E-03	**5.6E-03**

For peer review only

Cingulum hippocampus	18.5	9.1E-09	**4.7E-08**
Uncinate	12.1	5.4E-06	**1.6E-05**

For peer review only

Post-hoc Dunnett's test

Systemic GC vs. controls				Inhaled GC vs
Estimate	Lower CI	Upper CI	P value	Estimate
-3688	-10627	3252	0.39	3374
-1968	-5904	1968	0.44	1012
-1720	-6273	2833	0.61	2362
-3303	-6843	237	0.072	1033
1215	-824	3254	0.32	78
-13.1	-26.7	0.5	0.062	-6.5
77.8	24.5	131.1	**2.3E-03**	-2.7
0.8	-29.9	31.4	1.00	-18.0
-31.3	-98.2	35.6	0.48	-27.9
3.6	-74.0	81.1	0.99	-6.4
-4.0	-31.9	23.8	0.91	-23.9
178.7	82.2	275.0	**1.0E-04**	41.2
25.1	-18.4	68.5	0.34	-12.2
-36.2	-108.4	36.0	0.43	5.0
-21.5	-179.0	136.3	0.92	-7.4
-0.0037	-0.0064	-0.0010	**4.2E-03**	-0.0023
-0.0043	-0.0084	-1.2E-04	**0.043**	-0.0023
-0.0064	-0.011	-0.0017	**5.0E-03**	-0.0019
-0.0021	-0.0053	0.0012	0.27	-0.0032
-0.0017	-0.0062	0.0028	0.61	-0.0028
6.5E-05	-0.0046	0.0048	1.00	-0.0034
7.2E-06	3.2E-06	1.1E-05	**1.0E-04**	2.7E-06
6.9E-06	1.7E-06	1.2E-05	**6.0E-03**	4.8E-06
8.4E-06	2.2E-06	1.5E-05	**4.9E-03**	4.1E-06
4.4E-06	-3.8E-08	8.9E-06	5.2E-02	5.3E-06
2.9E-06	-8.5E-07	6.6E-06	1.6E-01	2.8E-06

For peer review only

5.0E-06	4.2E-07	9.5E-06	**2.9E-02**		5.6E-06
6.4E-06	2.2E-06	1.1E-05	**1.4E-03**		2.2E-06

For peer review only

. controls

Lower CI	Upper CI	P value
-1012	7760	0.16
-1476	3500	0.57
-516	5240	0.13
-1205	3270	0.49
-1211	1367	0.98
-15.1	2.1	0.17
-36.4	30.9	0.97
-37.3	1.42	0.074
-70.2	14.4	0.25
-55.4	42.6	0.93
-41.5	-6.2	
-19.8	102.0	
-39.7	15.3	
-40.6	50.7	
-107.0	92.4	
-0.0040	-5.7E-04	**5.7E-03**
-0.0049	3.0E-04	0.092
-0.0049	0.0011	0.27
-0.0052	-0.0012	**1.0E-03**
-0.0057	8.9E-06	0.051
-0.0063	-3.8E-04	**0.024**
1.7E-07	5.2E-06	**3.4E-02**
1.6E-06	8.1E-06	**2.0E-03**
1.7E-07	8.0E-06	**3.9E-02**
2.4E-06	8.1E-06	**1.0E-04**
4.7E-07	5.2E-06	**1.5E-02**

For peer review only

2.8E-06	8.5E-06	**<.0001**
-4.4E-07	4.9E-06	1.2E-01

For peer review only

Subanalysis: chronic systemic GC / chronic inhaled GC vs. controls

ANOVA of imaging parameters

	F value	P value	P_FDR
**Volumetric measures**			
Global volumes			
Total brain volume	17.0	4.1E-08	**1.5E-06**
Grey matter volume	12.2	5.0E-06	**9.1E-05**
White matter volume	10.8	2.0E-05	**1.8E-04**
Peripheral cortex	8.5	2.1E-04	**9.4E-04**
CSF volume	3.0	5.2E-02	**7.4E-02**
Subcortical volumes			
Accumbens	0.4	7.0E-01	7.1E-01
Amygdala	5.8	2.9E-03	**8.2E-03**
Caudate	7.2	7.6E-04	**2.9E-03**
Hippocampus	4.9	7.8E-03	**1.7E-02**
Pallidum	7.1	7.9E-04	**2.9E-03**
Putamen	5.0	6.9E-03	**1.6E-02**
Thalamus	6.7	1.3E-03	**4.1E-03**
Regional grey matter volumes			
Amygdala	10.1	4.2E-05	**3.0E-04**
Caudate	1.5	2.2E-01	2.4E-01
Cerebellum	4.1	1.6E-02	**2.9E-02**
Cingulate gyrus, anterior	0.2	8.3E-01	8.3E-01
Cingulate gyrus, posterior	4.2	1.6E-02	**2.9E-02**
Cuneal cortex	2.9	5.4E-02	7.4E-02
Hippocampus	9.1	1.1E-04	**6.6E-04**
Insular cortex	3.0	4.7E-02	7.1E-02
Medial frontal gyrus	1.6	2.1E-01	2.4E-01
Precuneal cortex	8.6	1.8E-04	**9.1E-04**
**DTI measures**			
Fractional anisotropy			
Global	5.4	4.4E-03	**1.1E-02**
Body of corpus callosum	2.8	5.8E-02	7.8E-02
Genu of corpus callosum	5.8	3.2E-03	**8.2E-03**
Splenium of corpus callosum	2.7	6.5E-02	8.3E-02
Cingulum cingulate	2.3	1.0E-01	1.3E-01
Cingulum hippocampus	3.7	2.4E-02	**3.9E-02**
Uncinate	1.3	2.7E-01	2.9E-01
Mean diffusivity			
Global	4.7	9.5E-03	**1.9E-02**
Body of corpus callosum	3.3	3.6E-02	5.7E-02
Genu of corpus callosum	6.3	1.8E-03	**5.3E-03**
Splenium of corpus callosum	3.9	2.0E-02	**3.5E-02**
Cingulum cingulate	0.5	6.3E-01	6.7E-01

For peer review only

Cingulum hippocampus	11.6	9.0E-06	**1.1E-04**
Uncinate	2.0	1.3E-01	1.6E-01

For peer review only

Post-hoc Dunnett's test

Systemic GC vs. controls				Inhaled GC vs
Estimate	Lower CI	Upper CI	P value	Estimate
-2535	-18869	13798	0.90	3553
-1552	-10808	7703	0.89	1636
-984	-11702	9735	0.96	1917
-2152	-10481	6177	0.78	940
-2408	-7198	2381	0.43	154
-9.9	-42	22.1	0.49	-1.8
52.1	-19.3	123.5	0.19	-20.6
112.7	-12.9	238.2	0.09	-5.0
59.2	-79.1	197.5	0.54	-38.4
4.01	-68.2	76.2	0.98	-23.0
-65.4	-222.8	92.0	0.55	-26.1
61.9	-120.7	244.5	0.66	-11.6
4.8	-60.8	70.3	0.97	-15.1
79.6	-147.1	306.0	0.65	57.1
25.7	-76.5	127.9	0.79	4.4
36.0	-158.8	230.7	0.87	25.5
63.5	-52.4	179.5	0.37	-24.3
-110.3	-280.0	59.3	0.26	34.4
170.0	-201.0	541.2	0.49	-59.9
-0.0066	-0.013	-3.2E-04	**0.038**	-0.0025
-0.0066	-0.016	0.0032	0.24	-0.0028
-0.014	-0.025	-0.0031	**8.7E-03**	-0.0020
-0.0049	-0.012	0.0028	0.27	-0.0032
0.00033	-0.010	0.0109	0.99	-0.0034
0.0032	-0.0078	0.014	0.73	-0.0034
9.4E-06	8.7E-08	1.9E-05	**0.048**	2.6E-06
1.1E-05	-1.6E-06	2.3E-05	0.10	4.4E-06
2.0E-05	5.5E-06	3.5E-05	**4.3E-03**	2.8E-06
8.1E-06	-2.4E-06	1.9E-05	0.16	5.2E-06
3.5E-06	-5.3E-06	1.2E-05	0.58	2.0E-06

For peer review only

8.2E-06	-2.4E-06	1.9E-05	0.16	6.3E-06
6.5E-06	-3.4E-06	1.6E-05	0.25	2.0E-06

For peer review only

[illegible]. controls

Lower CI	Upper CI	P value
-2340	9445	0.31
-1703	4975	0.45
-1950	5784	0.44
-2065	3945	0.70
-1573	1882	0.96
-11.7	11.4	0.97
-46.4	5.2	0.14
-50.3	40.3	0.95
-88.3	11.5	0.16
-49.0	3.1	0.094
-82.9	30.7	0.49
-77.5	54.3	0.88
-38.8	8.5	0.27
-24.7	139.0	0.22
-32.4	41.3	0.94
-44.8	95.8	0.63
-66.1	17.6	0.34
-26.8	95.6	0.36
-194.0	74.1	0.51
-0.0048	-2.3E-04	**0.027**
-0.0063	7.2E-04	0.14
-0.0060	0.0020	0.44
-0.0060	-4.9E-04	**0.017**
-0.0072	4.5E-04	0.093
-0.0074	6.4E-04	0.11
-7.7E-07	6.0E-06	0.16
4.7E-08	8.8E-06	**0.05**
-2.5E-06	8.0E-06	0.40
1.4E-06	9.0E-06	**4.4E-03**
-1.2E-06	5.2E-06	0.28

For peer review only

2.5E-06	1.0E-05	**5.0E-04**
-1.6E-06	5.6E-06	0.36

For peer review only

Primary comparison: systemic GC / inhaled GC vs. controls

	ANOVA of cognitive parameters			Post-hoc Dun
				Systemic GC v
	F value	P value	P_FDR	Estimate
Reaction time	1.0	0.37	0.41	
Fluid intelligence score	0.89	0.41	0.41	
Digit span	1.4	0.25	0.38	
Trail making A	5.6	0.0036	0.0073	-0.11
Trail making B	6.1	0.0023	0.0068	-0.12
Symbol substitution	10.3	3.5E-05	2.1E-04	-0.17

For peer review only

nett's test /s. controls			Inhaled GC vs. controls		
Lower CI	Upper CI	P value	Estimate	Lower CI	Upper CI
-0.28	0.06	0.25	-0.03	-0.15	0.09
-0.30	0.05	0.19	-0.01	-0.13	0.11
-0.34	-0.008	**0.04**	-0.04	-0.15	0.08

For peer review only

P value
0.78
0.98
0.72

For peer review only

Primary comparison: systemic GC / inhaled GC vs. controls

	**Likelihood ratio test of emotional parameters**			**Post-hoc pair**
				**Systemic GC v**
	**Chi squared statistic**	**P value**	**P_FDR**	**OR**
Depression	10.6	4.9E-03	4.9E-03	1.76
Disinterest	10.9	4.3E-03	4.9E-03	1.84
Tenseness	13.4	1.2E-03	2.5E-03	1.78
Tiredness	32.4	9.2E-08	3.7E-07	1.90

For the pairwise comparisons, P values in bold are statistically significant after Bonferroni correctio

For peer review only

wise comparisons

ʋs. controls			Inhaled GC vs. controls		
95% CI	P value		OR	95% CI	P value
1.25; 2.43	**8.2E-04**		1.10	0.87; 1.38	0.43
1.29; 2.56	**5.1E-04**		1.06	0.82; 1.36	0.64
1.29; 2.41	**3.0E-04**		1.16	0.92; 1.43	0.19
1.45; 2.50	**4.4E-06**		1.35	1.14; 1.60	**6.3E-04**

ın for family-wise error rate of two tests (P < 0.025)

For peer review only

Subanalysis: chronic systemic GC / chronic inhaled GC vs. controls

	ANOVA of cognitive parameters			Post-hoc Dunn
				Systemic GC v
	F value	P value	P_FDR	Estimate
Reaction time	0.17	0.84	0.84	
Fluid intelligence score	1.1	0.34	0.84	
Digit span	3.5	0.03	0.19	
Trail making A	0.41	0.66	0.84	0.12
Trail making B	0.28	0.75	0.84	-0.08
Symbol substitution	0.35	0.70	0.84	-0.08

For peer review only

nett's test					
ıs. controls			Inhaled GC vs. controls		
Lower CI	Upper CI	P value	Estimate	Lower CI	Upper CI
-0.26	0.51	0.69	-0.07	-0.24	0.10
-0.47	0.31	0.84	-4.7E-04	-0.17	0.17
-0.45	0.30	0.84	-0.05	-0.21	0.11

For peer review only

P value
0.55
1.00
0.71

For peer review only

Subanalysis: chronic systemic GC / chronic inhaled GC vs. controls

	**Likelihood ratio test of emotional parameters**			**Post-hoc pair[illegible]**
				**Systemic GC v[illegible]**
	**Chi squared statistic**	**P value**	**P_FDR**	**OR**
Depression	1.1	0.57	0.57	1.21
Disinterest	2.2	0.33	0.44	1.41
Tenseness	2.5	0.28	0.44	1.84
Tiredness	4.4	0.11	0.44	0.96

For the pairwise comparisons, P values in bold are statistically significant after Bonferroni c[illegible]

For peer review only

wise comparisons				
/s. controls		Inhaled GC vs. controls		
95% CI	P value	OR	95% CI	P value
0.45; 2.73	0.67	0.85	0.59; 1.18	0.34
0.53; 3.17	0.44	0.79	0.53; 1.13	0.21
0.84; 3.68	0.10	1.05	0.78; 1.40	0.73
0.49; 1.84	0.91	1.28	1.01; 1.61	**3.7E-02**

:orrection for family-wise error rate of two tests (P < 0.025)

For peer review only

Sensitivity analysis among participants without exclusion based on p

ANOVA of imaging parameters

	F value	P value	P_FDR
**Volumetric measures**			
Global volumes			
Total brain volume	17.7	2.2E-08	**1.3E-07**
Grey matter volume	22.3	2.0E-10	**2.4E-09**
White matter volume	5.5	4.1E-03	**6.7E-03**
Peripheral cortex	24.6	2.0E-11	**4.4E-10**
CSF volume	14.2	7.1E-07	**2.3E-06**
Subcortical volumes			
Accumbens	10.2	3.8E-05	**1.0E-04**
Amygdala	1.5	2.2E-01	2.4E-01
Caudate	4.5	1.1E-02	**1.7E-02**
Hippocampus	2.1	1.2E-01	1.5E-01
Pallidum	6.9	1.0E-03	**1.9E-03**
Putamen	9.8	5.6E-05	**1.5E-04**
Thalamus	9.3	9.4E-05	**2.3E-04**
Regional grey matter volumes			
Amygdala	21.0	7.8E-10	**7.0E-09**
Caudate	12.3	4.7E-06	**1.4E-05**
Cerebellum	5.8	3.1E-03	**5.2E-03**
Cingulate gyrus, anterior	1.6	2.0E-01	2.2E-01
Cingulate gyrus, posterior	0.6	5.3E-01	5.5E-01
Cuneal cortex	1.5	2.2E-01	2.4E-01
Hippocampus	1.8	1.6E-01	1.9E-01
Insular cortex	8.7	1.7E-04	**3.5E-04**
Medial frontal gyrus	0.6	5.7E-01	5.7E-01
Precuneal cortex	4.0	1.9E-02	**2.7E-02**
**DTI measures**			
Fractional anisotropy			
Global	15.5	1.8E-07	**9.4E-07**
Body of corpus callosum	8.9	1.4E-04	**3.1E-04**
Genu of corpus callosum	15.2	2.5E-07	**1.1E-06**
Splenium of corpus callosum	2.2	1.1E-01	1.3E-01
Cingulum cingulate	3.8	2.3E-02	**3.1E-02**
Cingulum hippocampus	2.7	6.4E-02	8.6E-02
Uncinate	2.5	8.2E-02	1.1E-01
Mean diffusivity			
Global	24.5	2.4E-11	**4.4E-10**
Body of corpus callosum	14.2	6.7E-07	**2.3E-06**
Genu of corpus callosum	17.9	1.7E-08	**1.2E-07**
Splenium of corpus callosum	6.7	1.2E-03	**2.2E-03**

For peer review only

Cingulum cingulate	4.9	7.6E-03	**1.2E-02**
Cingulum hippocampus	14.5	4.9E-07	**2.0E-06**
Uncinate	7.3	6.6E-04	**1.3E-03**

For peer review only

psychiatric, neurological or endocrine history (systemic GC / inhaled GC vs. c

Post-hoc Dunnett's test				
Oral GC vs. controls				Inhaled GC vs
Estimate	Lower CI	Upper CI	P value	Estimate
-3460	-9320	2400	0.32	3535
-2224	-5577	1130	0.25	1454
-1237	-5078	2604	0.69	2080
-3318	-6330	-307	**0.028**	1172
1220	-518	2958	0.12	223
-8.9	-20.4	2.7	0.16	-3.7
58.6	13.8	103.5	**7.2E-03**	-5.9
1.2	-24.5	27.0	0.99	-16.2
-33.8	-90.5	22.9	0.32	-20.1
-19.9	-86.2	46.5	0.72	-10.7
-6.7	-30.4	17.1	0.75	-21.7
149.6	66.9	232.4	**1.0E-04**	42.9
17.8	-19.4	54.9	0.47	-2.9
-42.1	-103.5	19.4	0.23	8.0
-9.7	-142.8	123.4	0.97	-1.7
-0.0031	-0.0055	-7.5E-04	**6.6E-03**	-0.0015
-0.0039	-0.0076	-0.0003	**0.032**	-0.0014
-0.0056	-0.0097	-0.0014	**5.5E-03**	-0.0013
-0.0014	-0.0052	0.0025	0.64	-0.0018
6.6E-06	3.0E-06	1.0E-05	**3.7E-05**	1.9E-06
6.7E-06	1.9E-06	1.1E-05	**3.4E-03**	4.0E-06
8.0E-06	2.5E-06	1.4E-05	**2.3E-03**	3.3E-06
3.7E-06	-3.1E-07	7.6E-06	0.076	4.0E-06

For peer review only

2.5E-06	-6.8E-07	5.7E-06	0.15	2.2E-06
2.6E-06	-1.3E-06	6.6E-06	0.25	4.5E-06
4.0E-06	2.9E-07	7.7E-06	**0.032**	1.6E-06

For peer review only

controls)

s. controls

Lower CI	Upper CI	P value
-121	7190	0.060
-637	3546	0.22
-316	4476	0.10
-706	3051	0.29
-861	1307	0.65
-10.9	3.5	0.41
-33.9	22.1	0.84
-32.3	-0.2	**0.047**
-55.5	15.3	0.35
-52.1	30.7	0.78
-36.5	-6.8	**2.3E-03**
-8.7	94.5	0.12
-26.1	20.3	0.93
-30.3	46.3	0.84
-84.7	81.3	1.00
-0.0030	-4.9E-05	**0.041**
-0.0036	8.9E-04	0.30
-0.0039	0.0013	0.44
-0.0042	5.9E-04	0.17
-3.2E-07	4.1E-06	0.057
1.1E-06	7.0E-06	**4.8E-03**
-1.4E-07	6.7E-06	0.062
1.5E-06	6.4E-06	**7.0E-04**

For peer review only

2.2E-07	4.2E-06	**0.026**
2.0E-06	7.0E-06	**1.0E-04**
-7.5E-07	3.9E-06	0.23

For peer review only

Sensitivity analysis among participants without exclusion based on psychia

	**ANOVA**			**Post-hoc Dun**
				**Systemic GC v**
	**F value**	**P value**	**P_FDR**	**Estimate**
Reaction time	1.0	0.35	0.42	
Fluid intelligence score	1.9	0.15	0.23	
Digit span	0.5	0.63	0.63	
Trail making A	6.6	0.0014	**0.0028**	-0.11
Trail making B	6.7	0.0013	**0.0028**	-0.12
Symbol substitution	9.7	6.2E-05	**0.00037**	-0.15

For peer review only

atric, neurological or endocrine history (systemic GC / inhaled GC vs. contrc

nett's test

ıs. controls			Inhaled GC vs. controls		
Lower CI	Upper CI	P value	Estimate	Lower CI	Upper CI
-0.26	0.03	0.16	0.020	-0.08	0.12
-0.27	0.02	0.10	-0.018	-0.12	0.08
-0.29	-0.01	**0.03**	-0.061	-0.16	0.04

For peer review only

ɔls)

P value
0.86
0.88
0.28

For peer review only

Sensitivity analysis among participants without exclusion based on psyc

	Likelihood ratio test of emotional parameters			Post-hoc pairv
				Systemic GC v
	**Chi squared statistic**	**P value**	**P_FDR**	**OR**
Depression	11.1	0.0039	3.9E-03	1.44
Disinterest	17.8	0.00014	1.9E-04	1.73
Tenseness	24.0	6.1E-06	1.2E-05	1.68
Tiredness	39.2	3.1E-09	1.2E-08	1.79

For the pairwise comparisons, P values in bold are statistically significant after Bonferroni co

For peer review only

:hiatric, neurological or endocrine history (systemic GC / inhaled GC vs. con

wise comparisons

/s. controls			Inhaled GC vs. controls		
95% CI	P value		OR	95% CI	P value
1.08; 1.89	**0.010**		1.23	1.03; 1.46	**0.023**
1.31; 2.27	**8.5E-05**		1.21	1.00; 1.45	0.041
1.29; 2.16	**7.0E-05**		1.31	1.11; 1.54	**1.4E-03**
1.42; 2.27	**9.0E-07**		1.33	1.15; 1.53	**1.1E-04**

rrection for family-wise error rate of two tests (P < 0.025)

For peer review only

ntrols)

For peer review only

	Systemic GC users (n = 222)	
	**Number of outliers**	**%**
**Volumetric measures**		
Global volumes		
Total brain volume	2	0.9
Grey matter volume	6	2.7
White matter volume	2	0.9
Peripheral cortex	6	2.7
CSF volume	9	4.1
Subcortical volumes		
Accumbens	2	0.9
Amygdala	3	1.4
Caudate	5	2.3
Hippocampus	3	1.4
Pallidum	2	0.9
Putamen	7	3.2
Thalamus	4	1.8
Regional grey matter volumes		
Amygdala	5	2.3
Caudate	18	8.1
Cerebellum	11	5.0
Cingulate gyrus, anterior	7	3.2
Cingulate gyrus, posterior	5	2.3
Cuneal cortex	3	1.4
Hippocampus	4	1.8
Insular cortex	6	2.7
Medial frontal gyrus	3	1.4
Precuneal cortex	7	3.2
**DTI measures**		
Fractional anisotropy		
Global	2	0.9
Body of corpus callosum	4	1.8
Genu of corpus callosum	10	4.5
Splenium of corpus callosum	4	1.8
Cingulum cingulate	4	1.8
Cingulum hippocampus	3	1.4
Uncinate	1	0.5
Mean diffusivity		
Global	7	3.2
Body of corpus callosum	3	1.4
Genu of corpus callosum	4	1.8
Splenium of corpus callosum	5	2.3
Cingulum cingulate	6	2.7

For peer review only

Cingulum hippocampus	4	1.8
Uncinate	4	1.8

For peer review only

Inhaled GC users (n = 557)		Controls (n = 24106)	
Number of outliers	%	Number of outliers	%
1	0.2	134	0.6
1	0.2	153	0.6
3	0.5	130	0.5
1	0.2	159	0.7
22	3.9	859	3.6
7	1.3	195	0.8
6	1.1	217	0.9
2	0.4	269	1.1
11	2.0	399	1.7
4	0.7	490	2.0
1	0.2	237	1.0
6	1.1	225	0.9
7	1.3	281	1.2
27	4.8	1138	4.7
6	1.1	316	1.3
21	3.8	758	3.1
11	2.0	359	1.5
2	0.4	304	1.3
7	1.3	248	1.0
2	0.4	239	1.0
4	0.7	229	0.9
2	0.4	216	0.9
12	2.2	388	1.6
13	2.3	456	1.9
12	2.2	520	2.2
20	3.6	331	1.4
4	0.7	215	0.9
4	0.7	277	1.1
6	1.1	221	0.9
11	2.0	613	2.5
15	2.7	468	1.9
11	2.0	535	2.2
12	2.2	543	2.3
10	1.8	447	1.9

For peer review only

16	2.9	343	1.4
6	1.1	322	1.3

For peer review only

Sensitivity analysis after exclusion of outlier values

ANOVA of imaging parameters

	F value	P value	P_FDR
**Volumetric measures**			
Global volumes			
Total brain volume	16.0	1.1E-07	**4.6E-07**
Grey matter volume	28.8	3.4E-13	**6.1E-12**
White matter volume	5.4	4.6E-03	**7.1E-03**
Peripheral cortex	27.0	2.0E-12	**1.8E-11**
CSF volume	16.8	5.0E-08	**2.3E-07**
Subcortical volumes			
Accumbens	13.0	2.3E-06	**5.8E-06**
Amygdala	2.7	6.9E-02	7.7E-02
Caudate	4.7	8.8E-03	**1.1E-02**
Hippocampus	5.4	4.7E-03	**7.1E-03**
Pallidum	4.9	7.4E-03	**9.8E-03**
Putamen	13.7	1.1E-06	**3.4E-06**
Thalamus	10.0	4.6E-05	**8.7E-05**
Regional grey matter volumes			
Amygdala	28.3	5.1E-13	**6.1E-12**
Caudate	12.6	3.5E-06	**8.4E-06**
Cerebellum	10.3	3.3E-05	**6.6E-05**
Cingulate gyrus, anterior	3.9	2.1E-02	**2.6E-02**
Cingulate gyrus, posterior	2.3	1.0E-01	1.1E-01
Cuneal cortex	1.5	2.2E-01	2.2E-01
Hippocampus	3.3	3.9E-02	**4.6E-02**
Insular cortex	13.1	2.0E-06	**5.5E-06**
Medial frontal gyrus	0.4	6.8E-01	6.8E-01
Precuneal cortex	5.2	5.4E-03	**7.5E-03**
**DTI measures**			
Fractional anisotropy			
Global	22.7	1.4E-10	**1.0E-09**
Body of corpus callosum	11.4	1.1E-05	**2.5E-05**
Genu of corpus callosum	15.3	2.3E-07	**8.4E-07**
Splenium of corpus callosum	2.7	6.4E-02	7.5E-02
Cingulum cingulate	6.5	1.5E-03	**2.5E-03**
Cingulum hippocampus	7.5	5.7E-04	**9.7E-04**
Uncinate	2.6	7.6E-02	8.3E-02
Mean diffusivity			
Global	29.1	2.4E-13	**6.1E-12**
Body of corpus callosum	17.1	3.6E-08	**1.9E-07**
Genu of corpus callosum	21.6	4.3E-10	**2.6E-09**
Splenium of corpus callosum	9.9	5.2E-05	**9.4E-05**

For peer review only

Cingulum cingulate	5.3	5.2E-03	**7.5E-03**
Cingulum hippocampus	13.7	1.1E-06	**3.4E-06**
Uncinate	11.3	1.2E-05	**2.5E-05**

P values in blue were not significant (P < 0.05) in the original analysis, but are in this analysis

For peer review only

Post-hoc Dunnett's test

Systemic GC vs. controls				Inhaled GC vs
**Estimate**	**Lower CI**	**Upper CI**	**P value**	**Estimate**
-3991	-10852	2869	0.33	3756
-3143	-7081	794	0.14	1120
-1861	-6349	2626	0.55	2374
-4412	-7948	-876	0.011	1148
1437	-210	3084	0.10	-449
-15.6	-28.8	-2.3	0.018	-4.6
69.4	18.4	120.3	**4.9E-03**	4.5
-17.1	-71.2	37.0	0.70	-17
5.7	-20.5	31.8	0.83	-9.8
-63	-127.1	1.0	0.055	-19.9
-25.6	-98.2	46.9	0.64	-0.6
-17.2	-43.8	9.4	0.26	-22.6
138.1	67.7	208.6	**<.0001**	15.1
-1.1	-42.8	40.6	1.00	-6.6
110.5	-7.8	229.0	0.071	27.1
24.3	-22.4	70.9	0.41	2.4
-74.8	-143.2	-6.4	0.029	8.7
-60.1	-213.6	93.3	0.59	0.014
-0.0043	-0.0067	-0.0018	**2.0E-04**	-0.0019
-0.0048	-0.0086	-0.0010	**9.7E-03**	-0.0021
-0.0059	-0.010	-0.0016	**4.8E-03**	-0.0017
-0.0022	-0.0065	0.0021	0.42	-0.0026
-1.2E-04	-0.0046	0.0044	1.00	-0.0036
7.1E-06	3.7E-06	1.1E-05	**<.0001**	2.5E-06
7.5E-06	2.8E-06	1.2E-05	**7.0E-04**	3.7E-06
9.5E-06	3.9E-06	1.5E-05	**3.0E-04**	3.6E-06
4.6E-06	7.3E-07	8.4E-06	0.016	4.2E-06

For peer review only

2.6E-06	-9.4E-07	6.1E-06	0.19	2.6E-06
4.4E-06	2.5E-07	8.6E-06	**0.035**	4.3E-06
5.8E-06	1.9E-06	9.7E-06	**1.8E-03**	2.4E-06

after exclusion of outliers.

For peer review only

. controls

Lower CI	Upper CI	P value
-565	8076	0.10
-1337	3576	0.50
-454	5203	0.11
-1058	3355	0.41
-1492	594	0.53
-13.0	3.7	0.37
-27.4	36.3	0.92
-51.3	17.3	0.44
-26.3	6.7	0.32
-59.7	20.0	0.44
-46.2	45.1	1.00
-39.3	-5.9	**0.01**
-28.8	59.1	0.66
-32.5	19.3	0.78
-47.9	102.0	0.63
-27.0	31.8	0.97
-34.1	51.4	0.85
-95.6	95.6	1.00
-0.0035	-3.4E-04	**0.013**
-0.0045	3.4E-04	0.11
-0.0044	0.0010	0.28
-0.0053	9.7E-05	0.061
-0.0064	-7.5E-04	**0.010**
3.1E-07	4.7E-06	**0.022**
6.9E-07	6.6E-06	**0.012**
2.9E-08	7.1E-06	**0.048**
1.8E-06	6.7E-06	**2.0E-04**

For peer review only

3.6E-07	4.8E-06	**0.019**
1.6E-06	6.9E-06	**6.0E-04**
-8.8E-08	4.8E-06	0.061

For peer review only

	Systemic GC users (n = 222)		Inhaled GC users (n = 557)
	**Number of outliers**	**%**	**Number of outliers**
Reaction time*	4	1.8	11
Fluid intelligence score	2	0.9	1
Digit span	1	0.5	24
Trail making A*	6	2.7	10
Trail making B*	2	0.9	2
Symbol substitution	5	2.3	3

* Reaction time, trail making A, and trail making B were log transformed for normalization.

For peer review only

	Controls (n = 24106)	
%	Number of outliers	%
2.0	389	1.6
0.2	50	0.2
4.3	79	0.3
1.8	423	1.8
0.4	338	1.4
0.5	172	0.7

For peer review only

Sensitivity analysis after exclusion of outlier values

	ANOVA			Post-hoc Dun[illegible]
				Systemic GC v[illegible]
	F value	P value	P_FDR	Estimate
Reaction time	1.0	0.38	0.45	
Fluid intelligence score	0.8	0.45	0.45	
Digit span	3.1	0.047	0.070	
Trail making A	5.2	5.7E-03	**0.011**	-0.10
Trail making B	9.6	6.8E-05	**2.0E-04**	-0.16
Symbol substitution	11.6	8.9E-06	**5.3E-05**	-0.18

For peer review only

nett's test

/s. controls				Inhaled GC vs. controls		
Lower CI	Upper CI	P value		Estimate	Lower CI	Upper CI
-0.25	0.05	0.25		-0.02	-0.12	0.09
-0.32	-0.01	**0.04**		-0.06	-0.17	0.04
-0.34	-0.02	**0.02**		-0.05	-0.16	0.06

For peer review only

P value
0.88
0.31
0.55

For peer review only

Mediation analysis for total body fat percentage

Region		Estimate
Caudate, subcortical volume	ACME	-1.2
	ADE	78.5
	Total effect	77.4
	Proportion mediated	0.0
Amygdala, regional grey matter volume	ACME	-1.2
	ADE	1.2
	Total effect	-0.1
	Proportion mediated	17.7
Caudate, regional grey matter volume	ACME	-0.9
	ADE	184.0
	Total effect	183.0
	Proportion mediated	0.0
Global FA	ACME	-2.5E-05
	ADE	-3.8E-03
	Total effect	-3.8E-03
	Proportion mediated	6.6E-03
Global MD	ACME	-4.8E-08
	ADE	7.5E-06
	Total effect	7.5E-06
	Proportion mediated	-6.4E-03

ACME, average causal mediation effects; ADE, average direct effects.

For peer review only

Lower CI	Upper CI	P value	Total sample size used
-3.7	1.0	0.27	23338
31.2	124.6	<2e-16	
30.0	123.3	<2e-16	
-0.1	0.0	0.27	
-3.6	1.0	0.29	23338
-24.2	26.7	0.93	
-25.7	26.0	0.98	
-1.9	1.9	0.94	
-3.1	0.7	0.27	23338
79.4	293.5	<2e-16	
77.9	293.0	<2e-16	
0.0	0.0	0.27	
-8.6E-05	0	0.3	23338
-6.3E-03	0	<2e-16	
-6.4E-03	0	<2e-16	
-5.5E-03	4.0E-02	0.3	
-1.5E-07	0	0.31	23338
3.7E-06	0	<2e-16	
3.7E-06	0	<2e-16	
-2.5E-02	1.0E-02	0.31	

For peer review only

Supplements

Supplements 1, 2 and 3 are separate files.

For peer review only

Supplement 4. Violin plots of the imaging outcomes for the main analysis. AU, arbitrary units; GC, glucocorticoids

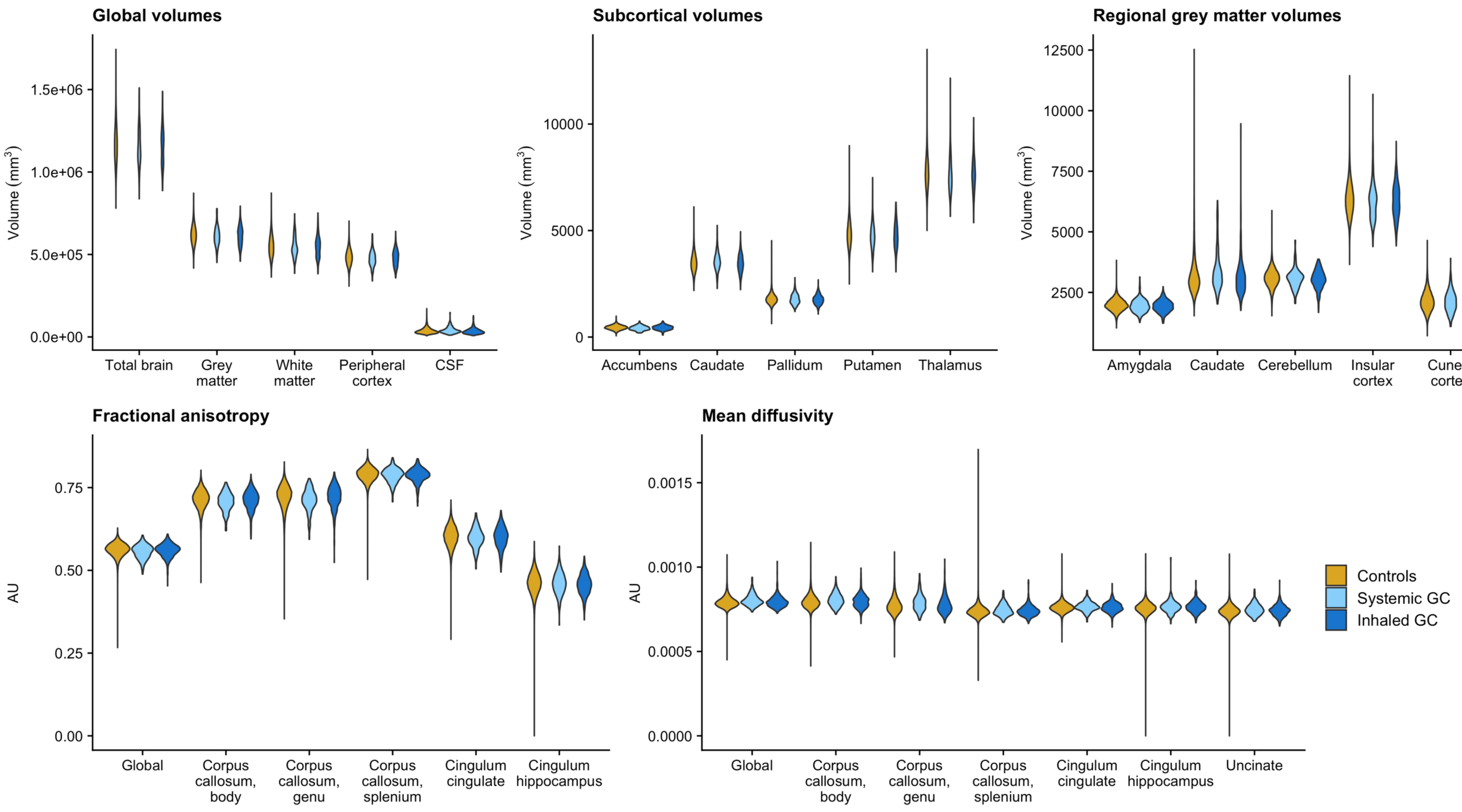

Supplement 5. Characteristics of included chronic glucocorticoid users and controls

	Patients using chronic systemic GC (n = 42)	Patients using chronic inhaled GC (n = 305)	Controls (n = 24106)	P value
**Sex: male, n (%)**	22 (52.4%)	137 (44.9%)	12154 (50.4%)	0.15
**Age at time of scanning in years, mean (SD)**	65.2 (7.0)	63.0 (7.6)	63.5 (7.5)	0.19
**Education level, n (%)**				0.81
College/University degree	24 (57.1)	171 (56.1)	12058 (50.0)	
A levels or equivalent	6 (14.3)	38 (12.5)	2930 (12.2)	
O levels/GCSE or equivalent	4 (9.5)	44 (14.4)	4155 (17.2)	
CSEs or equivalent	1 (2.4)	9 (3.0)	879 (3.6)	
NVQ, HND, HNC, or equivalent	1 (2.4)	14 (4.6)	1396 (5.8)	
Other professional qualifications	2 (4.8)	14 (4.6)	1150 (4.8)	
None of the above	1 (2.4)	14 (4.6)	1311 (5.4)	
Missing	3 (7.1)	1 (0.3)	227 (0.9)	
**BMI in kg/m^2, mean (SD)**	25.9 (3.7)	26.6 (4.4)	26.1 (4.1)	0.15
Number (%) missing	1 (2.4)	14 (4.6)	1325 (5.5)	
**Body fat percentage, mean (SD)**	30.0 (6.4)	32.0 (8.1)	30.2 (7.9)	4.6e-4
Number (%) missing	1 (2.4)	14 (4.6)	1331 (5.5)	
**Smoking status, n (%)**				0.42
Current	1 (2.4)	6 (2.0)	647 (2.7)	
Previous	8 (19.0)	112 (36.7)	7858 (32.6)	
Never	31 (73.8)	206 (67.5)	15380 (63.8)	
Missing	2 (4.8)	2 (0.7)	221 (0.9)	

For peer review only

BMI, body mass index; GC, glucocorticoids; n, number; SD, standard deviation.

P values were determined using analysis of variance (for continuous variables) and Fisher's exact test (for categorical variables, because of the low number of patients using chronic glucocorticoids).

For peer review only

Supplement 6. Imaging parameters, presented as the adjusted mean difference of patients using chronic systemic glucocorticoids (n = 42) or chronic inhaled glucocorticoids (n = 305) compared to controls (n = 24106)

	ANOVA			Systemic GC vs. controls			Inhaled GC vs. controls		
	**F value**	**P value**	**P_{FDR}**	**AMD***	**95% CI**	**P value**	**AMD***	**95% CI**	**P value**
Volumetric measures									
Global volumes (in mm^3)									
Total brain volume	17.0	4.1e-8	**1.5e-6**	-2535	-18869; 13798	0.90	3553	-2340; 9445	0.31
Grey matter volume	12.2	5.0e-6	**9.1e-5**	-1552	-10808; 7703	0.89	1636	-1703; 4975	0.45
White matter volume	10.8	2.0e-5	**1.8e-4**	-984	-11702; 9735	0.96	1917	-1950; 5784	0.44
Peripheral cortex	8.5	2.1e-4	**9.4e-4**	-2152	-10481; 6177	0.78	940	-2065; 3945	0.70
CSF volume	3.0	5.2e-2	**7.4e-2**	-2408	-7198; 2381	0.43	154	-1573; 1882	0.96
Subcortical volumes (in mm^3)									
Amygdala	5.8	2.9e-3	**8.2e-3**	52.1	-19.3; 123.5	0.19	-20.6	-46.4; 5.2	0.14
Caudate	7.2	7.6e-4	**2.9e-3**	112.7	-12.9; 238.2	0.09	-5.0	-50.3; 40.3	0.95
Hippocampus	4.9	7.8e-3	**1.7e-2**	59.2	-79.1; 197.5	0.54	-38.4	-88.3; 11.5	0.16
Pallidum	7.1	7.9e-4	**2.9e-3**	4.01	-68.2; 76.2	0.98	-23.0	-49.0; 3.1	0.094
Putamen	5.0	6.9e-3	**1.6e-2**	-65.4	-222.8; 92.0	0.55	-26.1	-82.9; 30.7	0.49
Thalamus	6.7	1.3e-3	**4.1e-3**	61.9	-120.7; 244.5	0.66	-11.6	-77.5; 54.3	0.88
Regional grey matter volumes (in mm^3)									
Amygdala	10.1	4.2e-5	**3.0e-4**	4.8	-60.8; 70.3	0.97	-15.1	-38.8; 8.5	0.27
Cerebellum	4.1	1.6e-2	**2.9e-2**	25.7	-76.5; 127.9	0.79	4.4	-32.4; 41.3	0.94
Cingulate gyrus, posterior	4.2	1.6e-2	**2.9e-2**	36.0	-158.8; 230.7	0.87	25.5	-44.8; 95.8	0.63

For peer review only

Hippocampus	9.1	1.1e-4	**6.6e-4**	63.5	-52.4; 179.5	0.37	-24.3	-66.1; 17.6	0.34
Precuneal cortex	8.6	1.8e-4	**9.1e-4**	170.0	-201.0; 541.2	0.49	-59.9	-194.0; 74.1	0.51
DTI measures									
Fractional anisotropy									
Global	5.4	4.4e-3	**1.1e-2**	-0.0066	-0.013; -3.2e-4	**0.038**	-0.0025	-0.0048; -2.3e-4	**0.027**
Genu of corpus callosum	5.8	3.2e-3	**8.2e-3**	-0.014	-0.025; -0.0031	**0.0087**	-0.0020	-0.0060; 0.0020	0.44
Cingulum hippocampus	3.7	2.4e-2	**3.9e-2**	0.0032	-0.0078; 0.014	0.73	-0.0034	-0.0074; 6.4e-4	0.11
Mean diffusivity									
Global	4.7	9.5e-3	**1.9e-2**	9.4e-6	8.7e-8; 1.9e-5	**0.05**	2.6e-6	-7.7e-7; 6.0e-6	0.16
Genu of corpus callosum	6.3	1.8e-3	**5.3e-3**	2.0e-5	5.5e-6; 3.5e-5	**0.0043**	2.8e-6	-2.5e-6; 8.0e-6	0.40
Splenium of corpus callosum	3.9	2.0e-2	**3.5e-2**	8.1e-6	-2.4e-6; 1.9e-5	0.16	5.2e-6	1.4e-6; 9.0e-6	**0.0044**
Cingulum hippocampus	11.6	9.0e-6	**1.1e-4**	8.2e-6	-2.4e-6; 1.9e-5	0.16	6.3e-6	2.5e-6; 1.0e-5	**5.0e-4**

* Adjusted mean difference, calculated using linear models, adjusted for age, sex, education, X-, Y-, and Z-position of the head in the scanner, head size, assessment centre, and year of imaging acquisition.

CI, confidence interval; P_{FDR}, Benjamini-Hochberg false discovery rate corrected P values; SE, standard error. P values in bold are statistically significant (P < 0.05).

For peer review only

Supplement 7. Cognitive outcome measures of chronic systemic glucocorticoid users (n = 42) and chronic inhaled glucocorticoid users (n = 305) vs. controls

	ANOVA			Systemic GC vs. controls			Inhaled GC vs. controls			Participants with available data, n (%)		
	F value	P value	P_{FDR}	AMD*	95% CI	P value	AMD*	95% CI	P value	Systemic GC	Inhaled GC	Controls
**Trail making A**	0.41	0.66	0.84	0.12	-0.26; 0.51	0.69	-0.07	-0.24; 0.10	0.55	30 (71)	151 (50)	16419 (68)
**Trail making B**	0.28	0.75	0.84	-0.08	-0.47; 0.31	0.84	0.00	-0.17; 0.17	1.00	28 (67)	148 (49)	16071 (67)
**Symbol substitution**	0.35	0.70	0.84	-0.08	-0.45; 0.30	0.84	-0.05	-0.21; 0.11	0.71	30 (71)	151 (50)	16442 (68)

* Adjusted mean difference between patients and controls, expressed in Z scores. Calculated using linear models, adjusting for age, sex, and education. Trail making A, and trail making B were log transformed before generation of Z scores because they were non-normally distributed. Variables were transformed such that higher values indicate a better performance.

CI, confidence interval; GC, glucocorticoids; n, number; P_{FDR}, Benjamini-Hochberg false discovery rate corrected P values

For peer review only

Supplement 8. Self-reported frequency of mental health parameters in the past two weeks of patients using systemic glucocorticoids (n = 222) or inhaled glucocorticoids (n = 557) and controls, presented as number of participants (%) per category

	**Systemic GC (n=222)**	**Inhaled GC (n=557)**	**Controls (n=24106)**
**Depressed mood**			
Not at all	170 (77)	455 (82)	19940 (83)
Several days	39 (18)	77 (14)	3017 (13)
More than half of the days	6 (2.7)	8 (1.4)	296 (1.2)
Nearly every day	1 (0.5)	3 (0.5)	150 (0.6)
Missing	6 (2.7)	14 (2.5)	703 (2.9)
**Disinterest**			
Not at all	174 (78)	468 (84)	20536 (85)
Several days	34 (15)	61 (11)	2568 (11)
More than half of the days	3 (1.3)	7 (1.3)	292 (1.2)
Nearly every day	5 (2.3)	5 (0.9)	174 (0.7)
Missing	6 (2.7)	16 (2.9)	536 (2.2)
**Tenseness/restlessness**			
Not at all	162 (73)	437 (78)	19412 (81)
Several days	46 (21)	89 (16)	3630 (15)
More than half of the days	3 (1.3)	12 (2.2)	272 (1.1)
Nearly every day	5 (2.3)	5 (0.9)	126 (0.5)
Missing	6 (2.7)	14 (2.5)	666 (2.8)
**Tiredness/lethargy**			
Not at all	95 (43)	280 (50)	13792 (57)
Several days	91 (41)	221 (40)	8345 (35)
More than half of the days	9 (4.1)	32 (5.7)	815 (3.4)
Nearly every day	19 (8.6)	15 (2.7)	555 (2.3)
Missing	8 (3.6)	9 (1.6)	599 (2.5)

GC, glucocorticoids; n, number.

For peer review only

Supplement 9. Self-reported frequency of mental health parameters in the past two weeks of chronic systemic glucocorticoid users (n = 42), chronic inhaled glucocorticoid users (n = 305) and controls, presented as number of participants (%) per category

	**Systemic GC (n = 42)**	**Inhaled GC (n = 305)**	**Controls (n = 24106)**
**Depressed mood**			
Not at all	33 (79)	257 (84)	19940 (83)
Several days	6 (14)	35 (11)	3017 (13)
More than half of the days	0 (0)	3 (0.9)	296 (1.2)
Nearly every day	0 (0)	1 (0.3)	150 (0.6)
Missing	3 (7.1)	9 (3.0)	703 (2.9)
**Disinterest**			
Not at all	34 (81)	267 (88)	20536 (85)
Several days	6 (14)	30 (9.8)	2568 (11)
More than half of the days	0 (0)	1 (0.3)	292 (1.2)
Nearly every day	0 (0)	0 (0)	174 (0.7)
Missing	2 (4.8)	7 (2.3)	536 (2.2)
**Tenseness/restlessness**			
Not at all	30 (71)	245 (80)	19412 (81)
Several days	10 (24)	48 (16)	3630 (15)
More than half of the days	0 (0)	6 (2.0)	272 (1.1)
Nearly every day	0 (0)	1 (0.3)	126 (0.5)
Missing	2 (4.8)	5 (1.6)	666 (2.8)
**Tiredness/lethargy**			
Not at all	24 (57)	156 (51)	13792 (57)
Several days	12 (29)	121 (40)	8345 (35)
More than half of the days	2 (4.8)	14 (4.6)	815 (3.4)
Nearly every day	2 (4.8)	8 (2.6)	555 (2.3)
Missing	2 (4.8)	6 (2.0)	599 (2.5)

GC, glucocorticoids; n, number.

For peer review only

Supplement 10. Likelihood of experiencing mental health complaints in the past two weeks of chronic systemic glucocorticoid users (n = 42) and chronic inhaled glucocorticoid users (n = 305) compared to controls

	Likelihood ratio test			Systemic GC vs. controls			Inhaled GC vs. controls		
	X^2	P value	P_{FDR}	OR	95% CI	P value	OR	95% CI	P value
**Depression**	1.1	0.57	0.57	1.21	0.45; 2.73	0.67	0.85	0.59; 1.18	0.34
**Disinterest**	2.2	0.33	0.44	1.41	0.53; 3.17	0.44	0.79	0.53; 1.13	0.21
**Tenseness**	2.5	0.28	0.44	1.84	0.84; 3.68	0.10	1.05	0.78; 1.40	0.73
**Tiredness**	4.4	0.11	0.44	0.96	0.49; 1.84	0.91	1.28	1.01; 1.61	**0.0037**

Calculated using logistic regression analysis, adjusting for age, sex, and education. P values in bold are statistically significant after Bonferroni correction for family-wise error rate of two tests ($P < 0.025$).

CI, confidence interval; GC, glucocorticoids; OR, odds ratio; P_{FDR}, Benjamini-Hochberg false discovery rate corrected P values.

For peer review only

Supplement 11. Sensitivity analysis: Characteristics of included glucocorticoid users and controls (without exclusion of participants based on psychiatric, neurological, or endocrine history or medication use)

	**Patients using systemic GC (n = 312)**	**Patients using inhaled GC (n = 806)**	**Controls (n = 36310)**	**P value**
**Sex: male, n (%)**	145 (46.5)	344 (42.7)	17041 (46.9)	0.057
**Age at time of scanning in years, mean (SD)**	66.1 (6.9)	62.8 (7.5)	63.7 (7.5)	3.6e-10
**Education level, n (%)**				0.37
College/University degree	143 (45.8)	407 (50.5)	17637 (48.6)	
A levels or equivalent	39 (12.5)	98 (12.2)	4392 (12.1)	
O levels/GCSE or equivalent	53 (17.0)	136 (16.9)	6400 (17.6)	
CSEs or equivalent	13 (4.2)	26 (3.2)	1372 (3.8)	
NVQ, HND, HNC, or equivalent	11 (3.5)	50 (6.2)	2142 (5.9)	
Other professional qualifications	21 (6.7)	45 (5.6)	1795 (4.9)	
None of the above	27 (8.7)	40 (5.0)	2208 (6.1)	
Missing	5 (1.6)	4 (0.5)	364 (1.0)	
**BMI in kg/m^2, mean (SD)**	26.7 (4.4)	27.1 (4.7)	26.5 (4.4)	2.2e-4
Number (%) missing	11 (3.5)	31 (3.8)	1932 (5.3)	
**Body fat percentage, mean (SD)**	31.9 (8.2)	32.6 (8.4)	31.1 (8.1)	5.5e-7
Number (%) missing	11 (3.5)	31 (3.8)	1942 (5.3)	
**Smoking status, n (%)**				0.096
Current	10 (3.2)	25 (3.1)	1231 (3.3)	
Previous	118 (37.8)	299 (37.1)	12063 (33.2)	
Never	181 (58.0)	477 (59.2)	22661 (62.4)	
Missing	3 (1.0)	5 (0.6)	355 (1.0)	

For peer review only

BMI, body mass index; GC, glucocorticoids; n, number; SD, standard deviation.

P values determined using analysis of variance (for continuous variables) and Pearson's Chi squared test (for categorical variables).

For peer review only

Supplement 12. Sensitivity analysis: Imaging parameters, presented as the adjusted mean difference of patients using systemic glucocorticoids (n = 312) or inhaled glucocorticoids (n = 806) compared to controls (n = 36310) (without exclusion of participants based on psychiatric, neurological, or endocrine history or medication use)

	ANOVA			Systemic GC vs. controls			Inhaled GC vs. controls		
	**F value**	**P value**	**P_{FDR}**	**AMD***	**95% CI**	**P value**	**AMD***	**95% CI**	**P value**
Volumetric measures									
Global volumes (in mm^3)									
Total brain volume	17.7	2.2e-8	**1.3e-7**	-3460	-9320; 2400	0.32	3535	-121; 7190	0.060
Grey matter volume	22.3	2.0e-10	**2.4e-9**	-2224	-5577; 1130	0.25	1454	-637; 3546	0.22
White matter volume	5.5	4.1e-3	**6.7e-3**	-1237	-5078; 2604	0.69	2080	-316; 4476	0.10
Peripheral cortex	24.6	2.0e-11	**4.4e-10**	-3318	-6330; -307	**0.028**	1172	-706; 3051	0.29
CSF volume	14.2	7.1e-7	**2.3e-6**	1220	-518; 2958	0.12	223	-861; 1307	0.65
Subcortical volumes (in mm^3)									
Accumbens	10.2	3.8e-5	**1.0e-4**	-8.9	-20.4; 2.7	0.16	-3.7	-10.9; 3.5	0.41
Caudate	4.5	1.1e-2	**1.7e-2**	58.6	13.8; 103.5	**0.0072**	-5.9	-33.9; 22.1	0.84
Pallidum	6.9	1.0e-3	**1.9e-3**	1.2	-24.5; 27.0	0.99	-16.2	-32.3; -0.2	**0.047**
Putamen	9.8	5.6e-5	**1.5e-4**	-33.8	-90.5; 22.9	0.32	-20.1	-55.5; 15.3	0.35
Thalamus	9.3	9.4e-5	**2.3e-4**	-19.9	-86.2; 46.5	0.72	-10.7	-52.1; 30.7	0.78
Regional grey matter volumes (in mm^3)									
Amygdala	21.0	7.8e-10	**7.0e-9**	-6.7	-30.4; 17.1	0.75	-21.7	-36.5; -6.8	**0.0023**
Caudate	12.3	4.7e-6	**1.4e-5**	149.6	66.9; 232.4	**1.0e-4**	42.9	-8.7; 94.5	0.12
Cerebellum	5.8	3.1e-3	**5.2e-3**	17.8	-19.4; 54.9	0.47	-2.9	-26.1; 20.3	0.93

For peer review only

Insular cortex	8.7	1.7e-4	**3.5e-4**	-42.1	-103.5; 19.4	0.23	8.0	-30.3; 46.3	0.84
Precuneal cortex	4.0	1.9e-2	**2.7e-2**	-9.7	-142.8; 123.4	0.97	-1.7	-84.7; 81.3	1.00
DTI measures									
Fractional anisotropy									
Global	15.5	1.8e-7	**9.4e-7**	-0.0031	-0.0055; -7.5e-4	**0.0066**	-0.0015	-0.0030; -4.9e-5	**0.041**
Body of corpus callosum	8.9	1.4e-4	**3.1e-4**	-0.0039	-0.0076; -0.0003	**0.032**	-0.0014	-0.0036; 8.9e-4	0.30
Genu of corpus callosum	15.2	2.5e-7	**1.1e-6**	-0.0056	-0.0097; -0.0014	**0.0055**	-0.0013	-0.0039; 0.0013	0.44
Cingulum cingulate	3.8	2.3e-2	**3.1e-2**	-0.0014	-0.0052; 0.0025	0.64	-0.0018	-0.0042; 5.9e-4	0.17
Mean diffusivity									
Global	24.5	2.4e-11	**4.4e-10**	6.6e-6	3.0e-6; 1.0e-5	**3.7e-5**	1.9e-6	-3.2e-7; 4.1e-6	5.7e-2
Body of corpus callosum	14.2	6.7e-7	**2.3e-6**	6.7e-6	1.9e-6; 1.1e-5	**0.0034**	4.0e-6	1.1e-6; 7.0e-6	**0.0048**
Genu of corpus callosum	17.9	1.7e-8	**1.2e-7**	8.0e-6	2.5e-6; 1.4e-5	**0.0023**	3.3e-6	-1.4e-7; 6.7e-6	0.0622
Splenium of corpus callosum	6.7	1.2e-3	**2.2e-3**	3.7e-6	-3.1e-7; 7.6e-6	0.076	4.0e-6	1.5e-6; 6.4e-6	**7.0e-4**
Cingulum cingulate	4.9	7.6e-3	**1.2e-2**	2.5e-6	-6.8e-7; 5.7e-6	0.15	2.2e-6	2.2e-7; 4.2e-6	**0.026**
Cingulum hippocampus	14.5	4.9e-7	**2.0e-6**	2.6e-6	-1.3e-6; 6.6e-6	0.25	4.5e-6	2.0e-6; 7.0e-6	**1.0e-4**
Uncinate fasciculus	7.3	6.6e-4	**1.3e-3**	4.0e-6	2.9e-7; 7.7e-6	**0.032**	1.6e-6	-7.5e-7; 3.9e-6	0.23

* Adjusted mean difference, calculated using linear models, adjusted for age, sex, education, X-, Y-, and Z-position of the head in the scanner, head size, assessment centre, and year of imaging acquisition.

P_{FDR}, Benjamini-Hochberg false discovery rate corrected P values; SE, standard error. P values in bold are statistically significant ($P < 0.05$).

For peer review only

Supplement 13. Sensitivity analysis: Cognitive outcome measures of systemic glucocorticoid users (n = 312) and inhaled glucocorticoid users (n = 806) vs. controls (n = 36310) (without exclusion of participants based on psychiatric, neurological, or endocrine history or medication use)

	**ANOVA**			**Systemic GC vs. controls**			**Inhaled GC vs. controls**			**Participants with available data, n (%)**		
	**F value**	**P value**	**P_{FDR}**	**AMD***	**95% CI**	**P value**	**AMD***	**95% CI**	**P value**	**Systemic GC**	**Inhaled GC**	**Controls**
**Trail making A**	6.6	0.0014	**0.0028**	-0.11	-0.26; 0.03	0.16	0.020	-0.08; 0.12	0.86	206 (66)	422 (52)	24297 (67)
**Trail making B**	6.7	0.0013	**0.0028**	-0.12	-0.27; 0.02	0.10	-0.018	-0.12; 0.08	0.88	194 (62)	415 (51)	23273 (64)
**Symbol substitution**	9.7	6.2e-5	**0.00037**	-0.15	-0.29; -0.01	**0.029**	-0.061	-0.16; 0.04	0.28	203 (65)	423 (52)	24337 (67)

* Adjusted mean difference between patients and controls, expressed in Z scores. Calculated using linear models, adjusted for age, sex, and education. Trail making A, and trail making B were log transformed before generation of Z scores because they were non-normally distributed. Variables were transformed such that higher values indicate a better performance.

GC, glucocorticoids; P_{FDR}, Benjamini-Hochberg false discovery rate corrected P values.

For peer review only

Supplement 14. Sensitivity analysis: Self-reported frequency of mental health parameters in the past two weeks of patients using systemic glucocorticoids (n = 312) or inhaled glucocorticoids (n = 806) and controls, presented as number of participants (%) per category (without exclusion of participants based on psychiatric, neurological, or endocrine history or medication use)

	**Systemic GC (n = 312)**	**Inhaled GC (n = 806)**	**Controls (n = 36310)**
**Depressed mood**			
Not at all	240 (76.9)	620 (76.9)	29014 (80.0)
Several days	55 (17.6)	139 (17.2)	5197 (14.3)
More than half of the days	8 (2.6)	14 (1.7)	593 (1.6)
Nearly every day	2 (0.6)	14 (1.7)	360 (1.0)
Missing	7 (2.2)	19 (2.4)	1146 (3.2)
**Disinterest**			
Not at all	237 (76.0)	639 (79.3)	29916 (82.4)
Several days	55 (17.6)	118 (14.6)	4583 (12.6)
More than half of the days	8 (2.6)	17 (2.1)	604 (1.7)
Nearly every day	5 (1.6)	12 (1.5)	357 (1.0)
Missing	7 (2.2)	20 (2.5)	850 (2.3)
**Tenseness/restlessness**			
Not at all	221 (70.8)	588 (73.0)	28266 (77.8)
Several days	71 (22.8)	157 (19.5)	6113 (16.8)
More than half of the days	6 (1.9)	23 (2.9)	565 (1.6)
Nearly every day	6 (1.9)	16 (2.0)	313 (0.9)
Missing	8 (2.6)	22 (2.7)	1053 (2.9)
**Tiredness/lethargy**			
Not at all	125 (40.0)	366 (45.4)	19107 (52.6)
Several days	130 (41.7)	321 (39.8)	13373 (36.8)
More than half of the days	22 (7.1)	53 (6.6)	1533 (4.2)
Nearly every day	26 (8.3)	51 (6.3)	1358 (3.7)
Missing	9 (2.9)	15 (1.9)	939 (2.6)

GC, glucocorticoids; n, number.

For peer review only

Supplement 15. Sensitivity analysis: Likelihood of experiencing mental health complaints in the past two weeks of systemic glucocorticoid users (n = 312) and inhaled glucocorticoid users (n = 806) compared to controls (without exclusion of participants based on psychiatric, neurological, or endocrine history or medication use)

	**Likelihood ratio test**			**Systemic GC vs. controls**			**Inhaled GC vs. controls**		
	**X^2**	**P value**	**P_{FDR}**	**OR**	**95% CI**	**P value**	**OR**	**95% CI**	**P value**
**Depression**	11.1	0.0039	0.0039	1.44	1.08; 1.89	**0.010**	1.23	1.03; 1.46	**0.023**
**Disinterest**	17.8	1.4e-4	1.9e-04	1.73	1.31; 2.27	**8.5e-05**	1.21	1.00; 1.45	0.041
**Tenseness**	24.0	6.1e-06	1.2e-05	1.68	1.29; 2.16	**7.0e-05**	1.31	1.11; 1.54	**0.0014**
**Tiredness**	39.2	3.1e-09	1.2e-08	1.79	1.42; 2.27	**9.0e-07**	1.33	1.15; 1.53	**1.1e-4**

Calculated using logistic regression analysis, adjusting for age, sex, and education. P values in bold are statistically significant after Bonferroni correction for family-wise error rate of two tests ($P < 0.025$).

CI, confidence interval; GC, glucocorticoids; OR, odds ratio; P_{FDR}, Benjamini-Hochberg false discovery rate corrected P values.

For peer review only

Supplement 16. Sensitivity analysis: Imaging parameters, presented as the adjusted mean difference of patients using systemic glucocorticoids (n = 222) or inhaled glucocorticoids (n = 557) compared to controls (n = 24106) (after exclusion of outlier values per group per variable)

	ANOVA			Systemic GC vs. controls			Inhaled GC vs. controls		
	**F value**	**P value**	**P_{FDR}**	**AMD***	**95% CI**	**P value**	**AMD***	**95% CI**	**P value**
Volumetric measures									
Global volumes (in mm^3)									
Total brain volume	16.0	1.1e-7	**4.6e-7**	-3991	-10852; 2869	0.33	3756	-565; 8076	0.10
Grey matter volume	28.8	3.4e-13	**6.1e-12**	-3143	-7081; 794	0.14	1120	-1337; 3576	0.50
White matter volume	5.4	4.6e-3	**7.1e-3**	-1861	-6349; 2626	0.55	2374	-454; 5203	0.11
Peripheral cortex	27.0	2.0e-12	**1.8e-11**	-4412	-7948; -876	**0.011**	1148	-1058; 3355	0.41
CSF volume	16.8	5.0e-8	**2.3e-7**	1437	-210; 3084	0.10	-449	-1492; 594	0.53
Subcortical volumes (in mm^3)									
Accumbens	13.0	2.3e-6	**5.8e-6**	-15.6	-28.8; -2.3	**0.018**	-4.6	-13.0; 3.7	0.37
Caudate	4.7	8.8e-3	**1.1e-2**	69.4	18.4; 120.3	**0.0049**	4.5	-27.4; 36.3	0.92
Hippocampus	5.4	4.7e-3	**7.1e-3**	-17.1	-71.2; 37.0	0.70	-17	-51.3; 17.3	0.44
Pallidum	4.9	7.4e-3	**9.8e-3**	5.7	-20.5; 31.8	0.83	-9.8	-26.3; 6.7	0.32
Putamen	13.7	1.1e-6	**3.4e-6**	-63	-127.1; 1.0	0.055	-19.9	-59.7; 20.0	0.44
Thalamus	10.0	4.6e-5	**8.7e-5**	-25.6	-98.2; 46.9	0.64	-0.6	-46.2; 45.1	1.00
Regional grey matter volumes (in mm^3)									
Amygdala	28.3	5.1e-13	**6.1e-12**	-17.2	-43.8; 9.4	0.26	-22.6	-39.3; -5.9	**0.01**
Caudate	12.6	3.5e-6	**8.4e-6**	138.1	67.7; 208.6	**<0.0001**	15.1	-28.8; 59.1	0.66
Cerebellum	10.3	3.3e-5	**6.6e-5**	-1.1	-42.8; 40.6	1.00	-6.6	-32.5; 19.3	0.78

For peer review only

Cingulate gyrus, anterior	3.9	2.1e-2	**2.6e-2**	110.5	-7.8; 229.0	0.071	27.1	-47.9; 102.0	0.63
Hippocampus	3.3	3.9e-2	**4.6e-2**	24.3	-22.4; 70.9	0.41	2.4	-27.0; 31.8	0.97
Insular cortex	13.1	2.0e-6	**5.5e-6**	-74.8	-143.2; -6.4	**0.029**	8.7	-34.1; 51.4	0.85
Precuneal cortex	5.2	5.4e-3	**7.5e-3**	-60.1	-213.6; 93.3	0.59	0.0	-95.6; 95.6	1.00
DTI measures									
Fractional anisotropy									
Global	22.7	1.4e-10	**1.0e-9**	-0.0043	-0.0067; -0.0018	**2.0e-4**	-0.0019	-0.0035; -3.4e-4	**0.013**
Body of corpus callosum	11.4	1.1e-5	**2.5e-5**	-0.0048	-0.0086; -0.0010	**0.0097**	-0.0021	-0.0045; 3.4e-4	0.11
Genu of corpus callosum	15.3	2.3e-7	**8.4e-7**	-0.0059	-0.010; -0.0016	**0.0048**	-0.0017	-0.0044; 0.0010	0.28
Cingulum cingulate	6.5	1.5e-3	**2.5e-3**	-0.0022	-0.0065; 0.0021	0.42	-0.0026	-0.0053; 9.7e-5	0.061
Cingulum hippocampus	7.5	5.7e-4	**9.7e-4**	-0.00012	-0.0046; 0.0044	1.00	-0.0036	-0.0064; -7.5e-4	**0.010**
Mean diffusivity									
Global	29.1	2.4e-13	**6.1e-12**	7.1e-6	3.7e-6; 1.1e-5	**<0.0001**	2.5e-6	3.1e-7; 4.7e-6	**0.022**
Body of corpus callosum	17.1	3.6e-8	**1.9e-7**	7.5e-6	2.8e-6; 1.2e-5	**7.0e-4**	3.7e-6	6.9e-7; 6.6e-6	**0.012**
Genu of corpus callosum	21.6	4.3e-10	**2.6e-9**	9.5e-6	3.9e-6; 1.5e-5	**3.0e-4**	3.6e-6	2.9e-8; 7.1e-6	**0.048**
Splenium of corpus callosum	9.9	5.2e-5	**9.4e-5**	4.6e-6	7.3e-7; 8.4e-6	**0.016**	4.2e-6	1.8e-6; 6.7e-6	**2.0e-4**
Cingulum cingulate	5.3	5.2e-3	**7.5e-3**	2.6e-6	-9.4e-7; 6.1e-6	0.19	2.6e-6	3.6e-7; 4.8e-6	**0.019**
Cingulum hippocampus	13.7	1.1e-6	**3.4e-6**	4.4e-6	2.5e-7; 8.6e-6	**0.035**	4.3e-6	1.6e-6; 6.9e-6	**6.0e-4**
Uncinate fasciculus	11.3	1.2e-5	**2.5e-5**	5.8e-6	1.9e-6; 9.7e-6	**0.0018**	2.4e-6	-8.8e-8; 4.8e-6	0.061

* Adjusted mean difference, calculated using linear models, adjusted for age, sex, education, X-, Y-, and Z-position of the head in the scanner, head size, assessment centre, and year of imaging acquisition.

P_{FDR}, Benjamini-Hochberg false discovery rate corrected P values; SE, standard error. P values in bold are statistically significant (P < 0.05).

For peer review only

Supplement 17. Cognitive outcome measures of systemic glucocorticoid users (n = 222) and inhaled glucocorticoid users (n = 557) vs. controls (after exclusion of outlier values per group per variable)

	ANOVA			Systemic GC vs. controls			Inhaled GC vs. controls			Participants with available data, n (%)		
	**F value**	**P value**	**P_{FDR}**	**AMD***	**95% CI**	**P value**	**AMD***	**95% CI**	**P value**	**Systemic GC**	**Inhaled GC**	**Controls**
**Trail making A**	5.2	0.0057	**0.011**	-0.10	-0.25; 0.05	0.25	-0.018	-0.12; 0.09	0.88	143 (64)	286 (51)	15996 (66)
**Trail making B**	9.6	6.8e-5	**2.0e-4**	-0.16	-0.32; -0.01	**0.038**	-0.064	-0.17; 0.04	0.31	137 (62)	289 (52)	15733 (65)
**Symbol substitution**	11.6	8.9e-6	**5.3e-5**	-0.18	-0.34; -0.02	**0.021**	-0.046	-0.16; 0.06	0.55	141 (64)	295 (53)	16270 (67)

* Adjusted mean difference between patients and controls, expressed in Z scores. Calculated using linear models, adjusted for age, sex, and education. Trail making A, and trail making B were log transformed before generation of Z scores because they were non-normally distributed. Variables were transformed such that higher values indicate a better performance.

GC, glucocorticoids; P_{FDR}, Benjamini-Hochberg false discovery rate corrected P values.

For peer review only

Supplement 18. STROBE Statement – Checklist of items that should be included in reports of cohort studies

	Item No	Recommendation	Where to be found
**Title and abstract**	1	(*a*) Indicate the study's design with a commonly used term in the title or the abstract	Abstract: Design (p.2)
		(*b*) Provide in the abstract an informative and balanced summary of what was done and what was found	Abstract: Main outcome measures, Results (p.2)
**Introduction**			
Background/ rationale	2	Explain the scientific background and rationale for the investigation being reported	Introduction (p.4)
Objectives	3	State specific objectives, including any prespecified hypotheses	Introduction (p.4)
**Methods**			
Study design	4	Present key elements of study design early in the paper	Study design (p.5)
Setting	5	Describe the setting, locations, and relevant dates, including periods of recruitment, exposure, follow-up, and data collection	Study design, Data collection (p.5)
Participants	6	(*a*) Give the eligibility criteria, and the sources and methods of selection of participants. Describe methods of follow-up	Participants (pp.5-6)
		(*b*) For matched studies, give matching criteria and number of exposed and unexposed	*Not applicable*
Variables	7	Clearly define all outcomes, exposures, predictors, potential confounders, and effect modifiers. Give diagnostic criteria, if applicable	Data collection, Imaging data, Cognitive and Emotional data, Statistical analysis (pp.5-9)
Data sources/ measurement	8	For each variable of interest, give sources of data and details of methods of assessment (measurement). Describe comparability of assessment methods if there is more than one group	Data collection, Imaging data, Cognitive and Emotional data (pp.5-7)
Bias	9	Describe any efforts to address potential sources of bias	Statistical analysis (pp.7-9)
Study size	10	Explain how the study size was arrived at	Participants (pp.5-6)
Quantitative variables	11	Explain how quantitative variables were handled in the analyses. If applicable, describe which groupings were chosen and why	Statistical analysis (pp.7-9)
Statistical methods	12	(*a*) Describe all statistical methods, including those used to control for confounding	Statistical analysis (pp.7-9)
		(*b*) Describe any methods used to examine subgroups and interactions	
		(*c*) Explain how missing data were addressed	
		(*d*) If applicable, explain how loss to follow-up was addressed	
		(*e*) Describe any sensitivity analyses	

For peer review only

**Results**			
Participants	13	(a) Report numbers of individuals at each stage of study – e.g. numbers potentially eligible, examined for eligibility, confirmed eligible, included in the study, completing follow-up, and analyzed	Demographic characteristics (p.10) and Figure 1
		(b) Give reasons for non-participation at each stage	
		(c) Consider use of a flow diagram	
Descriptive data	14	(a) Give characteristics of study participants (e.g. demographic, clinical, social) and information on exposures and potential confounders	Demographic characteristics (p.10) and Table 1
		(b) Indicate number of participants with missing data for each variable of interest	
		(c) Summarize follow-up time (e.g., average and total amount)	*Not applicable*
Outcome data	15*	Report numbers of outcome events or summary measures over time	Results (pp.12-19)
Main results	16	(*a*) Give unadjusted estimates and, if applicable, confounder-adjusted estimates and their precision (e.g., 95% confidence interval). Make clear which confounders were adjusted for and why they were included	Results (pp.12-19), Tables 2-4, Supplements
		(*b*) Report category boundaries when continuous variables were categorized	Statistical analysis (p.8)
		(*c*) If relevant, consider translating estimates of relative risk into absolute risk for a meaningful time period	*Not applicable*
Other analyses	17	Report other analyses done – e.g. analyses of subgroups and interactions, and sensitivity analyses	Results (p.20), Supplements
**Discussion**			
Key results	18	Summarize key results with reference to study objectives	Discussion (pp.21-22)
Limitations	19	Discuss limitations of the study, taking into account sources of potential bias or imprecision. Discuss both direction and magnitude of any potential bias	Strengths and limitations (pp.23-25)
Interpretation	20	Give a cautious overall interpretation of results considering objectives, limitations, multiplicity of analyses, results from similar studies, and other relevant evidence	Findings in context, Potential consequences and implications (pp.21-23)
Generalizability	21	Discuss the generalizability (external validity) of the study results	Strengths and limitations (pp.23-25)
**Other information**			
Funding	22	Give the source of funding and the role of the funders for the present study and, if applicable, for the original study on which the present article is based	Funding (p.26)

For peer review only

STROBE Statement – Checklist of items that should be included in reports of cohort studies

	Item No	Recommendation	Where to be found
**Title and abstract**	1	(*a*) Indicate the study's design with a commonly used term in the title or the abstract	Abstract: Design (p.2)
		(*b*) Provide in the abstract an informative and balanced summary of what was done and what was found	Abstract: Main outcome measures, Results (p.2)
**Introduction**			
Background/ rationale	2	Explain the scientific background and rationale for the investigation being reported	Introduction (p.4)
Objectives	3	State specific objectives, including any prespecified hypotheses	Introduction (p.4)
**Methods**			
Study design	4	Present key elements of study design early in the paper	Study design (p.5)
Setting	5	Describe the setting, locations, and relevant dates, including periods of recruitment, exposure, follow-up, and data collection	Study design, Data collection (p.5)
Participants	6	(*a*) Give the eligibility criteria, and the sources and methods of selection of participants. Describe methods of follow-up	Participants (pp.5-6)
		(*b*) For matched studies, give matching criteria and number of exposed and unexposed	*Not applicable*
Variables	7	Clearly define all outcomes, exposures, predictors, potential confounders, and effect modifiers. Give diagnostic criteria, if applicable	Data collection, Imaging data, Cognitive and Emotional data, Statistical analysis (pp.5-9)
Data sources/ measurement	8	For each variable of interest, give sources of data and details of methods of assessment (measurement). Describe comparability of assessment methods if there is more than one group	Data collection, Imaging data, Cognitive and Emotional data (pp.5-7)
Bias	9	Describe any efforts to address potential sources of bias	Statistical analysis (pp.7-9)
Study size	10	Explain how the study size was arrived at	Participants (pp.5-6)
Quantitative variables	11	Explain how quantitative variables were handled in the analyses. If applicable, describe which groupings were chosen and why	Statistical analysis (pp.7-9)
Statistical methods	12	(*a*) Describe all statistical methods, including those used to control for confounding	Statistical analysis (pp.7-9)
		(*b*) Describe any methods used to examine subgroups and interactions	
		(*c*) Explain how missing data were addressed	
		(*d*) If applicable, explain how loss to follow-up was addressed	
		(*e*) Describe any sensitivity analyses	

For peer review only

**Results**			
Participants	13	(a) Report numbers of individuals at each stage of study – e.g. numbers potentially eligible, examined for eligibility, confirmed eligible, included in the study, completing follow-up, and analyzed	Demographic characteristics (p.10) and Figure 1
		(b) Give reasons for non-participation at each stage	
		(c) Consider use of a flow diagram	
Descriptive data	14	(a) Give characteristics of study participants (e.g. demographic, clinical, social) and information on exposures and potential confounders	Demographic characteristics (p.10) and Table 1
		(b) Indicate number of participants with missing data for each variable of interest	
		(c) Summarize follow-up time (e.g., average and total amount)	*Not applicable*
Outcome data	15*	Report numbers of outcome events or summary measures over time	Results (pp.12-19)
Main results	16	(*a*) Give unadjusted estimates and, if applicable, confounder-adjusted estimates and their precision (e.g., 95% confidence interval). Make clear which confounders were adjusted for and why they were included	Results (pp.12-19), Tables 2-4, Supplements
		(*b*) Report category boundaries when continuous variables were categorized	Statistical analysis (p.8)
		(*c*) If relevant, consider translating estimates of relative risk into absolute risk for a meaningful time period	*Not applicable*
Other analyses	17	Report other analyses done – e.g. analyses of subgroups and interactions, and sensitivity analyses	Results (p.20), Supplements
**Discussion**			
Key results	18	Summarize key results with reference to study objectives	Discussion (pp.21-22)
Limitations	19	Discuss limitations of the study, taking into account sources of potential bias or imprecision. Discuss both direction and magnitude of any potential bias	Strengths and limitations (pp.23-25)
Interpretation	20	Give a cautious overall interpretation of results considering objectives, limitations, multiplicity of analyses, results from similar studies, and other relevant evidence	Findings in context, Potential consequences and implications (pp.21-23)
Generalizability	21	Discuss the generalizability (external validity) of the study results	Strengths and limitations (pp.23-25)
**Other information**			
Funding	22	Give the source of funding and the role of the funders for the present study and, if applicable, for the original study on which the present article is based	Funding (p.26)

For peer review only
